# Genetic Effects at Pleiotropic Loci Are Context-Dependent with Consequences for the Maintenance of Genetic Variation in Populations

**DOI:** 10.1371/journal.pgen.1002256

**Published:** 2011-09-08

**Authors:** Heather A. Lawson, Janet E. Cady, Charlyn Partridge, Jason B. Wolf, Clay F. Semenkovich, James M. Cheverud

**Affiliations:** 1 Washington University in St Louis, St Louis, Missouri, United States of America; 2 University of Bath, Bath, United Kingdom; The Wellcome Trust Centre for Human Genetics, University of Oxford, United Kingdom

## Abstract

Context-dependent genetic effects, including genotype-by-environment and genotype-by-sex interactions, are a potential mechanism by which genetic variation of complex traits is maintained in populations. Pleiotropic genetic effects are also thought to play an important role in evolution, reflecting functional and developmental relationships among traits. We examine context-dependent genetic effects at pleiotropic loci associated with normal variation in multiple metabolic syndrome (MetS) components (obesity, dyslipidemia, and diabetes-related traits). MetS prevalence is increasing in Western societies and, while environmental in origin, presents substantial variation in individual response. We identify 23 pleiotropic MetS quantitative trait loci (QTL) in an F_16_ advanced intercross between the LG/J and SM/J inbred mouse strains (Wustl:LG,SM-G16; n = 1002). Half of each family was fed a high-fat diet and half fed a low-fat diet; and additive, dominance, and parent-of-origin imprinting genotypic effects were examined in animals partitioned into sex, diet, and sex-by-diet cohorts. We examine the context-dependency of the underlying additive, dominance, and imprinting genetic effects of the traits associated with these pleiotropic QTL. Further, we examine sequence polymorphisms (SNPs) between LG/J and SM/J as well as differential expression of positional candidate genes in these regions. We show that genetic associations are different in different sex, diet, and sex-by-diet settings. We also show that over- or underdominance and ecological cross-over interactions for single phenotypes may not be common, however multidimensional synthetic phenotypes at loci with pleiotropic effects can produce situations that favor the maintenance of genetic variation in populations. Our findings have important implications for evolution and the notion of personalized medicine.

## Author Summary

We look at gene-by-diet and gene-by-sex interactions underlying natural variation in multiple metabolic traits mapping to the same regions of the genome in a mouse model. We find that the underlying genetic architecture of these traits is different in different sex and diet contexts. We further use expression data and whole-genome polymorphism data to identify compelling candidates for experimental follow-up. We use these results to examine theoretical evolutionary predictions about how variation in populations can be maintained. There has been much discussion of late on how to use evolutionary theory to inform medical genomics. Mouse models may be especially appropriate for bridging the divide between evolutionary and biomedical research, because they allow the study of the effects of natural alleles on normal variation and because human-mouse homology is well defined. Our study is unique in examining quantitative trait loci from both evolutionary and biomedical perspectives, and we highlight the complex connections of the traits comprising the metabolic syndrome and the evolutionary implications of their underlying genetic architecture. This is important for understanding disease etiology and is relevant to personalized medicine.

## Introduction

Metabolic syndrome (MetS) is an array of co-occurring disorders including dyslipidemia, high blood pressure, impaired glucose tolerance, and obesity. Individuals diagnosed with MetS have increased risk of developing cardiovascular disease (CVD) and type-2 diabetes (T2D) [1]. MetS prevalence currently exceeds 20% in the United States and is increasing in developing countries [2]. This increase is hypothesized to be the result of over-consumption of high-caloric foods in conjunction with sedentary lifestyles [3]. There is also a genetic component as individual responses to dietary environment and to lifestyle modifications vary [1], [4]. Understanding MetS etiology is challenging because phenotypic variation is caused by complex interactions of many genes of small effects, by environmental factors, and by gene-by-environment interactions [5]–[9]. Thus animal models are valuable because genetic and environmental influences can be controlled for and monitored in populations of known genetic structure [10].

Mouse models have made major contributions to our understanding of complex disease etiology, including hypertension, obesity, and T2D [11]–[15]. However, MetS *per se* is not well defined in mice because the physiological features of individual components vary between mice and humans, reflecting 65–85 million years of divergent evolution [16]–[19]. Nevertheless, mouse models have increased our understanding of the pathophysiology of metabolic disorders and genes with robust effects have been identified using both spontaneous (e.g. *ob/ob* mice) and transgenic models. Further understanding of MetS will come from interrogating genes with small allelic effects on physiological processes, the source of variation in complex traits relevant to evolution and biomedicine, rather than from single genes with large defects.

We present results of a study of loci associated with normal variation in multiple MetS components: obesity (fatpad and organ weights), serum lipid levels (cholesterol, triglycerides and free-fatty acids levels), and diabetes (serum insulin and glucose levels, and response to a glucose challenge) in an F_16_ generation of an Advanced Intercross Line (AIL) formed from the LG/J and SM/J inbred mouse strains (Wustl:LG,SM-G16). Variation in complex traits in LG/J x SM/J is due to many genes of small effect interacting with each other and with the environment. Quantitative trait loci (QTL) have previously been mapped for obesity, serum chemistries and growth-related phenotypes in crosses of these strains [20]–[25]. This study is the first to look at variation in multiple MetS components mapping to the same locus in a very advanced generation of the LG/J x SM/J AIL. Here we examine these MetS QTL under a systems biology framework, incorporating both biomedical and evolutionary perspectives.

We report additive and dominance genotypic effects in addition to parent-of-origin genomic imprinting effects. Parent-of-origin imprinting is defined as the unequal expression of maternally and paternally derived copies of an allele, and has been shown to affect variation in metabolic traits [26]–. We examine the context-dependency of these genetic effects – additive, dominance and imprinting – by examining response to high- and low-fat dietary treatments. Context-dependency, defined as genotype-by-environment and genotype-by-sex interactions [29] is a proposed mechanism by which genetic variation is maintained in populations [30]–[33]. We examine whether additive genotypic values for a given trait or trait combination change rank across different environments, which is consistent with a so-called ecological cross-over [34]. When different alleles are favored in different environments, selection can maintain genetic variation at the locus.

Another mechanism that can maintain genetic variation in a population is balancing selection at pleiotropic loci, those associated with variation in multiple phenotypes, with different dominance relations for the different traits, so-called differential dominance [22], [35]. When differential dominance is present, some linear combination of traits will display over- or underdominance, even when no single trait does. If directional selection occurs along these linear combinations, there is balancing selection on the locus and genetic variation will be maintained.

We examine context-dependent genetic effects and differential dominance at pleiotropic loci associated with MetS components. Patterns of pleiotropy are thought to reflect functional and developmental relationships among traits [36], and have been hypothesized to serve as potential constraints on adaptive evolution [37] as well as underlie correlated phenotypic responses to selection [38]. Although pleiotropy has long been proposed to be ubiquitous, few studies have measured enough traits in a focal population to analyze this aspect of genetic architecture [39]. Our results show that additive, dominance and parent-of-origin genomic imprinting genetic effects vary among diet, sex and diet-by-sex environments among metabolic traits mapping to the same locus. This indicates that context-dependency is an important aspect of pleiotropic connections among components of MetS, a result supported by recent work on the *foraging* gene in *Drosophila melanogaster* [40], [41]. Understanding these connections and their evolutionary implications is important for understanding disease etiology and is relevant to personalized medicine.

## Results

### Pleiotropic QTL

We identify 23 pleiotropic QTL associated with normal variation in two or more MetS components. Of these 23 loci, 12 pass genome-wide significance while 11 pass chromosome-wise significance. The average locus is associated with variation in 4 traits. The traits examined here show moderate to high genetic correlations among each other and are reported with their respective heritabilities in Ehrich *et al*. 2005 [22]. Fourteen loci (61%) are associated with both diabetes (glucose levels, glucose tolerance, and serum insulin) and obesity (fatpad and organ weights). Six loci (26%) are associated with both dyslipidemia (serum cholesterol, free-fatty acid and triglycerides levels) and obesity. Three loci (13%) are associated with adiposity (fatpad weight) and liver weight. Liver weight is moderately correlated with percent liver fat (r = 0.61) [42], and nonalcoholic fatty liver disease is strongly associated with MetS [43], [44].

Additive effects are found at 20 loci (87%), and dominance and imprinting effects are found at 21 loci (91%). On average, in cohorts showing additive effects, LL homozygotes have higher serum lipid levels (cholesterol, triglyceride, free-fatty acid) and heavier weights (fatpad and/or organ weights) but respond better to a glucose challenge (intra-peritoneal glucose tolerance test) than SS homozygotes. In cohorts showing dominance effects, the L allele is dominant to the S allele 52% of the time. In cohorts with dominance effects and no additivity, we find overdominance (heterozygotes have significantly higher genotypic values) 60% of the time and underdominance (heterozygotes have significantly lower genotypic values) 40% of the time. In cohorts showing parent-of-origin imprinting effects, 15% show maternal expression imprinting, 10% show paternal expression imprinting, 21% show polar dominance imprinting (no additive effects), and 54% show bipolar dominance imprinting (no additive or dominance effects) (Table S1). Description of the various parent-of-origin imprinting patterns is found in Wolf *et al*. (2008) [45]. High-fat fed males are the most commonly affected cohort for the organ weights and diabetes-related traits, and high-fat fed females are the most commonly affected cohort for the serum lipid levels and fatpad weights [24], [25], [46].

### Context-Dependency of Genetic Effects

Table S1 breaks down the context-dependency of the QTL reported here and lists candidate genes found in the intervals. The mean QTL support interval is ≈4.0 Mb and contains 39 genes, many previously associated with metabolic disorders. Some of these positional candidates show expression differences between LG/J and SM/J in liver and white-fat tissues (Tables S1, S2 and S3), and we have annotated SNPs between the two strains in both coding and noncoding DNA in these intervals (Table S4). For example, we find a highly significant QTL on chromosome 1, *DMetS1b*, associated with variation in both serum lipid levels and obesity. This region overlaps QTL previously associated with high-density lipoprotein cholesterol (HDL) levels in studies using multiple crosses of mouse [15], [25], [47], [48]. Additionally, this region was recently reported as associated with both cholesterol and free-fatty acid levels in LG/J x SM/J [25]. The current analysis reveals this region is also associated with variation in gonadal and total fat-pad weights.

**Figure 1:**
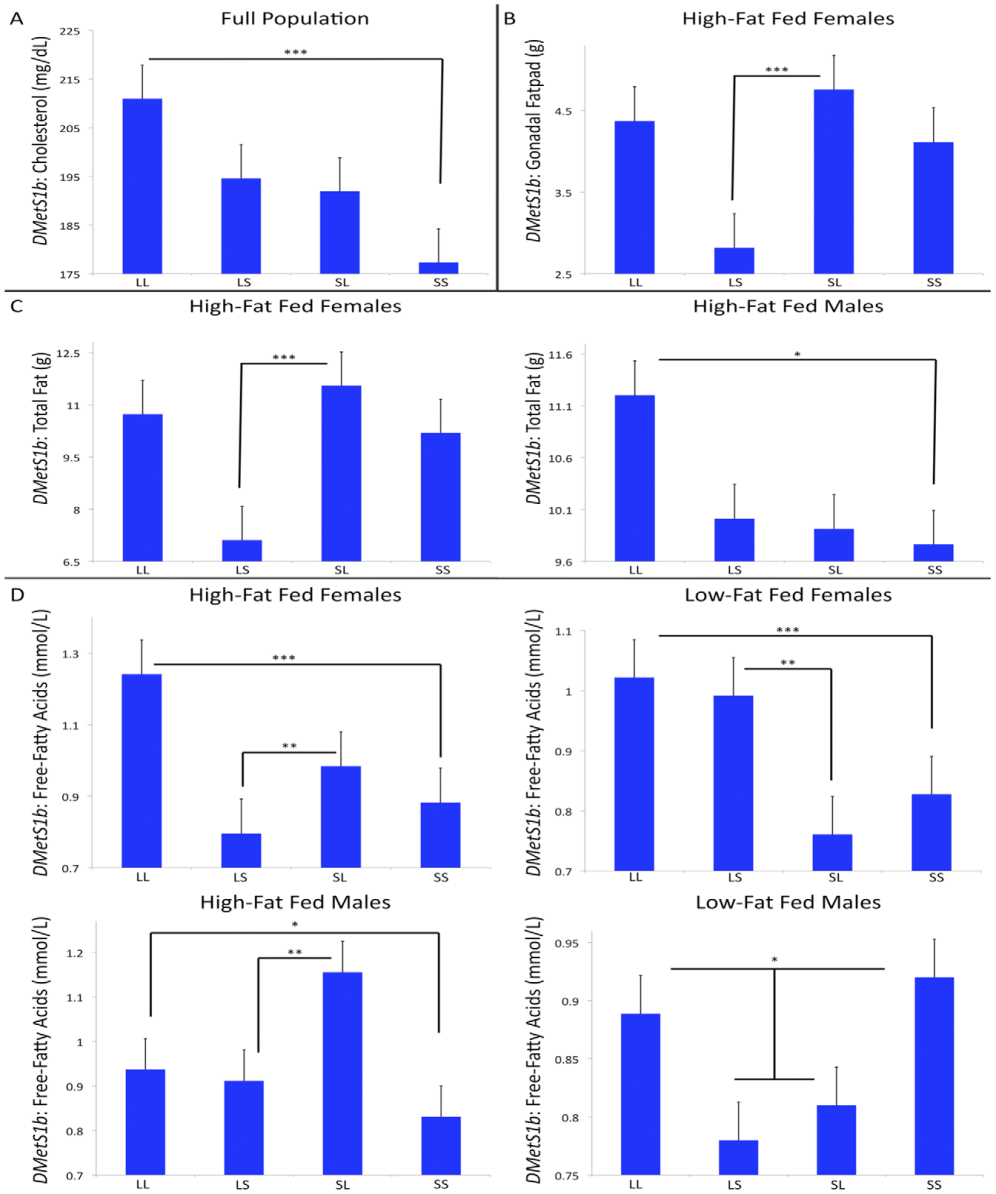
Context-dependent genetic effects at *DMetS1b*. Each single trait's genotypic value is represented on the y-axis, and the genotypes are represented on the x-axis. The different values reflect the different traits’ and cohorts’ different genotypic means. All of these traits map to the same genomic locus, however the cohort-specific genetic effects among these traits are highly context dependent, as illustrated in the patterns of the genotypic means. For cholesterol, there is an additive effect in the full population (A). For gonadal fatpad weight, high-fat fed females have bipolar dominance imprinting (B). For total fatpad weight, high-fat fed females have bipolar dominance imprinting and high-fat fed males have an additive effect (C). For free-fatty acid levels, in addition to additive effects, high-fat fed females have maternal expression imprinting, low-fat fed females have paternal expression imprinting, and high-fat fed males have polar dominance imprinting. Low fat-fed males have underdominance effects with no additive or imprinting effects (D). ***p≤0.001, **p≤0.01, *p≤0.05.

The genotypic effects at this QTL are complex (Figure 1a–1d). For cholesterol, there is an additive effect in the full population whereby individuals homozygous for the L allele have higher cholesterol. For free-fatty acid levels, in addition to this additive effect, high-fat fed females have maternal expression imprinting and low-fat fed females have paternal expression imprinting. High-fat fed males have polar dominance imprinting and low-fat fed males have underdominance effects with no significant additive or imprinting effects. For gonadal fatpad weight, high-fat fed females have bipolar dominance imprinting. For total fatpad weight, high-fat fed females have bipolar dominance imprinting and high-fat fed males have an additive effect.

This QTL spans 2.2Mb and contains 47 genes, 10 of which are candidates previously associated with metabolic disorders. Expression analysis of genes in this QTL show that in white-fat, 43 of these 47 genes are expressed in LG/J and SM/J, and 9 (21%) are significantly differentially expressed between the two strains. Three of these 9 genes, *F11r*, *Fcgr2b* and *Nr1i3*, are associated with variation in MetS components (Figure S1a-S1c and Table S2) [49]–[51]. In liver, 39 genes in the interval are expressed in LG/J and SM/J, and 10 (26%) of these genes are significantly differentially expressed between the two strains. Five of these 10 genes, *Apoa2*, *F11r*, *Hsd17b7*, *Nr1i3*, and *Usf1*, are MetS candidates (Figure S2a-S2e and Table S3) [47], [49]–[53].

There are 4,933 SNPs between LG/J and SM/J in *DMetS1b* (Table S4). Twenty–four of these SNPs are non-synonymous, and two of these non-synonymous SNPs (rs8258232 and rs8258226) fall in *Apoa2*. One of these SNPs, rs8258226, is the location of a mutation previously found to affect HDL cholesterol levels in multiple strains of mice [47]. The same Ala^61^ -to- Val^61^ substitution first identified by Wang *et al.* (2004) as the potential causal change underlying HDL variation is the same substitution found in LG/J. Many other *DMetS1b* SNPs, both in and around MetS candidates, fall within noncoding DNA having high regulatory potential [54].

### Differential Dominance

**Figure 2:**
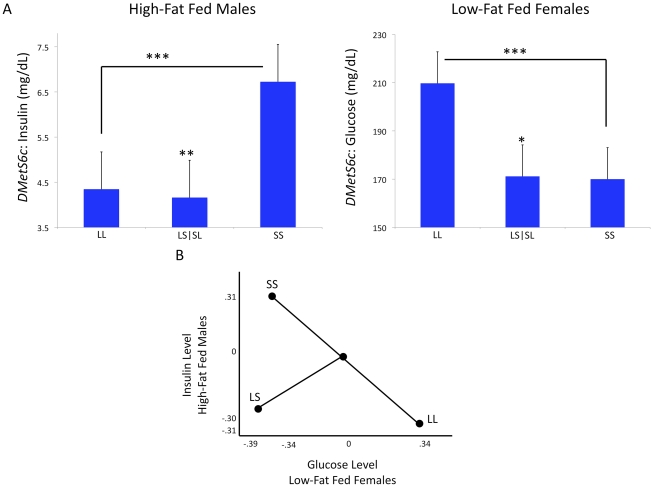
Differential dominance at *DMetS6c*. Variation in d/a ratios (dominance:additive genotypic values) between the two traits leads to differential dominance at this locus. Here the reciprocal heterozygotes have been pooled for simplicity because there are no significant imprinting effects at this locus. The two traits display antagonistic pleiotropy, meaning the additive genotypic values are significantly different between the two traits (A). Antagonistic pleiotropy is an example of a multidimensional synthetic crossing-over interaction, wherein the ranks of the homozygote genotypes change in different environments for some multivariate combination of traits. In this example the traits are serum glucose levels in low-fat fed females and serum insulin levels in high-fat fed males. In panel 2.B, the additive and dominance genotypic values for the homozygotes (LL and SS) and the heterozygotes (pooled LS and SL) have been standardized for graphical representation. The standardized insulin genotypic means in high-fat fed males are represented on the y-axis and the standardized glucose genotypic means in low-fat fed females are represented on the x-axis. The heterozygote values do not fall at the midpoint of the additive vector as expected under within-locus additivity. Directional selection on the orthogonal overdominance (represented by pooled LS and SL genotypic values) vector, for lower glucose and insulin levels, could result in balancing selection and maintenance of variation at this locus (B). ***p≤0.001, **p≤0.01, *p≤0.05.

Differential dominance is a property of pleiotropy that occurs when different traits mapping to the locus vary in the magnitude of their dominance ratios (*d/a*). Because the dominance ratios vary, the additive and dominance vectors are not colinear and some combination of traits will display over- or underdominance [35], [55], [56]. An example of differential dominance is found at a QTL on chromosome 6, *DMetS6c*. This locus is associated with variation in diabetes-related traits and liver weight (Table S1). The dominance ratios at *DmetS6c* differ between glucose levels in low-fat fed females (*d/a*  =  −0.9) and insulin levels in high-fat fed males (*d/a*  =  1.2). These two traits also display antagonistic pleiotropy, where glucose in the low-fat fed females has a significant positive additive genotypic value (LL homozygotes have higher levels) and insulin in the high-fat fed males has a significant negative additive genotypic value (SS homozygotes have higher levels) (Figure 2a-2b). Six loci (26%; *DMets2c, DMetS6b, DMetS6c, DMetS7c, DMetS10b, DMetS16a*) show differential dominance (Table S1).

### Gene-by-Environmental Interactions

**Figure 3:**
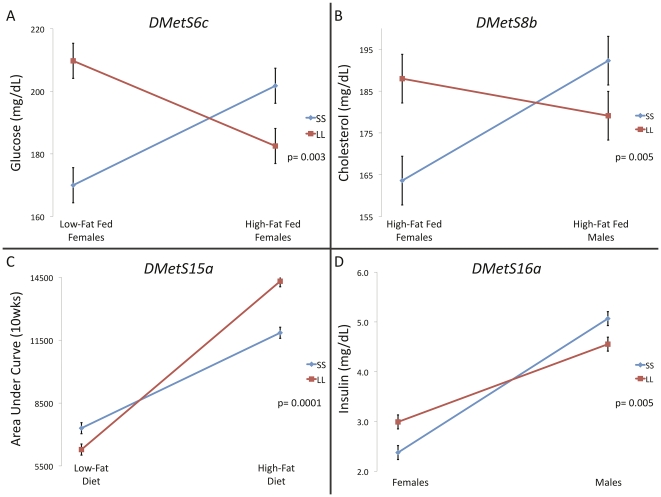
Crossing interactions at single traits comprising pleiotropic QTL. Significant crossing interactions, where the homozygote genotypic values change rank between environments (here diet and/or sex environment) for a trait, were found at 4 QTL. These genotype-by-environmental interactions, while rare, are consistent with an evolutionary ‘ecological cross-over’ hypothesis. Crossing interactions can act to maintain allelic variation at a locus.

Statistically significant interactions are of two types: ‘spreading’, where there is no difference between the genotypes in one sex and/or environment but a substantial difference in the alternate sex and/or environment, and ‘crossing’, where the rank order of allelic effects changes between sexes and/or environments [57]. Only crossing interactions can act to maintain allelic variation at a locus. We find 4 loci (17% of loci) having traits (6% of traits mapping to these loci) showing significant crossing interactions (*DMetS6c*, *DMetS8b*, *DMetS15a*, *DMetS16a*, Figure 3, Tables S1 and S7), indicating that with a few exceptions, the rank order of the homozygote genotypes remains the same in multiple environments for individual traits mapping to these loci. Three of the four crossing interactions occur in diabetes-related traits (glucose and insulin levels and area under the curve at 10wks), which have relatively lower genetic correlations among the traits mapped here [22]. This supports theoretical predictions that crossing genotype-by-environmental interactions would give rise to lower genetic correlations among traits [38].

## Discussion

Pleiotropic effects underlie genetic correlations among traits. Variation in pleiotropy is essential for selection to shape patterns of phenotypic covariation [58].

We identify 23 pleiotropic loci affecting normal variation in multiple components of MetS, including obesity-, T2D-, and CVD-related traits. We find additive, dominance and parent-of-origin imprinting effects are equally prevalent, highlighting the complex genetic architecture underlying these common/ complex traits that together characterize a syndrome. This result has implications for human genome-wide association studies (GWAS), which generally assume just additivity despite growing evidence for non-additive genetic effects on complex traits [59], [60]. Indeed a recent study identified parent-of-origin effects associated with T2D in the Icelandic genealogy database, finding the effects are different between males and females [61] and illustrating the complex connections among genomic sequence, genetic effects, environment, and disease risk.

Identifying these connections is a challenge in human population studies because recording or controlling an individual's environment over time is not possible, although some studies have successfully examined gene-by-environment interactions [5], [62]–[64]. We show that genotype interacts with environment in significant ways, and these interactions are not always consistent among genotypes, across environments, or across traits within the same population.

This is seen clearly at *DMetS1b* discussed above [25], [47], [65]. While multiple traits individually map to this locus (cholesterol, free-fatty acids, gonadal fatpad and total fatpad weight), the underlying genetic effects vary among the traits and are highly context dependent (Figure 1a–1d). We find the additivity is consistent among cohorts showing significant additive effects. However, when considering the heterozygotes, we find complex interactions between the L and S alleles in the different patterns of imprinting and dominance. For free-fatty acids, the females have opposite parent-of-origin imprinting effects depending on whether they were fed a high-fat (maternal expression imprinting) or low-fat (paternal expression imprinting) diet. If females were pooled together for analysis without considering dietary environment, no parent-of-origin effects would be detected because the two sex-by-diet cohorts' effects negate each other when combined. Further, for high-fat fed females, paternal inheritance of the L allele is protective for free-fatty acids, gonadal fatpad weight, and total fatpad. For gonadal fatpad and total fatpad weight, maternal inheritance of the L allele results in higher weight. Thus the same allele, in the same cohort, confers both protection and risk depending on parent-of-origin.

**Figure 4:**
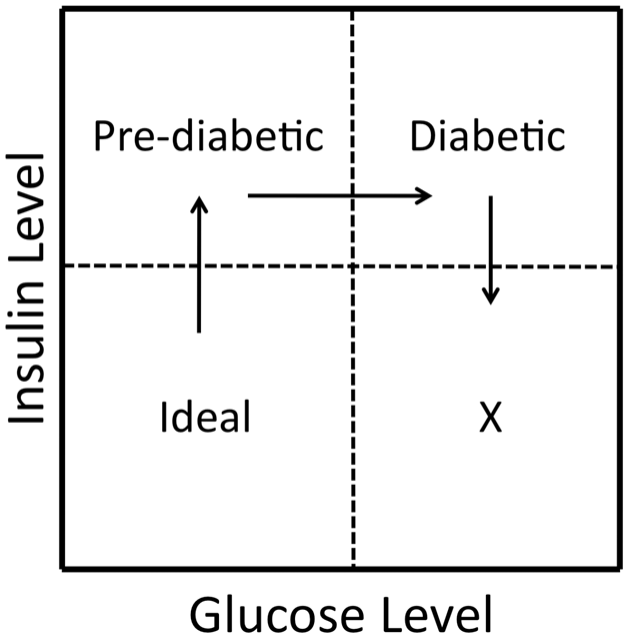
A scenario of fitness between traits at a pleiotropic locus. When considering differential dominance effects (as seen at *DMetS6c*) and the relationship not only among the genotypes, but also between insulin and glucose (as illustrated in Figure 2b), it is tempting to speculate that directional selection for the synthetic trait of “fitness” could result in balancing selection at a locus that would favor alleles contributing to low levels of both insulin and glucose. This is illustrated in the ‘Ideal’ quadrant representing relatively low levels of both insulin and glucose.

We find differential dominance among traits at some of these loci, for example at *DMets6c* discussed above. We acknowledge that our QTL contain multiple genes that may be tightly linked and may individually influence each trait mapped to the locus [66]. While our results are consistent with differential dominance occurring at some QTL, we do not have the resolution to test if this is due to multiple tightly linked genes. However, Keightley and Kascer showed that differential dominance is expected in systems in which nonlinearities are present, for example in the saturation and feedback inhibition systems of metabolic networks [67]. In this situation, some combination of traits will display under- or overdominance at the same locus, even in the absence of trait-specific under- or overdominance, a phenomenon called multivariate overdominance [55]. Directional selection on the synthetic phenotype could result in balancing selection at the locus and the maintenance of genetic variability [33]. At *DMetS6c*, directional selection for fitness could favor a genotype associated with lower levels of both insulin and glucose levels (Figure 4). It is tempting to speculate that maintenance of variability through interactions between alleles at a locus associated with metabolic traits could facilitate short-term adaptations to rapidly changing environments. While this issue is outside the scope of the current study, the results presented here can inform testing of evolutionary hypotheses such as The Thrifty Gene Hypothesis [68], under a systems biology framework, in an attempt to understand the determinants of the increasing prevalence of MetS and other disorders affecting metabolic homeostasis.

Genetic variation can also be maintained when the rank order of homozygous genotypes changes between environments. While most loci examined here do not show significant crossing interactions within the range of the experiment, we do see a few loci consistent with an ecological cross-over between sex and dietary environments for single traits (Figure 3). It is important to note, however, that an interaction between sexes is different than an interaction across environments. The evolutionary outcome of an ecological cross-over will depend on the frequency with which each environment is experienced. For sex it is generally ≈50∶50. Thus even in the absence of differential selection between the sexes, allelic variation will be maintained in a crossing scenario. For environmental interactions such as diet, allelic variation will be a function of the relative frequency of the environment experienced. Empirical evidence supporting the theory that heterogeneous environments produce crossing interactions is inconsistent [69], [70]. In this study, the genotypes for most of our single traits at these loci differ in magnitude between environments without significant crossing, a so-called spreading interaction. However, the QTL examined here are pleiotropic and multivariate combinations of traits may exhibit rank order changes in heterogeneous environments. Consider, for example, the antagonistic pleiotropy and hence rank order change of homozygous genotypes seen between glucose levels in low-fat fed females and insulin levels in high-fat fed males at *DMetS6c* (Figure 2a–2b). Such a multidimensional synthetic interaction is consistent with the complex nonlinear connection of the traits comprising the MetS, and remains an open question for further exploration [14].

Once a genomic association is made, examination of the QTL can lead to identification of the quantitative trait gene (QTG) and eventually the quantitative trait nucleotide (QTN) affecting variation in the trait. We acknowledge that our QTL may contain multiple QTG and QTN, even when we fail to reject pleiotropy. We have identified candidate QTGs in our QTL regions, both from the literature and by examining differential expression between LG/J and SM/J in relevant tissues. We further identified QTN for experimental follow-up by examining SNPs between the strains both in and around QTGs. We present many fruitful regions for follow-up, including some novel positional candidate genes, for example *Cacna7a* located in *DMetS8b*, which is associated with normal variation in obesity and cholesterol levels. This gene encodes the pre-forming A_1A_ subunit of voltage-gated calcium channels and has been found to influence the functionality of cholesterol-rich microdomains [71]. It is differentially expressed between LG/J and SM/J in both liver and white-fat and contains many SNPs in non-coding flanking and intronic regions having high-regulatory potential. This gene is associated with chronodisruption, the desynchronization of circadian rhythms [72], and with migraine headaches [73]. Recent research demonstrates an association among chronodisruption, migraine, and MetS components [74]–[76].

Another attractive locus for follow-up is the *Apoa2* gene, which falls in *DMetS1b*. Variations in the homologous human APOA2 sequence have been well studied for association with MetS components in humans [77], [78]. We not only find *Apoa2* is differentially expressed between LG/J and SM/J, but also identify a non-synonomous coding SNP (rs8258226) that has been independently associated with elevated cholesterol levels in multiple other strains of mice [47]. While this result is encouraging proof-of-principle in going from QTL → QTG → QTN, further experimentation is required to know if this mutation, let along this gene, is associated with variation in the other traits mapping to this locus. Indeed it has been found that, when a single locus is associated with multiple traits, different polymorphisms within the locus are independently associated with the various traits [29], [79]. So while the QTL is pleiotropic, the pleiotropy breaks down at the nucleotide level.

Overall, we find the genetic effects at these 23 QTL are highly context-dependent and are not consistent among the individual traits mapped. Our results indicate that if context such as sex and/or diet are not considered, not only can genetic signals in specific cohorts be masked and/or cancelled out in an aggregate study population, but also genetic effects can be erroneously assigned to specific cohorts within a population if the effects are pooled over all its members. We have shown that the genetic architecture underlying the individual traits mapping to these QTL is complicated, and so are the relationships among the traits themselves. Further, some patterns are consistent with evolutionary theory with respect to the maintenance of genetic variation in populations, even when specific variants are deleterious in particular environments or in particular combinations. While over- or underdominance and crossing interactions for single phenotypes may not be common, multidimensional synthetic phenotypes at QTL with pleiotropic effects can produce situations that favor the maintenance of genetic variation in populations. As Lewontin ([80]; p318) noted, “Context and interaction are of the essence”.

Gluckman *et al.* [81] recently discussed the challenges associated with understanding human biology in light of the current epidemic of metabolic disorders, and Sing *et al*. [6] proposed a series of steps a researcher should take to address issues of complex disease etiology. As the era of personalized medicine and individual whole-genome sequencing looms, it is important to keep in mind the ultimate goal of developing treatments and prevention strategies for *individuals*. For MetS, this goal may be attained through understanding the underlying genetic architecture of its disease components, of how these components relate to each other evolutionarily, and in what context. Mouse models may be especially appropriate for bridging the divide between evolutionary and biomedical research because they allow the study of the effects of natural alleles on normal variation, and human-mouse homology is well defined. Our results are important because they can be used to elucidate gene-by-environmental effects that could inform large-scale genomic study design in humans.

## Materials and Methods

### Ethics Statement

Our study involved mice and all animal care and handling procedures conformed to IACUC guidelines.

### Population

The LG/J x SM/J Advanced Intercross Line (AIL) is managed as a pseudo-randomly mated line starting from the F_2_ generation. The LG/J strain originated from a selection experiment for large body size at 60 days and the SM/J strain originated from a selection experiment for small body size at 60 days [82]. Animals from each strain have been inbred by brother-sister mating for over 150 generations making them genetically homozygous with the exception of spontaneous mutations and the *agouti* locus in SM/J which is maintained heterozygous (*a/A^w^*) for breeding purposes [83], [84].

The AIL was generated from an initial cross of 10 male SM/J mice and 10 female LG/J mice. Animals are randomly mated but brother-sister mating is not allowed. Only one male and one female are chosen from each family as breeders for the next generation, thereby eliminating variation in familial contributions to the next generation. This is an effective method of reducing inbreeding and doubling the effective population size of a colony relative to its census size [85]. The average number of breeding pairs in the AIL is 75, giving a census size of 150 and an effective population size of approximately 300 individuals.

This study used an experimental F_16_ population of 1,002 animals in 76 sibships, each averaging 6.8 animals. Animal husbandry details can be found in Ehrich *et al*. 2005 [86]. At weaning, males and females from each litter were partitioned into cohorts fed either high-fat (253 males; 248 females) or low-fat (247 males; 254 females) diets. The diets were isocaloric with the exception of calories from fat (Harlan Teklad cat. No. TD88137, 42% energy from fat; and Research Diets cat. No. D12284, 15% energy from fat, specially formulated; Table 1).

**Table 1:** Composition of the high- and low-fat diets used in this experiment.

Source	High-fat	Low-fat
Energy from fat	42%	15%
Casein (g/kg)	195	197
Sugars (g/kg)	341	307
Corn starch (g/kg)	150	313
Cellulose (g/kg)	50	30
Corn Oil (g/kg)	--	58
Hydrogenated coconut oil (g/kg)	--	7
Anhydrous milkfat (g/kg)	210	--
Cholesterol (g/kg)	1.5	--
Total energy (kJ/g)	18.95	16.99

### Phenotypes

Animals were weighed weekly for 20 weeks. A subset of animals (217 females, 113 fed the low-fat diet and 104 fed the high-fat diet; 213 males, 103 fed the low-fat diet and 110 fed the high-fat diet) were subject to an intra-peritoneal glucose tolerance test (IPGTT) at 10 and 20 weeks of age as described in Ehrich *et al*. 2005 [86]. Readings taken over the course of 2 hours were used to calculate the area under the curve (AUC), a measure of glucose tolerance. Animals were necropsied at 20 weeks of age (also described in Ehrich *et al*. 2005) and fasting (4 hr) serum cholesterol, free-fatty acid, triglyceride, glucose, and insulin were obtained from blood via cardiac puncture. Serum was frozen at −20°C until assayed by the Nutrition Obesity Research Center – Animal Model Research Core at Washington University. Additionally, fat pads (inguinal, mesenteric, renal and gonadal) and internal organs (heart, kidneys, liver, and spleen) were removed and weighed.

### Genotypes

DNA was extracted from liver tissue using the QIAGEN kit. 1,536 single nucleotide polymorphisms (SNPs) were chosen from the CTC/Oxford SNP survey (www.well.ox.ac.uk/mouse/INBREDS/) and scored with the Illumina Golden Gate Bead Array. Genotyping was performed at the Washington University Genome Sequencing and Analysis Center. 1,402 autosomal SNPs were reliably scored and used in this study (Table S5).

A genetic map was created based on physical order of the SNPs along the autosomes (mm9; NCBI build 37). Recombination fractions were estimated using R/qtl [87]. Ordered genotypes were reconstructed at each marker from familial SNP data (F_15_ parents and their F_16_ offspring) using the Integer Linear Programming algorithm as implemented in PedPhase 2.1 [88]. Due to the computational intensity of the algorithm, it was necessary to partition the larger chromosomes before running the program. Additive (X_a_) and dominance (X_d_) genotypic scores were assigned at each marker: X_a_  =  1, 0, −1 and X_d_  =  0, 1, 0 for the LL, LS and SL, and SS genotypes, respectively. ‘L’ refers to an allele derived from the LG/J strain and ‘S’ refers to an allele derived from the SM/J strain. Further, we assigned parent-of-origin imprinting genotypic scores (X_i_) to distinguish between reciprocal heterozygotes, LS and SL. By convention the first allele refers to that inherited from the father and the second from the mother. Imprinting genotypic scores for LL, LS, SL, and SS are X_i_  =  0, +1, −1, 0, respectively [89]. Additional genotypes were imputed at 1cM intervals between the markers using the equations of Haley and Knott [90] with the inclusion of equations derived for imputing imprinting genotypic scores [91].

### QTL Mapping

Single locus analyses were performed using maximum likelihood in the Mixed Procedure in SAS 9.2. Our full mapping included: sex, diet, sex-by-diet interaction, the direct effects of the genomic locations (X_a_, X_d_, X_i_), and their two- and three-way interactions with sex and diet as fixed effects. The full model explains variation in trait (Y) using the linear equation:
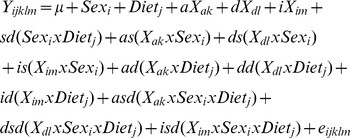
where µ is the population mean and e is the residual. The regression coefficients are the additive [a  =  (G_LL_)−(G_SS_))/2], dominance [d  =  ((G_LS_+G_SL_)−(G_LL_-G_SS_))/2] and imprinting [i  =  (G_LS_−G_SL_)/2] genotypic scores, where G refers to the mean phenotype of all individuals sharing the subscripted genotype, and their interactions with sex (s) and/or with diet (d). Family and its interactions with sex and diet, including the three-way interaction, were included as random effects in the model. The −2 ln(likelihood) of this model was compared to a null model including only sex, diet and sex-by-diet interaction terms using a chi-square test with 12 df. Probabilities were transformed into LOD  =  −log_10_(Pr).

The number of independent tests was calculated using the Li and Ji method based on the eigenvalues of the correlation matrix of marker additive genotype scores [92]. This was used to calculate Bonferroni adjusted significance thresholds, 1−(1−α)^1/M^, where M is the number of independent tests. A significance threshold was calculated at the genome-wide level (LOD ≥3.90) as well as separately for each autosome (Table S6). With chromosome-wise significance, we expect 1 false positive result per trait. Our results overwhelm this in that there are 6–10 times the number of significant results for each trait as expected by chance under a null model of no QTL. Further, QTL with chromosome-wise significance have a history of replication across different mapping populations of this cross [24].

### Pleiotropy

QTL for separately analyzed traits related to two or more metabolic syndrome (MetS) components mapping to the same cM position are considered pleiotropic QTL. When QTL support intervals for separately analyzed MetS component traits overlapped, but the separate trait peaks did not map to the same cM position, a formal test of pleiotropy was performed as described by Cheverud [36]. First, the most likely peak QTL positions for each single trait were identified, e.g. the position with the highest LOD score, and then the most likely combined position of the all the traits mapping to the region, weighted by their LOD scores, was identified. A X^2^ for model fit was obtained at each single trait peak and at the combined-trait position. The differences in X^2^ values between the separate and the combined-trait models were added together to generate a X^2^ test for pleiotropy [34]. The degrees of freedom were determined by the number of positions (corresponding to the number of traits) in the separate model minus the single position of the combined model. The null hypothesis is that the combined model fits the data better and there is most likely a single pleiotropic QTL. Because it is statistically simpler to have 1 QTL rather than multiple QTL, a significant result (Prob(X^2^) ≤ 0.05) indicates there is most likely more than one QTL in the region (hence rejecting the null hypothesis of pleiotropy). Support intervals were determined using a standard one-LOD drop from the highest LOD at the combined peak of the QTL [93] (Table S1).

### Crossing Analysis

The genotypic values for each cohort were obtained at QTL showing significant additive*sex, additive*diet, and/or additive*sex*diet interactions. When the genotypic values for individuals with LL and SS genotypes were of opposite ranks between two cohorts showing significant additive effects, a formal test for crossing interactions was run. The effect of differential dispersion of trait means in different cohorts was removed by standardizing the variance in trait means for each cohort by the following equation:
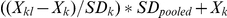
where X_kl_ is the trait value for the l^th^ individual of the k^th^ cohort. X_k_ and SD_k_ are the mean and standard deviation of the k^th^ cohort, and SD_pooled_ is the average SD over all cohorts. This standardization removes the effect of differential dispersion of cohorts' means, hence eliminating the differences in the scale of the genotypic effects and leaving only the changes in the order of the genotypic means as the source of the interaction [57], [94]. These transformed trait values were analyzed by the mixed model in SAS as described above. If the significant additive*sex, additive*diet, and/or additive*sex*diet interaction effects remained at the QTL, and the genotypic values for individuals with LL and SS genotypes were of opposite ranks between cohorts, the interaction was considered to be ‘crossing’ and not ‘spreading’ (Table S7).

### Expression Data

At necropsy, liver and gonadal fat pads (white fat) were collected from 4 males and 4 females representing each strain and diet. Tissue was immediately frozen in liquid nitrogen and stored at −80°C until extraction. RNA was extracted using RNeasy 96 Universal Tissue extraction kits (Qiagen, Valencia, CA), and quantified using a Nanodrop 2000 (Thermo Scientific, Wilmington, DE). Samples were submitted to the Washington University Microarray Core Facility, where quality was assessed using a 2100 Bioanalyzer (Agilent Tecnologies, Palo Alto, CA). RNA was reverse transcribed and amplified using an Illumina TotalPrep amplification kit (Ambion, Austin, TX) and then hybridized onto Illumina WG-6 v.2 BeadChips (Illumina, San Diego, CA). Arrays were scanned using the Illumina Beadstation 500, and images processed using Illumina BeadScan software. Intensity values were analyzed using Illumina BeadStudio.

Illumina raw data from 45,281 unique probes were examined using LUMI [95]. Data were transformed using a variance stabilization transformation [96], which takes into account the large number of technical replicates on Illumina arrays, and normalized using a robust spline normalization. Genes showing no significant expression were filtered from the data set prior to analysis, leaving 26,209 transcripts analyzed for the liver and 29,285 for white fat. The data were analyzed using Partek Genomics software v6.5 (Partek Incorporated, St. Louis, MO). Significant differences in gene expression were assessed using a 3-factor ANOVA, testing for the main effects of diet, of sex, of strain and of their interactions and correcting for multiple tests using the False Detection Rate approach. To correct for multiple tests within these focal regions, q-values were generated for genes falling in the 23 MetS support intervals using QVALUE (method  =  bootstrap)[97] (Tables S2 and S3).

### Whole-Genome Polymorphism Data

Whole-genome sequencing for the LG/J (≈20X haploid coverage) and the SM/J (≈14X haploid coverage) inbred mouse strains was completed by the Washington University School of Medicine Genome Sequencing Center using Illumina sequencing in two steps as described in Mardis et al. [98] and Ding et al. [99]. The reference genome used was the July 2007 assembly NCBI build37(mm9). Illumina reads from liver tissue from a single LG/J female and a single SM/J female were aligned to the reference genome using MAQ [100].

High-quality SNPs for each strain were called using SamTools (http://samtools.sourceforge.net/) [101], requiring three or more reads and a SNP quality score ≥ 20. We identified 4,406,015 high-confidence polymorphisms between LG/J and SM/J. These polymorphisms were annotated with custom Python programs using RefSeq [102] coordinates downloaded from the UCSC Genome Browser [103] accessed May 2010. The LG/J and SM/J whole-genome SNPs have been submitted to dbSNP [104] for public use under the handle “Cheverud”.

## Supporting Information

Figure S1 Expression differences in white fat between LG/J and SM/J for positional candidate genes in *DMetS1b*. Three of 9 genes in *DMetS1b* that are significantly differentially expressed between LG/J and SM/J in white fat are associated with MetS components. *F11r* is involved in lesion formation in atherosclerosis-prone mice [49] and shows higher levels of expression in LG/J. *Fcgrb2* influences atherosclerosis in apoE(-/-) male mice [50] and shows higher expression levels in SM/J. *Nr1i3* is involved in lipid homeostasis [51] and shows differential expression in a strain-by-diet interaction, where individuals fed a low-fat diet have higher expression levels in the LG/J strain and individuals fed a high-fat diet have higher expression levels in the SM/J strain.
(TIF)

Figure S2 Expression differences in liver between LG/J and SM/J for positional candidate genes in *DMetS1b*. Five of 10 genes that are significantly differentially expressed between LG/J and SM/J in liver are associated with MetS components. *F11r* is also differentially expressed in white fat, and in both tissues the LG/J strain shows the highest levels of expression. *Nr1i3* is differentially expressed in a strain-by-dietary context in white fat, and shows significantly higher expression levels in LG/J in liver. *Apoa2* affects cholesterol levels [47] and shows higher expression in LG/J. *Hsd17b7* is involved in fetal cholesterol synthesis [52] and shows higher expression levels in LG/J. *Usf1* is associated with blood serum lipid levels and MetS [53], and has higher levels of expression in SM/J.
(TIF)

Table S1 Pleiotropic QTL affecting MetS components, their interactions and MetS positional candidate genes.
(DOC)

Table S2 Differential expression in MetS QTL among genes that are expressed in LG/J and SM/J strains in white fat tissue.
(DOC)

Table S3 Differential expression in MetS QTL among genes that are expressed in LG/J and SM/J strains in liver tissue.
(DOC)

Table S4 Number of SNPs between LG/J and SM/J in both coding and non-coding sequence in MetS QTL.
(DOC)

Table S5 Markers used for QTL mapping in the F16 LG/J x SM/J Advanced Intercross.
(DOC)

Table S6 Chromosome-wise and genome-wide significance thresholds.
(DOC)

Table S7 Significant crossing interactions at MetS QTL.
(DOC)

## References

[1] Cornier MA, Dabelea D, Hernandez TL, Lindstrom RC, Steig AJ (2008). The metabolic syndrome.. Endocr Rev.

[2] Abegunde DO, Mathers CD, Adam T, Ortegon M, Strong K (2007). The burden and costs of chronic diseases in low-income and middle-income countries.. Lancet.

[3] Heymsfield SB (2009). How large is the energy gap that accounts for the obesity epidemic?. Am J Clin Nutr.

[4] Musani SK, Erickson S, Allison DB (2008). Obesity--still highly heritable after all these years.. Am J Clin Nutr.

[5] Nettleton JA, Steffen LM, Ballantyne CM, Boerwinkle E, Folsom AR (2007). Associations between HDL-cholesterol and polymorphisms in hepatic lipase and lipoprotein lipase genes are modified by dietary fat intake in African American and White adults.. Atherosclerosis.

[6] Sing CF, Stengard JH, Kardia SL (2003). Genes, environment, and cardiovascular disease.. Arterioscler Thromb Vasc Biol.

[7] Buchanan AV, Weiss KM, Fullerton SM (2006). Dissecting complex disease: the quest for the Philosopher's Stone?. Int J Epidemiol.

[8] Klos KL, Kardia SL, Hixson JE, Turner ST, Hanis C (2005). Linkage analysis of plasma ApoE in three ethnic groups: multiple genes with context-dependent effects.. Ann Hum Genet.

[9] Grove ML, Morrison A, Folsom AR, Boerwinkle E, Hoelscher DM (2007). Gene-environment interaction and the GNB3 gene in the Atherosclerosis Risk in Communities study.. Int J Obes (Lond).

[10] Lusis AJ, Attie AD, Reue K (2008). Metabolic syndrome: from epidemiology to systems biology.. Nat Rev Genet.

[11] Lawson HA, Cheverud JM (2010). Metabolic syndrome components in murine models.. Endocr Metab Immune Disord Drug Targets.

[12] Clee SM, Yandell BS, Schueler KM, Rabaglia ME, Richards OC (2006). Positional cloning of Sorcs1, a type 2 diabetes quantitative trait locus.. Nat Genet.

[13] Bennett BJ, Farber CR, Orozco L, Kang HM, Ghazalpour A (2010). A high-resolution association mapping panel for the dissection of complex traits in mice.. Genome Res.

[14] Stoehr JP, Nadler ST, Schueler KL, Rabaglia ME, Yandell BS (2000). Genetic obesity unmasks nonlinear interactions between murine type 2 diabetes susceptibility loci.. Diabetes.

[15] Wang X, Paigen B (2002). Quantitative trait loci and candidate genes regulating HDL cholesterol: a murine chromosome map.. Arterioscler Thromb Vasc Biol.

[16] Foote M, Hunter JP, Janis CM, Sepkoski JJ (1999). Evolutionary and preservational constraints on origins of biologic groups: divergence times of eutherian mammals.. Science.

[17] Lander ES, Linton LM, Birren B, Nusbaum C, Zody MC (2001). Initial sequencing and analysis of the human genome.. Nature.

[18] Waterston RH, Lindblad-Toh K, Birney E, Rogers J, Abril JF (2002). Initial sequencing and comparative analysis of the mouse genome.. Nature.

[19] Polotsky VY (2007). Mouse model of the metabolic syndrome: the quest continues.. J Appl Physiol.

[20] Cheverud JM, Ehrich TH, Hrbek T, Kenney JP, Pletscher LS (2004). Quantitative trait loci for obesity- and diabetes-related traits and their dietary responses to high-fat feeding in LGXSM recombinant inbred mouse strains.. Diabetes.

[21] Kenney-Hunt JP, Vaughn TT, Pletscher LS, Peripato A, Routman E (2006). Quantitative trait loci for body size components in mice.. Mamm Genome.

[22] Ehrich TH, Kenney-Hunt JP, Pletscher LS, Cheverud JM (2005). Genetic variation and correlation of dietary response in an advanced intercross mouse line produced from two divergent growth lines.. Genet Res.

[23] Fawcett GL, Jarvis JP, Roseman CC, Wang B, Wolf JB (2009). Fine-Mapping of Obesity-Related Quantitative Trait Loci in and F9/10 Advanced Intercross Line..

[24] Cheverud JM, Lawson HA, Fawcett GL, Wang B, Pletscher LS (2010). Diet-Dependent Genetic and Genomic Imprinting Effects on Obesity in Mice..

[25] Lawson HA, Zelle KM, Fawcett GL, Wang B, Pletscher LS (2010). Genetic, epigenetic, and gene-by-diet interaction effects underlie variation in serum lipids in a LG/J x SM/J murine model..

[26] Xie T, Chen M, Gavrilova O, Lai EW, Liu J (2008). Severe obesity and insulin resistance due to deletion of the maternal Gsalpha allele is reversed by paternal deletion of the Gsalpha imprint control region.. Endocrinology.

[27] Rampersaud E, Mitchell BD, Naj AC, Pollin TI (2008). Investigating parent of origin effects in studies of type 2 diabetes and obesity.. Curr Diabetes Rev.

[28] Weinstein LS, Xie T, Qasem A, Wang J, Chen M (2009). The role of GNAS and other imprinted genes in the development of obesity..

[29] Mackay TF, Stone EA, Ayroles JF (2009). The genetics of quantitative traits: challenges and prospects.. Nat Rev Genet.

[30] Burger R, Gimelfarb A (2002). Fluctuating environments and the role of mutation in maintaining quantitative genetic variation.. Genet Res.

[31] Gillespie JH, Turelli M (1989). Genotype-environment interactions and the maintenance of polygenic variation.. Genetics.

[32] Mackay TF (1981). Genetic Variation in Varying Environments.. Genetical Research.

[33] Turelli M, Barton NH (2004). Polygenic variation maintained by balancing selection: pleiotropy, sex-dependent allelic effects and G x E interactions.. Genetics.

[34] Lynch M, Walsh B (1998). Genetics and Analysis of Quantitative Traits..

[35] Kenney-Hunt JP, Cheverud JM (2009). Differential dominance of pleiotropic loci for mouse skeletal traits.. Evolution.

[36] Cheverud JM, Wagner GP (2001). The Genetic Architecture of Pleiotropic Relations and Differential Epistasis.. The Character Concept in Evolutionary Biology.

[37] Wagner GP (1988). The Gene and its Phenotype.. Biology and Philosophy.

[38] Falconer DS, Mackay TF (1996). Introduction to Quantitative Genetics..

[39] Wright S (1980). Genic and Organismic Selection.. Evolution.

[40] Kent CF, Daskalchuk T, Cook L, Sokolowski MB, Greenspan RJ (2009). The Drosophila foraging gene mediates adult plasticity and gene-environment interactions in behaviour, metabolites, and gene expression in response to food deprivation.. PLoS Genet.

[41] Houle D, Govindaraju DR, Omholt S (2010). Phenomics: the next challenge.. Nat Rev Genet.

[42] Minkina O, Kenney-Hunt J, Fawcett G, Semenkovich CF, Cheverud JM Quantitative trait loci affecting liver fat content in mice..

[43] Almeda-Valdes P, Cuevas-Ramos D, Aguilar-Salinas CA (2009). Metabolic syndrome and non-alcoholic fatty liver disease.. Ann Hepatol.

[44] Fabbrini E, Sullivan S, Klein S (2010). Obesity and nonalcoholic fatty liver disease: biochemical, metabolic, and clinical implications.. Hepatology.

[45] Wolf JB, Cheverud JM, Roseman C, Hager R (2008). Genome-wide analysis reveals a complex pattern of genomic imprinting in mice.. PLoS Genet.

[46] Lawson HA, Lee A, Fawcett GL, Pletscher LS, Maxwell TJ (2011). The importance of context to the genetic architecture of diabetes-related traits is revealed in a genome-wide scan of a LG/JxSM/J murine model..

[47] Wang X, Korstanje R, Higgins D, Paigen B (2004). Haplotype analysis in multiple crosses to identify a QTL gene.. Genome Res.

[48] Su Z, Cox A, Shen Y, Stylianou IM, Paigen B (2009). Farp2 and Stk25 are candidate genes for the HDL cholesterol locus on mouse chromosome 1.. Arterioscler Thromb Vasc Biol.

[49] Zernecke A, Liehn EA, Fraemohs L, von Hundelshausen P, Koenen RR (2006). Importance of junctional adhesion molecule-A for neointimal lesion formation and infiltration in atherosclerosis-prone mice.. Arterioscler Thromb Vasc Biol.

[50] Mendez-Fernandez YV, Stevenson BG, Diehl CJ, Braun NA, Wade NS (2011). The inhibitory FcgammaRIIb modulates the inflammatory response and influences atherosclerosis in male apoE(-/-) mice.. Atherosclerosis.

[51] Masson D, Qatanani M, Sberna AL, Xiao R, Pais de Barros JP (2008). Activation of the constitutive androstane receptor decreases HDL in wild-type and human apoA-I transgenic mice.. J Lipid Res.

[52] Jokela H, Rantakari P, Lamminen T, Strauss L, Ola R (2010). Hydroxysteroid (17beta) dehydrogenase 7 activity is essential for fetal de novo cholesterol synthesis and for neuroectodermal survival and cardiovascular differentiation in early mouse embryos.. Endocrinology.

[53] Singmann P, Baumert J, Herder C, Meisinger C, Holzapfel C (2009). Gene-gene interaction between APOA5 and USF1: two candidate genes for the metabolic syndrome.. Obes Facts.

[54] Taylor J, Tyekucheva S, King DC, Hardison RC, Miller W (2006). ESPERR: learning strong and weak signals in genomic sequence alignments to identify functional elements.. Genome Res.

[55] Klingenberg CP, Leamy LJ, Routman EJ, Cheverud JM (2001). Genetic architecture of mandible shape in mice: effects of quantitative trait loci analyzed by geometric morphometrics.. Genetics.

[56] Ehrich TH, Vaughn TT, Koreishi SF, Linsey RB, Pletscher LS (2003). Pleiotropic effects on mandibular morphology I. Developmental morphological integration and differential dominance.. J Exp Zool B Mol Dev Evol.

[57] De Brito RA, Pletscher LS, Cheverud JM (2005). The evolution of genetic architecture. I. diversification of genetic backgrounds by genetic drift.. Evolution.

[58] Pavlicev M, Kenney-Hunt JP, Norgard EA, Roseman CC, Wolf JB (2008). Genetic variation in pleiotropy: differential epistasis as a source of variation in the allometric relationship between long bone lengths and body weight.. Evolution.

[59] Maher B (2008). Personal genomes: The case of the missing heritability.. Nature.

[60] Hamon SC, Stengard JH, Clark AG, Salomaa V, Boerwinkle E (2004). Evidence for non-additive influence of single nucleotide polymorphisms within the apolipoprotein E gene.. Ann Hum Genet.

[61] Kong A, Steinthorsdottir V, Masson G, Thorleifsson G, Sulem P (2009). Parental origin of sequence variants associated with complex diseases.. Nature.

[62] Junyent M, Parnell LD, Lai CQ, Arnett DK, Tsai MY (2009). ADAM17_i33708A>G polymorphism interacts with dietary n-6 polyunsaturated fatty acids to modulate obesity risk in the Genetics of Lipid Lowering Drugs and Diet Network study..

[63] Kabagambe EK, Glasser SP, Ordovas JM, Warodomwichit D, Tsai MY (2009). TCF7L2 polymorphisms and inflammatory markers before and after treatment with fenofibrate.. Diabetol Metab Syndr.

[64] Chatterjee N, Kalaylioglu Z, Moslehi R, Peters U, Wacholder S (2006). Powerful multilocus tests of genetic association in the presence of gene-gene and gene-environment interactions.. Am J Hum Genet.

[65] Stewart TP, Kim HY, Saxton AM, Kim JH (2010). Genetic and genomic analysis of hyperlipidemia, obesity and diabetes using (C57BL/6J x TALLYHO/JngJ) F2 mice.. BMC Genomics.

[66] Bigham ET, Lamkey KR (1998). Role of chromosome blocks in heterosis and estimates of dominance and overdominance.. Concepts and Breeding Heterosis in Crop Plants.

[67] Keightley PD, Kacser H (1987). Dominance, pleiotropy and metabolic structure.. Genetics.

[68] Neel JV (1962). Diabetes mellitus: a “thrifty” genotype rendered detrimental by “progress”?. Am J Hum Genet.

[69] Yeaman S, Chen Y, Whitlock MC (2010). No effect of environmental heterogeneity on the maintenance of genetic variation in wing shape in Drosophila melanogaster.. Evolution.

[70] Ingleby FC, Hunt J, Hosken DJ (2010). The role of genotype-by-environment interactions in sexual selection.. J Evol Biol.

[71] Davies A, Douglas L, Hendrich J, Wratten J, Tran Van Minh A (2006). The calcium channel alpha2delta-2 subunit partitions with CaV2.1 into lipid rafts in cerebellum: implications for localization and function.. J Neurosci.

[72] van Oosterhout F, Michel S, Deboer T, Houben T, van de Ven RC (2008). Enhanced circadian phase resetting in R192Q Cav2.1 calcium channel migraine mice.. Ann Neurol.

[73] van den Maagdenberg AM, Pizzorusso T, Kaja S, Terpolilli N, Shapovalova M (2010). High cortical spreading depression susceptibility and migraine-associated symptoms in Ca(v)2.1 S218L mice.. Ann Neurol.

[74] Schurks M, Rist PM, Bigal ME, Buring JE, Lipton RB (2009). Migraine and cardiovascular disease: systematic review and meta-analysis.. BMJ.

[75] Bond DS, Roth J, Nash JM, Wing RR (2010). Migraine and obesity: epidemiology, possible mechanisms and the potential role of weight loss treatment..

[76] Garaulet M, Madrid JA (2010). Chronobiological aspects of nutrition, metabolic syndrome and obesity..

[77] Chen SN, Cilingiroglu M, Todd J, Lombardi R, Willerson JT (2009). Candidate genetic analysis of plasma high-density lipoprotein-cholesterol and severity of coronary atherosclerosis.. BMC Med Genet.

[78] Lara-Castro C, Hunter GR, Lovejoy JC, Gower BA, Fernandez JR (2005). Apolipoprotein A-II polymorphism and visceral adiposity in African-American and white women.. Obes Res.

[79] Flint J, Mackay TF (2009). Genetic architecture of quantitative traits in mice, flies, and humans.. Genome Res.

[80] Lewontin RC (1974). The Genetic Basis of Evolutionary Change..

[81] Gluckman PD, Hanson MA, Bateson P, Beedle AS, Law CM (2009). Towards a new developmental synthesis: adaptive developmental plasticity and human disease.. Lancet.

[82] Kramer MG, Vaugn TT, Pletscher S, King-Ellison K, Adams E (1998). Genetic variation in body weight gain and composition in the intercross of Large (LG/J) and Small (SM/J) inbred strains of mice.. Genetics and Molecular Biology.

[83] Festing M, Lyon MF, Raston S, Brown SDM (1996). Origins and characteristics of inbred strains of mice.. Variants and Strains of the Laboratory Mouse.

[84] Hrbek T, de Brito RA, Wang B, Pletscher LS, Cheverud JM (2006). Genetic characterization of a new set of recombinant inbred lines (LGXSM) formed from the inter-cross of SM/J and LG/J inbred mouse strains.. Mamm Genome.

[85] Templeton AR (2006). Population Genetics and Microevolutionary Theory..

[86] Ehrich TH, Hrbek T, Kenney-Hunt JP, Pletscher LS, Wang B (2005). Fine-mapping gene-by-diet interactions on chromosome 13 in a LG/J x SM/J murine model of obesity.. Diabetes.

[87] Broman KW, Saunak S (2009). A Guide to QTL Mapping with R/qtl..

[88] Li J, Jiang T (2005). Computing the minimum recombinant haplotype configuration from incomplete genotype data on a pedigree by integer linear programming.. J Comput Biol.

[89] Wolf JB, Hager R, Cheverud JM (2008). Genomic imprinting effects on complex traits: a phenotype-based perspective.. Epigenetics.

[90] Haley CS, Knott SA (1992). A simple regression method for mapping quantitative trait loci in line crosses using flanking markers.. Heredity.

[91] Cheverud JM, Lawson HA, Fawcett GL, Wang B, Pletscher LS (2011). Diet-Dependent Genetic and Genomic Imprinting Effects on Obesity in Mice.. Obesity (Silver Spring).

[92] Li J, Ji L (2005). Adjusting multiple testing in multilocus analyses using the eigenvalues of a correlation matrix.. Heredity.

[93] Lander ES, Botstein D (1989). Mapping mendelian factors underlying quantitative traits using RFLP linkage maps.. Genetics.

[94] Wade MJ (1990). Genotype-environment interaction of flour beetles, *Tribolium castaneum*.. Evolution.

[95] Du P, Kibbe WA, Lin SM (2008). lumi: a pipeline for processing Illumina microarray.. Bioinformatics.

[96] Lin SM, Du P, Huber W, Kibbe WA (2008). Model-based variance-stabilizing transformation for Illumina microarray data.. Nucleic Acids Res.

[97] Storey JD (2002). A direct approach to false discovery rates.. Journal of the Royal Statistical Society, Series B.

[98] Mardis ER, Ding L, Dooling DJ, Larson DE, McLellan MD (2009). Recurring mutations found by sequencing an acute myeloid leukemia genome.. N Engl J Med.

[99] Ding L, Ellis MJ, Li S, Larson DE, Chen K (2010). Genome remodelling in a basal-like breast cancer metastasis and xenograft.. Nature.

[100] Li H, Ruan J, Durbin R (2008). Mapping short DNA sequencing reads and calling variants using mapping quality scores.. Genome Res.

[101] Li H, Handsaker B, Wysoker A, Fennell T, Ruan J (2009). The Sequence Alignment/Map format and SAMtools.. Bioinformatics.

[102] Pruitt KD, Tatusova T, Maglott DR (2005). NCBI Reference Sequence (RefSeq): a curated non-redundant sequence database of genomes, transcripts and proteins.. Nucleic Acids Res.

[103] Rhead B, Karolchik D, Kuhn RM, Hinrichs AS, Zweig AS (2010). The UCSC Genome Browser database: update 2010.. Nucleic Acids Res.

[104] Sherry ST, Ward MH, Kholodov M, Baker J, Phan L (2001). dbSNP: the NCBI database of genetic variation.. Nucleic Acids Res.

