# Supplementary figures and images for: Genetic Effects at Pleiotropic Loci Are Context-Dependent with Consequences for the Maintenance of Genetic Variation in Populations
Source: PLoS Genet. 2011 Sep 8;7(9):e1002256. doi: 10.1371/journal.pgen.1002256 (PMC3169520; doi:10.1371/journal.pgen.1002256)

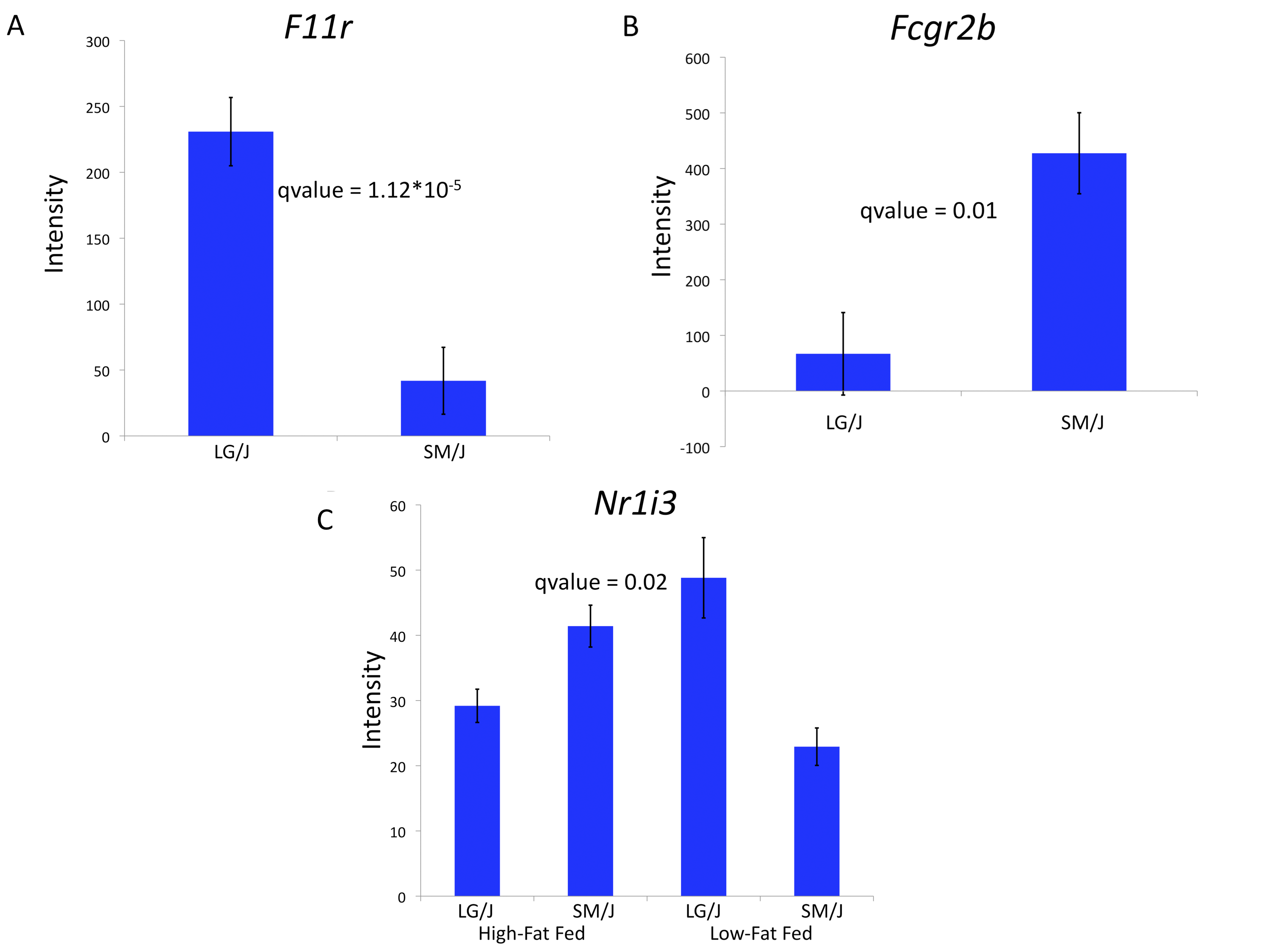

Supplement: Figure S1 — Expression differences in white fat between LG/J and SM/J for positional candidate genes in DMetS1b. Three of 9 genes in DMetS1b that are significantly differentially expressed between LG/J and SM/J in white fat are associated with MetS components. F11r is involved in lesion formation in atherosclerosis-prone mice [49] and shows higher levels of expression in LG/J. Fcgrb2 influences atherosclerosis in apoE(-/-) male mice [50] and shows higher expression levels in SM/J. Nr1i3 is involved in lipid homeostasis [51] and shows differential expression in a strain-by-diet interaction, where individuals fed a low-fat diet have higher expression levels in the LG/J strain and individuals fed a high-fat diet have higher expression levels in the SM/J strain. (TIF) [file pgen.1002256.s001.tif]

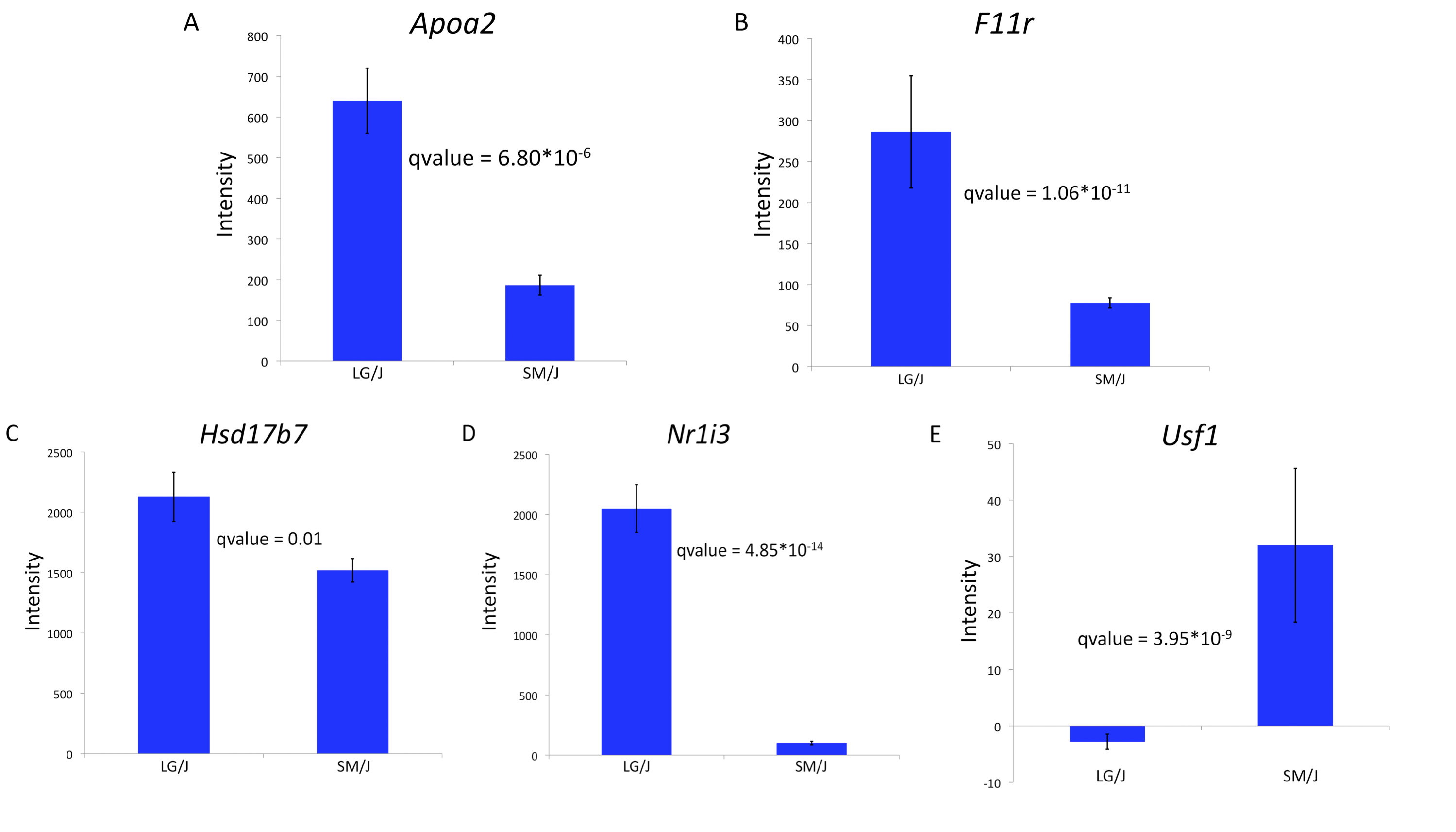

Supplement: Figure S2 — Expression differences in liver between LG/J and SM/J for positional candidate genes in DMetS1b. Five of 10 genes that are significantly differentially expressed between LG/J and SM/J in liver are associated with MetS components. F11r is also differentially expressed in white fat, and in both tissues the LG/J strain shows the highest levels of expression. Nr1i3 is differentially expressed in a strain-by-dietary context in white fat, and shows significantly higher expression levels in LG/J in liver. Apoa2 affects cholesterol levels [47] and shows higher expression in LG/J. Hsd17b7 is involved in fetal cholesterol synthesis [52] and shows higher expression levels in LG/J. Usf1 is associated with blood serum lipid levels and MetS [53], and has higher levels of expression in SM/J. (TIF) [file pgen.1002256.s002.tif]
