# Supplementary material for: Genetic Effects at Pleiotropic Loci Are Context-Dependent with Consequences for the Maintenance of Genetic Variation in Populations
Source: PLoS Genet. 2011 Sep 8;7(9):e1002256. doi: 10.1371/journal.pgen.1002256 (PMC3169520; doi:10.1371/journal.pgen.1002256)
Supplement: Table S1 — Pleiotropic QTL affecting MetS components, their interactions and MetS positional candidate genes. (DOC) [file pgen.1002256.s003.doc]

| ***DMetS1a*** | | | | | | | | | | | |
| --- | --- | --- | --- | --- | --- | --- | --- | --- | --- | --- | --- |
| LOD^a^ | | POS^b^ | | Prox | | Dist | | Total Genes | | MetS Candidates^c^ | |
| **4.43** | | 77.7 | | 76.4 | | 78.2 | | 2 | |  | |
| Traits^d^ | Cohort^e^ | | a^f^ | | d | i | Pr(a) | | Pr(d) | | Pr(i) |
| G | Full | | -0.13350 | | -0.00690 | 0.11620 | 0.0521 | | 0.935 | | 0.1173 |
|  | L | | 0.03693 | | 0.08784 | -0.02456 | 0.5269 | | 0.2378 | | 0.6919 |
|  | H | | -0.28510 | | -0.08749 | 0.23530 | 0.0179 | | 0.5678 | | 0.0655 |
|  | F | | -0.23500 | | -0.09915 | 0.19940 | 0.0541 | | 0.5079 | | 0.1218 |
|  | M | | -0.00129 | | 0.09388 | 0.00244 | 0.9825 | | 0.2171 | | 0.9694 |
|  | LF | | 0.04005 | | 0.05434 | -0.06678 | 0.6763 | | 0.6547 | | 0.5035 |
|  | HF | | -0.51700 | | -0.26770 | 0.47110 | 0.0166 | | 0.3323 | | 0.0392 |
|  | LM | | 0.03972 | | 0.13070 | 0.01516 | 0.5394 | | 0.1209 | | 0.8264 |
|  | HM | | 0.00109 | | 0.09901 | -0.00738 | 0.9907 | | 0.4368 | | 0.9403 |
| I | Full | | -0.18290 | | 0.01463 | 0.15700 | 0.0077 | | 0.8673 | | 0.0322 |
|  | L | | 0.02618 | | 0.07922 | -0.01036 | 0.7173 | | 0.4121 | | 0.8906 |
|  | H | | -0.31650 | | -0.03396 | 0.28600 | 0.0044 | | 0.8135 | | 0.0133 |
|  | F | | -0.19910 | | -0.14440 | 0.24830 | 0.0429 | | 0.2637 | | 0.0149 |
|  | M | | -0.13350 | | 0.18590 | 0.03369 | 0.1348 | | 0.1126 | | 0.7231 |
|  | LF | | 0.09484 | | -0.06876 | -0.04991 | 0.3212 | | 0.596 | | 0.6146 |
|  | HF | | -0.45270 | | -0.18520 | 0.51460 | 0.0053 | | 0.3987 | | 0.0017 |
|  | LM | | -0.03391 | | 0.27370 | 0.02725 | 0.745 | | 0.0547 | | 0.7979 |
|  | HM | | -0.19380 | | 0.14610 | 0.00423 | 0.1627 | | 0.4289 | | 0.9768 |
| T | Full | | -0.41390 | | 0.11630 | 0.34010 | 0.0191 | | 0.5952 | | 0.0742 |
|  | L | | 0.04689 | | 0.34390 | -0.10870 | 0.7763 | | 0.1088 | | 0.5313 |
|  | H | | -0.70620 | | -0.02591 | 0.69390 | 0.0181 | | 0.9456 | | 0.0283 |
|  | F | | -0.54160 | | -0.24890 | 0.62700 | 0.0603 | | 0.4914 | | 0.0387 |
|  | M | | -0.16290 | | 0.51890 | -0.01793 | 0.389 | | 0.0325 | | 0.9299 |
|  | LF | | 0.12040 | | 0.08184 | -0.17680 | 0.6275 | | 0.7987 | | 0.4915 |
|  | HF | | -1.13350 | | -0.64670 | 1.40050 | 0.0221 | | 0.3198 | | 0.0071 |
|  | LM | | -0.00761 | | 0.63100 | -0.03840 | 0.9714 | | 0.0253 | | 0.8643 |
|  | HM | | -0.22480 | | 0.51340 | -0.02027 | 0.4553 | | 0.1927 | | 0.9492 |
| bGLC_20 | Full | | 4.05030 | | -4.07560 | 2.11980 | 0.0312 | | 0.1001 | | 0.2783 |
|  | L | | 7.06720 | | 4.58280 | 5.18550 | 0.0053 | | 0.1819 | | 0.0395 |
|  | H | | 1.64530 | | -12.93220 | 0.62540 | 0.5155 | | 0.0003 | | 0.809 |
|  | F | | 6.03050 | | -2.18090 | 2.30380 | 0.0089 | | 0.4789 | | 0.3405 |
|  | M | | 2.60700 | | -6.24060 | 2.67570 | 0.3704 | | 0.1067 | | 0.361 |
|  | LF | | 9.38840 | | 2.00300 | 1.67590 | 0.0034 | | 0.6319 | | 0.6054 |
|  | HF | | 2.65990 | | -5.69110 | 2.66840 | 0.3984 | | 0.2031 | | 0.4164 |
|  | LM | | 3.06920 | | 8.56090 | 9.26860 | 0.4356 | | 0.1199 | | 0.0137 |
|  | HM | | 0.40520 | | -20.56850 | -1.23890 | 0.9162 | | 0.0003 | | 0.7472 |
| ***DMets1b*** | | | | | | | | | | | |
| LOD^a^ | | POS^b^ | | Prox | | Dist | | Total Genes | | MetS Candidates^c^ | |
| **7.97** | | 172.9 | | 171.3 | | 173.5 | | 47 | | ***Apoa2*^L^**^i^, *Fcer1g*, ***Fcgr2b*^W^**^i^, ***F11r*^L^**^i^;**^W^**^i^, ***Hsd17b7*^L^**^i^, *Rgs4, Rgs5,* ***Usf1*^L^**^i^***,*** *Dedd,* ***Nr1i3*^L^**^i^;**^W^**^iii^ | |
| Traits^d^ | Cohort^e^ | | a^f^ | | d | i | Pr(a) | | Pr(d) | | Pr(i) |
| Chol | Full | | 16.8259 | | -0.8794 | 1.3189 | 0.0001 | | 0.7921 | | 0.6002 |
|  | L | | 14.7204 | | -1.7754 | 2.9893 | 0.0001 | | 0.6092 | | 0.2302 |
|  | H | | 17.7215 | | 0.0584 | -2.8976 | 0.0001 | | 0.9917 | | 0.4794 |
|  | F | | 15.2598 | | 1.5947 | -0.2385 | 0.0001 | | 0.7341 | | 0.9432 |
|  | M | | 19.9968 | | -5.7763 | -0.0193 | 0.0001 | | 0.2142 | | 0.9956 |
|  | LF | | 13.9454 | | -3.6316 | 2.4457 | 0.0001 | | 0.4491 | | 0.4702 |
|  | HF | | 17.2579 | | 3.7540 | -4.9087 | 0.0050 | | 0.6372 | | 0.3624 |
|  | LM | | 16.6442 | | -2.1480 | 2.1967 | 0.0001 | | 0.6695 | | 0.5248 |
|  | HM | | 18.9492 | | -6.6298 | -3.8995 | 0.0024 | | 0.3839 | | 0.4902 |
| G | Full | | 0.0481 | | -0.0962 | -0.3070 | 0.5708 | | 0.3205 | | 0.0002 |
|  | L | | 0.0050 | | -0.1007 | -0.0964 | 0.9431 | | 0.2309 | | 0.1563 |
|  | H | | 0.1254 | | -0.1702 | -0.4672 | 0.3978 | | 0.3290 | | 0.0011 |
|  | F | | 0.0100 | | -0.2310 | -0.6238 | 0.9459 | | 0.1698 | | 0.0001 |
|  | M | | 0.0556 | | 0.0327 | 0.0192 | 0.4480 | | 0.7078 | | 0.7817 |
|  | LF | | 0.0295 | | -0.1915 | -0.1773 | 0.7901 | | 0.1497 | | 0.1068 |
|  | HF | | 0.1295 | | -0.4554 | -0.9700 | 0.6297 | | 0.1489 | | 0.0002 |
|  | LM | | -0.0452 | | 0.0141 | -0.0090 | 0.5765 | | 0.8857 | | 0.9056 |
|  | HM | | 0.1021 | | 0.0181 | 0.0270 | 0.3656 | | 0.8978 | | 0.7993 |
| T | Full | | 0.2750 | | -0.2801 | -0.6033 | 0.2044 | | 0.2621 | | 0.0039 |
|  | L | | -0.0187 | | -0.1237 | -0.2005 | 0.9246 | | 0.6083 | | 0.2956 |
|  | H | | 0.5498 | | -0.5594 | -0.9614 | 0.1310 | | 0.1949 | | 0.0065 |
|  | F | | 0.0634 | | -0.5649 | -1.3865 | 0.8543 | | 0.1625 | | 0.0001 |
|  | M | | 0.3851 | | 0.0229 | 0.1838 | 0.0984 | | 0.9340 | | 0.4140 |
|  | LF | | 0.1380 | | -0.3364 | -0.4192 | 0.6273 | | 0.3366 | | 0.1400 |
|  | HF | | 0.2684 | | -1.1318 | -2.2203 | 0.6582 | | 0.1243 | | 0.0002 |
|  | LM | | -0.2652 | | 0.1756 | 0.0404 | 0.3134 | | 0.5908 | | 0.8702 |
|  | HM | | 0.7195 | | -0.2256 | 0.3444 | 0.0435 | | 0.6016 | | 0.3251 |
| FFA | Full | | 0.0629 | | -0.0257 | -0.0394 | 0.0307 | | 0.4589 | | 0.1572 |
|  | L | | 0.0211 | | -0.0607 | 0.0371 | 0.5914 | | 0.2175 | | 0.3305 |
|  | H | | 0.1219 | | -0.0118 | -0.1107 | 0.0014 | | 0.8016 | | 0.0024 |
|  | F | | 0.1311 | | -0.0885 | 0.0208 | 0.0008 | | 0.0651 | | 0.5849 |
|  | M | | 0.0025 | | 0.0377 | -0.0758 | 0.9498 | | 0.4417 | | 0.0410 |
|  | LF | | 0.0972 | | -0.0484 | 0.1156 | 0.0008 | | 0.5008 | | 0.0049 |
|  | HF | | 0.1795 | | -0.1718 | -0.0942 | 0.0002 | | 0.0048 | | 0.0034 |
|  | LM | | -0.0157 | | -0.0910 | -0.0387 | 0.7528 | | 0.0161 | | 0.3992 |
|  | HM | | 0.0529 | | 0.1493 | -0.1218 | 0.0338 | | 0.0328 | | 0.0208 |
| ***DMetS2a*** | | | | | | | | | | | |
| LOD^a^ | | POS^b^ | | Prox | | Dist | | Total Genes | | MetS Candidates^c^ | |
| 3.65 | | 69.5 | | 68.4 | | 71.8 | | 37 | | *Bps5*, ***G6pc2*^L^**^i^, *Rapgef4, Lrp2*, ***Pdk1*^L^**^i^ | |
| Traits^d^ | Cohort^e^ | | a^f^ | | d | i | Pr(a) | | Pr(d) | | Pr(i) |
| INS | Full | | -0.2236 | | -0.1118 | -0.2404 | 0.0552 | | 0.4893 | | 0.0549 |
|  | L | | -0.0718 | | -0.0299 | 0.1446 | 0.5182 | | 0.8521 | | 0.2272 |
|  | H | | -0.4765 | | -0.1386 | -0.6991 | 0.0179 | | 0.6224 | | 0.0012 |
|  | F | | 0.0535 | | -0.2853 | -0.2478 | 0.6773 | | 0.1097 | | 0.0664 |
|  | M | | -0.5055 | | 0.0954 | -0.2441 | 0.0071 | | 0.7221 | | 0.2318 |
|  | LF | | 0.0511 | | -0.0665 | -0.0416 | 0.7133 | | 0.7421 | | 0.7761 |
|  | HF | | -0.0769 | | -0.5251 | -0.4288 | 0.7085 | | 0.0690 | | 0.0460 |
|  | LM | | -0.2286 | | -0.0002 | 0.3554 | 0.1782 | | 0.9994 | | 0.0581 |
|  | HM | | -0.8954 | | 0.4050 | -0.9238 | 0.0083 | | 0.4021 | | 0.0099 |
| G | Full | | 0.0578 | | -0.3165 | -0.0616 | 0.4179 | | 0.0007 | | 0.3913 |
|  | L | | -0.0434 | | -0.1644 | 0.0390 | 0.4699 | | 0.0449 | | 0.5371 |
|  | H | | 0.1317 | | -0.5166 | -0.1664 | 0.3044 | | 0.0022 | | 0.1687 |
|  | F | | 0.1737 | | -0.5925 | -0.1720 | 0.1710 | | 0.0003 | | 0.1753 |
|  | M | | -0.0765 | | -0.0273 | 0.0028 | 0.2053 | | 0.7407 | | 0.9634 |
|  | LF | | -0.0540 | | -0.2711 | -0.0669 | 0.5700 | | 0.0415 | | 0.5163 |
|  | HF | | 0.3312 | | -0.9765 | -0.2401 | 0.1599 | | 0.0012 | | 0.2667 |
|  | LM | | -0.0196 | | -0.0503 | 0.1200 | 0.7736 | | 0.5860 | | 0.0842 |
|  | HM | | -0.1262 | | 0.0105 | -0.1462 | 0.1950 | | 0.9379 | | 0.1143 |
| T | Full | | 0.0365 | | -0.8086 | -0.0806 | 0.8445 | | 0.0013 | | 0.6702 |
|  | L | | -0.2018 | | -0.5619 | 0.1378 | 0.2398 | | 0.0217 | | 0.4436 |
|  | H | | 0.1941 | | -1.1009 | -0.3738 | 0.5450 | | 0.0109 | | 0.2257 |
|  | F | | 0.3287 | | -1.5965 | -0.2550 | 0.2787 | | 0.0001 | | 0.4059 |
|  | M | | -0.3112 | | 0.0196 | -0.0533 | 0.1170 | | 0.9433 | | 0.7906 |
|  | LF | | -0.1422 | | -0.9726 | -0.1899 | 0.5689 | | 0.0074 | | 0.4758 |
|  | HF | | 0.5842 | | -2.3564 | -0.2342 | 0.2820 | | 0.0014 | | 0.6475 |
|  | LM | | -0.2650 | | -0.1650 | 0.4048 | 0.2394 | | 0.6066 | | 0.0808 |
|  | HM | | -0.3830 | | 0.2983 | -0.5687 | 0.2286 | | 0.4966 | | 0.0612 |
| ***DMetS2b*** | | | | | | | | | | | |
| LOD^a^ | | POS^b^ | | Prox | | Dist | | Total Genes | | MetS Candidates^c^ | |
| **4.19** | | 86.3 | | 84.6 | | 87.4 | | 38 | | *Ctnnd1***, *Aplnr*^W^**^i^, ***Serping1*^L^**^i^**^;W^**^i^ | |
| Traits^d^ | Cohort^e^ | | a^f^ | | d | i | Pr(a) | | Pr(d) | | Pr(i) |
| L | Full | | 0.04713 | | -0.05729 | -0.09012 | 0.0793 | | 0.0927 | | 0.0016 |
|  | L | | 0.01332 | | 0.0177 | 0.01985 | 0.518 | | 0.4957 | | 0.348 |
|  | H | | 0.08035 | | -0.1222 | -0.1998 | 0.0824 | | 0.0477 | | <.0001 |
|  | F | | 0.03611 | | -0.06679 | -0.08389 | 0.2894 | | 0.1264 | | 0.0174 |
|  | M | | 0.06206 | | -0.04709 | -0.09836 | 0.1076 | | 0.3585 | | 0.0179 |
|  | LF | | -0.02075 | | 0.03014 | 0.013 | 0.4452 | | 0.4057 | | 0.6347 |
|  | HF | | 0.08117 | | -0.1478 | -0.1784 | 0.1647 | | 0.0603 | | 0.004 |
|  | LM | | 0.05068 | | -0.00627 | 0.00495 | 0.0771 | | 0.8628 | | 0.8648 |
|  | HM | | 0.08119 | | -0.07038 | -0.2041 | 0.2209 | | 0.4486 | | 0.0061 |
| M | Full | | 0.009379 | | -0.02026 | -0.0176 | 0.7073 | | 0.5292 | | 0.5019 |
|  | L | | -0.02527 | | 0.07687 | 0.07004 | 0.344 | | 0.0313 | | 0.0104 |
|  | H | | 0.04225 | | -0.1055 | -0.1099 | 0.281 | | 0.0436 | | 0.0081 |
|  | F | | -0.00464 | | -0.05173 | -0.04676 | 0.8952 | | 0.2658 | | 0.1991 |
|  | M | | 0.02367 | | 0.01245 | 0.01267 | 0.4658 | | 0.7734 | | 0.7104 |
|  | LF | | -0.01815 | | 0.02703 | 0.01631 | 0.5993 | | 0.5649 | | 0.642 |
|  | HF | | -0.00009 | | -0.1226 | -0.09498 | 0.9987 | | 0.128 | | 0.1175 |
|  | LM | | -0.02852 | | 0.1284 | 0.116 | 0.4611 | | 0.0148 | | 0.0032 |
|  | HM | | 0.08494 | | -0.08494 | -0.08699 | 0.0813 | | 0.1965 | | 0.0875 |
| ***DMetS2c*** | | | | | | | | | | | |
| LOD^a^ | | POS^b^ | | Prox | | Dist | | Total Genes | | MetS Candidates^c^ | |
| **5.60** | | 103.3 | | 102.7 | | 106.0 | | 24 | | ***Cat*^L^**^i^;**^W^**^i^, ***Cd59a*^L^**^i^ | |
| Traits^d^ | Cohort^e^ | | a^f^ | | d | i | Pr(a) | | Pr(d) | | Pr(i) |
| INS | Full | | -0.0093 | | -0.3960 | 0.0258 | 0.9389 | | 0.0152 | | 0.8381 |
|  | L | | -0.0306 | | 0.2621 | 0.1986 | 0.7895 | | 0.1065 | | 0.1019 |
|  | H | | 0.0108 | | -1.1024 | -0.1750 | 0.9587 | | 0.0001 | | 0.4110 |
|  | F | | 0.1534 | | -0.2409 | 0.2067 | 0.2523 | | 0.1828 | | 0.1281 |
|  | M | | -0.1652 | | -0.5643 | -0.1784 | 0.3964 | | 0.0385 | | 0.3913 |
|  | LF | | -0.0804 | | 0.3766 | 0.2640 | 0.5557 | | 0.0647 | | 0.0755 |
|  | HF | | 0.3446 | | -0.9391 | 0.1003 | 0.1140 | | 0.0014 | | 0.6261 |
|  | LM | | 0.0207 | | 0.1006 | 0.0926 | 0.9075 | | 0.6892 | | 0.6161 |
|  | HM | | -0.2800 | | -1.2500 | -0.4556 | 0.4103 | | 0.0127 | | 0.2221 |
| G | Full | | -0.0536 | | -0.2277 | -0.0942 | 0.4554 | | 0.0134 | | 0.2146 |
|  | L | | -0.0075 | | 0.0334 | 0.1464 | 0.9015 | | 0.6732 | | 0.0222 |
|  | H | | -0.1204 | | -0.5003 | -0.2951 | 0.3363 | | 0.0027 | | 0.0238 |
|  | F | | -0.0817 | | -0.3550 | -0.1644 | 0.5282 | | 0.0300 | | 0.2140 |
|  | M | | -0.0094 | | -0.0958 | -0.0502 | 0.8749 | | 0.2378 | | 0.4406 |
|  | LF | | -0.0513 | | 0.1872 | 0.1826 | 0.5905 | | 0.1396 | | 0.0730 |
|  | HF | | -0.1905 | | -0.9481 | -0.4123 | 0.4142 | | 0.0017 | | 0.0675 |
|  | LM | | 0.0339 | | -0.1606 | 0.0753 | 0.6162 | | 0.0764 | | 0.2869 |
|  | HM | | -0.0403 | | 0.0184 | -0.1626 | 0.6689 | | 0.8913 | | 0.1085 |
| M | Full | | -0.0355 | | -0.0557 | -0.0501 | 0.1340 | | 0.0673 | | 0.0458 |
|  | L | | -0.0383 | | 0.0191 | 0.0245 | 0.1295 | | 0.5751 | | 0.3631 |
|  | H | | -0.0325 | | -0.1330 | -0.1253 | 0.3901 | | 0.0073 | | 0.0015 |
|  | F | | -0.0360 | | -0.0682 | -0.0788 | 0.2924 | | 0.1250 | | 0.0236 |
|  | M | | -0.0335 | | -0.0531 | -0.0255 | 0.2717 | | 0.1948 | | 0.4433 |
|  | LF | | -0.0113 | | 0.1220 | -0.0181 | 0.7267 | | 0.0067 | | 0.6013 |
|  | HF | | -0.0712 | | -0.2665 | -0.1288 | 0.2135 | | 0.0004 | | 0.0186 |
|  | LM | | -0.0706 | | -0.1017 | 0.0487 | 0.0518 | | 0.0429 | | 0.2048 |
|  | HM | | 0.0010 | | 0.0083 | -0.1006 | 0.9833 | | 0.8967 | | 0.0476 |
| N | Full | | -0.8789 | | -0.6319 | -0.8489 | 0.0292 | | 0.2107 | | 0.0456 |
|  | L | | -0.8633 | | 0.5239 | 0.2985 | 0.0480 | | 0.3603 | | 0.5199 |
|  | H | | -0.8837 | | -1.9550 | -1.8748 | 0.1669 | | 0.0176 | | 0.0046 |
|  | F | | -0.7151 | | -0.9210 | -0.9551 | 0.2485 | | 0.2391 | | 0.1262 |
|  | M | | -1.0272 | | -0.5475 | -0.7994 | 0.0316 | | 0.3883 | | 0.1240 |
|  | LF | | -0.6244 | | 1.5828 | 0.8609 | 0.2722 | | 0.0401 | | 0.1573 |
|  | HF | | -1.1575 | | -3.5502 | -2.3483 | 0.2721 | | 0.0081 | | 0.0179 |
|  | LM | | -1.2047 | | -0.9763 | -0.4917 | 0.0516 | | 0.2419 | | 0.4513 |
|  | HM | | -0.9161 | | -0.0542 | -1.0575 | 0.1879 | | 0.9550 | | 0.1655 |
| ***DMetS4a*** | | | | | | | | | | | |
| LOD^a^ | | POS^b^ | | Prox | | Dist | | Total Genes | | MetS Candidates^c^ | |
| 3.09 | | 151.6 | | 150.5 | | 152.9 | | 82 | | ***Agtrap*^W^**^i^;**^L^**^i^, *H6pd, Mtor, Pgd,* ***Pik3cd*^W^***^i^*, ***Rbp7*^W^**^i^, *Uts2* | |
| Traits^d^ | Cohort^e^ | | a^f^ | | d | i | Pr(a) | | Pr(d) | | Pr(i) |
| AUC_20 | Full | | -243.7800 | | -71.2669 | 1369.3300 | 0.4635 | | 0.8911 | | 0.0003 |
|  | L | | -507.8800 | | -399.5400 | 293.3600 | 0.0923 | | 0.4000 | | 0.3993 |
|  | H | | -20.8812 | | -58.0073 | 2326.9700 | 0.9721 | | 0.9492 | | 0.0003 |
|  | F | | -488.5500 | | -145.4800 | 471.2500 | 0.0359 | | 0.6883 | | 0.0594 |
|  | M | | 12.7718 | | 35.8024 | 2392.1800 | 0.9835 | | 0.9706 | | 0.0009 |
|  | LF | | -238.2800 | | -347.5400 | 134.9900 | 0.4087 | | 0.4646 | | 0.6876 |
|  | HF | | -813.3100 | | -8.9544 | 779.9500 | 0.0308 | | 0.9870 | | 0.0362 |
|  | LM | | -821.6000 | | -617.7300 | 404.2000 | 0.1336 | | 0.4569 | | 0.5144 |
|  | HM | | 671.6900 | | -30.6371 | 4069.6000 | 0.5309 | | 0.9853 | | 0.0010 |
| S | Full | | 0.0044 | | 0.0065 | 0.0039 | 0.0156 | | 0.0045 | | 0.0386 |
|  | L | | 0.0035 | | 0.0029 | 0.0038 | 0.0838 | | 0.2778 | | 0.0866 |
|  | H | | 0.0049 | | 0.0094 | 0.0032 | 0.0838 | | 0.0102 | | 0.2735 |
|  | F | | 0.0038 | | 0.0061 | 0.0017 | 0.1181 | | 0.0519 | | 0.5100 |
|  | M | | 0.0045 | | 0.0056 | 0.0058 | 0.0700 | | 0.0898 | | 0.0298 |
|  | LF | | 0.0039 | | 0.0049 | 0.0018 | 0.1637 | | 0.2051 | | 0.5618 |
|  | HF | | 0.0026 | | 0.0071 | 0.0010 | 0.4970 | | 0.1597 | | 0.7821 |
|  | LM | | 0.0022 | | 0.0000 | 0.0045 | 0.4170 | | 0.9948 | | 0.1227 |
|  | HM | | 0.0061 | | 0.0091 | 0.0061 | 0.1142 | | 0.0845 | | 0.1446 |
| ***DMetS6a*** | | | | | | | | | | | |
| LOD^a^ | | POS^b^ | | Prox | | Dist | | Total Genes | | MetS Candidates^c^ | |
| 2.92 | | 48.3 | | 47.1 | | 49.8 | | 42 | | *Igf2bp3, Npy, Rarres2* | |
| Traits^d^ | Cohort^e^ | | a^f^ | | d | i | Pr(a) | | Pr(d) | | Pr(i) |
| AUC_20 | Full | | -59.3092 | | 756.2400 | -375.2300 | 0.8508 | | 0.0949 | | 0.2714 |
|  | L | | 514.2000 | | -377.0800 | -6.0839 | 0.0643 | | 0.3366 | | 0.9845 |
|  | H | | -632.2700 | | 1765.7500 | -718.4100 | 0.2727 | | 0.0277 | | 0.2119 |
|  | F | | 237.2900 | | -265.0800 | -77.8513 | 0.2795 | | 0.4108 | | 0.7556 |
|  | M | | -338.5700 | | 1723.6700 | -655.6800 | 0.5754 | | 0.0418 | | 0.2874 |
|  | LF | | 328.7300 | | -281.1800 | -156.8400 | 0.2110 | | 0.4958 | | 0.6339 |
|  | HF | | 86.6860 | | -233.1000 | 33.9246 | 0.8047 | | 0.6392 | | 0.9267 |
|  | LM | | 600.3600 | | -601.3100 | 70.6115 | 0.2326 | | 0.3755 | | 0.8928 |
|  | HM | | -1140.4400 | | 3487.0100 | -1404.9400 | 0.2970 | | 0.0187 | | 0.1692 |
| M | Full | | -0.0113 | | 0.1149 | -0.0648 | 0.6818 | | 0.0040 | | 0.0146 |
|  | L | | 0.0192 | | 0.1332 | -0.0255 | 0.5078 | | 0.0035 | | 0.3842 |
|  | H | | -0.0214 | | 0.0951 | -0.0874 | 0.6274 | | 0.1403 | | 0.0372 |
|  | F | | -0.0409 | | 0.1263 | -0.0941 | 0.2868 | | 0.0323 | | 0.0175 |
|  | M | | 0.0132 | | 0.1038 | -0.0335 | 0.7228 | | 0.0528 | | 0.3227 |
|  | LF | | -0.0019 | | 0.1299 | -0.0912 | 0.9593 | | 0.0327 | | 0.0230 |
|  | HF | | -0.0458 | | 0.1123 | -0.0630 | 0.4747 | | 0.2626 | | 0.3255 |
|  | LM | | 0.0453 | | 0.1224 | 0.0322 | 0.2989 | | 0.0691 | | 0.4329 |
|  | HM | | 0.0002 | | 0.1052 | -0.1128 | 0.9971 | | 0.1908 | | 0.0268 |
| ***DMetS6b*** | | | | | | | | | | | |
| LOD^a^ | | POS^b^ | | Prox | | Dist | | Total Genes | | MetS Candidates^c^ | |
| **5.30** | | 90.7 | | 88.0 | | 91.8 | | 53 | | *Klf15, Mgll, Tpra1* | |
| Traits^d^ | Cohort^e^ | | a^f^ | | d | i | Pr(a) | | Pr(d) | | Pr(i) |
| AUC_20 | Full | | 974.8100 | | -1099.0300 | -842.3500 | 0.0124 | | 0.0307 | | 0.0132 |
|  | L | | -95.1250 | | 169.7000 | 107.2300 | 0.8036 | | 0.7184 | | 0.7255 |
|  | H | | 1717.5600 | | -2069.5800 | -1922.3000 | 0.0072 | | 0.0181 | | 0.0020 |
|  | F | | -179.8300 | | 325.7500 | 198.7000 | 0.5415 | | 0.3953 | | 0.4296 |
|  | M | | 1956.9100 | | -2243.9200 | -1781.2900 | 0.0047 | | 0.0131 | | 0.0036 |
|  | LF | | -465.7900 | | 108.4200 | 290.1300 | 0.2376 | | 0.8237 | | 0.3172 |
|  | HF | | -7.4711 | | 715.8700 | 19.4091 | 0.9860 | | 0.2213 | | 0.9626 |
|  | LM | | 95.1570 | | 475.8700 | -172.7500 | 0.8817 | | 0.5431 | | 0.7455 |
|  | HM | | 3079.5500 | | -4068.9500 | -3361.2400 | 0.0059 | | 0.0078 | | 0.0019 |
| G | Full | | 0.0563 | | 0.3387 | 0.1438 | 0.5649 | | 0.0090 | | 0.1175 |
|  | L | | 0.1455 | | -0.0014 | -0.0174 | 0.0794 | | 0.9902 | | 0.8176 |
|  | H | | 0.1062 | | 0.6949 | 0.2241 | 0.5091 | | 0.0023 | | 0.1525 |
|  | F | | 0.0426 | | 0.6517 | 0.2670 | 0.8124 | | 0.0070 | | 0.1057 |
|  | M | | 0.0713 | | 0.0903 | -0.0051 | 0.3672 | | 0.4036 | | 0.9459 |
|  | LF | | 0.2031 | | 0.1004 | -0.1666 | 0.1349 | | 0.5939 | | 0.1744 |
|  | HF | | 0.0509 | | 1.3171 | 0.6220 | 0.8663 | | 0.0023 | | 0.0326 |
|  | LM | | 0.0846 | | -0.0586 | 0.0794 | 0.3447 | | 0.6277 | | 0.3315 |
|  | HM | | 0.2016 | | 0.2582 | -0.0823 | 0.0812 | | 0.1278 | | 0.4594 |
| N | Full | | 1.0025 | | 1.2719 | 0.5374 | 0.0793 | | 0.0885 | | 0.3103 |
|  | L | | 0.6816 | | 0.4748 | -0.1633 | 0.2722 | | 0.5725 | | 0.7698 |
|  | H | | 1.6891 | | 2.1357 | 0.5896 | 0.0516 | | 0.0723 | | 0.4797 |
|  | F | | 0.5737 | | 3.3612 | 1.1563 | 0.5139 | | 0.0053 | | 0.1484 |
|  | M | | 1.6261 | | -0.1378 | -0.7939 | 0.0119 | | 0.8748 | | 0.1939 |
|  | LF | | 0.5403 | | 0.9092 | -0.7496 | 0.5193 | | 0.4404 | | 0.3100 |
|  | HF | | 1.1354 | | 5.7688 | 2.8580 | 0.4126 | | 0.0039 | | 0.0318 |
|  | LM | | 0.8637 | | 0.3117 | 0.0951 | 0.3059 | | 0.7866 | | 0.9009 |
|  | HM | | 2.7108 | | -1.1081 | -2.1613 | 0.0026 | | 0.3811 | | 0.0142 |
| L | Full | | 0.0523 | | 0.0373 | 0.0002 | 0.1444 | | 0.4033 | | 0.9959 |
|  | L | | 0.0000 | | -0.0244 | -0.0070 | 0.9997 | | 0.4663 | | 0.7764 |
|  | H | | 0.1264 | | 0.0687 | -0.0202 | 0.0399 | | 0.3875 | | 0.7341 |
|  | F | | 0.0844 | | 0.0215 | 0.0793 | 0.0756 | | 0.7230 | | 0.0650 |
|  | M | | 0.0594 | | 0.0166 | -0.0958 | 0.2076 | | 0.7893 | | 0.0383 |
|  | LF | | 0.0252 | | -0.0522 | -0.0316 | 0.4913 | | 0.2762 | | 0.3346 |
|  | HF | | 0.1542 | | 0.0645 | 0.1710 | 0.0504 | | 0.5432 | | 0.0259 |
|  | LM | | -0.0165 | | -0.0008 | 0.0014 | 0.6233 | | 0.9855 | | 0.9647 |
|  | HM | | 0.1486 | | 0.0211 | -0.2019 | 0.0725 | | 0.8509 | | 0.0149 |
| ***DMetS6c*** | | | | | | | | | | | |
| LOD^a^ | | POS^b^ | | Prox | | Dist | | Total Genes | | MetS Candidates^c^ | |
| **6.26** | | 107.8 | | 105.1 | | 111.3 | | 12 | | *Edem, Grm7, Oxtr* | |
| Traits^d^ | Cohort^e^ | | a^f^ | | d | i | Pr(a) | | Pr(d) | | Pr(i) |
| L | Full | | -0.0979 | | -0.0443 | 0.0376 | 0.0002 | | 0.1761 | | 0.1587 |
|  | L | | 0.0016 | | 0.0046 | -0.0182 | 0.9365 | | 0.8564 | | 0.3592 |
|  | H | | -0.1844 | | -0.1000 | 0.0924 | <.0001 | | 0.0911 | | 0.0522 |
|  | F | | -0.0593 | | -0.0487 | 0.0278 | 0.0853 | | 0.2519 | | 0.4080 |
|  | M | | -0.1051 | | -0.0249 | 0.0365 | 0.0049 | | 0.6177 | | 0.3469 |
|  | LF | | 0.0152 | | 0.0174 | -0.0412 | 0.5877 | | 0.6300 | | 0.1270 |
|  | HF | | -0.1303 | | -0.1374 | 0.0980 | 0.0310 | | 0.0723 | | 0.0904 |
|  | LM | | -0.0055 | | -0.0051 | 0.0008 | 0.8382 | | 0.8849 | | 0.9761 |
|  | HM | | -0.1889 | | -0.0428 | 0.0661 | 0.0038 | | 0.6385 | | 0.3519 |
| AUC_20 | Full | | -949.0900 | | -110.6700 | -162.0000 | 0.0041 | | 0.8089 | | 0.6195 |
|  | L | | -26.6614 | | 387.7300 | -684.6300 | 0.9256 | | 0.3341 | | 0.0219 |
|  | H | | -1960.3600 | | -497.1600 | 362.0900 | 0.0014 | | 0.5415 | | 0.5212 |
|  | F | | -346.2800 | | 299.3200 | -8.7857 | 0.1507 | | 0.3655 | | 0.9697 |
|  | M | | -1503.5200 | | -574.9400 | -229.0000 | 0.0123 | | 0.4979 | | 0.7096 |
|  | LF | | -137.4600 | | 375.1500 | -408.9000 | 0.6229 | | 0.3647 | | 0.1797 |
|  | HF | | -537.0600 | | 246.8800 | 332.0700 | 0.1747 | | 0.6313 | | 0.3289 |
|  | LM | | 270.3900 | | 346.1300 | -855.6500 | 0.5865 | | 0.6162 | | 0.0969 |
|  | HM | | -3077.1300 | | -1468.3000 | 613.9500 | 0.0039 | | 0.3195 | | 0.5646 |
| GLC | Full | | 1.8444 | | -12.6564 | 1.3082 | 0.5553 | | 0.0015 | | 0.6825 |
|  | L | | 8.0831 | | -13.6627 | -2.3499 | 0.0465 | | 0.0143 | | 0.5990 |
|  | H | | -5.5960 | | -10.6732 | 3.0887 | 0.1964 | | 0.0587 | | 0.4637 |
|  | F | | 6.2041 | | -19.1249 | 6.7511 | 0.1720 | | 0.0012 | | 0.1314 |
|  | M | | -0.2076 | | -5.8655 | -5.0780 | 0.9583 | | 0.2646 | | 0.2333 |
|  | LF | | 19.8690 | | -18.7202 | 6.0991 | 0.0005 | | 0.0208 | | 0.3110 |
|  | HF | | -9.6230 | | -11.5649 | 2.0686 | 0.1366 | | 0.1610 | | 0.7283 |
|  | LM | | 0.3550 | | -5.9272 | -11.2914 | 0.9441 | | 0.4107 | | 0.0545 |
|  | HM | | -2.3295 | | -8.6131 | 3.7186 | 0.6805 | | 0.2616 | | 0.5215 |
| INS | Full | | -0.2627 | | -0.1382 | 0.0085 | 0.0332 | | 0.3994 | | 0.9468 |
|  | L | | -0.0048 | | 0.3101 | -0.0818 | 0.9652 | | 0.0559 | | 0.5091 |
|  | H | | -0.6139 | | -0.5528 | 0.0801 | 0.0055 | | 0.0540 | | 0.7049 |
|  | F | | 0.1046 | | 0.1798 | 0.0523 | 0.4455 | | 0.3275 | | 0.7022 |
|  | M | | -0.5994 | | -0.4277 | -0.0307 | 0.0016 | | 0.1100 | | 0.8808 |
|  | LF | | 0.1159 | | 0.0420 | -0.1431 | 0.4048 | | 0.8384 | | 0.3498 |
|  | HF | | 0.0483 | | 0.3167 | 0.2435 | 0.8314 | | 0.2941 | | 0.2502 |
|  | LM | | -0.1166 | | 0.5346 | -0.0204 | 0.4763 | | 0.0319 | | 0.9153 |
|  | HM | | -1.1899 | | -1.3755 | -0.1028 | 0.0007 | | 0.0042 | | 0.7639 |
| ***DMetS7a*** | | | | | | | | | | | |
| LOD^a^ | | POS^b^ | | Prox | | Dist | | Total Genes | | MetS Candidates^c^ | |
| 3.51 | | 66.9 | | 65.9 | | 72.8 | | 19 | | *Atp10a* | |
| Traits^d^ | Cohort^e^ | | a^f^ | | d | i | Pr(a) | | Pr(d) | | Pr(i) |
| bGLC_10 | Full | | 6.5818 | | -3.0489 | -3.4665 | 0.0025 | | 0.2685 | | 0.1181 |
|  | L | | 7.9519 | | -3.8061 | -6.0757 | 0.0056 | | 0.3078 | | 0.0455 |
|  | H | | 6.1614 | | -1.4325 | -1.3896 | 0.0319 | | 0.7140 | | 0.6435 |
|  | F | | 7.4292 | | 1.0514 | 1.8134 | 0.0026 | | 0.7581 | | 0.5130 |
|  | M | | 6.1891 | | -4.0216 | -8.5067 | 0.0635 | | 0.3473 | | 0.0080 |
|  | LF | | 11.2299 | | 1.2870 | -3.5021 | 0.0017 | | 0.7918 | | 0.3582 |
|  | HF | | 6.3621 | | 2.3526 | 5.6847 | 0.0321 | | 0.5994 | | 0.1238 |
|  | LM | | 4.1709 | | -5.0616 | -7.8624 | 0.3130 | | 0.3772 | | 0.0708 |
|  | HM | | 7.0937 | | -4.0692 | -7.7620 | 0.1294 | | 0.5103 | | 0.0787 |
| INS | Full | | 0.3366 | | -0.0214 | 0.4893 | 0.0071 | | 0.9018 | | 0.0002 |
|  | L | | 0.3812 | | 0.0736 | 0.1649 | 0.0010 | | 0.6682 | | 0.1888 |
|  | H | | 0.2843 | | -0.1384 | 0.8210 | 0.1810 | | 0.6437 | | 0.0002 |
|  | F | | 0.2121 | | -0.0667 | 0.3794 | 0.1112 | | 0.7295 | | 0.0095 |
|  | M | | 0.4670 | | 0.0062 | 0.5918 | 0.0207 | | 0.9827 | | 0.0043 |
|  | LF | | 0.2461 | | -0.0046 | 0.0227 | 0.0908 | | 0.9834 | | 0.8854 |
|  | HF | | 0.1586 | | -0.1971 | 0.7305 | 0.4383 | | 0.5227 | | 0.0018 |
|  | LM | | 0.4951 | | 0.1426 | 0.2760 | 0.0049 | | 0.5830 | | 0.1478 |
|  | HM | | 0.4726 | | -0.1405 | 0.9167 | 0.1957 | | 0.7858 | | 0.0100 |
| L | Full | | 0.0359 | | 0.0141 | 0.0599 | 0.2467 | | 0.7303 | | 0.0573 |
|  | L | | 0.0004 | | -0.0006 | 0.0259 | 0.9847 | | 0.9851 | | 0.2754 |
|  | H | | 0.0658 | | 0.0293 | 0.0981 | 0.2278 | | 0.6898 | | 0.0750 |
|  | F | | -0.0467 | | -0.0645 | 0.1002 | 0.2181 | | 0.2141 | | 0.0110 |
|  | M | | 0.1065 | | 0.1072 | 0.0317 | 0.0151 | | 0.0774 | | 0.4760 |
|  | LF | | -0.0441 | | 0.0093 | 0.0318 | 0.1501 | | 0.8249 | | 0.2864 |
|  | HF | | -0.0631 | | -0.1289 | 0.1834 | 0.3197 | | 0.1653 | | 0.0104 |
|  | LM | | 0.0320 | | -0.0062 | 0.0386 | 0.2787 | | 0.8881 | | 0.2455 |
|  | HM | | 0.2040 | | 0.2203 | 0.0227 | 0.0120 | | 0.0423 | | 0.7638 |
| M | Full | | 0.0687 | | 0.0144 | 0.0430 | 0.0151 | | 0.6899 | | 0.1303 |
|  | L | | 0.0444 | | 0.0604 | 0.0432 | 0.1224 | | 0.1324 | | 0.1480 |
|  | H | | 0.0914 | | -0.0398 | 0.0490 | 0.0401 | | 0.4916 | | 0.2724 |
|  | F | | -0.0109 | | -0.0408 | 0.0845 | 0.7769 | | 0.4320 | | 0.0337 |
|  | M | | 0.1450 | | 0.0882 | 0.0095 | <.0001 | | 0.0654 | | 0.7919 |
|  | LF | | -0.0066 | | 0.0232 | 0.0338 | 0.8657 | | 0.6575 | | 0.3722 |
|  | HF | | -0.0162 | | -0.1052 | 0.1435 | 0.7889 | | 0.2270 | | 0.0339 |
|  | LM | | 0.0882 | | 0.1085 | 0.0612 | 0.0267 | | 0.0684 | | 0.1705 |
|  | HM | | 0.2007 | | 0.0755 | -0.0151 | 0.0004 | | 0.2936 | | 0.7717 |
| ***DMetS7b*** | | | | | | | | | | | |
| LOD^a^ | | POS^b^ | | Prox | | Dist | | Total Genes | | MetS Candidates^c^ | |
| **4.25** | | 80.0 | | 78.9 | | 80.1 | | 3 | |  | |
| Traits^d^ | Cohort^e^ | | a^f^ | | d | i | Pr(a) | | Pr(d) | | Pr(i) |
| AUC_20 | Full | | -153.9600 | | 1209.5200 | -179.2300 | 0.6517 | | 0.0115 | | 0.5781 |
|  | L | | 422.9000 | | -24.8230 | 351.2600 | 0.1751 | | 0.9525 | | 0.2361 |
|  | H | | -660.1800 | | 2433.1900 | -680.5100 | 0.2673 | | 0.0043 | | 0.2229 |
|  | F | | 9.3424 | | -156.7800 | 322.2900 | 0.9687 | | 0.6448 | | 0.1812 |
|  | M | | -228.9000 | | 2595.9200 | -675.5200 | 0.7263 | | 0.0038 | | 0.2409 |
|  | LF | | 56.3878 | | 67.9910 | 335.0400 | 0.8575 | | 0.8758 | | 0.2443 |
|  | HF | | -38.5393 | | -383.7000 | 261.3500 | 0.9119 | | 0.4654 | | 0.5079 |
|  | LM | | 808.0300 | | -45.1740 | 232.1500 | 0.1329 | | 0.9503 | | 0.6668 |
|  | HM | | -1153.0100 | | 4778.8600 | -1246.7600 | 0.3349 | | 0.0026 | | 0.1747 |
| G | Full | | 0.2019 | | -0.1799 | 0.0887 | 0.0085 | | 0.0676 | | 0.2662 |
|  | L | | 0.1093 | | 0.0892 | 0.0727 | 0.0964 | | 0.3059 | | 0.2802 |
|  | H | | 0.3520 | | -0.4346 | 0.0800 | 0.0064 | | 0.0125 | | 0.5653 |
|  | F | | 0.3352 | | -0.3814 | 0.1480 | 0.0136 | | 0.0280 | | 0.2905 |
|  | M | | 0.0852 | | 0.0038 | 0.0308 | 0.1922 | | 0.9659 | | 0.6571 |
|  | LF | | 0.1724 | | 0.1127 | 0.0807 | 0.1082 | | 0.4261 | | 0.4550 |
|  | HF | | 0.6070 | | -0.8698 | 0.2254 | 0.0083 | | 0.0050 | | 0.3685 |
|  | LM | | 0.0526 | | 0.0623 | 0.0717 | 0.4690 | | 0.5317 | | 0.3442 |
|  | HM | | 0.1066 | | -0.0255 | -0.0721 | 0.2887 | | 0.8607 | | 0.5102 |
| R | Full | | 0.1063 | | -0.0186 | 0.0294 | 0.0054 | | 0.7083 | | 0.4611 |
|  | L | | 0.0358 | | 0.0590 | 0.0181 | 0.2855 | | 0.2009 | | 0.6001 |
|  | H | | 0.1946 | | -0.0950 | 0.0174 | 0.0023 | | 0.2669 | | 0.7995 |
|  | F | | 0.1592 | | -0.1226 | 0.0799 | 0.0068 | | 0.1109 | | 0.1923 |
|  | M | | 0.0655 | | 0.0777 | -0.0357 | 0.1434 | | 0.2048 | | 0.4518 |
|  | LF | | 0.0820 | | 0.0587 | 0.0442 | 0.0972 | | 0.3819 | | 0.3781 |
|  | HF | | 0.2631 | | -0.3019 | 0.1197 | 0.0078 | | 0.0251 | | 0.2724 |
|  | LM | | -0.0091 | | 0.0536 | -0.0109 | 0.8395 | | 0.3945 | | 0.8153 |
|  | HM | | 0.1389 | | 0.0958 | -0.0981 | 0.0580 | | 0.3514 | | 0.2129 |
| T | Full | | 0.5707 | | -0.1865 | 0.2684 | 0.0036 | | 0.4583 | | 0.1889 |
|  | L | | 0.2755 | | 0.3521 | 0.2282 | 0.1314 | | 0.1550 | | 0.2248 |
|  | H | | 1.0172 | | -0.6417 | 0.2106 | 0.0015 | | 0.1334 | | 0.5400 |
|  | F | | 0.7518 | | -0.7405 | 0.3847 | 0.0164 | | 0.0723 | | 0.2414 |
|  | M | | 0.4737 | | 0.3693 | 0.1233 | 0.0232 | | 0.1889 | | 0.5753 |
|  | LF | | 0.3801 | | 0.3219 | 0.2914 | 0.1616 | | 0.3798 | | 0.2915 |
|  | HF | | 1.3271 | | -1.6973 | 0.5026 | 0.0107 | | 0.0186 | | 0.3846 |
|  | LM | | 0.2020 | | 0.3765 | 0.1786 | 0.3918 | | 0.2535 | | 0.4687 |
|  | HM | | 0.7656 | | 0.3587 | -0.1091 | 0.0176 | | 0.4226 | | 0.7506 |
| ***DMetS7c*** | | | | | | | | | | | |
| LOD^a^ | | POS^b^ | | Prox | | Dist | | Total Genes | | MetS Candidates^c^ | |
| **4.40** | | 89.0 | | 87.4 | | 93.2 | | 44 | | Stard5 | |
| Traits^d^ | Cohort^e^ | | a^f^ | | d | i | Pr(a) | | Pr(d) | | Pr(i) |
| G | Full | | 0.2735 | | 0.0047 | 0.0002 | 0.0005 | | 0.9593 | | 0.9982 |
|  | L | | 0.1254 | | -0.0073 | 0.0152 | 0.0608 | | 0.9286 | | 0.8065 |
|  | H | | 0.4706 | | -0.0319 | -0.0651 | 0.0004 | | 0.8433 | | 0.6014 |
|  | F | | 0.4868 | | -0.0438 | -0.0537 | 0.0005 | | 0.7901 | | 0.6799 |
|  | M | | 0.0754 | | 0.0106 | 0.0256 | 0.2545 | | 0.8971 | | 0.6809 |
|  | LF | | 0.1423 | | -0.0245 | -0.0062 | 0.1940 | | 0.8549 | | 0.9510 |
|  | HF | | 0.9186 | | -0.1494 | -0.1224 | 0.0001 | | 0.6079 | | 0.5824 |
|  | LM | | 0.1046 | | -0.0145 | 0.0415 | 0.1538 | | 0.8746 | | 0.5419 |
|  | HM | | 0.0301 | | -0.0344 | -0.0565 | 0.7698 | | 0.7963 | | 0.5486 |
| R | Full | | 0.0793 | | 0.0575 | -0.0120 | 0.0457 | | 0.2244 | | 0.7439 |
|  | L | | 0.0437 | | 0.0203 | 0.0081 | 0.2063 | | 0.6389 | | 0.7930 |
|  | H | | 0.1386 | | 0.0711 | -0.0498 | 0.0368 | | 0.3806 | | 0.4222 |
|  | F | | 0.2010 | | 0.0439 | -0.0420 | 0.0010 | | 0.5509 | | 0.4527 |
|  | M | | -0.0304 | | 0.0508 | 0.0021 | 0.5168 | | 0.3740 | | 0.9612 |
|  | LF | | 0.0681 | | 0.0055 | 0.0310 | 0.1838 | | 0.9319 | | 0.5023 |
|  | HF | | 0.3616 | | 0.0430 | -0.1184 | 0.0005 | | 0.7370 | | 0.2134 |
|  | LM | | 0.0190 | | 0.0312 | -0.0181 | 0.6812 | | 0.5915 | | 0.6610 |
|  | HM | | -0.0769 | | 0.0510 | -0.0087 | 0.3203 | | 0.5953 | | 0.9023 |
| T | Full | | 0.6371 | | 0.1819 | -0.0866 | 0.0018 | | 0.4566 | | 0.6489 |
|  | L | | 0.3636 | | 0.2277 | 0.0567 | 0.0568 | | 0.3391 | | 0.7426 |
|  | H | | 1.0067 | | 0.1094 | -0.3522 | 0.0025 | | 0.7896 | | 0.2534 |
|  | F | | 1.1624 | | 0.2282 | -0.1833 | 0.0004 | | 0.5689 | | 0.5428 |
|  | M | | 0.1389 | | 0.0313 | -0.0711 | 0.5242 | | 0.9074 | | 0.7230 |
|  | LF | | 0.4491 | | 0.1302 | 0.2644 | 0.1113 | | 0.7124 | | 0.2995 |
|  | HF | | 2.0154 | | 0.1857 | -0.6771 | 0.0003 | | 0.7876 | | 0.1751 |
|  | LM | | 0.2745 | | 0.2511 | -0.1379 | 0.2610 | | 0.4202 | | 0.5309 |
|  | HM | | -0.0212 | | -0.2333 | -0.1819 | 0.9504 | | 0.5845 | | 0.5554 |
| N | Full | | 1.7069 | | 1.3207 | 0.1128 | 0.0002 | | 0.0149 | | 0.7929 |
|  | L | | 1.5167 | | 1.5436 | 0.2965 | 0.0021 | | 0.0128 | | 0.5119 |
|  | H | | 2.3247 | | 1.0970 | -0.3199 | 0.0009 | | 0.2030 | | 0.6325 |
|  | F | | 2.6598 | | 1.0423 | -0.3295 | <.0001 | | 0.2104 | | 0.5977 |
|  | M | | 1.1757 | | 1.4015 | 0.2310 | 0.0309 | | 0.0401 | | 0.6536 |
|  | LF | | 1.4173 | | 1.3691 | 0.6177 | 0.0289 | | 0.1003 | | 0.2880 |
|  | HF | | 4.2608 | | 0.6740 | -1.3139 | 0.0001 | | 0.6232 | | 0.1916 |
|  | LM | | 1.8057 | | 1.4687 | -0.0101 | 0.0088 | | 0.0995 | | 0.9873 |
|  | HM | | 0.6587 | | 1.1980 | 0.0910 | 0.4076 | | 0.2371 | | 0.9023 |
| Chol | Full | | 5.6211 | | 13.6620 | -2.4109 | 0.0309 | | <.0001 | | 0.3329 |
|  | L | | -0.6204 | | 7.8836 | 1.9991 | 0.8153 | | 0.0210 | | 0.4213 |
|  | H | | 13.0263 | | 18.6206 | -4.5784 | 0.0018 | | 0.0004 | | 0.2498 |
|  | F | | 1.4654 | | 10.0953 | -0.6604 | 0.6797 | | 0.0268 | | 0.8423 |
|  | M | | 11.7507 | | 15.5060 | -2.9607 | 0.0008 | | 0.0005 | | 0.3709 |
|  | LF | | -2.4169 | | 8.5464 | 2.9154 | 0.4975 | | 0.0690 | | 0.3745 |
|  | HF | | 5.7834 | | 10.3898 | -2.1307 | 0.3103 | | 0.1683 | | 0.6905 |
|  | LM | | 4.5071 | | 6.7272 | 2.3413 | 0.2116 | | 0.1604 | | 0.4871 |
|  | HM | | 22.7951 | | 26.0206 | -6.2311 | <.0001 | | 0.0004 | | 0.2337 |
| K | Full | | 0.0129 | | 0.0082 | -0.0003 | <.0001 | | 0.0118 | | 0.9012 |
|  | L | | 0.0114 | | 0.0072 | 0.0021 | 0.0006 | | 0.1075 | | 0.5182 |
|  | H | | 0.0141 | | 0.0095 | -0.0029 | <.0001 | | 0.0367 | | 0.3889 |
|  | F | | 0.0081 | | 0.0029 | -0.0007 | 0.0005 | | 0.3297 | | 0.7563 |
|  | M | | 0.0184 | | 0.0138 | 0.0000 | <.0001 | | 0.0139 | | 0.9966 |
|  | LF | | 0.0062 | | 0.0076 | 0.0029 | 0.0480 | | 0.0704 | | 0.3164 |
|  | HF | | 0.0103 | | -0.0002 | -0.0041 | 0.0006 | | 0.9518 | | 0.1364 |
|  | LM | | 0.0169 | | 0.0070 | 0.0014 | 0.0031 | | 0.3623 | | 0.7946 |
|  | HM | | 0.0192 | | 0.0219 | -0.0024 | 0.0014 | | 0.0063 | | 0.6804 |
| ***DMetS8a*** | | | | | | | | | | | |
| LOD^a^ | | POS^b^ | | Prox | | Dist | | Total Genes | | MetS Candidates^c^ | |
| **4.44** | | 25.5 | | 18.3 | | 31.8 | | 113 | | *Ikbkb, Enpp1*, ***Adrb3*^L^**^i^, ***Agpat6*^W^**^iv^, *Star, Eif4ebp1* | |
| Traits^d^ | Cohort^e^ | | a^f^ | | d | i | Pr(a) | | Pr(d) | | Pr(i) |
| AUC_10 | Full | | -1050.5200 | | -962.4500 | -622.2700 | 0.0010 | | 0.0922 | | 0.0741 |
|  | L | | -375.2700 | | -906.1200 | -284.3200 | 0.2352 | | 0.1328 | | 0.4202 |
|  | H | | -1786.3400 | | -822.5000 | -1102.6300 | 0.0007 | | 0.3829 | | 0.0545 |
|  | F | | -744.2400 | | -845.0800 | -312.6200 | 0.0019 | | 0.0617 | | 0.2317 |
|  | M | | -1328.4000 | | -712.6500 | -940.4400 | 0.0199 | | 0.4958 | | 0.1391 |
|  | LF | | -743.7100 | | -529.2400 | -483.4500 | 0.0103 | | 0.3505 | | 0.1410 |
|  | HF | | -700.6900 | | -995.1300 | -172.4200 | 0.0581 | | 0.1539 | | 0.6664 |
|  | LM | | -50.6339 | | -1194.8200 | -95.7556 | 0.9292 | | 0.2669 | | 0.8772 |
|  | HM | | -2736.3400 | | 320.2800 | -2478.3000 | 0.0027 | | 0.8509 | | 0.0205 |
| G | Full | | -0.3049 | | -0.1094 | -0.0627 | 0.0052 | | 0.5095 | | 0.5297 |
|  | L | | -0.1199 | | -0.0043 | -0.0720 | 0.2020 | | 0.9767 | | 0.3929 |
|  | H | | -0.5348 | | -0.1589 | -0.0518 | 0.0036 | | 0.5852 | | 0.7607 |
|  | F | | -0.6021 | | -0.0611 | -0.0731 | 0.0010 | | 0.8231 | | 0.6697 |
|  | M | | 0.0470 | | 0.0187 | -0.0676 | 0.6301 | | 0.9047 | | 0.4282 |
|  | LF | | -0.2053 | | 0.1691 | 0.1074 | 0.1390 | | 0.4394 | | 0.4264 |
|  | HF | | -1.0544 | | -0.2817 | -0.1767 | 0.0010 | | 0.5679 | | 0.5534 |
|  | LM | | 0.0197 | | -0.0777 | -0.2458 | 0.8639 | | 0.6669 | | 0.0073 |
|  | HM | | 0.0194 | | 0.1177 | 0.0548 | 0.8921 | | 0.6296 | | 0.6783 |
| R | Full | | -0.1874 | | -0.0696 | -0.0893 | 0.0004 | | 0.4185 | | 0.0810 |
|  | L | | -0.1056 | | -0.0740 | -0.0868 | 0.0201 | | 0.3431 | | 0.0518 |
|  | H | | -0.2766 | | -0.0432 | -0.1068 | 0.0019 | | 0.7699 | | 0.2123 |
|  | F | | -0.2763 | | -0.0485 | -0.1146 | 0.0004 | | 0.7003 | | 0.1425 |
|  | M | | -0.0811 | | -0.0330 | -0.0853 | 0.2150 | | 0.7662 | | 0.1597 |
|  | LF | | -0.1070 | | -0.0669 | 0.0413 | 0.0933 | | 0.5392 | | 0.5368 |
|  | HF | | -0.4598 | | -0.0688 | -0.2128 | 0.0007 | | 0.7551 | | 0.1070 |
|  | LM | | -0.0958 | | -0.0704 | -0.2189 | 0.1322 | | 0.5190 | | 0.0002 |
|  | HM | | -0.0788 | | 0.0031 | 0.0070 | 0.4604 | | 0.9866 | | 0.9450 |
| M | Full | | -0.1086 | | -0.0536 | -0.0510 | 0.0023 | | 0.3078 | | 0.1172 |
|  | L | | -0.0554 | | -0.0463 | -0.0927 | 0.1510 | | 0.4293 | | 0.0060 |
|  | H | | -0.1457 | | 0.0003 | 0.0034 | 0.0086 | | 0.9971 | | 0.9478 |
|  | F | | -0.2049 | | -0.0568 | -0.0118 | <.0001 | | 0.4294 | | 0.7906 |
|  | M | | -0.0011 | | 0.0065 | -0.0917 | 0.9813 | | 0.9307 | | 0.0313 |
|  | LF | | -0.0730 | | -0.0261 | -0.0062 | 0.1222 | | 0.7228 | | 0.8905 |
|  | HF | | -0.3139 | | -0.0583 | 0.0164 | <.0001 | | 0.6296 | | 0.8190 |
|  | LM | | -0.0148 | | -0.0270 | -0.1806 | 0.8084 | | 0.7686 | | 0.0001 |
|  | HM | | 0.0017 | | 0.0698 | -0.0072 | 0.9808 | | 0.5383 | | 0.9129 |
| I | Full | | -0.2928 | | -0.1473 | -0.2314 | 0.0041 | | 0.3680 | | 0.0197 |
|  | L | | -0.1808 | | -0.3901 | -0.2138 | 0.0828 | | 0.0278 | | 0.0397 |
|  | H | | -0.4235 | | 0.1141 | -0.2497 | 0.0078 | | 0.6675 | | 0.1082 |
|  | F | | -0.4285 | | -0.0921 | -0.1567 | 0.0019 | | 0.6856 | | 0.2639 |
|  | M | | -0.1538 | | -0.1506 | -0.3094 | 0.2561 | | 0.5105 | | 0.0155 |
|  | LF | | -0.1571 | | -0.4618 | 0.0988 | 0.2303 | | 0.0436 | | 0.4796 |
|  | HF | | -0.6578 | | 0.1972 | -0.3028 | 0.0032 | | 0.6029 | | 0.1769 |
|  | LM | | -0.1879 | | -0.3261 | -0.5279 | 0.2207 | | 0.2218 | | 0.0003 |
|  | HM | | -0.1941 | | -0.0130 | -0.1646 | 0.3473 | | 0.9708 | | 0.4010 |
| T | Full | | -0.9825 | | -0.2719 | -0.3722 | 0.0004 | | 0.5343 | | 0.1505 |
|  | L | | -0.5335 | | -0.4590 | -0.3935 | 0.0421 | | 0.2832 | | 0.1017 |
|  | H | | -1.4937 | | 0.0883 | -0.3486 | 0.0010 | | 0.9047 | | 0.4084 |
|  | F | | -1.7675 | | 0.0256 | -0.2460 | <.0001 | | 0.9694 | | 0.5452 |
|  | M | | -0.1048 | | -0.0962 | -0.5160 | 0.7396 | | 0.8526 | | 0.0639 |
|  | LF | | -0.6877 | | -0.2036 | 0.2470 | 0.0560 | | 0.7304 | | 0.4881 |
|  | HF | | -2.7847 | | 0.1084 | -0.4696 | 0.0001 | | 0.9259 | | 0.4883 |
|  | LM | | -0.2734 | | -0.5058 | -1.0405 | 0.4538 | | 0.4003 | | 0.0006 |
|  | HM | | -0.1653 | | 0.2872 | -0.1589 | 0.7271 | | 0.7219 | | 0.7150 |
| N | Full | | -2.5532 | | -0.8656 | -0.6220 | <.0001 | | 0.3731 | | 0.2845 |
|  | L | | -1.2527 | | -1.1186 | -0.8115 | 0.0696 | | 0.3148 | | 0.1986 |
|  | H | | -3.6826 | | -0.0618 | -0.3580 | 0.0002 | | 0.9685 | | 0.6926 |
|  | F | | -3.7351 | | -0.6789 | -0.1800 | <.0001 | | 0.6254 | | 0.8316 |
|  | M | | -1.0554 | | -0.3175 | -1.0193 | 0.1919 | | 0.8110 | | 0.1501 |
|  | LF | | -1.3593 | | -1.0639 | 0.7958 | 0.1083 | | 0.4504 | | 0.3424 |
|  | HF | | -5.9314 | | -0.6847 | -0.6377 | <.0001 | | 0.7681 | | 0.6400 |
|  | LM | | -0.7140 | | -0.7083 | -2.4295 | 0.5017 | | 0.6858 | | 0.0055 |
|  | HM | | -1.4274 | | 0.2800 | -0.0011 | 0.2053 | | 0.8847 | | 0.9992 |
| ***DMetS8b*** | | | | | | | | | | | |
| LOD^a^ | | POS^b^ | | Prox | | Dist | | Total Genes | | MetS Candidates^c^ | |
| 2.78 | | 86.1 | | 85.3 | | 87.1 | | 36 | | *Ptger1, Ucp1,* ***Cacna1a*^L^**^i^;**^W^**^i^ | |
| Traits^d^ | Cohort^e^ | | a^f^ | | d | i | Pr(a) | | Pr(d) | | Pr(i) |
| M | Full | | 0.0915 | | 0.0074 | -0.0463 | 0.0029 | | 0.8388 | | 0.0836 |
|  | L | | 0.0669 | | 0.0535 | -0.0133 | 0.0386 | | 0.1886 | | 0.6339 |
|  | H | | 0.1252 | | -0.0479 | -0.0657 | 0.0104 | | 0.4229 | | 0.1339 |
|  | F | | 0.0783 | | -0.0310 | -0.0304 | 0.0613 | | 0.5505 | | 0.4167 |
|  | M | | 0.1095 | | 0.0431 | -0.0611 | 0.0080 | | 0.3898 | | 0.0847 |
|  | LF | | 0.0777 | | 0.0026 | -0.0287 | 0.0736 | | 0.9607 | | 0.4168 |
|  | HF | | 0.0867 | | -0.0734 | -0.0262 | 0.1901 | | 0.4059 | | 0.6885 |
|  | LM | | 0.0597 | | 0.1026 | 0.0070 | 0.1936 | | 0.0905 | | 0.8689 |
|  | HM | | 0.1665 | | -0.0181 | -0.1176 | 0.0116 | | 0.8177 | | 0.0281 |
| Chol | Full | | 2.2431 | | 2.9523 | -3.8995 | 0.4445 | | 0.4090 | | 0.1364 |
|  | L | | 0.8346 | | 6.4258 | 1.9919 | 0.7789 | | 0.0870 | | 0.4461 |
|  | H | | 3.7312 | | -0.2593 | -10.8445 | 0.4350 | | 0.9656 | | 0.0134 |
|  | F | | 7.3743 | | 5.5567 | -7.0576 | 0.0517 | | 0.2531 | | 0.0413 |
|  | M | | -3.5465 | | 0.6361 | -0.0698 | 0.3951 | | 0.9018 | | 0.9848 |
|  | LF | | 2.2711 | | 9.4265 | -3.8758 | 0.5791 | | 0.0690 | | 0.2556 |
|  | HF | | 12.1907 | | 2.4837 | -12.2931 | 0.0377 | | 0.7584 | | 0.0368 |
|  | LM | | -0.0817 | | 3.0424 | 8.9392 | 0.9837 | | 0.5675 | | 0.0180 |
|  | HM | | -6.5849 | | 1.2375 | -7.0205 | 0.3545 | | 0.8872 | | 0.2371 |
| ***DMetS10a*** | | | | | | | | | | | |
| LOD^a^ | | POS^b^ | | Prox | | Dist | | Total Genes | | MetS Candidates^c^ | |
| **4.53** | | 97.5 | | 95.3 | | 100.4 | | 36 | |  | |
| Traits^d^ | Cohort^e^ | | a^f^ | | d | i | Pr(a) | | Pr(d) | | Pr(i) |
| S | Full | | 0.0103 | | -0.0039 | 0.0010 | <.0001 | | 0.1190 | | 0.6148 |
|  | L | | 0.0088 | | -0.0091 | 0.0030 | <.0001 | | 0.0013 | | 0.1871 |
|  | H | | 0.0106 | | 0.0012 | -0.0015 | 0.0005 | | 0.7613 | | 0.6152 |
|  | F | | 0.0079 | | -0.0015 | 0.0004 | 0.0017 | | 0.6576 | | 0.8663 |
|  | M | | 0.0117 | | -0.0057 | 0.0022 | <.0001 | | 0.1144 | | 0.4244 |
|  | LF | | 0.0082 | | -0.0038 | 0.0032 | 0.0052 | | 0.3560 | | 0.3324 |
|  | HF | | 0.0075 | | 0.0021 | -0.0034 | 0.0550 | | 0.6899 | | 0.3671 |
|  | LM | | 0.0077 | | -0.0124 | 0.0034 | 0.0124 | | 0.0024 | | 0.2478 |
|  | HM | | 0.0148 | | 0.0001 | 0.0008 | 0.0004 | | 0.9809 | | 0.8450 |
| TG | Full | | -6.5143 | | -2.4149 | -6.1623 | 0.0063 | | 0.4537 | | 0.0183 |
|  | L | | -5.6709 | | 1.6893 | -5.4025 | 0.0429 | | 0.6675 | | 0.0712 |
|  | H | | -7.0935 | | -5.3024 | -7.7621 | 0.0461 | | 0.2707 | | 0.0473 |
|  | F | | -10.1165 | | -0.8656 | -12.0374 | 0.0007 | | 0.8343 | | 0.0003 |
|  | M | | -2.5617 | | -2.4269 | -2.0417 | 0.4599 | | 0.6109 | | 0.5759 |
|  | LF | | -5.3751 | | 2.7173 | -12.7619 | 0.1777 | | 0.6386 | | 0.0047 |
|  | HF | | -15.3450 | | -3.8879 | -10.5530 | 0.0004 | | 0.4993 | | 0.0254 |
|  | LM | | -4.8298 | | 1.9391 | 0.2326 | 0.1857 | | 0.7032 | | 0.9486 |
|  | HM | | 0.5695 | | -4.7783 | -6.2381 | 0.9158 | | 0.5259 | | 0.2947 |
| N | Full | | 1.5937 | | 1.0731 | -0.0138 | 0.0016 | | 0.1466 | | 0.9791 |
|  | L | | 1.1667 | | 1.4712 | -0.6846 | 0.0324 | | 0.0762 | | 0.2238 |
|  | H | | 2.1400 | | 0.8469 | 0.3812 | 0.0069 | | 0.4735 | | 0.6436 |
|  | F | | 1.2608 | | 1.2753 | -0.4861 | 0.0879 | | 0.2474 | | 0.5297 |
|  | M | | 2.1484 | | 0.6571 | 0.1872 | 0.0006 | | 0.4834 | | 0.7665 |
|  | LF | | 0.5986 | | 1.6736 | -0.9607 | 0.3924 | | 0.1262 | | 0.1871 |
|  | HF | | 2.5257 | | 0.8433 | -0.0136 | 0.0412 | | 0.6498 | | 0.9915 |
|  | LM | | 1.6398 | | 1.3174 | -0.6113 | 0.0407 | | 0.2835 | | 0.4435 |
|  | HM | | 2.4292 | | 0.3926 | 0.8223 | 0.0062 | | 0.7720 | | 0.3677 |
| ***DMetS10b*** | | | | | | | | | | | |
| LOD^a^ | | POS^b^ | | Prox | | Dist | | Total Genes | | MetS Candidates^c^ | |
| 2.91 | | 11.5 | | 11.4 | | 11.6 | | 23 | | *Lyz2* | |
| Traits^d^ | Cohort^e^ | | a^f^ | | d | i | Pr(a) | | Pr(d) | | Pr(i) |
| I | Full | | 0.0058 | | 0.2417 | 0.1648 | 0.9386 | | 0.0125 | | 0.0241 |
|  | L | | 0.0290 | | 0.0889 | 0.1329 | 0.7160 | | 0.3962 | | 0.0846 |
|  | H | | -0.0383 | | 0.4050 | 0.2143 | 0.7553 | | 0.0111 | | 0.0685 |
|  | F | | -0.2256 | | 0.5497 | 0.1275 | 0.0404 | | 0.0001 | | 0.2165 |
|  | M | | 0.1761 | | -0.0466 | 0.2823 | 0.0678 | | 0.7113 | | 0.0033 |
|  | LF | | 0.0000 | | 0.3131 | 0.1413 | 1.0000 | | 0.0267 | | 0.1689 |
|  | HF | | -0.4968 | | 0.7910 | 0.1608 | 0.0117 | | 0.0021 | | 0.3533 |
|  | LM | | 0.0900 | | -0.1260 | 0.1909 | 0.4477 | | 0.4191 | | 0.0877 |
|  | HM | | 0.2619 | | 0.0831 | 0.3886 | 0.0680 | | 0.6641 | | 0.0085 |
| T | Full | | -0.0168 | | 0.6351 | 0.3091 | 0.9337 | | 0.0127 | | 0.1101 |
|  | L | | 0.0448 | | 0.3073 | 0.1899 | 0.8122 | | 0.2121 | | 0.2958 |
|  | H | | -0.0681 | | 0.8656 | 0.4856 | 0.8416 | | 0.0481 | | 0.1358 |
|  | F | | -0.6800 | | 1.5022 | 0.2202 | 0.0407 | | 0.0004 | | 0.4744 |
|  | M | | 0.4741 | | -0.1919 | 0.5864 | 0.0248 | | 0.4846 | | 0.0055 |
|  | LF | | -0.1756 | | 1.0229 | 0.1489 | 0.5191 | | 0.0052 | | 0.5769 |
|  | HF | | -1.2071 | | 1.8332 | 0.4477 | 0.0508 | | 0.0202 | | 0.4055 |
|  | LM | | 0.3312 | | -0.4954 | 0.4053 | 0.1855 | | 0.1313 | | 0.0896 |
|  | HM | | 0.6311 | | 0.1730 | 0.7670 | 0.0484 | | 0.6841 | | 0.0202 |
| L | Full | | 0.0501 | | 0.1280 | 0.0167 | 0.1367 | | 0.0046 | | 0.5733 |
|  | L | | 0.0108 | | 0.1130 | -0.0194 | 0.6611 | | 0.0010 | | 0.3911 |
|  | H | | 0.1026 | | 0.1325 | 0.0580 | 0.0972 | | 0.1073 | | 0.2640 |
|  | F | | 0.0404 | | 0.1112 | 0.0054 | 0.3587 | | 0.0591 | | 0.8840 |
|  | M | | 0.0704 | | 0.1487 | 0.0461 | 0.1471 | | 0.0294 | | 0.2954 |
|  | LF | | 0.0213 | | 0.1446 | -0.0320 | 0.5119 | | 0.0021 | | 0.2831 |
|  | HF | | 0.0812 | | 0.0479 | 0.0524 | 0.3392 | | 0.6662 | | 0.4235 |
|  | LM | | 0.0054 | | 0.0806 | -0.0030 | 0.8786 | | 0.1033 | | 0.9254 |
|  | HM | | 0.1400 | | 0.2166 | 0.0885 | 0.1015 | | 0.0734 | | 0.2512 |
| ***DMetS14a*** | | | | | | | | | | | |
| LOD^a^ | | POS^b^ | | Prox | | Dist | | Total Genes | | MetS Candidates^c^ | |
| 3.66 | | 26.1 | | 23.3 | | 28.9 | | 34 | |  | |
| Traits^d^ | Cohort^e^ | | a^f^ | | d | i | Pr(a) | | Pr(d) | | Pr(i) |
| G | Full | | 0.0095 | | 0.0448 | 0.1058 | 0.8954 | | 0.6385 | | 0.1868 |
|  | L | | 0.0238 | | 0.0293 | -0.0538 | 0.7020 | | 0.7264 | | 0.4243 |
|  | H | | -0.0051 | | 0.0392 | 0.3636 | 0.9671 | | 0.8195 | | 0.0111 |
|  | F | | -0.0187 | | 0.2229 | 0.2460 | 0.8852 | | 0.1960 | | 0.0858 |
|  | M | | 0.0290 | | -0.1465 | 0.0024 | 0.6300 | | 0.0756 | | 0.9716 |
|  | LF | | -0.0594 | | 0.1481 | -0.0771 | 0.5707 | | 0.2867 | | 0.4873 |
|  | HF | | -0.0002 | | 0.2394 | 0.7152 | 0.9992 | | 0.4466 | | 0.0060 |
|  | LM | | 0.0917 | | -0.0823 | -0.0387 | 0.1663 | | 0.3707 | | 0.5968 |
|  | HM | | -0.0237 | | -0.2206 | 0.0530 | 0.8008 | | 0.1042 | | 0.6215 |
| N | Full | | 0.3950 | | 1.7110 | 0.9025 | 0.5327 | | 0.1797 | | 0.0977 |
|  | L | | -0.1316 | | 0.3749 | -0.3170 | 0.8499 | | 0.7975 | | 0.5924 |
|  | H | | 0.7327 | | 1.7330 | 2.9075 | 0.4573 | | 0.4040 | | 0.0009 |
|  | F | | 0.1059 | | 3.2046 | 1.5177 | 0.9122 | | 0.1158 | | 0.0690 |
|  | M | | 0.3593 | | -0.5633 | 0.9147 | 0.6293 | | 0.7169 | | 0.1636 |
|  | LF | | -0.6701 | | 2.3250 | 0.4520 | 0.4765 | | 0.2535 | | 0.5660 |
|  | HF | | 0.2110 | | 2.5167 | 3.8067 | 0.8912 | | 0.4686 | | 0.0075 |
|  | LM | | 0.0248 | | -1.7294 | -0.8705 | 0.9789 | | 0.3936 | | 0.2969 |
|  | HM | | 0.9514 | | -0.4038 | 2.9138 | 0.3773 | | 0.8622 | | 0.0024 |
| AUC_10 | Full | | 496.9500 | | -221.7000 | 618.5400 | 0.0539 | | 0.5275 | | 0.0318 |
|  | L | | 340.2200 | | 246.7900 | 397.8000 | 0.1758 | | 0.4962 | | 0.1739 |
|  | H | | 781.9200 | | -866.6800 | 1034.7700 | 0.0679 | | 0.1407 | | 0.0259 |
|  | F | | -25.0897 | | 82.4202 | 307.6700 | 0.8989 | | 0.7698 | | 0.1593 |
|  | M | | 1069.4800 | | -652.1900 | 1093.5700 | 0.0181 | | 0.2958 | | 0.0327 |
|  | LF | | 455.9600 | | 284.0300 | 692.0100 | 0.0516 | | 0.4137 | | 0.0146 |
|  | HF | | -571.6800 | | 73.8556 | 29.6274 | 0.0723 | | 0.8654 | | 0.9254 |
|  | LM | | 277.0300 | | 195.0500 | 186.5600 | 0.5371 | | 0.7617 | | 0.7157 |
|  | HM | | 1987.3900 | | -1521.1400 | 2257.4100 | 0.0073 | | 0.1386 | | 0.0070 |
| ***DMetS15a*** | | | | | | | | | | | |
| LOD^a^ | | POS^b^ | | Prox | | Dist | | Total Genes | | MetS Candidates^c^ | |
| **5.04** | | 67.2 | | 66.8 | | 68.4 | | 6 | | *Wisp1, Ndrgi,* ***St3gal1*^L^**^i^; **^W^**^i^; **^W^**^iii^;**^W^**^iv^ | |
| Traits^d^ | Cohort^e^ | | a^f^ | | d | i | Pr(a) | | Pr(d) | | Pr(i) |
| AUC_10 | Full | | 113.7300 | | -516.3900 | -133.6100 | 0.6792 | | 0.1505 | | 0.6085 |
|  | L | | -510.6500 | | 414.0200 | -265.7300 | 0.0559 | | 0.2658 | | 0.3319 |
|  | H | | 1229.8300 | | -1886.4000 | 29.5469 | 0.0072 | | 0.0014 | | 0.9400 |
|  | F | | 37.9497 | | -522.0400 | 135.4200 | 0.8507 | | 0.0650 | | 0.5034 |
|  | M | | 359.2700 | | -652.3400 | -427.0700 | 0.4790 | | 0.3133 | | 0.3501 |
|  | LF | | -520.4000 | | -495.2200 | -84.1795 | 0.0367 | | 0.1757 | | 0.7655 |
|  | HF | | 710.5900 | | -683.5700 | 328.9000 | 0.0323 | | 0.1121 | | 0.2510 |
|  | LM | | -468.6800 | | 1456.9000 | -551.4900 | 0.3341 | | 0.0266 | | 0.2254 |
|  | HM | | 1784.9400 | | -3139.3200 | -282.1600 | 0.0317 | | 0.0038 | | 0.6909 |
| M | Full | | -0.0338 | | -0.0107 | 0.0367 | 0.1672 | | 0.7379 | | 0.1332 |
|  | L | | 0.0075 | | 0.0199 | -0.0312 | 0.7655 | | 0.5726 | | 0.2457 |
|  | H | | -0.0444 | | -0.0295 | 0.1130 | 0.2600 | | 0.5650 | | 0.0032 |
|  | F | | -0.0256 | | -0.0772 | 0.0851 | 0.4430 | | 0.0916 | | 0.0136 |
|  | M | | -0.0250 | | 0.0718 | -0.0013 | 0.4464 | | 0.0969 | | 0.9683 |
|  | LF | | 0.0015 | | -0.0275 | 0.0056 | 0.9642 | | 0.5508 | | 0.8725 |
|  | HF | | -0.0283 | | -0.1272 | 0.1681 | 0.6078 | | 0.0969 | | 0.0030 |
|  | LM | | 0.0137 | | 0.0882 | -0.0549 | 0.7081 | | 0.0904 | | 0.1578 |
|  | HM | | -0.0234 | | 0.0588 | 0.0505 | 0.6488 | | 0.3699 | | 0.2877 |
| ***DMetS16a*** | | | | | | | | | | | |
| LOD^a^ | | POS^b^ | | Prox | | Dist | | Total Genes | | MetS Candidates^c^ | |
| 3.75 | | 55.3 | | 53.0 | | 59.2 | | 51 | |  | |
| Traits^d^ | Cohort^e^ | | a^f^ | | d | i | Pr(a) | | Pr(d) | | Pr(i) |
| K | Full | | 0.0044 | | -0.0058 | -0.0007 | 0.0719 | | 0.0512 | | 0.7829 |
|  | L | | -0.0029 | | -0.0081 | -0.0043 | 0.3538 | | 0.0475 | | 0.1691 |
|  | H | | 0.0121 | | -0.0046 | 0.0026 | 0.0004 | | 0.2674 | | 0.4321 |
|  | F | | 0.0007 | | -0.0053 | 0.0022 | 0.7386 | | 0.0493 | | 0.2879 |
|  | M | | 0.0077 | | -0.0066 | -0.0029 | 0.0585 | | 0.1995 | | 0.4925 |
|  | LF | | -0.0051 | | -0.0041 | 0.0047 | 0.0831 | | 0.2850 | | 0.0886 |
|  | HF | | 0.0068 | | -0.0061 | -0.0009 | 0.0212 | | 0.1095 | | 0.7398 |
|  | LM | | -0.0009 | | -0.0123 | -0.0134 | 0.8674 | | 0.0845 | | 0.0139 |
|  | HM | | 0.0170 | | -0.0017 | 0.0061 | 0.0028 | | 0.8128 | | 0.2804 |
| INS | Full | | 0.0237 | | 0.2788 | -0.2787 | 0.8505 | | 0.0803 | | 0.0213 |
|  | L | | -0.0303 | | 0.4678 | -0.1352 | 0.7929 | | 0.0031 | | 0.2233 |
|  | H | | 0.1360 | | 0.0813 | -0.4846 | 0.5315 | | 0.7689 | | 0.0196 |
|  | F | | 0.3078 | | -0.0871 | -0.0533 | 0.0289 | | 0.6247 | | 0.6786 |
|  | M | | -0.2569 | | 0.6797 | -0.5337 | 0.1924 | | 0.0097 | | 0.0071 |
|  | LF | | 0.0352 | | 0.3191 | 0.1313 | 0.8091 | | 0.1074 | | 0.3293 |
|  | HF | | 0.6360 | | -0.4687 | -0.2395 | 0.0049 | | 0.1003 | | 0.2369 |
|  | LM | | -0.0888 | | 0.6409 | -0.4341 | 0.6077 | | 0.0078 | | 0.0124 |
|  | HM | | -0.3480 | | 0.6485 | -0.6916 | 0.3150 | | 0.1742 | | 0.0482 |
| ***DMetS17a*** | | | | | | | | | | | |
| LOD^a^ | | POS^b^ | | Prox | | Dist | | Total Genes | | MetS Candidates^c^ | |
| 2.86 | | 25.5 | | 23.5 | | 25.9 | | 112 | | ***Pdpkl***^Li^, *Sstr5, Sox8, Abca3, Lmfl* | |
| Traits^d^ | Cohort^e^ | | a^f^ | | d | i | Pr(a) | | Pr(d) | | Pr(i) |
| AUC_10 | Full | | -76.4953 | | -309.5400 | 709.1800 | 0.7705 | | 0.3770 | | 0.0090 |
|  | L | | -0.8478 | | 448.6600 | 342.3000 | 0.9974 | | 0.2187 | | 0.2132 |
|  | H | | 26.1792 | | -947.1400 | 1129.5500 | 0.9514 | | 0.1095 | | 0.0114 |
|  | F | | 50.9864 | | 277.4100 | 115.4600 | 0.7938 | | 0.3348 | | 0.5894 |
|  | M | | -102.3600 | | -703.3600 | 1257.7300 | 0.8214 | | 0.2514 | | 0.0090 |
|  | LF | | 367.9900 | | 529.7000 | -213.2300 | 0.1320 | | 0.1493 | | 0.4387 |
|  | HF | | -254.5200 | | 60.0438 | 405.7500 | 0.3929 | | 0.8900 | | 0.2044 |
|  | LM | | -361.3700 | | 353.8400 | 842.8800 | 0.4181 | | 0.5626 | | 0.0717 |
|  | HM | | 419.4400 | | -1633.2200 | 1782.7600 | 0.5707 | | 0.1228 | | 0.0276 |
| K | Full | | 0.0021 | | -0.0074 | 0.0022 | 0.3735 | | 0.0126 | | 0.0033 |
|  | L | | 0.0030 | | -0.0032 | -0.0022 | 0.3337 | | 0.4270 | | 0.4844 |
|  | H | | 0.0007 | | -0.0100 | 0.0055 | 0.8260 | | 0.0196 | | 0.1148 |
|  | F | | 0.0001 | | -0.0009 | -0.0021 | 0.9456 | | 0.7559 | | 0.3578 |
|  | M | | 0.0039 | | -0.0130 | 0.0059 | 0.3285 | | 0.0107 | | 0.1535 |
|  | LF | | -0.0015 | | -0.0025 | -0.0022 | 0.5777 | | 0.5084 | | 0.4577 |
|  | HF | | -0.0001 | | 0.0017 | -0.0014 | 0.9783 | | 0.6621 | | 0.6530 |
|  | LM | | 0.0073 | | -0.0038 | -0.0023 | 0.1790 | | 0.5937 | | 0.6770 |
|  | HM | | 0.0009 | | -0.0184 | 0.0115 | 0.8732 | | 0.0119 | | 0.0462 |
| ***DMetS17b*** | | | | | | | | | | | |
| LOD^a^ | | POS^b^ | | Prox | | Dist | | Total Genes | | MetS Candidates^c^ | |
| 3.24 | | 31.2 | | 29.8 | | 33.2 | | 56 | | *Abcg1, Glp1r, Cbs* | |
| Traits^d^ | Cohort^e^ | | a^f^ | | d | i | Pr(a) | | Pr(d) | | Pr(i) |
| G | Full | | 0.1827 | | -0.08844 | -0.03415 | 0.023 | | 0.4107 | | 0.6865 |
|  | L | | 0.03548 | | -0.1763 | 0.04026 | 0.601 | | 0.0561 | | 0.5573 |
|  | H | | 0.3274 | | -0.02984 | 0.008476 | 0.0172 | | 0.8782 | | 0.9551 |
|  | F | | 0.4352 | | 0.07233 | -0.05061 | 0.0022 | | 0.7021 | | 0.7353 |
|  | M | | -0.04046 | | -0.2076 | -0.00402 | 0.5486 | | 0.0291 | | 0.9551 |
|  | LF | | 0.1816 | | -0.1939 | 0.0282 | 0.1018 | | 0.2033 | | 0.8018 |
|  | HF | | 0.6473 | | 0.3037 | 0.05788 | 0.0087 | | 0.379 | | 0.8313 |
|  | LM | | -0.1058 | | -0.1307 | 0.06008 | 0.1517 | | 0.1998 | | 0.4197 |
|  | HM | | 0.0248 | | -0.3229 | -0.06003 | 0.812 | | 0.0427 | | 0.5996 |
| M | Full | | 0.06281 | | -0.08877 | -0.01798 | 0.0235 | | 0.014 | | 0.522 |
|  | L | | 0.04916 | | -0.09076 | -0.02065 | 0.0919 | | 0.0211 | | 0.4643 |
|  | H | | 0.0822 | | -0.08974 | -0.00057 | 0.0597 | | 0.1288 | | 0.9901 |
|  | F | | 0.1299 | | -0.02149 | -0.00726 | 0.0007 | | 0.676 | | 0.8518 |
|  | M | | -0.00666 | | -0.1199 | -0.01943 | 0.853 | | 0.0137 | | 0.5959 |
|  | LF | | 0.06445 | | -0.04224 | -0.01623 | 0.0946 | | 0.4148 | | 0.6544 |
|  | HF | | 0.1763 | | 0.001104 | 0.03955 | 0.0039 | | 0.99 | | 0.5609 |
|  | LM | | 0.03029 | | -0.1298 | -0.01808 | 0.4599 | | 0.025 | | 0.6585 |
|  | HM | | -0.04182 | | -0.09465 | -0.02122 | 0.4462 | | 0.2081 | | 0.7058 |
| T | Full | | 0.4469 | | -0.2672 | 0.04524 | 0.0345 | | 0.3521 | | 0.8369 |
|  | L | | 0.1679 | | -0.4734 | 0.008938 | 0.39 | | 0.0826 | | 0.9633 |
|  | H | | 0.7349 | | -0.1277 | 0.2617 | 0.0347 | | 0.798 | | 0.4894 |
|  | F | | 1.1701 | | 0.2194 | -0.00125 | 0.0006 | | 0.6369 | | 0.9972 |
|  | M | | -0.2012 | | -0.5393 | 0.09746 | 0.3675 | | 0.0862 | | 0.6748 |
|  | LF | | 0.4904 | | -0.4832 | 0.03 | 0.0937 | | 0.2377 | | 0.9173 |
|  | HF | | 1.6311 | | 0.7979 | 0.3142 | 0.0035 | | 0.3325 | | 0.6129 |
|  | LM | | -0.1502 | | -0.3825 | 0.04089 | 0.5433 | | 0.2786 | | 0.8691 |
|  | HM | | -0.2434 | | -0.5644 | 0.1883 | 0.4877 | | 0.272 | | 0.6155 |
| N | Full | | 1.54 | | -0.4886 | 0.2647 | 0.00 | | 0.3972 | | 0.5736 |
|  | L | | 1.04 | | -1.361 | 0.2218 | 0.03 | | 0.0349 | | 0.6517 |
|  | H | | 2.05 | | 0.5836 | 0.8064 | 0.00 | | 0.5419 | | 0.2976 |
|  | F | | 2.69 | | 0.47 | -0.1914 | <.0001 | | 0.5934 | | 0.7864 |
|  | M | | 0.52 | | -1.1061 | 0.8462 | 0.32 | | 0.1265 | | 0.1362 |
|  | LF | | 1.69 | | -0.861 | -0.2212 | 0.01 | | 0.331 | | 0.7356 |
|  | HF | | 3.24 | | 1.8068 | 0.7698 | 0.00 | | 0.2314 | | 0.5214 |
|  | LM | | 0.30 | | -1.7002 | 0.7999 | 0.66 | | 0.0643 | | 0.2423 |
|  | HM | | 0.71 | | 0.1015 | 0.8894 | 0.36 | | 0.9275 | | 0.3058 |
| Chol | Full | | 6.825 | | -1.3836 | 7.9515 | 0.0086 | | 0.669 | | 0.0027 |
|  | L | | 4.5033 | | -0.00404 | 4.75 | 0.0867 | | 0.999 | | 0.0627 |
|  | H | | 9.0896 | | -0.8325 | 12.8387 | 0.028 | | 0.8781 | | 0.0038 |
|  | F | | 7.8145 | | -1.6879 | 7.1869 | 0.0218 | | 0.706 | | 0.0397 |
|  | M | | 3.7588 | | -2.137 | 8.8877 | 0.2882 | | 0.6389 | | 0.0151 |
|  | LF | | 6.6984 | | -2.8859 | 4.2543 | 0.0583 | | 0.5271 | | 0.203 |
|  | HF | | 7.6859 | | 0.2237 | 14.1897 | 0.1428 | | 0.9765 | | 0.0187 |
|  | LM | | 1.917 | | 3.1492 | 4.6596 | 0.586 | | 0.5063 | | 0.187 |
|  | HM | | 6.3079 | | -2.2514 | 13.8989 | 0.2697 | | 0.7624 | | 0.0189 |
| ***DMetS18a*** | | | | | | | | | | | |
| LOD^a^ | | POS^b^ | | Prox | | Dist | | Total Genes | | MetS Candidates^c^ | |
| 2.67 | | 72.4 | | 71.2 | | 73.5 | | 5 | | *Dcc* | |
| Traits^d^ | Cohort^e^ | | a^f^ | | d | i | Pr(a) | | Pr(d) | | Pr(i) |
| Chol | Full | | 2.5905 | | 8.0702 | 4.0289 | 0.3072 | | 0.0136 | | 0.1161 |
|  | L | | 3.5061 | | 1.6184 | 1.3397 | 0.1561 | | 0.6380 | | 0.6154 |
|  | H | | 0.3551 | | 14.2947 | 8.3123 | 0.9327 | | 0.0085 | | 0.0396 |
|  | F | | 3.3875 | | 12.3411 | 2.1328 | 0.3129 | | 0.0077 | | 0.5430 |
|  | M | | 1.1809 | | 2.7983 | 8.2624 | 0.7297 | | 0.5341 | | 0.0154 |
|  | LF | | 3.2564 | | 7.8356 | 2.8917 | 0.3134 | | 0.1014 | | 0.4339 |
|  | HF | | 3.6364 | | 18.5245 | 3.1525 | 0.5246 | | 0.0185 | | 0.5661 |
|  | LM | | 3.5509 | | -5.0524 | 1.7743 | 0.2932 | | 0.2948 | | 0.6224 |
|  | HM | | -4.6094 | | 8.2379 | 14.9440 | 0.4096 | | 0.2687 | | 0.0053 |
| T | Full | | 0.4429 | | 0.1701 | 0.0094 | 0.0236 | | 0.4823 | | 0.9621 |
|  | L | | 0.3312 | | -0.1859 | 0.1165 | 0.0575 | | 0.4290 | | 0.5314 |
|  | H | | 0.7234 | | 0.6341 | 0.0471 | 0.0302 | | 0.1320 | | 0.8839 |
|  | F | | 0.5442 | | 0.8763 | 0.3214 | 0.0830 | | 0.0281 | | 0.3149 |
|  | M | | 0.3115 | | -0.3527 | -0.1922 | 0.1315 | | 0.1834 | | 0.3555 |
|  | LF | | 0.3367 | | 0.1707 | 0.3359 | 0.1826 | | 0.6218 | | 0.2455 |
|  | HF | | 1.2260 | | 1.6463 | 0.5413 | 0.0311 | | 0.0228 | | 0.2935 |
|  | LM | | 0.3058 | | -0.5011 | -0.0261 | 0.1798 | | 0.1068 | | 0.9097 |
|  | HM | | 0.2299 | | -0.1321 | -0.3574 | 0.4756 | | 0.7565 | | 0.2754 |
| ***DMetS19a*** | | | | | | | | | | | |
| LOD^a^ | | POS^b^ | | Prox | | Dist | | Total Genes | | MetS Candidates^c^ | |
| **4.06** | | 14.8 | | 11.9 | | 16.6 | | 29 | | *Cntf* | |
| Traits^d^ | Cohort^e^ | | a^f^ | | d | i | Pr(a) | | Pr(d) | | Pr(i) |
| K | Full | | -0.0015 | | 0.0042 | 0.0070 | 0.5243 | | 0.1809 | | 0.0065 |
|  | L | | -0.0029 | | 0.0002 | 0.0063 | 0.3506 | | 0.9678 | | 0.0612 |
|  | H | | 0.0017 | | 0.0080 | 0.0093 | 0.6088 | | 0.0741 | | 0.0108 |
|  | F | | -0.0001 | | -0.0012 | 0.0020 | 0.9689 | | 0.6744 | | 0.3872 |
|  | M | | -0.0023 | | 0.0097 | 0.0130 | 0.5732 | | 0.0758 | | 0.0035 |
|  | LF | | -0.0035 | | 0.0021 | 0.0044 | 0.2112 | | 0.6004 | | 0.1572 |
|  | HF | | 0.0023 | | -0.0034 | -0.0001 | 0.4209 | | 0.4123 | | 0.9847 |
|  | LM | | -0.0018 | | -0.0022 | 0.0091 | 0.7429 | | 0.7734 | | 0.1218 |
|  | HM | | 0.0002 | | 0.0199 | 0.0189 | 0.9761 | | 0.0100 | | 0.0021 |
| G | Full | | 0.1707 | | 0.2571 | 0.0455 | 0.0244 | | 0.0116 | | 0.5673 |
|  | L | | 0.0309 | | 0.0804 | 0.0860 | 0.6296 | | 0.3694 | | 0.2022 |
|  | H | | 0.3185 | | 0.5155 | 0.0192 | 0.0130 | | 0.0042 | | 0.8899 |
|  | F | | 0.3174 | | 0.3577 | -0.0003 | 0.0169 | | 0.0475 | | 0.9985 |
|  | M | | 0.0523 | | 0.1819 | 0.0993 | 0.4131 | | 0.0434 | | 0.1464 |
|  | LF | | 0.0389 | | 0.1320 | 0.1756 | 0.7015 | | 0.3692 | | 0.1094 |
|  | HF | | 0.5770 | | 0.7513 | -0.1253 | 0.0152 | | 0.0218 | | 0.6138 |
|  | LM | | 0.0190 | | 0.0452 | 0.0077 | 0.7912 | | 0.6517 | | 0.9184 |
|  | HM | | 0.1441 | | 0.3408 | 0.1511 | 0.1268 | | 0.0181 | | 0.1515 |
| T | Full | | 0.4179 | | 0.6855 | 0.2443 | 0.0313 | | 0.0093 | | 0.2321 |
|  | L | | 0.0543 | | 0.0656 | 0.4005 | 0.7618 | | 0.7977 | | 0.0359 |
|  | H | | 0.7615 | | 1.4898 | 0.1421 | 0.0159 | | 0.0009 | | 0.6759 |
|  | F | | 0.7770 | | 0.9036 | 0.1873 | 0.0123 | | 0.0360 | | 0.5659 |
|  | M | | 0.1227 | | 0.5245 | 0.3276 | 0.5522 | | 0.0698 | | 0.1382 |
|  | LF | | 0.0386 | | 0.2350 | 0.5764 | 0.8822 | | 0.5385 | | 0.0420 |
|  | HF | | 1.3563 | | 1.9439 | -0.1253 | 0.0113 | | 0.0110 | | 0.8230 |
|  | LM | | 0.0437 | | -0.0474 | 0.2357 | 0.8510 | | 0.8868 | | 0.3384 |
|  | HM | | 0.3309 | | 1.1781 | 0.4225 | 0.2858 | | 0.0100 | | 0.2200 |
| N | Full | | 1.4209 | | 1.3236 | 0.4046 | 0.0005 | | 0.0095 | | 0.3431 |
|  | L | | 0.4013 | | 0.4000 | 1.2222 | 0.3566 | | 0.4921 | | 0.0088 |
|  | H | | 2.6649 | | 2.5550 | -0.1399 | <.0001 | | 0.0018 | | 0.8365 |
|  | F | | 1.7585 | | 1.6287 | 0.3955 | 0.0036 | | 0.0375 | | 0.5339 |
|  | M | | 1.4043 | | 1.1782 | 0.7084 | 0.0034 | | 0.0655 | | 0.1731 |
|  | LF | | 0.3739 | | 0.5215 | 1.3640 | 0.5100 | | 0.5109 | | 0.0278 |
|  | HF | | 3.1901 | | 3.1902 | -0.5699 | 0.0016 | | 0.0161 | | 0.5938 |
|  | LM | | 0.4620 | | 0.4287 | 1.1204 | 0.4547 | | 0.6079 | | 0.0931 |
|  | HM | | 2.7119 | | 2.2755 | 0.5029 | <.0001 | | 0.0155 | | 0.4988 |
| L | Full | | 0.0914 | | 0.0622 | -0.0040 | 0.0009 | | 0.0969 | | 0.8988 |
|  | L | | 0.0400 | | 0.0348 | 0.0149 | 0.0522 | | 0.2189 | | 0.5202 |
|  | H | | 0.1538 | | 0.0940 | -0.0218 | 0.0013 | | 0.1684 | | 0.6995 |
|  | F | | 0.0914 | | 0.0752 | -0.0110 | 0.0083 | | 0.1163 | | 0.7727 |
|  | M | | 0.1067 | | 0.0630 | 0.0060 | 0.0056 | | 0.2630 | | 0.8978 |
|  | LF | | 0.0511 | | 0.0301 | 0.0073 | 0.0606 | | 0.4456 | | 0.8081 |
|  | HF | | 0.1405 | | 0.1311 | -0.0405 | 0.0208 | | 0.1298 | | 0.5586 |
|  | LM | | 0.0343 | | 0.0483 | 0.0261 | 0.2166 | | 0.2169 | | 0.4246 |
|  | HM | | 0.1904 | | 0.0778 | -0.0219 | 0.0049 | | 0.4532 | | 0.7974 |
| ^a^ LOD, logarithm of odds. The LOD score in the table refers to the highest score at the combined-trait position. Boldfaced type indicates genome-wide significance (LOD ≥ 3.97). All others are significant at their respective chromosome-wise threshold (Table S6).  ^b^ Genomic position in Mb (NCBI Build37/ mm9).  ^c^ Genes known to affect MetS components. Boldfaced type indicates genes that are differentially expressed between LG/J and SM/J in liver (L) or white fat (W). i: differentially expressed between the strains; ii: differentially expressed among sex*strain cohorts; iii: differentially expressed among diet*strain cohorts; iv: differentially expressed among sex*diet*strain cohorts.  ^d^ Chol: cholesterol; FFA: free-fatty acids; TG: triglycerides; INS: insulin; GLC: glucose; AUC_10: area under the curve at 10 weeks; AUC_20: area under the curve at 20 weeks; bGLC_10: fasting glucose at 10 weeks; bGLC_20: fasting glucose at 20 weeks; G: gonadal fatpad weight; I: inguinal fatpad weight; M: mesenteric fatpad weight; N: total weight at necropsy; R: renal fatpad weight; T: total fatpad weight; K: kidney weight; L = liver weight; S: spleen weight.  ^e^ Full: full F_16_ population; H: all high fat-fed individuals; L: all low-fat fed individuals; F: all females; M: all males; HF: high-fat fed females; HM: high-fat fed males; LF: low-fat fed females; LM: low-fat fed males.  ^f^ a: additive genetic effects; d: dominance genetic effects; i: imprinting genetic effects. | | | | | | | | | | | |
