# Supplementary material for: Genetic Effects at Pleiotropic Loci Are Context-Dependent with Consequences for the Maintenance of Genetic Variation in Populations
Source: PLoS Genet. 2011 Sep 8;7(9):e1002256. doi: 10.1371/journal.pgen.1002256 (PMC3169520; doi:10.1371/journal.pgen.1002256)
Supplement: Table S2 — Differential expression in MetS QTL among genes that are expressed in LG/J and SM/J strains in white fat tissue. (DOC) [file pgen.1002256.s004.doc]

| **Gene** | **Start** | **Stop** | **p-val**  **(Strain)** | **q-val**  **(Strain)** | **p-val**  **(Diet * Sex * Strain)** | **q-val**  **(Diet * Sex* Strain)** | **p-val**  **(Diet * Strain)** | **q-val**  **(Diet * Strain)** | **p-val**  **(Sex * Strain)** | **q-val**  **(Sex * Strain)** |
| --- | --- | --- | --- | --- | --- | --- | --- | --- | --- | --- |
| ***DMetS1a*** | | | | | | | | | | |
| Epha4 | 77363760 | 77511663 | 0.2137 | 0.4591 | 0.7534 | 0.7420 | 0.0048 | 0.1371 | 0.7293 | 0.6611 |
| ***DMetS1b*** | | | | | | | | | | |
| Rgs5 | 171585632 | 171625944 | 0.2532 | 0.4999 | 0.6117 | 0.7095 | 0.9929 | 0.7661 | 0.1111 | 0.4500 |
| Rgs4 | 171671608 | 171677773 | 0.2648 | 0.5076 | 0.8561 | 0.7682 | 0.1147 | 0.4200 | 0.2076 | 0.5019 |
| 1700084C01Rik | 171858779 | 171864784 | 0.0521 | 0.2327 | 0.1728 | 0.5008 | 0.8093 | 0.7248 | 0.0028 | 0.2251 |
| Hsd17b7 | 171879666 | 171899372 | 0.0175 | 0.1190 | 0.2160 | 0.5535 | 0.1697 | 0.4642 | 0.2825 | 0.5236 |
| Ddr2 | 171907777 | 172040690 | 0.7216 | 0.7375 | 0.0800 | 0.4040 | 0.5621 | 0.6815 | 0.5277 | 0.6206 |
| Uap1 | 172072069 | 172105088 | 0.4364 | 0.6325 | 0.2543 | 0.5849 | 0.5996 | 0.6943 | 0.1537 | 0.4703 |
| C820018A03Rik | 172123551 | 172145528 | 0.9395 | 0.7819 | 0.1340 | 0.4606 | 0.5437 | 0.6784 | 0.9544 | 0.7069 |
| Uhmk1 | 172123551 | 172145528 | 0.1459 | 0.3929 | 0.0290 | 0.2919 | 0.4185 | 0.6387 | 0.3677 | 0.5689 |
| 4930500M09Rik | 172144991 | 172145687 | 0.3411 | 0.5697 | 0.8037 | 0.7522 | 0.5204 | 0.6724 | 0.6731 | 0.6502 |
| Nos1ap | 172232799 | 172519992 | 0.3647 | 0.5866 | 0.8137 | 0.7550 | 0.6294 | 0.6999 | 0.0523 | 0.4020 |
| 1700015E13Rik | 172238992 | 172242258 | 0.0488 | 0.2269 | 0.2488 | 0.5797 | 0.9203 | 0.7505 | 0.7385 | 0.6611 |
| Olfml2b | 172574663 | 172612920 | 0.6249 | 0.7145 | 0.1405 | 0.4657 | 0.0277 | 0.2765 | 0.1052 | 0.4486 |
| Atf6 | 172634588 | 172797902 | 0.9768 | 0.7878 | 0.9709 | 0.7902 | 0.7801 | 0.7211 | 0.7500 | 0.6611 |
| Fcrla | 172847707 | 172857714 | 0.8955 | 0.7733 | 0.1323 | 0.4595 | 0.2903 | 0.5678 | 0.0414 | 0.3869 |
| Fcgr2b | 172890689 | 172906678 | 0.0005 | **0.0067** | 0.0164 | 0.2352 | 0.0236 | 0.2551 | 0.9306 | 0.6990 |
| Fcgr4 | 172949051 | 172959892 | 0.0027 | **0.0276** | 0.0138 | 0.2351 | 0.9813 | 0.7641 | 0.6062 | 0.6413 |
| Fcgr3 | 172981301 | 172989493 | 0.1584 | 0.3967 | 0.0640 | 0.3722 | 0.0632 | 0.3564 | 0.4471 | 0.5963 |
| 1700009P17Rik | 173044049 | 173057098 | 0.5006 | 0.6617 | 0.3973 | 0.6536 | 0.7840 | 0.7212 | 0.3313 | 0.5582 |
| Sdhc | 173057296 | 173080734 | 0.0378 | 0.1950 | 0.0198 | 0.2521 | 0.0345 | 0.2958 | 0.3965 | 0.5823 |
| Mpz | 173080842 | 173091261 | 0.0774 | 0.2898 | 0.4996 | 0.6752 | 0.8029 | 0.7235 | 0.4728 | 0.6030 |
| Pcp4l1 | 173103393 | 173126399 | 0.6618 | 0.7242 | 0.7048 | 0.7340 | 0.6838 | 0.7111 | 0.7667 | 0.6637 |
| Nr1i3 | 173144101 | 173150832 | 0.0000 | **0.0000** | 0.0033 | 0.1402 | 0.0001 | **0.0259** | 0.0062 | 0.2769 |
| Apoa2 | 173155185 | 173156510 | 0.2924 | 0.5292 | 0.3468 | 0.6251 | 0.5408 | 0.6784 | 0.8358 | 0.6809 |
| Ndufs2 | 173164984 | 173177253 | 0.0041 | **0.0381** | 0.0003 | **0.0349** | 0.0007 | 0.0724 | 0.0173 | 0.3041 |
| Adamts4 | 173180552 | 173190768 | 0.0019 | **0.0216** | 0.2297 | 0.5634 | 0.9168 | 0.7505 | 0.7791 | 0.6642 |
| B4galt3 | 173200459 | 173207027 | 0.2123 | 0.4591 | 0.7326 | 0.7395 | 0.9787 | 0.7641 | 0.6324 | 0.6456 |
| Ppox | 173206121 | 173211317 | 0.0076 | 0.0655 | 0.9137 | 0.7791 | 0.2684 | 0.5484 | 0.0907 | 0.4290 |
| Usp23 | 173212076 | 173218122 | 0.0145 | 0.1072 | 0.2034 | 0.5364 | 0.5996 | 0.6943 | 0.6331 | 0.6456 |
| Usp21 | 173212076 | 173218122 | 0.0000 | **0.0000** | 0.7523 | 0.7420 | 0.1265 | 0.4370 | 0.6112 | 0.6424 |
| Ufc1 | 173218694 | 173225155 | 0.1549 | 0.3962 | 0.7384 | 0.7413 | 0.6131 | 0.6943 | 0.6819 | 0.6512 |
| EG622236 | 173235215 | 173235433 | 0.9737 | 0.7871 | 0.2047 | 0.5383 | 0.0558 | 0.3444 | 0.3197 | 0.5507 |
| Dedd | 173259276 | 173272462 | 0.0148 | 0.1079 | 0.4615 | 0.6635 | 0.0010 | 0.0724 | 0.6216 | 0.6440 |
| Nit1 | 173268139 | 173275777 | 0.0024 | **0.0257** | 0.4520 | 0.6611 | 0.9189 | 0.7505 | 0.0489 | 0.4020 |
| Pfdn2 | 173275801 | 173289385 | 0.3687 | 0.5894 | 0.1143 | 0.4471 | 0.0192 | 0.2478 | 0.5451 | 0.6220 |
| Klhdc9 | 173288577 | 173290929 | 0.0222 | 0.1424 | 0.5552 | 0.6882 | 0.4385 | 0.6467 | 0.1974 | 0.5003 |
| Pvrl4 | 173300230 | 173318729 | 0.3151 | 0.5464 | 0.4059 | 0.6536 | 0.0963 | 0.4073 | 0.1156 | 0.4528 |
| Arhgap30 | 173319085 | 173340429 | 0.0115 | 0.0918 | 0.0138 | 0.2351 | 0.4543 | 0.6490 | 0.2396 | 0.5100 |
| Usf1 | 173341444 | 173349273 | 0.0000 | **0.0000** | 0.6594 | 0.7186 | 0.8453 | 0.7350 | 0.4666 | 0.6016 |
| EG226654 | 173349164 | 173350485 | 0.4600 | 0.6407 | 0.1657 | 0.4940 | 0.3542 | 0.6139 | 0.9748 | 0.7100 |
| F11r | 173367666 | 173394734 | 0.0000 | **0.0000** | 0.0520 | 0.3559 | 0.1527 | 0.4642 | 0.2284 | 0.5057 |
| Refbp2 | 173433609 | 173434881 | 0.0088 | 0.0741 | 0.4340 | 0.6563 | 0.3667 | 0.6193 | 0.4828 | 0.6084 |
| Cd244 | 173489324 | 173515449 | 0.6822 | 0.7311 | 0.5868 | 0.7056 | 0.2873 | 0.5643 | 0.7981 | 0.6696 |
| Ly9 | 173518755 | 173537541 | 0.6428 | 0.7186 | 0.0148 | 0.2351 | 0.7675 | 0.7211 | 0.0511 | 0.4020 |
| ***DMetS2a*** | | | | | | | | | | |
| 4932414N04Rik | 68494543 | 68586520 | 0.2863 | 0.5253 | 0.5041 | 0.6756 | 0.9911 | 0.7659 | 0.2341 | 0.5080 |
| Lass6 | 68699498 | 68952339 | 0.8880 | 0.7719 | 0.2624 | 0.5938 | 0.2254 | 0.5177 | 0.5423 | 0.6215 |
| Nostrin | 68973857 | 69027387 | 0.1191 | 0.3646 | 0.1592 | 0.4895 | 0.8685 | 0.7422 | 0.4731 | 0.6030 |
| Spc25 | 69031952 | 69044251 | 0.6013 | 0.7095 | 0.0964 | 0.4272 | 0.0197 | 0.2481 | 0.1008 | 0.4413 |
| G6pc2 | 69049130 | 69065898 | 0.3462 | 0.5733 | 0.6568 | 0.7186 | 0.1409 | 0.4525 | 0.7831 | 0.6644 |
| Dhrs9 | 69218502 | 69242590 | 0.1487 | 0.3944 | 0.2450 | 0.5772 | 0.0606 | 0.3519 | 0.1608 | 0.4703 |
| Lrp2 | 69262397 | 69424122 | 0.2534 | 0.4999 | 0.5221 | 0.6790 | 0.2819 | 0.5595 | 0.3668 | 0.5689 |
| Bbs5 | 69485228 | 69505628 | 0.8104 | 0.7617 | 0.8597 | 0.7682 | 0.4623 | 0.6513 | 0.9864 | 0.7149 |
| Kbtbd10 | 69508177 | 69522287 | 0.6607 | 0.7242 | 0.1329 | 0.4603 | 0.1150 | 0.4200 | 0.8023 | 0.6708 |
| Fastkd1 | 69524872 | 69551573 | 0.0362 | 0.1941 | 0.0000 | **0.0042** | 0.1208 | 0.4287 | 0.0220 | 0.3226 |
| Ppig | 69560602 | 69592069 | 0.5961 | 0.7071 | 0.7023 | 0.7340 | 0.5845 | 0.6919 | 0.3109 | 0.5430 |
| Phospho2 | 69627680 | 69638062 | 0.5612 | 0.6878 | 0.0112 | 0.2301 | 0.6415 | 0.7064 | 0.2126 | 0.5019 |
| Klhl23 | 69660001 | 69674708 | 0.1499 | 0.3949 | 0.3249 | 0.6083 | 0.3385 | 0.6037 | 0.1595 | 0.4703 |
| Myo3b | 69921131 | 70267255 | 0.0599 | 0.2521 | 0.9432 | 0.7880 | 0.9503 | 0.7588 | 0.0520 | 0.4020 |
| Sp5 | 70312980 | 70315786 | 0.7090 | 0.7375 | 0.3507 | 0.6273 | 0.4672 | 0.6534 | 0.7089 | 0.6568 |
| 1500002O10Rik | 70327997 | 70401414 | 0.9967 | 0.7909 | 0.9569 | 0.7881 | 0.4196 | 0.6395 | 0.6820 | 0.6512 |
| 4933404M02Rik | 70346876 | 70378941 | 0.7002 | 0.7335 | 0.2886 | 0.5994 | 0.9895 | 0.7655 | 0.0968 | 0.4374 |
| Gad1 | 70391129 | 70440071 | 0.9890 | 0.7909 | 0.0739 | 0.3974 | 0.0391 | 0.3081 | 0.8499 | 0.6854 |
| Gorasp2 | 70499633 | 70550693 | 0.0792 | 0.2925 | 0.8170 | 0.7550 | 0.1603 | 0.4642 | 0.6164 | 0.6436 |
| Tlk1 | 70550464 | 70663785 | 0.7204 | 0.7375 | 0.9256 | 0.7816 | 0.9196 | 0.7505 | 0.7573 | 0.6611 |
| Mettl8 | 70802618 | 70893640 | 0.0492 | 0.2273 | 0.2856 | 0.5994 | 0.2644 | 0.5445 | 0.1431 | 0.4703 |
| 4833418A01Rik | 70893385 | 70937199 | 0.0555 | 0.2402 | 0.7151 | 0.7374 | 0.3568 | 0.6139 | 0.0384 | 0.3810 |
| Cybrd1 | 70955980 | 70980983 | 0.3790 | 0.5957 | 0.0026 | 0.1206 | 0.0544 | 0.3415 | 0.5932 | 0.6375 |
| Dync1i2 | 71049763 | 71101360 | 0.0376 | 0.1950 | 0.0239 | 0.2667 | 0.3378 | 0.6033 | 0.9227 | 0.6971 |
| Slc25a12 | 71109120 | 71205806 | 0.0149 | 0.1079 | 0.4553 | 0.6620 | 0.0889 | 0.3905 | 0.0172 | 0.3041 |
| Hat1 | 71227015 | 71279679 | 0.2936 | 0.5307 | 0.5927 | 0.7072 | 0.9615 | 0.7614 | 0.3363 | 0.5586 |
| Metapl1 | 71291333 | 71363251 | 0.0056 | **0.0492** | 0.0000 | **0.0023** | 0.0003 | **0.0473** | 0.0090 | 0.2769 |
| Dlx1as | 71354511 | 71375962 | 0.6315 | 0.7158 | 0.5802 | 0.7020 | 0.4835 | 0.6593 | 0.7572 | 0.6611 |
| Dlx2 | 71381466 | 71384749 | 0.6128 | 0.7141 | 0.8804 | 0.7701 | 0.4327 | 0.6467 | 0.2783 | 0.5207 |
| Gm1631 | 71557446 | 71569023 | 0.0000 | **0.0004** | 0.7212 | 0.7374 | 0.4708 | 0.6535 | 0.0154 | 0.3041 |
| Itga6 | 71583673 | 71696473 | 0.0000 | **0.0009** | 0.6595 | 0.7186 | 0.0326 | 0.2925 | 0.7835 | 0.6644 |
| Pdk1 | 71711281 | 71741915 | 0.0088 | 0.0741 | 0.0039 | 0.1509 | 0.2044 | 0.4962 | 0.1233 | 0.4657 |
| 6330581N18Rik | 71819297 | 72095531 | 0.0019 | **0.0210** | 0.2913 | 0.6006 | 0.6476 | 0.7097 | 0.3104 | 0.5430 |
| Rapgef4 | 71819297 | 72095531 | 0.1834 | 0.4284 | 0.2734 | 0.5993 | 0.3871 | 0.6250 | 0.0552 | 0.4020 |
| ***DMetS2b*** | | | | | | | | | | |
| Zc3h15 | 83484592 | 83504779 | 0.6930 | 0.7329 | 0.5907 | 0.7066 | 0.8439 | 0.7350 | 0.5287 | 0.6206 |
| Itgav | 83564554 | 83647073 | 0.5147 | 0.6676 | 0.5947 | 0.7072 | 0.3878 | 0.6250 | 0.3817 | 0.5763 |
| Fam171b | 83652793 | 83723643 | 0.0000 | **0.0000** | 0.0076 | 0.2219 | 0.9762 | 0.7641 | 0.4019 | 0.5823 |
| Zswim2 | 83755236 | 83781385 | 0.0716 | 0.2811 | 0.4903 | 0.6732 | 0.2619 | 0.5420 | 0.0171 | 0.3041 |
| Tfpi | 84273012 | 84316932 | 0.9043 | 0.7733 | 0.0338 | 0.3040 | 0.0085 | 0.1725 | 0.2049 | 0.5019 |
| Catns | 84440228 | 84490922 | 0.9280 | 0.7808 | 0.2138 | 0.5497 | 0.4098 | 0.6326 | 0.0277 | 0.3411 |
| 2700094K13Rik | 84509375 | 84510927 | 0.0880 | 0.3069 | 0.1922 | 0.5179 | 0.0726 | 0.3725 | 0.3857 | 0.5787 |
| Med19 | 84518559 | 84528372 | 0.0315 | 0.1740 | 0.5336 | 0.6800 | 0.8463 | 0.7350 | 0.4062 | 0.5847 |
| Zdhhc5 | 84528127 | 84555337 | 0.8434 | 0.7653 | 0.2836 | 0.5994 | 0.4404 | 0.6467 | 0.6106 | 0.6423 |
| Clp1 | 84563279 | 84567507 | 0.3196 | 0.5496 | 0.4467 | 0.6611 | 0.3570 | 0.6139 | 0.4736 | 0.6030 |
| Ypel4 | 84574215 | 84578812 | 0.1827 | 0.4283 | 0.3890 | 0.6532 | 0.5380 | 0.6784 | 0.0049 | 0.2616 |
| Serping1 | 84605544 | 84615601 | 0.0000 | **0.0005** | 0.0046 | 0.1668 | 0.0061 | 0.1453 | 0.3039 | 0.5384 |
| Ube2l6 | 84638985 | 84650492 | 0.0363 | 0.1941 | 0.7197 | 0.7374 | 0.0154 | 0.2260 | 0.5208 | 0.6205 |
| Smtnl1 | 84651333 | 84662809 | 0.8718 | 0.7719 | 0.0152 | 0.2352 | 0.0171 | 0.2394 | 0.3401 | 0.5586 |
| Timm10 | 84667154 | 84670370 | 0.0279 | 0.1659 | 0.1560 | 0.4856 | 0.2866 | 0.5643 | 0.6862 | 0.6514 |
| Slc43a1 | 84679007 | 84703751 | 0.0447 | 0.2157 | 0.0846 | 0.4148 | 0.7682 | 0.7211 | 0.8769 | 0.6908 |
| Rtn4rl2 | 84712081 | 84726867 | 0.6969 | 0.7329 | 0.7956 | 0.7506 | 0.0436 | 0.3081 | 0.0124 | 0.3005 |
| Slc43a3 | 84776736 | 84798666 | 0.8125 | 0.7620 | 0.7350 | 0.7402 | 0.9648 | 0.7614 | 0.8776 | 0.6908 |
| Prg2 | 84820618 | 84823789 | 0.0217 | 0.1400 | 0.1746 | 0.5008 | 0.0039 | 0.1334 | 0.6963 | 0.6523 |
| P2rx3 | 84838740 | 84877619 | 0.7101 | 0.7375 | 0.7449 | 0.7420 | 0.3152 | 0.5865 | 0.3542 | 0.5680 |
| Ssrp1 | 84877391 | 84887266 | 0.5025 | 0.6633 | 0.2181 | 0.5548 | 0.0511 | 0.3323 | 0.3022 | 0.5374 |
| Tnks1bp1 | 84888179 | 84913205 | 0.0783 | 0.2917 | 0.3358 | 0.6159 | 0.2961 | 0.5695 | 0.6242 | 0.6443 |
| Aplnr | 84976557 | 84980080 | 0.0004 | **0.0056** | 0.3108 | 0.6076 | 0.0221 | 0.2551 | 0.0894 | 0.4290 |
| Olfr988 | 85193152 | 85203215 | 0.1967 | 0.4431 | 0.8594 | 0.7682 | 0.1274 | 0.4370 | 0.1483 | 0.4703 |
| Olfr996 | 85419398 | 85420342 | 0.8835 | 0.7719 | 0.0593 | 0.3706 | 0.5233 | 0.6733 | 0.2243 | 0.5051 |
| Olfr1006 | 85514368 | 85515330 | 0.5619 | 0.6878 | 0.8095 | 0.7550 | 0.1517 | 0.4642 | 0.4544 | 0.5968 |
| Olfr1018 | 85663130 | 85664065 | 0.0762 | 0.2871 | 0.3090 | 0.6058 | 0.9612 | 0.7614 | 0.0973 | 0.4374 |
| Olfr1020 | 85689611 | 85690564 | 0.5041 | 0.6640 | 0.1411 | 0.4657 | 0.0983 | 0.4095 | 0.0431 | 0.3950 |
| Olfr1023 | 85726959 | 85727894 | 0.9185 | 0.7799 | 0.7565 | 0.7420 | 0.6886 | 0.7111 | 0.9642 | 0.7084 |
| Olfr1031 | 85831976 | 85832986 | 0.1839 | 0.4284 | 0.7393 | 0.7413 | 0.6151 | 0.6943 | 0.6819 | 0.6512 |
| Olfr1043 | 86002160 | 86003104 | 0.0678 | 0.2703 | 0.5216 | 0.6790 | 0.0746 | 0.3725 | 0.0141 | 0.3041 |
| Olfr1065 | 86285196 | 86286137 | 0.3814 | 0.5957 | 0.9125 | 0.7791 | 0.0278 | 0.2765 | 0.2443 | 0.5113 |
| Olfr1085 | 86497672 | 86498613 | 0.5739 | 0.6943 | 0.7302 | 0.7389 | 0.5600 | 0.6815 | 0.7260 | 0.6598 |
| Olfr1105 | 86873438 | 86874376 | 0.1374 | 0.3812 | 0.5645 | 0.6945 | 0.0828 | 0.3861 | 0.4268 | 0.5892 |
| Olfr259 | 86947604 | 86948542 | 0.9325 | 0.7819 | 0.4258 | 0.6536 | 0.5766 | 0.6894 | 0.4338 | 0.5892 |
| Olfr1110 | 86975538 | 86976476 | 0.8779 | 0.7719 | 0.9496 | 0.7880 | 0.7919 | 0.7213 | 0.1378 | 0.4703 |
| Olfr1112 | 87031846 | 87032802 | 0.3473 | 0.5745 | 0.6132 | 0.7095 | 0.0352 | 0.2983 | 0.1649 | 0.4733 |
| Olfr1113 | 87053051 | 87054031 | 0.1866 | 0.4320 | 0.0560 | 0.3700 | 0.1008 | 0.4095 | 0.8611 | 0.6874 |
| Olfr1132 | 87474976 | 87475902 | 0.4455 | 0.6350 | 0.7208 | 0.7374 | 0.8081 | 0.7246 | 0.0761 | 0.4214 |
| Olfr1133 | 87485337 | 87486278 | 0.7596 | 0.7420 | 0.1845 | 0.5111 | 0.4965 | 0.6612 | 0.8103 | 0.6731 |
| Olfr1154 | 87742899 | 87743831 | 0.6242 | 0.7145 | 0.1948 | 0.5196 | 0.2386 | 0.5294 | 0.3788 | 0.5763 |
| Olfr1155 | 87782839 | 87783783 | 0.2640 | 0.5074 | 0.2638 | 0.5940 | 0.3961 | 0.6322 | 0.4924 | 0.6114 |
| Olfr1158 | 87830270 | 87831211 | 0.1920 | 0.4383 | 0.2964 | 0.6021 | 0.2697 | 0.5497 | 0.9271 | 0.6986 |
| Olfr1160 | 87845974 | 87846933 | 0.8324 | 0.7637 | 0.8636 | 0.7682 | 0.4433 | 0.6467 | 0.2531 | 0.5124 |
| Olfr1161 | 87864881 | 87865846 | 0.1573 | 0.3963 | 0.0639 | 0.3722 | 0.0313 | 0.2925 | 0.4195 | 0.5871 |
| Olfr1166 | 87964190 | 87965140 | 0.3718 | 0.5906 | 0.7235 | 0.7374 | 0.5050 | 0.6653 | 0.9992 | 0.7174 |
| Olfr1167 | 87989224 | 87990174 | 0.2217 | 0.4662 | 0.5988 | 0.7072 | 0.2915 | 0.5678 | 0.2598 | 0.5160 |
| ***DMetS2c*** | | | | | | | | | | |
| Cd44 | 102651298 | 102741767 | 0.3419 | 0.5704 | 0.0301 | 0.2945 | 0.1242 | 0.4349 | 0.4238 | 0.5881 |
| Apip | 102913832 | 102932801 | 0.2730 | 0.5125 | 0.0140 | 0.2351 | 0.0987 | 0.4095 | 0.2405 | 0.5100 |
| Ehf | 103103590 | 103143435 | 0.9597 | 0.7844 | 0.4534 | 0.6611 | 0.2762 | 0.5536 | 0.0703 | 0.4190 |
| Elf5 | 103251870 | 103291146 | 0.3650 | 0.5866 | 0.8259 | 0.7575 | 0.8654 | 0.7422 | 0.2066 | 0.5019 |
| Cat | 103294006 | 103325317 | 0.0000 | **0.0000** | 0.9497 | 0.7880 | 0.4753 | 0.6555 | 0.8919 | 0.6923 |
| Nat10 | 103561413 | 103601427 | 0.9409 | 0.7819 | 0.4534 | 0.6611 | 0.1059 | 0.4159 | 0.8296 | 0.6780 |
| Caprin1 | 103603098 | 103637806 | 0.2599 | 0.5046 | 0.7711 | 0.7424 | 0.5908 | 0.6926 | 0.9689 | 0.7097 |
| 4930547E08Rik | 103644609 | 103650779 | 0.1334 | 0.3789 | 0.5821 | 0.7022 | 0.6109 | 0.6943 | 0.7178 | 0.6581 |
| Lmo2 | 103798143 | 103822031 | 0.4449 | 0.6350 | 0.1198 | 0.4543 | 0.1700 | 0.4642 | 0.1500 | 0.4703 |
| 4931422A03Rik | 103807100 | 103868518 | 0.9823 | 0.7884 | 0.3948 | 0.6536 | 0.2620 | 0.5420 | 0.2291 | 0.5057 |
| Fbxo3 | 103867878 | 103903397 | 0.2420 | 0.4915 | 0.8285 | 0.7575 | 0.6907 | 0.7111 | 0.4618 | 0.5987 |
| Cd59b | 103910006 | 103931344 | 0.0392 | 0.1978 | 0.6046 | 0.7085 | 0.7285 | 0.7146 | 0.6358 | 0.6456 |
| Cd59a | 103935958 | 103955511 | 0.0536 | 0.2348 | 0.4888 | 0.6732 | 0.9833 | 0.7649 | 0.3560 | 0.5689 |
| A930018P22Rik | 103962926 | 103964906 | 0.4210 | 0.6230 | 0.0106 | 0.2276 | 0.5464 | 0.6791 | 0.0306 | 0.3488 |
| D430041D05Rik | 103983230 | 104250150 | 0.5903 | 0.7026 | 0.7275 | 0.7375 | 0.7444 | 0.7169 | 0.8272 | 0.6774 |
| Hipk3 | 104266638 | 104334603 | 0.5254 | 0.6710 | 0.0954 | 0.4272 | 0.2033 | 0.4952 | 0.6207 | 0.6438 |
| Cstf3 | 104430680 | 104505586 | 0.0000 | **0.0000** | 0.2965 | 0.6021 | 0.6045 | 0.6943 | 0.2967 | 0.5331 |
| EG622282 | 104551832 | 104552931 | 0.7241 | 0.7375 | 0.3744 | 0.6477 | 0.8540 | 0.7387 | 0.4013 | 0.5823 |
| 4732486I23Rik | 104594952 | 104656853 | 0.0000 | **0.0000** | 0.1653 | 0.4940 | 0.3697 | 0.6193 | 0.3687 | 0.5689 |
| 2310047K21Rik | 104656991 | 104659148 | 0.0005 | **0.0071** | 0.4400 | 0.6587 | 0.1875 | 0.4805 | 0.3881 | 0.5789 |
| Ga17 | 104839813 | 104857237 | 0.5070 | 0.6650 | 0.8536 | 0.7671 | 0.8268 | 0.7281 | 0.3991 | 0.5823 |
| 0610012H03Rik | 105064477 | 105219953 | 0.0237 | 0.1487 | 0.1314 | 0.4595 | 0.1618 | 0.4642 | 0.1143 | 0.4528 |
| Dph4 | 105806866 | 105843706 | 0.2695 | 0.5123 | 0.4110 | 0.6536 | 0.0894 | 0.3908 | 0.5578 | 0.6234 |
| ***DMetS4a*** | | | | | | | | | | |
| OTTMUSG00000010671 | 146866526 | 146887595 | 0.7388 | 0.7375 | 0.2298 | 0.5634 | 0.6564 | 0.7111 | 0.0643 | 0.4109 |
| Gm13154 | 146927386 | 146959307 | 0.0151 | 0.1086 | 0.0341 | 0.3040 | 0.5902 | 0.6926 | 0.0978 | 0.4374 |
| 2610305D13Rik | 146986046 | 147016622 | 0.0000 | **0.0000** | 0.4088 | 0.6536 | 0.1403 | 0.4525 | 0.0194 | 0.3154 |
| Gm13143 | 147197095 | 147222475 | 0.4522 | 0.6360 | 0.1028 | 0.4304 | 0.2545 | 0.5370 | 0.9159 | 0.6971 |
| Miip | 147234887 | 147242775 | 0.0006 | **0.0077** | 0.4651 | 0.6658 | 0.1553 | 0.4642 | 0.2266 | 0.5054 |
| Fv1 | 147243088 | 147244467 | 0.0300 | 0.1702 | 0.1413 | 0.4657 | 0.8802 | 0.7446 | 0.1632 | 0.4732 |
| Mfn2 | 147247708 | 147278813 | 0.2699 | 0.5123 | 0.4296 | 0.6555 | 0.1419 | 0.4548 | 0.6502 | 0.6456 |
| Plod1 | 147283862 | 147310876 | 0.3210 | 0.5496 | 0.0665 | 0.3775 | 0.3164 | 0.5865 | 0.3267 | 0.5550 |
| 2510039O18Rik | 147315004 | 147321423 | 0.5380 | 0.6773 | 0.3575 | 0.6317 | 0.8688 | 0.7422 | 0.4218 | 0.5874 |
| Nppb | 147359897 | 147361314 | 0.8949 | 0.7733 | 0.7281 | 0.7376 | 0.9482 | 0.7586 | 0.9189 | 0.6971 |
| Nppa | 147374831 | 147376188 | 0.2884 | 0.5253 | 0.1713 | 0.5008 | 0.6315 | 0.7011 | 0.1035 | 0.4467 |
| Clcn6 | 147378368 | 147412930 | 0.1871 | 0.4320 | 0.2479 | 0.5790 | 0.0454 | 0.3119 | 0.0202 | 0.3158 |
| Agtrap | 147451170 | 147462140 | 0.0000 | **0.0000** | 0.0659 | 0.3757 | 0.3313 | 0.6000 | 0.0094 | 0.2769 |
| 2610109H07Rik | 147472546 | 147504807 | 0.0005 | **0.0067** | 0.0642 | 0.3722 | 0.9986 | 0.7661 | 0.6399 | 0.6456 |
| 2610109H07Rik | 147472546 | 147504807 | 0.2238 | 0.4664 | 0.7393 | 0.7413 | 0.6793 | 0.7111 | 0.0244 | 0.3351 |
| Mad2l2 | 147504493 | 147519808 | 0.1961 | 0.4424 | 0.6497 | 0.7157 | 0.2309 | 0.5227 | 0.5168 | 0.6191 |
| Fbxo6 | 147519825 | 147526249 | 0.0000 | **0.0000** | 0.7751 | 0.7424 | 0.1647 | 0.4642 | 0.8065 | 0.6717 |
| Fbxo44 | 147526912 | 147534203 | 0.8168 | 0.7620 | 0.9998 | 0.7951 | 0.0022 | 0.0947 | 0.0185 | 0.3084 |
| Fbxo2 | 147534730 | 147540533 | 0.0013 | **0.0155** | 0.5858 | 0.7055 | 0.6989 | 0.7111 | 0.0609 | 0.4105 |
| Ptchd2 | 147614373 | 147662074 | 0.7419 | 0.7375 | 0.4361 | 0.6581 | 0.1007 | 0.4095 | 0.3688 | 0.5689 |
| Ubiad1 | 147808604 | 147818880 | 0.8777 | 0.7719 | 0.0604 | 0.3706 | 0.3314 | 0.6000 | 0.9214 | 0.6971 |
| Frap1 | 147822720 | 147931792 | 0.0398 | 0.1998 | 0.9244 | 0.7816 | 0.0623 | 0.3543 | 0.4671 | 0.6016 |
| Angptl7 | 147869292 | 147874569 | 0.0028 | **0.0283** | 0.0005 | **0.0451** | 0.0003 | **0.0473** | 0.0922 | 0.4290 |
| Exosc10 | 147932538 | 147956510 | 0.0626 | 0.2587 | 0.6383 | 0.7152 | 0.7025 | 0.7111 | 0.7184 | 0.6581 |
| Srm | 147965612 | 147969102 | 0.6530 | 0.7229 | 0.4706 | 0.6689 | 0.1175 | 0.4217 | 0.7248 | 0.6592 |
| Masp2 | 147976663 | 147989608 | 0.0001 | **0.0024** | 0.0244 | 0.2667 | 0.4415 | 0.6467 | 0.8383 | 0.6814 |
| Casz1 | 148178538 | 148328998 | 0.2567 | 0.5013 | 0.7496 | 0.7420 | 0.0091 | 0.1725 | 0.0838 | 0.4270 |
| Pex14 | 148334644 | 148473985 | 0.5872 | 0.7019 | 0.0930 | 0.4270 | 0.2940 | 0.5690 | 0.6293 | 0.6456 |
| Dffa | 148478255 | 148494756 | 0.2147 | 0.4591 | 0.7886 | 0.7469 | 0.9434 | 0.7577 | 0.7317 | 0.6611 |
| Cort | 148499143 | 148500872 | 0.0094 | 0.0770 | 0.9736 | 0.7902 | 0.8001 | 0.7235 | 0.0276 | 0.3411 |
| Apitd1 | 148501230 | 148511738 | 0.0009 | **0.0106** | 0.6730 | 0.7276 | 0.0251 | 0.2610 | 0.1930 | 0.4979 |
| Pgd | 148524100 | 148540880 | 0.8236 | 0.7628 | 0.6846 | 0.7318 | 0.1605 | 0.4642 | 0.1120 | 0.4500 |
| Kif1b | 148550428 | 148681802 | 0.0610 | 0.2545 | 0.5277 | 0.6794 | 0.4064 | 0.6326 | 0.5126 | 0.6170 |
| Ube4b | 148702525 | 148800858 | 0.2480 | 0.4970 | 0.7121 | 0.7374 | 0.3786 | 0.6193 | 0.9453 | 0.7037 |
| Rbp7 | 148823796 | 148829087 | 0.0000 | **0.0000** | 0.3688 | 0.6433 | 0.9960 | 0.7661 | 0.5875 | 0.6345 |
| Nmnat1 | 148841681 | 148859311 | 0.1032 | 0.3363 | 0.0006 | **0.0451** | 0.0010 | 0.0724 | 0.0089 | 0.2769 |
| Lzic | 148859338 | 148870777 | 0.4722 | 0.6449 | 0.5119 | 0.6774 | 0.1506 | 0.4642 | 0.6920 | 0.6523 |
| Ctnnbip1 | 148892345 | 148940546 | 0.2497 | 0.4974 | 0.1163 | 0.4524 | 0.8399 | 0.7350 | 0.6623 | 0.6477 |
| Clstn1 | 148960577 | 149023008 | 0.0381 | 0.1959 | 0.7166 | 0.7374 | 0.0813 | 0.3827 | 0.0515 | 0.4020 |
| Pik3cd | 149023277 | 149076680 | 0.0040 | **0.0377** | 0.0094 | 0.2228 | 0.6721 | 0.7111 | 0.0744 | 0.4190 |
| Tmem201 | 149089484 | 149112153 | 0.0018 | **0.0205** | 0.0758 | 0.4001 | 0.1854 | 0.4798 | 0.5014 | 0.6114 |
| Slc25a33 | 149118145 | 149148386 | 0.8716 | 0.7719 | 0.0058 | 0.1900 | 0.6615 | 0.7111 | 0.5715 | 0.6298 |
| Spsb1 | 149270392 | 149329152 | 0.1638 | 0.4023 | 0.0410 | 0.3166 | 0.9476 | 0.7586 | 0.3422 | 0.5586 |
| H6pd | 149353584 | 149383132 | 0.1520 | 0.3949 | 0.8330 | 0.7600 | 0.8120 | 0.7252 | 0.6479 | 0.6456 |
| Eno1 | 149610830 | 149622988 | 0.7094 | 0.7375 | 0.0358 | 0.3081 | 0.0008 | 0.0724 | 0.0020 | 0.2041 |
| Errfi1 | 150228028 | 150243001 | 0.0023 | **0.0244** | 0.1463 | 0.4724 | 0.0394 | 0.3081 | 0.2743 | 0.5179 |
| Park7 | 150271242 | 150288546 | 0.3389 | 0.5678 | 0.9886 | 0.7949 | 0.7255 | 0.7146 | 0.4492 | 0.5968 |
| Tnfrsf9 | 150288671 | 150320211 | 0.4327 | 0.6307 | 0.2868 | 0.5994 | 0.6061 | 0.6943 | 0.0897 | 0.4290 |
| Per3 | 150377761 | 150418774 | 0.8872 | 0.7719 | 0.5711 | 0.6970 | 0.9989 | 0.7661 | 0.7639 | 0.6634 |
| Vamp3 | 150421409 | 150432072 | 0.2211 | 0.4662 | 0.3196 | 0.6077 | 0.9245 | 0.7505 | 0.2694 | 0.5179 |
| Camta1 | 150433634 | 151235985 | 0.6766 | 0.7290 | 0.8170 | 0.7550 | 0.6966 | 0.7111 | 0.7546 | 0.6611 |
| Dnajc11 | 151307800 | 151356246 | 0.4083 | 0.6144 | 0.4866 | 0.6732 | 0.2569 | 0.5370 | 0.7013 | 0.6531 |
| 2210418H06Rik | 151356748 | 151363106 | 0.0048 | **0.0444** | 0.3065 | 0.6045 | 0.0090 | 0.1725 | 0.5505 | 0.6227 |
| Phf13 | 151363742 | 151370367 | 0.7188 | 0.7375 | 0.2173 | 0.5548 | 0.0589 | 0.3515 | 0.6852 | 0.6512 |
| Klhl21 | 151382912 | 151391789 | 0.3575 | 0.5814 | 0.1867 | 0.5131 | 0.7417 | 0.7157 | 0.7490 | 0.6611 |
| Tas1r1 | 151402023 | 151412677 | 0.6455 | 0.7187 | 0.3927 | 0.6536 | 0.2530 | 0.5370 | 0.6478 | 0.6456 |
| Nol9 | 151413430 | 151435603 | 0.2508 | 0.4981 | 0.4379 | 0.6585 | 0.8901 | 0.7468 | 0.5753 | 0.6298 |
| Plekhg5 | 151446607 | 151489509 | 0.7457 | 0.7385 | 0.0793 | 0.4040 | 0.0387 | 0.3075 | 0.3366 | 0.5586 |
| Tnfrsf25 | 151490043 | 151494228 | 0.8997 | 0.7733 | 0.0316 | 0.2945 | 0.1351 | 0.4469 | 0.1265 | 0.4703 |
| Espn | 151494440 | 151526480 | 0.1265 | 0.3692 | 0.4152 | 0.6536 | 0.0340 | 0.2943 | 0.0406 | 0.3837 |
| Acot7 | 151552243 | 151645961 | 0.0230 | 0.1459 | 0.7785 | 0.7424 | 0.4408 | 0.6467 | 0.4217 | 0.5874 |
| Gpr153 | 151648341 | 151659446 | 0.4827 | 0.6478 | 0.0049 | 0.1732 | 0.3867 | 0.6250 | 0.7426 | 0.6611 |
| 1700008E11Rik | 151671336 | 151681230 | 0.2984 | 0.5316 | 0.6919 | 0.7320 | 0.7163 | 0.7128 | 0.6208 | 0.6438 |
| Rnf207 | 151681128 | 151693083 | 0.3083 | 0.5400 | 0.0532 | 0.3601 | 0.2723 | 0.5513 | 0.6017 | 0.6413 |
| Rpl22 | 151699851 | 151708180 | 0.0247 | 0.1518 | 0.2128 | 0.5490 | 0.9098 | 0.7505 | 0.2723 | 0.5179 |
| Chd5 | 151712996 | 151764290 | 0.0035 | **0.0340** | 0.0335 | 0.3040 | 0.6903 | 0.7111 | 0.1385 | 0.4703 |
| Kcnab2 | 151764851 | 151851980 | 0.0289 | 0.1689 | 0.0157 | 0.2352 | 0.8678 | 0.7422 | 0.3436 | 0.5586 |
| Nphp4 | 151850815 | 151937158 | 0.6559 | 0.7238 | 0.0714 | 0.3908 | 0.5039 | 0.6651 | 0.8813 | 0.6908 |
| Ajap1 | 152747330 | 152856939 | 0.5150 | 0.6676 | 0.3018 | 0.6021 | 0.6558 | 0.7111 | 0.3364 | 0.5586 |
| ***DMetS6a*** | | | | | | | | | | |
| Cntnap2 | 45010060 | 47251368 | 0.2078 | 0.4539 | 0.8316 | 0.7598 | 0.7796 | 0.7211 | 0.4539 | 0.5968 |
| Cul1 | 47403397 | 47476138 | 0.0377 | 0.1950 | 0.5664 | 0.6948 | 0.0607 | 0.3519 | 0.0391 | 0.3810 |
| A930035D04Rik | 47403775 | 47404319 | 0.6600 | 0.7242 | 0.3062 | 0.6045 | 0.2791 | 0.5573 | 0.5883 | 0.6345 |
| Ezh2 | 47480273 | 47545340 | 0.8771 | 0.7719 | 0.7538 | 0.7420 | 0.9150 | 0.7505 | 0.3152 | 0.5466 |
| Pdia4 | 47746141 | 47763355 | 0.1710 | 0.4116 | 0.4471 | 0.6611 | 0.9181 | 0.7505 | 0.0113 | 0.2975 |
| Zfp786 | 47769265 | 47780866 | 0.2091 | 0.4552 | 0.4327 | 0.6555 | 0.6671 | 0.7111 | 0.0882 | 0.4290 |
| Zfp398 | 47785660 | 47818256 | 0.5219 | 0.6698 | 0.1005 | 0.4295 | 0.0594 | 0.3515 | 0.0070 | 0.2769 |
| Zfp282 | 47827203 | 47858484 | 0.0502 | 0.2290 | 0.7556 | 0.7420 | 0.0017 | 0.0792 | 0.7769 | 0.6642 |
| Zfp212 | 47870475 | 47882638 | 0.7485 | 0.7392 | 0.1576 | 0.4879 | 0.2635 | 0.5444 | 0.1719 | 0.4770 |
| AI894139 | 47903389 | 47915299 | 0.9435 | 0.7825 | 0.3345 | 0.6159 | 0.4505 | 0.6490 | 0.2984 | 0.5331 |
| Zfp777 | 47974204 | 47998910 | 0.6291 | 0.7153 | 0.9627 | 0.7881 | 0.0151 | 0.2251 | 0.5140 | 0.6181 |
| Zfp746 | 48012396 | 48036592 | 0.8197 | 0.7625 | 0.5210 | 0.6790 | 0.0737 | 0.3725 | 0.1823 | 0.4888 |
| Krba1 | 48345585 | 48369544 | 0.0852 | 0.3023 | 0.4469 | 0.6611 | 0.6222 | 0.6965 | 0.9936 | 0.7168 |
| Zfp467 | 48377696 | 48395824 | 0.0100 | 0.0809 | 0.8858 | 0.7702 | 0.8841 | 0.7446 | 0.5452 | 0.6220 |
| Atp6v0e2 | 48487700 | 48491793 | 0.1312 | 0.3765 | 0.0438 | 0.3274 | 0.2511 | 0.5365 | 0.5470 | 0.6226 |
| Lrrc61 | 48504795 | 48520721 | 0.2155 | 0.4591 | 0.3455 | 0.6237 | 0.0468 | 0.3184 | 0.5342 | 0.6215 |
| Rarres2 | 48519697 | 48522669 | 0.0025 | **0.0258** | 0.0789 | 0.4040 | 0.0013 | 0.0792 | 0.1888 | 0.4955 |
| Repin1 | 48543882 | 48549081 | 0.1935 | 0.4395 | 0.5251 | 0.6790 | 0.0051 | 0.1387 | 0.0014 | 0.2041 |
| Zfp775 | 48563179 | 48573226 | 0.0000 | **0.0000** | 0.1673 | 0.4965 | 0.6079 | 0.6943 | 0.3194 | 0.5507 |
| AI854703 | 48577934 | 48583688 | 0.2055 | 0.4539 | 0.2831 | 0.5994 | 0.1661 | 0.4642 | 0.0643 | 0.4109 |
| Gimap8 | 48597233 | 48610874 | 0.0372 | 0.1950 | 0.6037 | 0.7082 | 0.8771 | 0.7436 | 0.2152 | 0.5019 |
| Gimap9 | 48626128 | 48629113 | 0.0905 | 0.3102 | 0.5986 | 0.7072 | 0.1339 | 0.4464 | 0.3221 | 0.5519 |
| Gimap4 | 48634548 | 48642059 | 0.7116 | 0.7375 | 0.5018 | 0.6752 | 0.1681 | 0.4642 | 0.9384 | 0.7010 |
| Gimap6 | 48651581 | 48658224 | 0.0001 | **0.0012** | 0.4906 | 0.6732 | 0.2317 | 0.5236 | 0.9900 | 0.7156 |
| Gimap5 | 48668606 | 48704209 | 0.0005 | **0.0067** | 0.2054 | 0.5383 | 0.7550 | 0.7207 | 0.4287 | 0.5892 |
| Gimap7 | 48668620 | 48674635 | 0.3380 | 0.5676 | 0.4940 | 0.6732 | 0.8297 | 0.7288 | 0.3426 | 0.5586 |
| Gimap1 | 48689053 | 48693789 | 0.1810 | 0.4260 | 0.1356 | 0.4606 | 0.3241 | 0.5917 | 0.2169 | 0.5019 |
| Tmem176b | 48783828 | 48790977 | 0.1057 | 0.3386 | 0.0638 | 0.3722 | 0.0236 | 0.2551 | 0.2438 | 0.5113 |
| Tmem176a | 48791508 | 48795363 | 0.4415 | 0.6338 | 0.0000 | **0.0023** | 0.0328 | 0.2925 | 0.9944 | 0.7169 |
| Abp1 | 48845253 | 48859187 | 0.8804 | 0.7719 | 0.9075 | 0.7788 | 0.7957 | 0.7218 | 0.8703 | 0.6891 |
| Gpnmb | 48986612 | 49006778 | 0.0243 | 0.1514 | 0.0411 | 0.3166 | 0.0861 | 0.3900 | 0.1492 | 0.4703 |
| 2410003K15Rik | 49023794 | 49036750 | 0.1057 | 0.3386 | 0.1496 | 0.4771 | 0.7390 | 0.7153 | 0.4945 | 0.6114 |
| Igf2bp3 | 49035220 | 49164712 | 0.2572 | 0.5013 | 0.0479 | 0.3367 | 0.4051 | 0.6326 | 0.3623 | 0.5689 |
| Tra2a | 49193920 | 49214051 | 0.2060 | 0.4539 | 0.2935 | 0.6021 | 0.3998 | 0.6326 | 0.7633 | 0.6634 |
| Ccdc126 | 49269273 | 49291581 | 0.5095 | 0.6651 | 0.4242 | 0.6536 | 0.3557 | 0.6139 | 0.5261 | 0.6205 |
| D330028D13Rik | 49317738 | 49339904 | 0.6714 | 0.7283 | 0.5965 | 0.7072 | 0.5430 | 0.6784 | 0.8899 | 0.6916 |
| Npy | 49772709 | 49779506 | 0.8143 | 0.7620 | 0.4778 | 0.6693 | 0.4474 | 0.6490 | 0.8840 | 0.6908 |
| ***DMetS6b*** | | | | | | | | | | |
| Rpn1 | 88034514 | 88053950 | 0.2981 | 0.5316 | 0.0704 | 0.3871 | 0.6999 | 0.7111 | 0.2219 | 0.5051 |
| Gata2 | 88148328 | 88156483 | 0.6867 | 0.7311 | 0.6934 | 0.7320 | 0.7073 | 0.7116 | 0.8827 | 0.6908 |
| Dnajb8 | 88172262 | 88173251 | 0.1376 | 0.3812 | 0.0694 | 0.3862 | 0.5426 | 0.6784 | 0.3518 | 0.5678 |
| Eefsec | 88207865 | 88396507 | 0.7348 | 0.7375 | 0.1561 | 0.4856 | 0.0566 | 0.3453 | 0.8496 | 0.6854 |
| Ruvbl1 | 88415403 | 88447566 | 0.4723 | 0.6449 | 0.0450 | 0.3316 | 0.0880 | 0.3905 | 0.0464 | 0.3950 |
| Sec61a1 | 88453595 | 88468899 | 0.0031 | **0.0312** | 0.0312 | 0.2945 | 0.3351 | 0.6014 | 0.0244 | 0.3351 |
| Mgll | 88674406 | 88778354 | 0.3989 | 0.6068 | 0.4330 | 0.6555 | 0.8946 | 0.7474 | 0.3105 | 0.5430 |
| Abtb1 | 88785910 | 88791860 | 0.0839 | 0.3014 | 0.7808 | 0.7426 | 0.5640 | 0.6815 | 0.2285 | 0.5057 |
| Podxl2 | 88792552 | 88825038 | 0.0006 | **0.0075** | 0.4839 | 0.6723 | 0.1796 | 0.4765 | 0.2102 | 0.5019 |
| Mcm2 | 88833469 | 88848694 | 0.1734 | 0.4152 | 0.0384 | 0.3112 | 0.3778 | 0.6193 | 0.6187 | 0.6438 |
| Tpra40 | 88852245 | 88862232 | 0.3833 | 0.5957 | 0.1025 | 0.4304 | 0.1891 | 0.4824 | 0.0210 | 0.3163 |
| Chchd6 | 89333154 | 89545609 | 0.3632 | 0.5861 | 0.4536 | 0.6611 | 0.7875 | 0.7212 | 0.6945 | 0.6523 |
| Txnrd3 | 89593982 | 89625523 | 0.0837 | 0.3014 | 0.8478 | 0.7663 | 0.1272 | 0.4370 | 0.9992 | 0.7174 |
| V1rb9 | 89696476 | 89697408 | 0.8349 | 0.7637 | 0.3078 | 0.6046 | 0.2587 | 0.5390 | 0.6744 | 0.6505 |
| V1ra2 | 89881644 | 89890501 | 0.0400 | 0.1998 | 0.1561 | 0.4856 | 0.0420 | 0.3081 | 0.2137 | 0.5019 |
| BC048671 | 90251264 | 90255442 | 0.1504 | 0.3949 | 0.2935 | 0.6021 | 0.7870 | 0.7212 | 0.1124 | 0.4500 |
| Chst13 | 90259322 | 90275179 | 0.0016 | **0.0182** | 0.0752 | 0.3990 | 0.6012 | 0.6943 | 0.4870 | 0.6089 |
| Uroc1 | 90283300 | 90314545 | 0.0520 | 0.2327 | 0.1345 | 0.4606 | 0.0232 | 0.2551 | 0.3164 | 0.5466 |
| Zxdc | 90319486 | 90353484 | 0.0748 | 0.2848 | 0.9177 | 0.7804 | 0.4045 | 0.6326 | 0.0264 | 0.3411 |
| C230069K22Rik | 90353730 | 90378791 | 0.8643 | 0.7700 | 0.1302 | 0.4595 | 0.5460 | 0.6791 | 0.9047 | 0.6958 |
| Klf15 | 90412570 | 90425232 | 0.8826 | 0.7719 | 0.0760 | 0.4001 | 0.0057 | 0.1421 | 0.0555 | 0.4020 |
| Aldh1l1 | 90436421 | 90550197 | 0.1085 | 0.3464 | 0.8347 | 0.7601 | 0.7794 | 0.7211 | 0.7778 | 0.6642 |
| Slc41a3 | 90554719 | 90596406 | 0.0264 | 0.1596 | 0.8272 | 0.7575 | 0.8174 | 0.7260 | 0.0321 | 0.3488 |
| D6Ertd349e | 90609592 | 90714135 | 0.0392 | 0.1978 | 0.8594 | 0.7682 | 0.4784 | 0.6557 | 0.5408 | 0.6215 |
| EG665685 | 90855813 | 90856447 | 0.0001 | **0.0022** | 0.5249 | 0.6790 | 0.8327 | 0.7304 | 0.5387 | 0.6215 |
| Nup210 | 90963062 | 91066823 | 0.6341 | 0.7164 | 0.7618 | 0.7420 | 0.6038 | 0.6943 | 0.7243 | 0.6592 |
| Hdac11 | 91106659 | 91124686 | 0.7905 | 0.7554 | 0.6803 | 0.7313 | 0.8585 | 0.7397 | 0.7135 | 0.6577 |
| Fbln2 | 91162449 | 91222534 | 0.1551 | 0.3962 | 0.5011 | 0.6752 | 0.2417 | 0.5297 | 0.5525 | 0.6227 |
| Chchd4 | 91414271 | 91423417 | 0.0025 | **0.0264** | 0.0209 | 0.2529 | 0.0038 | 0.1327 | 0.1460 | 0.4703 |
| Tmem43 | 91423697 | 91438453 | 0.0362 | 0.1941 | 0.5861 | 0.7055 | 0.1585 | 0.4642 | 0.7180 | 0.6581 |
| Xpc | 91439299 | 91465878 | 0.0440 | 0.2134 | 0.0164 | 0.2352 | 0.0527 | 0.3396 | 0.1150 | 0.4528 |
| Lsm3 | 91465922 | 91472619 | 0.6020 | 0.7097 | 0.5227 | 0.6790 | 0.0152 | 0.2251 | 0.6573 | 0.6466 |
| Slc6a6 | 91634089 | 91709055 | 0.0491 | 0.2273 | 0.9987 | 0.7951 | 0.6402 | 0.7064 | 0.0483 | 0.4020 |
| ***DMetS6c*** | | | | | | | | | | |
| Il5ra | 106660378 | 106699031 | 0.6078 | 0.7123 | 0.9879 | 0.7949 | 0.7701 | 0.7211 | 0.1371 | 0.4703 |
| Crbn | 106728243 | 106750068 | 0.1463 | 0.3929 | 0.4784 | 0.6693 | 0.3846 | 0.6236 | 0.9731 | 0.7098 |
| Lrrn1 | 107479777 | 107520204 | 0.4261 | 0.6271 | 0.3878 | 0.6532 | 0.5693 | 0.6832 | 0.4136 | 0.5860 |
| Setmar | 108015039 | 108027116 | 0.9459 | 0.7825 | 0.9001 | 0.7752 | 0.1951 | 0.4879 | 0.2408 | 0.5100 |
| Itpr1 | 108163112 | 108501103 | 0.1665 | 0.4053 | 0.1116 | 0.4403 | 0.1838 | 0.4798 | 0.1748 | 0.4770 |
| Bhlhb2 | 108610623 | 108616919 | 0.2501 | 0.4976 | 0.6259 | 0.7135 | 0.1152 | 0.4200 | 0.3409 | 0.5586 |
| Arl10c | 108733076 | 108773542 | 0.5308 | 0.6742 | 0.7258 | 0.7374 | 0.5625 | 0.6815 | 0.4103 | 0.5860 |
| Edem1 | 108778635 | 108809350 | 0.2073 | 0.4539 | 0.8531 | 0.7671 | 0.2381 | 0.5293 | 0.7575 | 0.6611 |
| ***DMetS7a*** | | | | | | | | | | |
| Atp10a | 65913702 | 66084161 | 0.8499 | 0.7674 | 0.0037 | 0.1495 | 0.7660 | 0.7211 | 0.1955 | 0.4989 |
| Ube3a | 66484122 | 66562097 | 0.4594 | 0.6407 | 0.3226 | 0.6077 | 0.0073 | 0.1666 | 0.5052 | 0.6141 |
| Snurf | 67133488 | 67144657 | 0.0004 | **0.0064** | 0.0462 | 0.3345 | 0.0186 | 0.2478 | 0.7542 | 0.6611 |
| EG664849 | 67300315 | 67301328 | 0.4687 | 0.6449 | 0.4794 | 0.6693 | 0.7950 | 0.7218 | 0.0077 | 0.2769 |
| B230209E15Rik | 68673671 | 68760213 | 0.4617 | 0.6407 | 0.2938 | 0.6021 | 0.0725 | 0.3725 | 0.3607 | 0.5689 |
| Ndn | 69493163 | 69494814 | 0.0004 | **0.0056** | 0.6341 | 0.7152 | 0.1615 | 0.4642 | 0.5255 | 0.6205 |
| Magel2 | 69524573 | 69526522 | 0.2347 | 0.4825 | 0.6633 | 0.7197 | 0.1868 | 0.4798 | 0.9664 | 0.7095 |
| Mkrn3 | 69563293 | 69564927 | 0.0117 | 0.0924 | 0.7521 | 0.7420 | 0.1555 | 0.4642 | 0.8066 | 0.6717 |
| Chrna7 | 70243578 | 70357399 | 0.1839 | 0.4284 | 0.1017 | 0.4304 | 0.0497 | 0.3266 | 0.0891 | 0.4290 |
| Klf13 | 71036253 | 71083743 | 0.1439 | 0.3912 | 0.2604 | 0.5922 | 0.0288 | 0.2777 | 0.2178 | 0.5019 |
| Trpm1 | 71298731 | 71414643 | 0.0915 | 0.3119 | 0.0274 | 0.2842 | 0.3918 | 0.6277 | 0.7119 | 0.6576 |
| BB128963 | 71432556 | 71485692 | 0.0496 | 0.2278 | 0.1392 | 0.4657 | 0.7500 | 0.7195 | 0.8430 | 0.6831 |
| 6030441H18Rik | 71506378 | 71518981 | 0.5890 | 0.7019 | 0.8234 | 0.7575 | 0.8655 | 0.7422 | 0.5790 | 0.6311 |
| 2810453H10Rik | 71521462 | 71537154 | 0.3734 | 0.5914 | 0.0419 | 0.3166 | 0.2608 | 0.5413 | 0.8564 | 0.6859 |
| Mcee | 71537531 | 71557007 | 0.1238 | 0.3673 | 0.1235 | 0.4579 | 0.4599 | 0.6513 | 0.2477 | 0.5114 |
| Apba2 | 71646592 | 71898756 | 0.0025 | **0.0258** | 0.0195 | 0.2521 | 0.0610 | 0.3519 | 0.0338 | 0.3576 |
| 5730507A09Rik | 71900999 | 72301456 | 0.0555 | 0.2402 | 0.4230 | 0.6536 | 0.1677 | 0.4642 | 0.0546 | 0.4020 |
| Tjp1 | 72441051 | 72516125 | 0.1478 | 0.3937 | 0.2763 | 0.5994 | 0.3538 | 0.6139 | 0.1639 | 0.4733 |
| LOC381892 | 72657935 | 72672667 | 0.4745 | 0.6468 | 0.6176 | 0.7112 | 0.4501 | 0.6490 | 0.7982 | 0.6696 |
| Tarsl2 | 72789784 | 72836977 | 0.0164 | 0.1143 | 0.0096 | 0.2229 | 0.0014 | 0.0792 | 0.1985 | 0.5003 |
| ***DMetS7b*** | | | | | | | | | | |
| Rgma | 80520406 | 80564785 | 0.2126 | 0.4591 | 0.9642 | 0.7886 | 0.5074 | 0.6657 | 0.5492 | 0.6227 |
| Chd2 | 80574494 | 80686716 | 0.0133 | 0.1011 | 0.4396 | 0.6587 | 0.5089 | 0.6657 | 0.5329 | 0.6215 |
| 1810026B05Rik | 80700038 | 80703279 | 0.3995 | 0.6069 | 0.9521 | 0.7880 | 0.0885 | 0.3905 | 0.8189 | 0.6761 |
| ***DMetS7c*** | | | | | | | | | | |
| Prc1 | 87439403 | 87461145 | 0.0001 | **0.0011** | 0.0093 | 0.2228 | 0.8678 | 0.7422 | 0.0053 | 0.2733 |
| Rccd1 | 87461482 | 87469340 | 0.0000 | **0.0006** | 0.3236 | 0.6077 | 0.4669 | 0.6534 | 0.1889 | 0.4955 |
| Unc45a | 87470178 | 87485891 | 0.0000 | **0.0000** | 0.4751 | 0.6693 | 0.4370 | 0.6467 | 0.4062 | 0.5847 |
| 1700052O22Rik | 87505336 | 87516012 | 0.0000 | **0.0000** | 0.0231 | 0.2667 | 0.7399 | 0.7153 | 0.0542 | 0.4020 |
| Fes | 87522641 | 87532781 | 0.0163 | 0.1143 | 0.0201 | 0.2521 | 0.9756 | 0.7641 | 0.3651 | 0.5689 |
| Furin | 87533471 | 87550322 | 0.0008 | **0.0103** | 0.2063 | 0.5388 | 0.0048 | 0.1371 | 0.1288 | 0.4703 |
| Blm | 87599977 | 87679996 | 0.6625 | 0.7242 | 0.6773 | 0.7304 | 0.5801 | 0.6904 | 0.5575 | 0.6234 |
| Crtc3 | 87731513 | 87833763 | 0.0049 | **0.0447** | 0.8137 | 0.7550 | 0.6860 | 0.7111 | 0.8007 | 0.6704 |
| Iqgap1 | 87857673 | 87948180 | 0.0153 | 0.1098 | 0.1741 | 0.5008 | 0.1846 | 0.4798 | 0.4533 | 0.5968 |
| Zscan2 | 88005806 | 88021402 | 0.6590 | 0.7242 | 0.1632 | 0.4940 | 0.3734 | 0.6193 | 0.5549 | 0.6232 |
| Wdr73 | 88035609 | 88046155 | 0.8639 | 0.7700 | 0.3818 | 0.6497 | 0.8267 | 0.7281 | 0.2662 | 0.5179 |
| Nmb | 88047114 | 88049962 | 0.6680 | 0.7266 | 0.0477 | 0.3367 | 0.7392 | 0.7153 | 0.5377 | 0.6215 |
| Sec11a | 88049775 | 88092666 | 0.7609 | 0.7420 | 0.1812 | 0.5092 | 0.0866 | 0.3900 | 0.2490 | 0.5114 |
| Zfp592 | 88138598 | 88190050 | 0.1239 | 0.3673 | 0.2746 | 0.5993 | 0.6141 | 0.6943 | 0.0327 | 0.3508 |
| Alpk3 | 88202486 | 88250498 | 0.9978 | 0.7909 | 0.8355 | 0.7603 | 0.1848 | 0.4798 | 0.4858 | 0.6089 |
| Slc28a1 | 88259685 | 88315302 | 0.5282 | 0.6727 | 0.2225 | 0.5587 | 0.1301 | 0.4413 | 0.8532 | 0.6859 |
| Cpeb1 | 88491912 | 88600351 | 0.1469 | 0.3932 | 0.0403 | 0.3166 | 0.0231 | 0.2551 | 0.7302 | 0.6611 |
| Ap3b2 | 88605285 | 88638811 | 0.3144 | 0.5458 | 0.4367 | 0.6582 | 0.4444 | 0.6467 | 0.8886 | 0.6914 |
| BC048679 | 88639161 | 88643167 | 0.9370 | 0.7819 | 0.2627 | 0.5938 | 0.2664 | 0.5458 | 0.9167 | 0.6971 |
| Fsd2 | 88679240 | 88711867 | 0.0000 | **0.0000** | 0.2772 | 0.5994 | 0.3524 | 0.6139 | 0.0271 | 0.3411 |
| Whdc1 | 88716164 | 88741374 | 0.0500 | 0.2287 | 0.5798 | 0.7020 | 0.6728 | 0.7111 | 0.7100 | 0.6568 |
| 9330120H11Rik | 88747139 | 88747525 | 0.2986 | 0.5316 | 0.5290 | 0.6798 | 0.0600 | 0.3519 | 0.6507 | 0.6456 |
| Homer2 | 88754601 | 88851695 | 0.1557 | 0.3962 | 0.3978 | 0.6536 | 0.8925 | 0.7468 | 0.4555 | 0.5968 |
| 2610204K14Rik | 88907811 | 88914377 | 0.3983 | 0.6068 | 0.9535 | 0.7880 | 0.6997 | 0.7111 | 0.9562 | 0.7069 |
| 3110040N11Rik | 88927068 | 88934364 | 0.0474 | 0.2226 | 0.7716 | 0.7424 | 0.2591 | 0.5390 | 0.2971 | 0.5331 |
| Btbd1 | 88938404 | 88974350 | 0.0145 | 0.1072 | 0.6293 | 0.7144 | 0.7026 | 0.7111 | 0.5888 | 0.6345 |
| Tm6sf1 | 89003887 | 89029320 | 0.2960 | 0.5316 | 0.0952 | 0.4272 | 0.8246 | 0.7277 | 0.1437 | 0.4703 |
| Bnc1 | 89111717 | 89137132 | 0.5110 | 0.6658 | 0.9133 | 0.7791 | 0.4845 | 0.6593 | 0.8653 | 0.6878 |
| Sh3gl3 | 89408482 | 89455929 | 0.6718 | 0.7283 | 0.6117 | 0.7095 | 0.2736 | 0.5523 | 0.4983 | 0.6114 |
| Adamtsl3 | 89722310 | 89762956 | 0.0129 | 0.0995 | 0.1320 | 0.4595 | 0.7301 | 0.7146 | 0.8926 | 0.6923 |
| 1700129I04Rik | 89781470 | 89797038 | 0.0408 | 0.2014 | 0.5007 | 0.6752 | 0.1868 | 0.4798 | 0.1641 | 0.4733 |
| Eftud1 | 89797124 | 89926362 | 0.0009 | **0.0106** | 0.4064 | 0.6536 | 0.0113 | 0.1988 | 0.0267 | 0.3411 |
| A530021J07Rik | 90296980 | 90327939 | 0.9971 | 0.7909 | 0.3994 | 0.6536 | 0.8029 | 0.7235 | 0.8837 | 0.6908 |
| Tmc3 | 90733441 | 90772219 | 0.6990 | 0.7335 | 0.8976 | 0.7743 | 0.9600 | 0.7614 | 0.6612 | 0.6476 |
| Stard5 | 90780469 | 90801637 | 0.6303 | 0.7153 | 0.2850 | 0.5994 | 0.9132 | 0.7505 | 0.1219 | 0.4631 |
| Il16 | 90791358 | 90894236 | 0.1260 | 0.3692 | 0.4941 | 0.6732 | 0.0943 | 0.4026 | 0.8387 | 0.6814 |
| 1700026D08Rik | 90922611 | 90943390 | 0.1916 | 0.4381 | 0.4886 | 0.6732 | 0.7685 | 0.7211 | 0.1562 | 0.4703 |
| Mesdc1 | 91028383 | 91032815 | 0.0000 | **0.0002** | 0.5469 | 0.6837 | 0.3337 | 0.6000 | 0.2725 | 0.5179 |
| 6330404C01Rik | 91081367 | 91235012 | 0.2076 | 0.4539 | 0.2705 | 0.5993 | 0.3011 | 0.5726 | 0.2241 | 0.5051 |
| 2210412D01Rik | 91257866 | 91300403 | 0.7570 | 0.7420 | 0.7053 | 0.7340 | 0.6988 | 0.7111 | 0.9401 | 0.7015 |
| Arnt2 | 91394788 | 91510178 | 0.2698 | 0.5123 | 0.1032 | 0.4304 | 0.8536 | 0.7387 | 0.0921 | 0.4290 |
| Fah | 91733669 | 91755232 | 0.0025 | **0.0258** | 0.3773 | 0.6489 | 0.1513 | 0.4642 | 0.6271 | 0.6456 |
| Zfand6 | 91763564 | 91827861 | 0.1288 | 0.3728 | 0.3795 | 0.6492 | 0.8326 | 0.7304 | 0.5937 | 0.6375 |
| ***DMetS8a*** | | | | | | | | | | |
| EG574083 | 22056169 | 22061750 | 0.8339 | 0.7637 | 0.5105 | 0.6774 | 0.5411 | 0.6784 | 0.8695 | 0.6891 |
| AY761185 | 22083372 | 22084427 | 0.6951 | 0.7329 | 0.7404 | 0.7415 | 0.7134 | 0.7128 | 0.6840 | 0.6512 |
| Defcr21 | 22165224 | 22166195 | 0.4147 | 0.6164 | 0.1690 | 0.4989 | 0.1857 | 0.4798 | 0.0511 | 0.4020 |
| Defcr-rs7 | 22331293 | 22332228 | 0.0331 | 0.1796 | 0.4298 | 0.6555 | 0.9184 | 0.7505 | 0.9277 | 0.6986 |
| Defcr3 | 22427088 | 22428056 | 0.0369 | 0.1950 | 0.6402 | 0.7152 | 0.3619 | 0.6172 | 0.4824 | 0.6084 |
| Defcr5 | 22437118 | 22438054 | 0.8413 | 0.7653 | 0.7048 | 0.7340 | 0.8644 | 0.7422 | 0.7476 | 0.6611 |
| Defcr-rs1 | 22465566 | 22466699 | 0.2621 | 0.5060 | 0.2761 | 0.5994 | 0.3184 | 0.5879 | 0.3699 | 0.5689 |
| Defcr20 | 22619730 | 22620709 | 0.7877 | 0.7533 | 0.5952 | 0.7072 | 0.5004 | 0.6625 | 0.6715 | 0.6502 |
| AY761184 | 22812991 | 22814117 | 0.0961 | 0.3195 | 0.2275 | 0.5634 | 0.1259 | 0.4370 | 0.2870 | 0.5274 |
| Defb1 | 22887071 | 22905657 | 0.9910 | 0.7909 | 0.1591 | 0.4895 | 0.1539 | 0.4642 | 0.9579 | 0.7069 |
| Defb2 | 22950398 | 22953954 | 0.7773 | 0.7503 | 0.4188 | 0.6536 | 0.4160 | 0.6356 | 0.7741 | 0.6642 |
| Defb10 | 22969373 | 22972483 | 0.2546 | 0.5007 | 0.5343 | 0.6800 | 0.5182 | 0.6712 | 0.2570 | 0.5135 |
| Defb9 | 22992185 | 22995893 | 0.0308 | 0.1715 | 0.0933 | 0.4270 | 0.0683 | 0.3619 | 0.2774 | 0.5207 |
| Defb11 | 23015848 | 23016884 | 0.9553 | 0.7839 | 0.2463 | 0.5772 | 0.2431 | 0.5305 | 0.9503 | 0.7058 |
| Defb15 | 23040271 | 23043182 | 0.0155 | 0.1098 | 0.0237 | 0.2667 | 0.0219 | 0.2551 | 0.0177 | 0.3041 |
| Defb35 | 23048824 | 23051350 | 0.0299 | 0.1702 | 0.1073 | 0.4362 | 0.0882 | 0.3905 | 0.0353 | 0.3635 |
| Defb13 | 23057234 | 23059322 | 0.0406 | 0.2014 | 0.0990 | 0.4276 | 0.0683 | 0.3619 | 0.0356 | 0.3635 |
| Ccdc70 | 23080247 | 23084513 | 0.6801 | 0.7301 | 0.4981 | 0.6752 | 0.5534 | 0.6815 | 0.1498 | 0.4703 |
| Atp7b | 23103257 | 23170777 | 0.9210 | 0.7801 | 0.8114 | 0.7550 | 0.1176 | 0.4217 | 0.9475 | 0.7040 |
| Alg11 | 23171193 | 23182099 | 0.2645 | 0.5076 | 0.5719 | 0.6970 | 0.8169 | 0.7260 | 0.2191 | 0.5019 |
| Nek3 | 23238755 | 23276907 | 0.0034 | **0.0331** | 0.6962 | 0.7320 | 0.9020 | 0.7505 | 0.4833 | 0.6084 |
| Ckap2 | 23278921 | 23296291 | 0.5885 | 0.7019 | 0.0098 | 0.2229 | 0.4521 | 0.6490 | 0.0128 | 0.3039 |
| Vps36 | 23303281 | 23331315 | 0.2805 | 0.5208 | 0.9695 | 0.7902 | 0.4621 | 0.6513 | 0.7941 | 0.6690 |
| Thsd1 | 23337785 | 23371806 | 0.0182 | 0.1222 | 0.0085 | 0.2224 | 0.7765 | 0.7211 | 0.9048 | 0.6958 |
| Tpte | 23393913 | 23481890 | 0.5884 | 0.7019 | 0.0025 | 0.1206 | 0.0206 | 0.2512 | 0.0206 | 0.3163 |
| Slc25a15 | 23486479 | 23509025 | 0.8537 | 0.7674 | 0.3543 | 0.6306 | 0.1184 | 0.4237 | 0.1709 | 0.4770 |
| Mrps31 | 23521913 | 23539945 | 0.0226 | 0.1444 | 0.0000 | **0.0049** | 0.0003 | **0.0473** | 0.0586 | 0.4056 |
| AI316807 | 23573095 | 23587140 | 0.1273 | 0.3692 | 0.2495 | 0.5797 | 0.0292 | 0.2778 | 0.8690 | 0.6891 |
| Slc20a2 | 23587260 | 23680079 | 0.9381 | 0.7819 | 0.7624 | 0.7420 | 0.1552 | 0.4642 | 0.1912 | 0.4960 |
| Vdac3 | 23687573 | 23704230 | 0.3157 | 0.5467 | 0.0217 | 0.2558 | 0.5256 | 0.6747 | 0.0585 | 0.4056 |
| Polb | 23738737 | 23763886 | 0.0176 | 0.1192 | 0.4053 | 0.6536 | 0.2284 | 0.5207 | 0.2497 | 0.5114 |
| Ikbkb | 23769684 | 23817061 | 0.9981 | 0.7909 | 0.0523 | 0.3559 | 0.0651 | 0.3564 | 0.5799 | 0.6311 |
| Plat | 23868240 | 23893316 | 0.2794 | 0.5208 | 0.0589 | 0.3706 | 0.0230 | 0.2551 | 0.1160 | 0.4528 |
| Ap3m2 | 23897827 | 23916099 | 0.5520 | 0.6839 | 0.6273 | 0.7140 | 0.4451 | 0.6471 | 0.9962 | 0.7174 |
| 1700041G16Rik | 23918747 | 23919204 | 0.0072 | 0.0629 | 0.0836 | 0.4116 | 0.0110 | 0.1959 | 0.0007 | 0.1692 |
| Myst3 | 23970007 | 24053731 | 0.4197 | 0.6217 | 0.7902 | 0.7475 | 0.2995 | 0.5713 | 0.1046 | 0.4486 |
| Ank1 | 24085294 | 24260969 | 0.8181 | 0.7620 | 0.9552 | 0.7881 | 0.9258 | 0.7507 | 0.7154 | 0.6579 |
| Agpat6 | 24284207 | 24318833 | 0.0742 | 0.2848 | 0.0004 | **0.0451** | 0.0008 | 0.0724 | 0.0029 | 0.2251 |
| Gins4 | 24337097 | 24348131 | 0.0246 | 0.1518 | 0.5986 | 0.7072 | 0.1033 | 0.4095 | 0.1607 | 0.4703 |
| Golga7 | 24351825 | 24367546 | 0.1048 | 0.3385 | 0.1939 | 0.5193 | 0.0006 | 0.0724 | 0.1571 | 0.4703 |
| Sfrp1 | 24521974 | 24560104 | 0.7791 | 0.7514 | 0.0610 | 0.3706 | 0.9087 | 0.7505 | 0.5484 | 0.6227 |
| Zmat4 | 24746491 | 25267057 | 0.0376 | 0.1950 | 0.8012 | 0.7522 | 0.6526 | 0.7111 | 0.0315 | 0.3488 |
| 1810011O10Rik | 25549048 | 25549368 | 0.7406 | 0.7375 | 0.5081 | 0.6766 | 0.9380 | 0.7555 | 0.5444 | 0.6220 |
| Indol1 | 25642366 | 25686805 | 0.2433 | 0.4927 | 0.6294 | 0.7144 | 0.5131 | 0.6691 | 0.5090 | 0.6162 |
| Indo | 25694608 | 25707481 | 0.1516 | 0.3949 | 0.6399 | 0.7152 | 0.8280 | 0.7286 | 0.0732 | 0.4190 |
| Adam18 | 25712718 | 25785227 | 0.0567 | 0.2436 | 0.0598 | 0.3706 | 0.0756 | 0.3725 | 0.1007 | 0.4413 |
| Adam3 | 25787706 | 25836297 | 0.1520 | 0.3949 | 0.6141 | 0.7095 | 0.7094 | 0.7116 | 0.1611 | 0.4703 |
| Adam5 | 25837565 | 25934841 | 0.1686 | 0.4075 | 0.6926 | 0.7320 | 0.8019 | 0.7235 | 0.1585 | 0.4703 |
| Adam32 | 25946615 | 26059276 | 0.1105 | 0.3501 | 0.5597 | 0.6920 | 0.6827 | 0.7111 | 0.0922 | 0.4290 |
| Adam9 | 26060097 | 26127266 | 0.0692 | 0.2734 | 0.9839 | 0.7941 | 0.5625 | 0.6815 | 0.3567 | 0.5689 |
| Tm2d2 | 26127752 | 26133731 | 0.7335 | 0.7375 | 0.8623 | 0.7682 | 0.3768 | 0.6193 | 0.3383 | 0.5586 |
| Htra4 | 26135400 | 26149434 | 0.0384 | 0.1965 | 0.0516 | 0.3559 | 0.0597 | 0.3517 | 0.2271 | 0.5054 |
| Plekha2 | 26149616 | 26212666 | 0.0307 | 0.1715 | 0.0316 | 0.2945 | 0.0379 | 0.3075 | 0.4582 | 0.5970 |
| Tacc1 | 26265024 | 26311921 | 0.5785 | 0.6978 | 0.4324 | 0.6555 | 0.0215 | 0.2551 | 0.6841 | 0.6512 |
| Fgfr1 | 26624126 | 26685253 | 0.0990 | 0.3271 | 0.0818 | 0.4070 | 0.9894 | 0.7655 | 0.8710 | 0.6891 |
| D030041N04Rik | 26702132 | 26707968 | 0.8819 | 0.7719 | 0.1935 | 0.5193 | 0.5594 | 0.6815 | 0.1313 | 0.4703 |
| Whsc1l1 | 26712073 | 26830139 | 0.6195 | 0.7141 | 0.3963 | 0.6536 | 0.0759 | 0.3725 | 0.0913 | 0.4290 |
| Ppapdc1b | 26830533 | 26835359 | 0.0865 | 0.3048 | 0.0184 | 0.2521 | 0.1287 | 0.4391 | 0.0068 | 0.2769 |
| Ddhd2 | 26835821 | 26864672 | 0.0008 | **0.0099** | 0.0004 | **0.0445** | 0.0002 | **0.0353** | 0.0018 | 0.2041 |
| Bag4 | 26877669 | 26895674 | 0.1377 | 0.3812 | 0.2270 | 0.5634 | 0.3808 | 0.6203 | 0.2791 | 0.5207 |
| Lsm1 | 26895780 | 26913003 | 0.5993 | 0.7084 | 0.4172 | 0.6536 | 0.3712 | 0.6193 | 0.1102 | 0.4500 |
| Star | 26918946 | 26925195 | 0.5242 | 0.6702 | 0.1035 | 0.4304 | 0.0454 | 0.3119 | 0.0250 | 0.3351 |
| Ash2l | 26926663 | 26958166 | 0.6221 | 0.7141 | 0.7383 | 0.7413 | 0.0150 | 0.2251 | 0.8761 | 0.6908 |
| Kcnu1 | 26960095 | 27048405 | 0.2367 | 0.4850 | 0.0146 | 0.2351 | 0.0404 | 0.3081 | 0.6158 | 0.6436 |
| Hgsnat | 27054931 | 27087216 | 0.3247 | 0.5531 | 0.1269 | 0.4595 | 0.0592 | 0.3515 | 0.5942 | 0.6375 |
| 4930444A02Rik | 27091076 | 27104593 | 0.6168 | 0.7141 | 0.0720 | 0.3909 | 0.1670 | 0.4642 | 0.1587 | 0.4703 |
| Fnta | 27109446 | 27126070 | 0.0095 | 0.0777 | 0.0116 | 0.2306 | 0.1326 | 0.4432 | 0.3507 | 0.5670 |
| Hook3 | 27131893 | 27229696 | 0.3299 | 0.5579 | 0.1975 | 0.5248 | 0.0993 | 0.4095 | 0.4008 | 0.5823 |
| Rnf170 | 27229840 | 27254343 | 0.0203 | 0.1339 | 0.5550 | 0.6882 | 0.0810 | 0.3827 | 0.7396 | 0.6611 |
| Thap1 | 27268684 | 27273412 | 0.2054 | 0.4539 | 0.0602 | 0.3706 | 0.0017 | 0.0792 | 0.8480 | 0.6854 |
| Zfp703 | 28087826 | 28091938 | 0.5048 | 0.6640 | 0.4212 | 0.6536 | 0.7921 | 0.7213 | 0.0554 | 0.4020 |
| Erlin2 | 28134331 | 28149896 | 0.0643 | 0.2616 | 0.8710 | 0.7682 | 0.4500 | 0.6490 | 0.5271 | 0.6206 |
| Prosc | 28153075 | 28165038 | 0.0000 | **0.0000** | 0.0703 | 0.3871 | 0.6254 | 0.6985 | 0.4465 | 0.5961 |
| Brf2 | 28234304 | 28239104 | 0.5622 | 0.6878 | 0.3035 | 0.6021 | 0.3160 | 0.5865 | 0.2473 | 0.5114 |
| 4833414G05Rik | 28249245 | 28285120 | 0.6113 | 0.7141 | 0.3744 | 0.6477 | 0.6005 | 0.6943 | 0.0270 | 0.3411 |
| Got1l1 | 28307932 | 28313019 | 0.0001 | **0.0011** | 0.5325 | 0.6800 | 0.0332 | 0.2925 | 0.0761 | 0.4214 |
| Adrb3 | 28336248 | 28340060 | 0.1571 | 0.3963 | 0.0960 | 0.4272 | 0.6009 | 0.6943 | 0.7710 | 0.6642 |
| Eif4ebp1 | 28370850 | 28385985 | 0.4184 | 0.6212 | 0.7305 | 0.7389 | 0.9947 | 0.7661 | 0.6817 | 0.6512 |
| 732482 | 28406517 | 28407672 | 0.0000 | **0.0001** | 0.0360 | 0.3081 | 0.0017 | 0.0792 | 0.7879 | 0.6665 |
| Tex24 | 28454866 | 28459659 | 0.2402 | 0.4901 | 0.6356 | 0.7152 | 0.5303 | 0.6755 | 0.1322 | 0.4703 |
| Chrna6 | 28513686 | 28524356 | 0.1017 | 0.3325 | 0.2709 | 0.5993 | 0.4661 | 0.6534 | 0.1824 | 0.4888 |
| LOC382003 | 28714823 | 28730924 | 0.1230 | 0.3673 | 0.8417 | 0.7632 | 0.3502 | 0.6139 | 0.1880 | 0.4955 |
| ***DMetS8b*** | | | | | | | | | | |
| Rnf150 | 85387255 | 85608713 | 0.2662 | 0.5093 | 0.7223 | 0.7374 | 0.2263 | 0.5185 | 0.8486 | 0.6854 |
| Tbc1d9 | 85689251 | 85796833 | 0.0053 | **0.0473** | 0.6450 | 0.7152 | 0.6186 | 0.6955 | 0.7740 | 0.6642 |
| Ucp1 | 85814261 | 85821951 | 0.5649 | 0.6886 | 0.0011 | 0.0664 | 0.0008 | 0.0724 | 0.4024 | 0.5823 |
| Elmod2 | 85836921 | 85856385 | 0.4367 | 0.6325 | 0.6595 | 0.7186 | 0.7826 | 0.7212 | 0.0364 | 0.3684 |
| Clgn | 85913766 | 85952451 | 0.1514 | 0.3949 | 0.4246 | 0.6536 | 0.4620 | 0.6513 | 0.1622 | 0.4719 |
| Scoc | 85958392 | 85982290 | 0.0291 | 0.1690 | 0.9210 | 0.7816 | 0.9279 | 0.7510 | 0.0297 | 0.3488 |
| Ndufb7 | 86090570 | 86095525 | 0.0149 | 0.1079 | 0.0053 | 0.1754 | 0.2049 | 0.4962 | 0.7399 | 0.6611 |
| Gpsn2 | 86095599 | 86118361 | 0.0461 | 0.2197 | 0.0520 | 0.3559 | 0.7759 | 0.7211 | 0.0589 | 0.4056 |
| Dnajb1 | 86132172 | 86135801 | 0.5425 | 0.6795 | 0.1282 | 0.4595 | 0.0020 | 0.0898 | 0.1162 | 0.4528 |
| Gipc1 | 86176597 | 86188580 | 0.0002 | **0.0030** | 0.5673 | 0.6948 | 0.0565 | 0.3453 | 0.3585 | 0.5689 |
| Pkn1 | 86193661 | 86223078 | 0.0000 | **0.0000** | 0.0007 | 0.0522 | 0.0013 | 0.0792 | 0.0003 | 0.1004 |
| Cd97 | 86247150 | 86265225 | 0.0000 | **0.0000** | 0.1807 | 0.5088 | 0.0869 | 0.3900 | 0.5425 | 0.6215 |
| Lphn1 | 86424004 | 86465853 | 0.2665 | 0.5093 | 0.1424 | 0.4657 | 0.2371 | 0.5293 | 0.3577 | 0.5689 |
| Asf1b | 86479406 | 86494096 | 0.6957 | 0.7329 | 0.6468 | 0.7152 | 0.6431 | 0.7069 | 0.0303 | 0.3488 |
| Prkaca | 86496887 | 86520334 | 0.1206 | 0.3656 | 0.8167 | 0.7550 | 0.1968 | 0.4879 | 0.3577 | 0.5689 |
| Samd1 | 86521571 | 86524285 | 0.0554 | 0.2402 | 0.0064 | 0.1988 | 0.1092 | 0.4200 | 0.2479 | 0.5114 |
| 1700067K01Rik | 86525422 | 86528669 | 0.9969 | 0.7909 | 0.7496 | 0.7420 | 0.7743 | 0.7211 | 0.1269 | 0.4703 |
| 2210011C24Rik | 86534129 | 86535341 | 0.0592 | 0.2506 | 0.4149 | 0.6536 | 0.0400 | 0.3081 | 0.2743 | 0.5179 |
| 4432412L15Rik | 86545370 | 86554194 | 0.1853 | 0.4302 | 0.1549 | 0.4856 | 0.1981 | 0.4879 | 0.4869 | 0.6089 |
| Il27ra | 86554217 | 86566474 | 0.0574 | 0.2445 | 0.4562 | 0.6620 | 0.0549 | 0.3415 | 0.1309 | 0.4703 |
| Rln3 | 86566966 | 86568878 | 0.8005 | 0.7564 | 0.4909 | 0.6732 | 0.4617 | 0.6513 | 0.1083 | 0.4500 |
| C330011M18Rik | 86589135 | 86591186 | 0.0291 | 0.1690 | 0.7206 | 0.7374 | 0.1076 | 0.4200 | 0.6471 | 0.6456 |
| Rfx1 | 86590765 | 86620901 | 0.0094 | 0.0770 | 0.8403 | 0.7631 | 0.5630 | 0.6815 | 0.2363 | 0.5092 |
| BC057552 | 86620971 | 86628661 | 0.0933 | 0.3139 | 0.8918 | 0.7716 | 0.1635 | 0.4642 | 0.1557 | 0.4703 |
| Podnl1 | 86649888 | 86656426 | 0.0302 | 0.1702 | 0.7020 | 0.7340 | 0.1406 | 0.4525 | 0.1721 | 0.4770 |
| Cc2d1a | 86656727 | 86671796 | 0.0043 | **0.0401** | 0.5495 | 0.6856 | 0.8914 | 0.7468 | 0.6484 | 0.6456 |
| 4930432K21Rik | 86671924 | 86696489 | 0.1346 | 0.3812 | 0.0803 | 0.4040 | 0.1406 | 0.4525 | 0.2573 | 0.5135 |
| Nanos3 | 86697632 | 86700451 | 0.0172 | 0.1179 | 0.0187 | 0.2521 | 0.0062 | 0.1453 | 0.0208 | 0.3163 |
| Zswim4 | 86735845 | 86760954 | 0.9524 | 0.7838 | 0.1276 | 0.4595 | 0.1946 | 0.4879 | 0.1768 | 0.4779 |
| D8Ertd738e | 86770143 | 86773609 | 0.0928 | 0.3139 | 0.0124 | 0.2351 | 0.3138 | 0.5865 | 0.1193 | 0.4576 |
| Ccdc130 | 86781694 | 86794259 | 0.0012 | **0.0143** | 0.9965 | 0.7951 | 0.1684 | 0.4642 | 0.1002 | 0.4413 |
| Cacna1a | 86912339 | 87164145 | 0.0006 | **0.0077** | 0.7052 | 0.7340 | 0.2114 | 0.5056 | 0.2251 | 0.5051 |
| ***DMetS10a*** | | | | | | | | | | |
| Eea1 | 95403297 | 95508152 | 0.2965 | 0.5316 | 0.9355 | 0.7843 | 0.3751 | 0.6193 | 0.4662 | 0.6016 |
| Btg1 | 96079661 | 96082261 | 0.0228 | 0.1453 | 0.2342 | 0.5692 | 0.0195 | 0.2478 | 0.8795 | 0.6908 |
| Dcn | 96945001 | 96980785 | 0.0516 | 0.2327 | 0.9802 | 0.7920 | 0.0230 | 0.2551 | 0.2135 | 0.5019 |
| Lum | 97028464 | 97035338 | 0.0642 | 0.2616 | 0.0128 | 0.2351 | 0.0527 | 0.3396 | 0.1691 | 0.4770 |
| Atp2b1 | 98377804 | 98486420 | 0.3930 | 0.6047 | 0.0190 | 0.2521 | 0.3183 | 0.5879 | 0.1339 | 0.4703 |
| Wdr51b | 98569670 | 98660708 | 0.1212 | 0.3656 | 0.1887 | 0.5163 | 0.1560 | 0.4642 | 0.5841 | 0.6339 |
| Dusp6 | 98725865 | 98730118 | 0.0301 | 0.1702 | 0.2639 | 0.5940 | 0.8076 | 0.7246 | 0.3163 | 0.5466 |
| B530045E10Rik | 98883156 | 98885681 | 0.6551 | 0.7238 | 0.3439 | 0.6229 | 0.6030 | 0.6943 | 0.5264 | 0.6205 |
| Csl | 99220435 | 99221835 | 0.0486 | 0.2269 | 0.7526 | 0.7420 | 0.7923 | 0.7213 | 0.0973 | 0.4374 |
| Kitl | 99478264 | 99563047 | 0.1187 | 0.3646 | 0.9609 | 0.7881 | 0.2498 | 0.5365 | 0.5369 | 0.6215 |
| 4930430F08Rik | 100034969 | 100051876 | 0.1043 | 0.3385 | 0.9105 | 0.7791 | 0.3586 | 0.6150 | 0.1138 | 0.4528 |
| 1700017N19Rik | 100055020 | 100081025 | 0.1125 | 0.3533 | 0.2254 | 0.5619 | 0.6667 | 0.7111 | 0.0648 | 0.4109 |
| ***DMetS10b*** | | | | | | | | | | |
| Trhde | 113835879 | 114239363 | 0.3544 | 0.5814 | 0.2520 | 0.5815 | 0.6279 | 0.6998 | 0.9456 | 0.7037 |
| Tph2 | 114515697 | 114622078 | 0.5194 | 0.6685 | 0.1745 | 0.5008 | 0.5526 | 0.6815 | 0.0118 | 0.3005 |
| Tbc1d15 | 114635514 | 114688521 | 0.0035 | **0.0340** | 0.0344 | 0.3040 | 0.7768 | 0.7211 | 0.0861 | 0.4290 |
| Rab21 | 114726919 | 114752647 | 0.1352 | 0.3812 | 0.3388 | 0.6182 | 0.0017 | 0.0792 | 0.7506 | 0.6611 |
| Tmem19 | 114777795 | 114799318 | 0.1464 | 0.3929 | 0.1398 | 0.4657 | 0.2644 | 0.5445 | 0.1981 | 0.5003 |
| Zfc3h1 | 114822015 | 114869828 | 0.1226 | 0.3673 | 0.2113 | 0.5477 | 0.7955 | 0.7218 | 0.9745 | 0.7100 |
| Lgr5 | 114887367 | 115024836 | 0.7204 | 0.7375 | 0.1695 | 0.4989 | 0.2561 | 0.5370 | 0.7721 | 0.6642 |
| Tspan8 | 115254340 | 115286672 | 0.0636 | 0.2616 | 0.5459 | 0.6836 | 0.2309 | 0.5227 | 0.2683 | 0.5179 |
| Ptprr | 115455269 | 115711988 | 0.1648 | 0.4038 | 0.1089 | 0.4362 | 0.2666 | 0.5458 | 0.7488 | 0.6611 |
| 4933416C03Rik | 115549539 | 115550675 | 0.1537 | 0.3962 | 0.4612 | 0.6635 | 0.9191 | 0.7505 | 0.0624 | 0.4109 |
| Ptprb | 115738562 | 115820989 | 0.0000 | **0.0000** | 0.4435 | 0.6611 | 0.4226 | 0.6419 | 0.2695 | 0.5179 |
| Kcnmb4 | 115854924 | 115910579 | 0.1362 | 0.3812 | 0.2494 | 0.5797 | 0.1089 | 0.4200 | 0.9716 | 0.7098 |
| Cnot2 | 115922222 | 116018557 | 0.1581 | 0.3967 | 0.6965 | 0.7320 | 0.6416 | 0.7064 | 0.6449 | 0.6456 |
| 5330438D12Rik | 116018540 | 116019211 | 0.0000 | **0.0000** | 0.3405 | 0.6185 | 0.1200 | 0.4269 | 0.0502 | 0.4020 |
| Gm239 | 116213601 | 116333935 | 0.0007 | **0.0084** | 0.5040 | 0.6756 | 0.7889 | 0.7212 | 0.4112 | 0.5860 |
| Rab3ip | 116343833 | 116387487 | 0.6302 | 0.7153 | 0.5641 | 0.6945 | 0.5169 | 0.6710 | 0.0750 | 0.4190 |
| Lrrc10 | 116482397 | 116483824 | 0.7594 | 0.7420 | 0.8921 | 0.7716 | 0.0570 | 0.3453 | 0.0827 | 0.4262 |
| Cct2 | 116488059 | 116500834 | 0.6071 | 0.7120 | 0.1196 | 0.4543 | 0.5929 | 0.6934 | 0.6052 | 0.6413 |
| Frs2 | 116507185 | 116585530 | 0.1938 | 0.4395 | 0.0769 | 0.4030 | 0.9083 | 0.7505 | 0.0555 | 0.4020 |
| Yeats4 | 116652277 | 116661546 | 0.0000 | **0.0000** | 0.3320 | 0.6148 | 0.2366 | 0.5293 | 0.4827 | 0.6084 |
| Lyz2 | 116714389 | 116719320 | 0.3217 | 0.5501 | 0.0905 | 0.4264 | 0.0330 | 0.2925 | 0.2728 | 0.5179 |
| Lyz1 | 116724853 | 116729924 | 0.0000 | **0.0000** | 0.0259 | 0.2745 | 0.0138 | 0.2190 | 0.0181 | 0.3062 |
| Cpsf6 | 116792994 | 116814029 | 0.0000 | **0.0000** | 0.6417 | 0.7152 | 0.0433 | 0.3081 | 0.5606 | 0.6248 |
| ***DMetS14a*** | | | | | | | | | | |
| 5033413D16Rik | 115442101 | 115445949 | 0.4374 | 0.6325 | 0.2368 | 0.5701 | 0.3970 | 0.6322 | 0.6371 | 0.6456 |
| Gpc5 | 115491848 | 116923659 | 0.0129 | **0.0995** | 0.0111 | 0.2301 | 0.1374 | 0.4509 | 0.1350 | 0.4703 |
| ***DMetS15a*** | | | | | | | | | | |
| E130113K22Rik | 66409134 | 66476817 | 0.0738 | 0.2848 | 0.8832 | 0.7701 | 0.4289 | 0.6466 | 0.9034 | 0.6958 |
| Tg | 66502332 | 66682275 | 0.1493 | 0.3949 | 0.0390 | 0.3133 | 0.0538 | 0.3415 | 0.1882 | 0.4955 |
| Sla | 66612434 | 66644284 | 0.0188 | 0.1245 | 0.0016 | 0.0927 | 0.3353 | 0.6014 | 0.5380 | 0.6215 |
| Wisp1 | 66722882 | 66754763 | 0.2704 | 0.5125 | 0.0908 | 0.4264 | 0.9614 | 0.7614 | 0.0247 | 0.3351 |
| Ndrg1 | 66760880 | 66801203 | 0.0675 | 0.2703 | 0.1895 | 0.5167 | 0.2926 | 0.5678 | 0.2634 | 0.5166 |
| St3gal1 | 66934437 | 67008392 | 0.0000 | **0.0000** | 0.0000 | **0.0080** | 0.0055 | 0.1407 | 0.0000 | **0.0000** |
| Zfat | 67915328 | 68090418 | 0.9104 | 0.7764 | 0.1186 | 0.4543 | 0.3588 | 0.6150 | 0.3527 | 0.5678 |
| ***DMetS16a*** | | | | | | | | | | |
| Nfkbiz | 55811488 | 55839012 | 0.0116 | 0.0924 | 0.4148 | 0.6536 | 0.2968 | 0.5695 | 0.7749 | 0.6642 |
| Gm1752 | 55843318 | 55866498 | 0.1764 | 0.4210 | 0.0207 | 0.2529 | 0.6637 | 0.7111 | 0.1905 | 0.4960 |
| Lrriq2 | 55900001 | 55934968 | 0.0000 | **0.0005** | 0.3119 | 0.6077 | 0.0242 | 0.2580 | 0.0916 | 0.4290 |
| Rpl24 | 55966388 | 55980835 | 0.9315 | 0.7817 | 0.2979 | 0.6021 | 0.6037 | 0.6943 | 0.0649 | 0.4109 |
| 2310061J03Rik | 55973388 | 55974730 | 0.0080 | 0.0687 | 0.4230 | 0.6536 | 0.9243 | 0.7505 | 0.2635 | 0.5166 |
| Zbtb11 | 55989079 | 56006355 | 0.0142 | 0.1069 | 0.7257 | 0.7374 | 0.0424 | 0.3081 | 0.4771 | 0.6050 |
| Pcnp | 56007358 | 56029852 | 0.0000 | **0.0003** | 0.1319 | 0.4595 | 0.8147 | 0.7260 | 0.9533 | 0.7067 |
| Rg9mtd1 | 56032722 | 56037932 | 0.0000 | **0.0000** | 0.7755 | 0.7424 | 0.5652 | 0.6815 | 0.0044 | 0.2616 |
| Senp7 | 56075522 | 56190124 | 0.0000 | **0.0000** | 0.0157 | 0.2352 | 0.6365 | 0.7049 | 0.1539 | 0.4703 |
| Senp7 | 56075522 | 56190124 | 0.0000 | **0.0000** | 0.3185 | 0.6077 | 0.8430 | 0.7350 | 0.0100 | 0.2819 |
| Impg2 | 56204426 | 56273869 | 0.4987 | 0.6599 | 0.6196 | 0.7126 | 0.0700 | 0.3664 | 0.4116 | 0.5860 |
| Abi3bp | 56478013 | 56689430 | 0.0000 | **0.0000** | 0.1042 | 0.4311 | 0.6378 | 0.7054 | 0.5771 | 0.6298 |
| Tfg | 56690445 | 56717563 | 0.0406 | 0.2014 | 0.0114 | 0.2301 | 0.0329 | 0.2925 | 0.1292 | 0.4703 |
| Gpr128 | 56724722 | 56795968 | 0.9136 | 0.7773 | 0.1338 | 0.4606 | 0.5952 | 0.6934 | 0.0812 | 0.4262 |
| Tmem45a | 56805274 | 56886279 | 0.0000 | **0.0000** | 0.3019 | 0.6021 | 0.4839 | 0.6593 | 0.0087 | 0.2769 |
| 2310005G13Rik | 57038793 | 57049695 | 0.0000 | **0.0000** | 0.8091 | 0.7550 | 0.9933 | 0.7661 | 0.0819 | 0.4262 |
| Tomm70a | 57121827 | 57154643 | 0.8983 | 0.7733 | 0.1193 | 0.4543 | 0.2199 | 0.5129 | 0.8845 | 0.6908 |
| Nit2 | 57157007 | 57167428 | 0.3689 | 0.5894 | 0.2415 | 0.5772 | 0.4362 | 0.6467 | 0.2987 | 0.5331 |
| Tbc1d23 | 57168979 | 57231579 | 0.5410 | 0.6783 | 0.5197 | 0.6790 | 0.6835 | 0.7111 | 0.6559 | 0.6465 |
| Tmem30c | 57266252 | 57292978 | 0.8175 | 0.7620 | 0.7084 | 0.7362 | 0.6812 | 0.7111 | 0.5967 | 0.6392 |
| 2610528E23Rik | 57302113 | 57606956 | 0.0000 | **0.0000** | 0.8184 | 0.7553 | 0.6269 | 0.6995 | 0.0067 | 0.2769 |
| Col8a1 | 57624371 | 57754850 | 0.0001 | **0.0018** | 0.3237 | 0.6077 | 0.4084 | 0.6326 | 0.2236 | 0.5051 |
| Dcbld2 | 58408556 | 58469840 | 0.0000 | **0.0006** | 0.6931 | 0.7320 | 0.0037 | 0.1327 | 0.2976 | 0.5331 |
| St3gal6 | 58468238 | 58524356 | 0.0000 | **0.0000** | 0.2282 | 0.5634 | 0.0578 | 0.3485 | 0.6965 | 0.6523 |
| E330017A01Rik | 58635375 | 58638516 | 0.4693 | 0.6449 | 0.3012 | 0.6021 | 0.6028 | 0.6943 | 0.2652 | 0.5174 |
| Cpox | 58670405 | 58680474 | 0.4372 | 0.6325 | 0.5308 | 0.6800 | 0.8834 | 0.7446 | 0.1058 | 0.4490 |
| Cldnd1 | 58728023 | 58734364 | 0.0001 | **0.0019** | 0.6477 | 0.7154 | 0.3779 | 0.6193 | 0.7778 | 0.6642 |
| Olfr173 | 58796675 | 58797640 | 0.8674 | 0.7719 | 0.3017 | 0.6021 | 0.7094 | 0.7116 | 0.1939 | 0.4980 |
| Olfr192 | 59097892 | 59098816 | 0.9620 | 0.7851 | 0.0816 | 0.4070 | 0.1604 | 0.4642 | 0.0541 | 0.4020 |
| Olfr198 | 59201330 | 59202250 | 0.5924 | 0.7039 | 0.0979 | 0.4272 | 0.1455 | 0.4599 | 0.1757 | 0.4770 |
| ***DMetS17a*** | | | | | | | | | | |
| Gm5146 | 23688040 | 23690455 | 0.4936 | 0.6576 | 0.1186 | 0.4543 | 0.1065 | 0.4173 | 0.2868 | 0.5274 |
| Zfp213 | 23693737 | 23701096 | 0.0278 | 0.1659 | 0.1338 | 0.4606 | 0.2329 | 0.5241 | 0.6056 | 0.6413 |
| Zfp13 | 23712819 | 23736454 | 0.1154 | 0.3574 | 0.0372 | 0.3084 | 0.1959 | 0.4879 | 0.0318 | 0.3488 |
| Ccdc64b | 23797487 | 23805577 | 0.9896 | 0.7909 | 0.0928 | 0.4270 | 0.7678 | 0.7211 | 0.5551 | 0.6232 |
| Thoc6 | 23805581 | 23810849 | 0.7693 | 0.7452 | 0.9924 | 0.7951 | 0.2302 | 0.5227 | 0.8089 | 0.6727 |
| Hcfc1r1 | 23810924 | 23812192 | 0.0000 | **0.0000** | 0.0394 | 0.3133 | 0.0446 | 0.3119 | 0.0884 | 0.4290 |
| Tnfrsf12a | 23812414 | 23814400 | 0.0952 | 0.3178 | 0.1594 | 0.4895 | 0.2560 | 0.5370 | 0.0202 | 0.3158 |
| Cldn6 | 23816332 | 23819413 | 0.4758 | 0.6468 | 0.4534 | 0.6611 | 0.3641 | 0.6185 | 0.3987 | 0.5823 |
| Cldn9 | 23819551 | 23820985 | 0.0171 | 0.1179 | 0.0344 | 0.3040 | 0.0267 | 0.2753 | 0.1093 | 0.4500 |
| 1520401A03Rik | 23841455 | 23859750 | 0.0004 | **0.0056** | 0.4936 | 0.6732 | 0.0174 | 0.2403 | 0.0447 | 0.3950 |
| Pkmyt1 | 23869441 | 23873694 | 0.7100 | 0.7375 | 0.7801 | 0.7425 | 0.2145 | 0.5073 | 0.5756 | 0.6298 |
| Kremen2 | 23878166 | 23882796 | 0.0818 | 0.2997 | 0.1456 | 0.4710 | 0.0584 | 0.3501 | 0.8848 | 0.6908 |
| E030034P13Rik | 23886187 | 23888634 | 0.0000 | **0.0000** | 0.5121 | 0.6774 | 0.2231 | 0.5148 | 0.2153 | 0.5019 |
| Flywch2 | 23913883 | 23923048 | 0.0002 | **0.0029** | 0.2528 | 0.5825 | 0.2535 | 0.5370 | 0.3842 | 0.5780 |
| Srrm2 | 23940154 | 23961706 | 0.2988 | 0.5316 | 0.1043 | 0.4311 | 0.1770 | 0.4720 | 0.6551 | 0.6464 |
| Tceb2 | 23961707 | 23966065 | 0.2808 | 0.5208 | 0.9455 | 0.7880 | 0.2237 | 0.5153 | 0.8788 | 0.6908 |
| 4931440B09Rik | 23973752 | 23981139 | 0.1037 | 0.3374 | 0.4779 | 0.6693 | 0.1853 | 0.4798 | 0.1624 | 0.4719 |
| Prss21 | 24005023 | 24010081 | 0.2962 | 0.5316 | 0.3207 | 0.6077 | 0.1671 | 0.4642 | 0.0038 | 0.2616 |
| Dcpp1 | 24017843 | 24019820 | 0.9145 | 0.7775 | 0.8590 | 0.7682 | 0.7990 | 0.7235 | 0.9718 | 0.7098 |
| Dcpp2 | 24035689 | 24037754 | 0.8373 | 0.7638 | 0.4701 | 0.6689 | 0.7515 | 0.7202 | 0.2797 | 0.5210 |
| Tmprss8 | 24109093 | 24112197 | 0.6386 | 0.7180 | 0.8267 | 0.7575 | 0.7139 | 0.7128 | 0.2535 | 0.5124 |
| Prss27 | 24175111 | 24182921 | 0.3127 | 0.5435 | 0.6969 | 0.7320 | 0.7829 | 0.7212 | 0.6799 | 0.6512 |
| Kctd5 | 24184701 | 24210452 | 0.0910 | 0.3108 | 0.7795 | 0.7424 | 0.3299 | 0.5998 | 0.8174 | 0.6757 |
| Pdpk1 | 24210647 | 24287891 | 0.1418 | 0.3875 | 0.0052 | 0.1754 | 0.1980 | 0.4879 | 0.3891 | 0.5794 |
| Amdhd2 | 24292800 | 24300733 | 0.0810 | 0.2974 | 0.1621 | 0.4940 | 0.5915 | 0.6928 | 0.0428 | 0.3950 |
| Tbc1d24 | 24315731 | 24342460 | 0.4316 | 0.6307 | 0.5579 | 0.6909 | 0.4517 | 0.6490 | 0.4929 | 0.6114 |
| Ntn2l | 24340793 | 24346332 | 0.0752 | 0.2856 | 0.5403 | 0.6809 | 0.0187 | 0.2478 | 0.5413 | 0.6215 |
| Ccnf | 24360177 | 24388270 | 0.1594 | 0.3973 | 0.1625 | 0.4940 | 0.7594 | 0.7209 | 0.2617 | 0.5160 |
| Abca17 | 24401204 | 24487974 | 0.5391 | 0.6773 | 0.4320 | 0.6555 | 0.1976 | 0.4879 | 0.1940 | 0.4980 |
| Abca3 | 24488895 | 24547146 | 0.0524 | 0.2334 | 0.9562 | 0.7881 | 0.0869 | 0.3900 | 0.6509 | 0.6456 |
| Rnps1 | 24551620 | 24562840 | 0.0000 | **0.0000** | 0.9605 | 0.7881 | 0.6908 | 0.7111 | 0.3605 | 0.5689 |
| Dci | 24563639 | 24576261 | 0.8862 | 0.7719 | 0.0005 | **0.0451** | 0.0181 | 0.2468 | 0.2304 | 0.5057 |
| Dnase1l2 | 24577026 | 24580050 | 0.1638 | 0.4023 | 0.9022 | 0.7759 | 0.8152 | 0.7260 | 0.2791 | 0.5207 |
| E4f1 | 24580737 | 24592256 | 0.1952 | 0.4412 | 0.7378 | 0.7413 | 0.8179 | 0.7260 | 0.3987 | 0.5823 |
| Pgp | 24607418 | 24608536 | 0.1780 | 0.4225 | 0.1371 | 0.4632 | 0.6640 | 0.7111 | 0.9586 | 0.7069 |
| Gbl | 24610497 | 24615996 | 0.5799 | 0.6987 | 0.9101 | 0.7791 | 0.0248 | 0.2610 | 0.5697 | 0.6297 |
| 9930021D14Rik | 24610829 | 24612414 | 0.2046 | 0.4539 | 0.3295 | 0.6126 | 0.4429 | 0.6467 | 0.6471 | 0.6456 |
| Caskin1 | 24625728 | 24645850 | 0.1661 | 0.4053 | 0.6594 | 0.7186 | 0.4436 | 0.6467 | 0.9963 | 0.7174 |
| Traf7 | 24645795 | 24664775 | 0.3802 | 0.5957 | 0.1527 | 0.4805 | 0.5543 | 0.6815 | 0.7214 | 0.6585 |
| Rab26 | 24665999 | 24670833 | 0.3769 | 0.5951 | 0.1251 | 0.4595 | 0.0429 | 0.3081 | 0.6467 | 0.6456 |
| Pkd1 | 24686895 | 24733459 | 0.2544 | 0.5007 | 0.4765 | 0.6693 | 0.6978 | 0.7111 | 0.6931 | 0.6523 |
| Tsc2 | 24732882 | 24769574 | 0.3859 | 0.5976 | 0.6081 | 0.7095 | 0.0017 | 0.0792 | 0.7450 | 0.6611 |
| Nthl1 | 24769655 | 24775782 | 0.7321 | 0.7375 | 0.5026 | 0.6756 | 0.3533 | 0.6139 | 0.1524 | 0.4703 |
| Slc9a3r2 | 24776233 | 24787223 | 0.3370 | 0.5676 | 0.2804 | 0.5994 | 0.0656 | 0.3564 | 0.9845 | 0.7148 |
| Zfp598 | 24806697 | 24818960 | 0.6208 | 0.7141 | 0.6557 | 0.7186 | 0.3394 | 0.6043 | 0.9251 | 0.6983 |
| Syngr3 | 24822037 | 24826900 | 0.7001 | 0.7335 | 0.5824 | 0.7022 | 0.8470 | 0.7350 | 0.4135 | 0.5860 |
| Gfer | 24830136 | 24833018 | 0.9601 | 0.7844 | 0.0591 | 0.3706 | 0.0952 | 0.4047 | 0.4428 | 0.5955 |
| Noxo1 | 24833179 | 24837474 | 0.2075 | 0.4539 | 0.7252 | 0.7374 | 0.0424 | 0.3081 | 0.4023 | 0.5823 |
| Tbl3 | 24834894 | 24844605 | 0.1553 | 0.3962 | 0.5353 | 0.6800 | 0.1372 | 0.4509 | 0.6734 | 0.6502 |
| Rps2 | 24855061 | 24858874 | 0.0091 | 0.0759 | 0.1595 | 0.4895 | 0.8641 | 0.7422 | 0.4431 | 0.5955 |
| Ndufb10 | 24859005 | 24861423 | 0.7322 | 0.7375 | 0.0006 | **0.0451** | 0.1165 | 0.4203 | 0.5114 | 0.6168 |
| Rpl3l | 24869630 | 24873090 | 0.5661 | 0.6894 | 0.0968 | 0.4272 | 0.5044 | 0.6652 | 0.4128 | 0.5860 |
| Sepx1 | 24873587 | 24879723 | 0.0560 | 0.2414 | 0.1927 | 0.5182 | 0.9213 | 0.7505 | 0.8839 | 0.6908 |
| Hs3st6 | 24889948 | 24895628 | 0.5266 | 0.6720 | 0.7744 | 0.7424 | 0.8164 | 0.7260 | 0.4052 | 0.5847 |
| 4930528F23Rik | 24966014 | 24976644 | 0.0000 | **0.0000** | 0.3214 | 0.6077 | 0.2331 | 0.5241 | 0.7286 | 0.6611 |
| Fahd1 | 24986363 | 24987046 | 0.0000 | **0.0001** | 0.0000 | **0.0023** | 0.0000 | **0.0028** | 0.0123 | 0.3005 |
| Hagh | 24987435 | 25001395 | 0.6625 | 0.7242 | 0.0211 | 0.2529 | 0.0763 | 0.3732 | 0.2968 | 0.5331 |
| Igfals | 25015715 | 25018953 | 0.6144 | 0.7141 | 0.0455 | 0.3330 | 0.1838 | 0.4798 | 0.0437 | 0.3950 |
| Nubp2 | 25019564 | 25023273 | 0.0000 | **0.0000** | 0.0234 | 0.2667 | 0.1454 | 0.4599 | 0.4605 | 0.5983 |
| Spsb3 | 25023588 | 25029097 | 0.0000 | **0.0000** | 0.0145 | 0.2351 | 0.0006 | 0.0724 | 0.4003 | 0.5823 |
| Eme2 | 25025431 | 25032032 | 0.0000 | **0.0000** | 0.6164 | 0.7112 | 0.0622 | 0.3543 | 0.3666 | 0.5689 |
| Mapk8ip3 | 25029098 | 25073913 | 0.0000 | **0.0000** | 0.0282 | 0.2874 | 0.9925 | 0.7661 | 0.5991 | 0.6406 |
| Mrps34 | 25032061 | 25034447 | 0.2228 | 0.4664 | 0.0185 | 0.2521 | 0.1525 | 0.4642 | 0.1899 | 0.4960 |
| Nme3 | 25033445 | 25034467 | 0.7247 | 0.7375 | 0.4847 | 0.6728 | 0.3716 | 0.6193 | 0.0876 | 0.4290 |
| Hn1l | 25079415 | 25097568 | 0.2479 | 0.4970 | 0.0041 | 0.1509 | 0.7122 | 0.7128 | 0.0058 | 0.2769 |
| 2810430B18Rik | 25079415 | 25097568 | 0.0156 | 0.1103 | 0.5730 | 0.6977 | 0.7177 | 0.7128 | 0.0164 | 0.3041 |
| Cramp1l | 25098248 | 25152171 | 0.9745 | 0.7872 | 0.1654 | 0.4940 | 0.7079 | 0.7116 | 0.4856 | 0.6089 |
| AI661311 | 25153036 | 25236440 | 0.0004 | **0.0058** | 0.5912 | 0.7066 | 0.9237 | 0.7505 | 0.7467 | 0.6611 |
| Tmem204 | 25194647 | 25218126 | 0.0252 | 0.1542 | 0.6215 | 0.7129 | 0.9442 | 0.7577 | 0.6224 | 0.6443 |
| Telo2 | 25236515 | 25252912 | 0.6123 | 0.7141 | 0.3574 | 0.6317 | 0.5848 | 0.6919 | 0.8366 | 0.6811 |
| Clcn7 | 25270336 | 25299049 | 0.0020 | **0.0221** | 0.3471 | 0.6251 | 0.0107 | 0.1939 | 0.5064 | 0.6143 |
| Gm317 | 25299787 | 25308796 | 0.3164 | 0.5471 | 0.8676 | 0.7682 | 0.9092 | 0.7505 | 0.5707 | 0.6298 |
| BC003965 | 25321520 | 25322450 | 0.0665 | 0.2679 | 0.0683 | 0.3844 | 0.0506 | 0.3305 | 0.8926 | 0.6923 |
| Gnptg | 25371262 | 25377061 | 0.1149 | 0.3568 | 0.2877 | 0.5994 | 0.6745 | 0.7111 | 0.9369 | 0.7010 |
| 0610007P22Rik | 25377149 | 25379742 | 0.0464 | 0.2205 | 0.3863 | 0.6526 | 0.0769 | 0.3734 | 0.0736 | 0.4190 |
| Baiap3 | 25379605 | 25389080 | 0.2402 | 0.4901 | 0.1804 | 0.5088 | 0.8120 | 0.7252 | 0.2734 | 0.5179 |
| Ube2i | 25397456 | 25410336 | 0.0001 | **0.0025** | 0.7327 | 0.7395 | 0.4948 | 0.6609 | 0.3623 | 0.5689 |
| Prss34 | 25435339 | 25437106 | 0.0514 | 0.2325 | 0.4983 | 0.6752 | 0.1978 | 0.4879 | 0.2515 | 0.5115 |
| Prss29 | 25457228 | 25459625 | 0.3566 | 0.5814 | 0.5775 | 0.7003 | 0.2863 | 0.5643 | 0.1112 | 0.4500 |
| Tpsab1 | 25480190 | 25482507 | 0.0000 | **0.0000** | 0.1476 | 0.4743 | 0.0540 | 0.3415 | 0.0560 | 0.4020 |
| Mcpt6 | 25503278 | 25505037 | 0.0000 | **0.0002** | 0.1126 | 0.4428 | 0.4055 | 0.6326 | 0.7669 | 0.6637 |
| Cacna1h | 25511230 | 25570728 | 0.6069 | 0.7120 | 0.5945 | 0.7072 | 0.6653 | 0.7111 | 0.8055 | 0.6717 |
| Sox8 | 25702838 | 25707631 | 0.6132 | 0.7141 | 0.5203 | 0.6790 | 0.5647 | 0.6815 | 0.9229 | 0.6971 |
| Lmf1 | 25716122 | 25799771 | 0.1932 | 0.4395 | 0.5054 | 0.6756 | 0.0359 | 0.3016 | 0.5700 | 0.6297 |
| Gng13 | 25854473 | 25856029 | 0.0849 | 0.3022 | 0.1643 | 0.4940 | 0.8573 | 0.7397 | 0.7027 | 0.6537 |
| Chtf18 | 25855976 | 25864347 | 0.7763 | 0.7499 | 0.0987 | 0.4275 | 0.3678 | 0.6193 | 0.1901 | 0.4960 |
| Rpusd1 | 25864696 | 25868401 | 0.6804 | 0.7301 | 0.3729 | 0.6476 | 0.1014 | 0.4095 | 0.5819 | 0.6326 |
| Mslnl | 25872985 | 25885275 | 0.0003 | **0.0053** | 0.9335 | 0.7833 | 0.1026 | 0.4095 | 0.2149 | 0.5019 |
| ***DMetS17b*** | | | | | | | | | | |
| 1300018I05Rik | 29686744 | 29842924 | 0.8854 | 0.7719 | 0.2355 | 0.5694 | 0.0411 | 0.3081 | 0.1544 | 0.4703 |
| Rnf8 | 29751735 | 29840304 | 0.5813 | 0.6992 | 0.2013 | 0.5338 | 0.1811 | 0.4771 | 0.7435 | 0.6611 |
| 1110021J02Rik | 29826323 | 29853957 | 0.0006 | **0.0079** | 0.0089 | 0.2228 | 0.1350 | 0.4469 | 0.0077 | 0.2769 |
| Mdga1 | 29964903 | 30024827 | 0.4617 | 0.6407 | 0.7521 | 0.7420 | 0.0363 | 0.3016 | 0.0549 | 0.4020 |
| Zfand3 | 30142032 | 30346964 | 0.0874 | 0.3062 | 0.9994 | 0.7951 | 0.7160 | 0.7128 | 0.4460 | 0.5961 |
| Btbd9 | 30357046 | 30667310 | 0.3843 | 0.5965 | 0.0544 | 0.3667 | 0.0707 | 0.3675 | 0.2546 | 0.5135 |
| Glo1 | 30729806 | 30749539 | 0.6857 | 0.7311 | 0.8662 | 0.7682 | 0.5665 | 0.6815 | 0.4331 | 0.5892 |
| Dnahc8 | 30763936 | 31012209 | 0.1563 | 0.3962 | 0.9533 | 0.7880 | 0.7276 | 0.7146 | 0.1393 | 0.4703 |
| Abcg1 | 31194643 | 31252722 | 0.1545 | 0.3962 | 0.0464 | 0.3345 | 0.5675 | 0.6816 | 0.9584 | 0.7069 |
| Tff3 | 31262251 | 31266591 | 0.6652 | 0.7247 | 0.8785 | 0.7701 | 0.1362 | 0.4496 | 0.3072 | 0.5397 |
| Tff2 | 31277994 | 31281227 | 0.8226 | 0.7628 | 0.8137 | 0.7550 | 0.3140 | 0.5865 | 0.2120 | 0.5019 |
| Rsph1 | 31391969 | 31414252 | 0.3091 | 0.5407 | 0.9419 | 0.7879 | 0.9649 | 0.7614 | 0.5053 | 0.6141 |
| Slc37a1 | 31433702 | 31487569 | 0.0417 | 0.2038 | 0.6105 | 0.7095 | 0.9148 | 0.7505 | 0.0450 | 0.3950 |
| Wdr4 | 31632569 | 31649202 | 0.1301 | 0.3750 | 0.1064 | 0.4362 | 0.0089 | 0.1725 | 0.7649 | 0.6634 |
| 1500032D16Rik | 31657120 | 31668268 | 0.0003 | **0.0043** | 0.0000 | **0.0042** | 0.0000 | **0.0005** | 0.0019 | 0.2041 |
| 4833413E03Rik | 31694071 | 31696058 | 0.3549 | 0.5814 | 0.1643 | 0.4940 | 0.0078 | 0.1693 | 0.4678 | 0.6018 |
| Pknox1 | 31720641 | 31744629 | 0.1008 | 0.3318 | 0.1363 | 0.4618 | 0.3980 | 0.6326 | 0.1271 | 0.4703 |
| Cbs | 31749568 | 31774144 | 0.8255 | 0.7628 | 0.1432 | 0.4657 | 0.1220 | 0.4307 | 0.9902 | 0.7156 |
| U2af1 | 31784028 | 31795660 | 0.4786 | 0.6468 | 0.8991 | 0.7749 | 0.1080 | 0.4200 | 0.6537 | 0.6464 |
| Snf1lk | 31981193 | 31992737 | 0.0000 | **0.0000** | 0.3362 | 0.6159 | 0.1864 | 0.4798 | 0.3329 | 0.5585 |
| Rrp1b | 32173045 | 32199810 | 0.0001 | **0.0021** | 0.1514 | 0.4775 | 0.0093 | 0.1725 | 0.0658 | 0.4119 |
| Notch3 | 32257765 | 32303825 | 0.0001 | **0.0014** | 0.6340 | 0.7152 | 0.1140 | 0.4200 | 0.1126 | 0.4500 |
| Abhd9 | 32320715 | 32326494 | 0.1420 | 0.3875 | 0.0341 | 0.3040 | 0.1981 | 0.4879 | 0.3067 | 0.5397 |
| Brd4 | 32333219 | 32421667 | 0.0000 | **0.0006** | 0.9226 | 0.7816 | 0.1553 | 0.4642 | 0.5443 | 0.6220 |
| Akap8 | 32440621 | 32458098 | 0.4132 | 0.6159 | 0.8277 | 0.7575 | 0.0749 | 0.3725 | 0.7586 | 0.6611 |
| Akap8l | 32458370 | 32483746 | 0.4839 | 0.6478 | 0.8897 | 0.7716 | 0.0890 | 0.3905 | 0.2672 | 0.5179 |
| Wiz | 32491011 | 32526361 | 0.7395 | 0.7375 | 0.9385 | 0.7860 | 0.2191 | 0.5122 | 0.6550 | 0.6464 |
| A430107D22Rik | 32527604 | 32540528 | 0.1403 | 0.3846 | 0.0468 | 0.3345 | 0.4150 | 0.6356 | 0.2459 | 0.5113 |
| Cyp4f39 | 32589668 | 32630265 | 0.7342 | 0.7375 | 0.7375 | 0.7413 | 0.4888 | 0.6609 | 0.2050 | 0.5019 |
| Cyp4f16 | 32673574 | 32688742 | 0.0001 | **0.0010** | 0.2454 | 0.5772 | 0.7095 | 0.7116 | 0.1317 | 0.4703 |
| EG631304 | 32796431 | 32813425 | 0.5557 | 0.6864 | 0.0641 | 0.3722 | 0.0736 | 0.3725 | 0.5960 | 0.6390 |
| Cyp4f15 | 32822624 | 32840296 | 0.6632 | 0.7242 | 0.7046 | 0.7340 | 0.7218 | 0.7146 | 0.6695 | 0.6502 |
| 9030612M13Rik | 32910210 | 32924492 | 0.0000 | **0.0000** | 0.0793 | 0.4040 | 0.8915 | 0.7468 | 0.2611 | 0.5160 |
| Zfp811 | 32933958 | 32937883 | 0.4431 | 0.6345 | 0.4888 | 0.6732 | 0.4027 | 0.6326 | 0.6577 | 0.6466 |
| BC066107 | 33016173 | 33023028 | 0.0000 | **0.0000** | 0.3200 | 0.6077 | 0.3171 | 0.5871 | 0.2031 | 0.5019 |
| Cyp4f14 | 33042016 | 33054023 | 0.1543 | 0.3962 | 0.9451 | 0.7880 | 0.6663 | 0.7111 | 0.4425 | 0.5955 |
| Cyp4f13 | 33061633 | 33084347 | 0.4789 | 0.6468 | 0.4069 | 0.6536 | 0.0055 | 0.1407 | 0.9751 | 0.7100 |
| Zfp472 | 33102759 | 33116178 | 0.0177 | 0.1192 | 0.2914 | 0.6006 | 0.8030 | 0.7235 | 0.0249 | 0.3351 |
| 1700065O13Rik | 33153808 | 33170347 | 0.1507 | 0.3949 | 0.4478 | 0.6611 | 0.7141 | 0.7128 | 0.8246 | 0.6767 |
| ***DMetS18a*** | | | | | | | | | | |
| Dcc | 71418392 | 72510723 | 0.5519 | 0.6839 | 0.5193 | 0.6790 | 0.1232 | 0.4337 | 0.6099 | 0.6421 |
| ***DMetS19a*** | | | | | | | | | | |
| Stx3 | 11849609 | 11893893 | 0.2162 | 0.4591 | 0.3596 | 0.6328 | 0.4974 | 0.6612 | 0.1210 | 0.4612 |
| Olfr1420 | 11970513 | 11971442 | 0.3847 | 0.5965 | 0.9358 | 0.7843 | 0.5393 | 0.6784 | 0.3393 | 0.5586 |
| Patl1 | 11986948 | 12019456 | 0.5732 | 0.6943 | 0.8143 | 0.7550 | 0.0652 | 0.3564 | 0.3433 | 0.5586 |
| Osbp | 12040431 | 12066533 | 0.9920 | 0.7909 | 0.8519 | 0.7671 | 0.7040 | 0.7111 | 0.5074 | 0.6150 |
| Olfr1424 | 12133299 | 12134240 | 0.4232 | 0.6249 | 0.0858 | 0.4149 | 0.5292 | 0.6754 | 0.3425 | 0.5586 |
| Olfr232 | 12342722 | 12343660 | 0.8334 | 0.7637 | 0.0926 | 0.4270 | 0.2720 | 0.5513 | 0.7827 | 0.6644 |
| Olfr1436 | 12372673 | 12373620 | 0.4772 | 0.6468 | 0.1431 | 0.4657 | 0.0129 | 0.2176 | 0.5193 | 0.6203 |
| Olfr1437 | 12396377 | 12397315 | 0.2872 | 0.5253 | 0.1844 | 0.5111 | 0.3323 | 0.6000 | 0.8765 | 0.6908 |
| Mpeg1 | 12535546 | 12538291 | 0.0300 | 0.1702 | 0.2401 | 0.5754 | 0.8240 | 0.7277 | 0.3959 | 0.5823 |
| Dtx4 | 12540831 | 12575944 | 0.2410 | 0.4910 | 0.4682 | 0.6681 | 0.3686 | 0.6193 | 0.7590 | 0.6611 |
| Fam111a | 12620230 | 12664258 | 0.0776 | 0.2898 | 0.0802 | 0.4040 | 0.8753 | 0.7435 | 0.5356 | 0.6215 |
| Gm4952 | 12674578 | 12702103 | 0.5121 | 0.6665 | 0.1309 | 0.4595 | 0.8164 | 0.7260 | 0.2447 | 0.5113 |
| Glyat | 12707798 | 12728401 | 0.1371 | 0.3812 | 0.3607 | 0.6328 | 0.2990 | 0.5712 | 0.3787 | 0.5763 |
| Olfr1443 | 12752656 | 12758333 | 0.3104 | 0.5409 | 0.4250 | 0.6536 | 0.1596 | 0.4642 | 0.3493 | 0.5654 |
| Keg1 | 12770276 | 12794392 | 0.6756 | 0.7288 | 0.3131 | 0.6077 | 0.4344 | 0.6467 | 0.9339 | 0.7003 |
| Zfp91 | 12838150 | 12870616 | 0.7251 | 0.7375 | 0.3310 | 0.6138 | 0.7292 | 0.7146 | 0.5851 | 0.6345 |
| Lpxn | 12873133 | 12908301 | 0.4475 | 0.6360 | 0.0083 | 0.2224 | 0.4850 | 0.6593 | 0.1593 | 0.4703 |
| EG433224 | 12980338 | 12980970 | 0.0016 | **0.0182** | 0.0000 | **0.0023** | 0.0000 | **0.0028** | 0.0013 | 0.2041 |
| Olfr1449 | 13009230 | 13010174 | 0.8877 | 0.7719 | 0.1255 | 0.4595 | 0.4599 | 0.6513 | 0.8416 | 0.6825 |
| Olfr1459 | 13220224 | 13221147 | 0.5234 | 0.6698 | 0.0143 | 0.2351 | 0.0810 | 0.3827 | 0.1601 | 0.4703 |
| Olfr1463 | 13308742 | 13309674 | 0.6960 | 0.7329 | 0.5961 | 0.7072 | 0.4405 | 0.6467 | 0.1490 | 0.4703 |
| Olfr1471 | 13519504 | 13520448 | 0.9736 | 0.7871 | 0.7647 | 0.7424 | 0.4658 | 0.6534 | 0.3238 | 0.5519 |
| Olfr1477 | 13575036 | 13577782 | 0.9011 | 0.7733 | 0.9473 | 0.7880 | 0.6662 | 0.7111 | 0.2093 | 0.5019 |
| Olfr1480 | 13604165 | 13605112 | 0.9312 | 0.7817 | 0.0367 | 0.3081 | 0.2548 | 0.5370 | 0.4445 | 0.5961 |
| Olfr1496 | 13855104 | 13856057 | 0.9191 | 0.7799 | 0.6261 | 0.7135 | 0.2833 | 0.5597 | 0.7879 | 0.6665 |
| Olfr1500 | 13901949 | 13902884 | 0.9346 | 0.7819 | 0.5383 | 0.6802 | 0.7006 | 0.7111 | 0.1740 | 0.4770 |
| Olfr1502 | 13936285 | 13937235 | 0.8422 | 0.7653 | 0.5323 | 0.6800 | 0.8060 | 0.7246 | 0.1604 | 0.4703 |
| Tle4 | 14522562 | 14672473 | 0.0000 | **0.0007** | 0.8783 | 0.7701 | 0.0331 | 0.2925 | 0.9374 | 0.7010 |
| Psat1 | 15979168 | 16021827 | 0.2132 | 0.4591 | 0.4202 | 0.6536 | 0.0016 | 0.0792 | 0.8621 | 0.6874 |
| Cep78 | 16030264 | 16059479 | 0.2672 | 0.5097 | 0.2067 | 0.5388 | 0.0083 | 0.1725 | 0.8812 | 0.6908 |
