# Supplementary material for: Genetic Effects at Pleiotropic Loci Are Context-Dependent with Consequences for the Maintenance of Genetic Variation in Populations
Source: PLoS Genet. 2011 Sep 8;7(9):e1002256. doi: 10.1371/journal.pgen.1002256 (PMC3169520; doi:10.1371/journal.pgen.1002256)
Supplement: Table S3 — Differential expression in MetS QTL among genes that are expressed in LG/J and SM/J strains in liver tissue. (DOC) [file pgen.1002256.s005.doc]

| **Gene** | **Start** | **Stop** | **p-val**  **(Strain)** | **q-val**  **(Strain)** | **p-val**  **(Diet * Sex * Strain)** | **q-val**  **(Diet * Sex* Strain)** | **p-val**  **(Diet * Strain)** | **q-val**  **(Diet * Strain)** | **p-val**  **(Sex * Strain)** | **q-val**  **(Sex * Strain)** |
| --- | --- | --- | --- | --- | --- | --- | --- | --- | --- | --- |
| ***DMetS1a*** | | | | | | | | | | |
| Epha4 | 77363760 | 77511663 | 0.5249 | 0.6445 | 0.1027 | 0.7008 | 0.3801 | 0.7233 | 0.5017 | 0.9106 |
| ***DMetS1b*** | | | | | | | | | | |
| Rgs5 | 171585632 | 171625944 | 0.7213 | 0.7009 | 0.7438 | 0.8937 | 0.5041 | 0.7761 | 0.6739 | 0.9430 |
| Rgs4 | 171671608 | 171677773 | 0.4540 | 0.6188 | 0.2559 | 0.7770 | 0.8794 | 0.8407 | 0.9061 | 0.9544 |
| 1700084C01Rik | 171858779 | 171864784 | 0.0919 | 0.3101 | 0.0390 | 0.5912 | 0.1088 | 0.5103 | 0.2596 | 0.8710 |
| Hsd17b7 | 171879666 | 171899372 | 0.0013 | **0.0136** | 0.7437 | 0.8937 | 0.0601 | 0.4581 | 0.7390 | 0.9430 |
| Ddr2 | 171907777 | 172040690 | 0.5969 | 0.6668 | 0.1348 | 0.7239 | 0.2843 | 0.6777 | 0.2313 | 0.8710 |
| Uap1 | 172072069 | 172105088 | 0.1318 | 0.3824 | 0.4211 | 0.8391 | 0.3149 | 0.6932 | 0.7166 | 0.9430 |
| Kist | 172123551 | 172145528 | 0.3883 | 0.5896 | 0.4894 | 0.8470 | 0.8471 | 0.8353 | 0.2942 | 0.8790 |
| 4930500M09Rik | 172144991 | 172145687 | 0.2529 | 0.5115 | 0.9561 | 0.9151 | 0.7504 | 0.8288 | 0.7303 | 0.9430 |
| Nos1ap | 172232799 | 172519992 | 0.6847 | 0.6933 | 0.0535 | 0.6276 | 0.4616 | 0.7606 | 0.9142 | 0.9544 |
| 1700015E13Rik | 172238992 | 172242258 | 0.3590 | 0.5775 | 0.6698 | 0.8868 | 0.9067 | 0.8467 | 0.8604 | 0.9544 |
| Olfml2b | 172574663 | 172612920 | 0.7363 | 0.7064 | 0.2173 | 0.7525 | 0.4213 | 0.7459 | 0.1371 | 0.8627 |
| Fcrla | 172847707 | 172857714 | 0.1837 | 0.4425 | 0.2856 | 0.7777 | 0.3320 | 0.6972 | 0.3437 | 0.8951 |
| Fcgr2b | 172890689 | 172906678 | 0.0646 | 0.2614 | 0.3069 | 0.7852 | 0.7317 | 0.8216 | 0.2116 | 0.8710 |
| Fcgr4 | 172949051 | 172959892 | 0.0000 | **0.0001** | 0.9536 | 0.9148 | 0.0022 | 0.1393 | 0.0017 | 0.4619 |
| Fcgr3 | 172981301 | 172989493 | 0.6782 | 0.6919 | 0.2468 | 0.7770 | 0.0010 | 0.1077 | 0.1091 | 0.8558 |
| 1700009P17Rik | 173044049 | 173057098 | 0.6292 | 0.6770 | 0.3069 | 0.7852 | 0.8691 | 0.8369 | 0.9260 | 0.9544 |
| Sdhc | 173057296 | 173080734 | 0.5802 | 0.6588 | 0.2385 | 0.7717 | 0.1874 | 0.6355 | 0.7813 | 0.9476 |
| Pcp4l1 | 173103393 | 173126399 | 0.0000 | **0.0000** | 0.2877 | 0.7801 | 0.3888 | 0.7263 | 0.0000 | **0.0092** |
| Nr1i3 | 173144101 | 173150832 | 0.0000 | **0.0000** | 0.8153 | 0.9032 | 0.9995 | 0.8591 | 0.5540 | 0.9281 |
| Apoa2 | 173155185 | 173156510 | 0.0000 | **0.0000** | 0.5875 | 0.8716 | 0.5834 | 0.8022 | 0.1102 | 0.8558 |
| Ndufs2 | 173164984 | 173177253 | 0.0855 | 0.3008 | 0.0043 | 0.3723 | 0.1243 | 0.5490 | 0.2259 | 0.8710 |
| Adamts4 | 173180552 | 173190768 | 0.1755 | 0.4354 | 0.4296 | 0.8426 | 0.9987 | 0.8591 | 0.4344 | 0.9106 |
| B4galt3 | 173200459 | 173207027 | 0.4100 | 0.5970 | 0.7157 | 0.8905 | 0.2306 | 0.6584 | 0.1999 | 0.8710 |
| Ppox | 173206121 | 173211317 | 0.0000 | **0.0001** | 0.8215 | 0.9032 | 0.0534 | 0.4581 | 0.3606 | 0.8971 |
| Usp21 | 173212076 | 173218122 | 0.0000 | **0.0000** | 0.6271 | 0.8758 | 0.2651 | 0.6777 | 0.9724 | 0.9609 |
| Ufc1 | 173218694 | 173225155 | 0.0681 | 0.2730 | 0.2750 | 0.7770 | 0.2277 | 0.6584 | 0.4452 | 0.9106 |
| EG622236 | 173235215 | 173235433 | 0.8497 | 0.7207 | 0.5701 | 0.8705 | 0.3990 | 0.7322 | 0.9216 | 0.9544 |
| Dedd | 173259276 | 173272462 | 0.0313 | 0.1598 | 0.7871 | 0.9000 | 0.6819 | 0.8083 | 0.8835 | 0.9544 |
| Nit1 | 173268139 | 173275777 | 0.0291 | 0.1518 | 0.2736 | 0.7770 | 0.9021 | 0.8450 | 0.4531 | 0.9106 |
| Pfdn2 | 173275801 | 173289385 | 0.0209 | 0.1200 | 0.2845 | 0.7777 | 0.6719 | 0.8083 | 0.9353 | 0.9544 |
| Klhdc9 | 173288577 | 173290929 | 0.4486 | 0.6188 | 0.7425 | 0.8937 | 0.5637 | 0.7990 | 0.9275 | 0.9544 |
| Pvrl4 | 173300230 | 173318729 | 0.2766 | 0.5291 | 0.8565 | 0.9082 | 0.6030 | 0.8022 | 0.0810 | 0.8387 |
| Arhgap30 | 173319085 | 173340429 | 0.6764 | 0.6916 | 0.6028 | 0.8716 | 0.1418 | 0.5661 | 0.5993 | 0.9408 |
| Usf1 | 173341444 | 173349273 | 0.0000 | **0.0000** | 0.5151 | 0.8636 | 0.3127 | 0.6931 | 0.9421 | 0.9544 |
| EG226654 | 173349164 | 173350485 | 0.0000 | **0.0001** | 0.3236 | 0.7900 | 0.7165 | 0.8146 | 0.0429 | 0.8252 |
| F11r | 173367666 | 173394734 | 0.0000 | **0.0000** | 0.6319 | 0.8758 | 0.4100 | 0.7390 | 0.8518 | 0.9544 |
| Refbp2 | 173433609 | 173434881 | 0.0071 | 0.0545 | 0.9947 | 0.9208 | 0.8525 | 0.8368 | 0.2140 | 0.8710 |
| Cd244 | 173489324 | 173515449 | 0.1435 | 0.3969 | 0.5804 | 0.8716 | 0.6899 | 0.8083 | 0.6303 | 0.9430 |
| Ly9 | 173518755 | 173537541 | 0.0711 | 0.2783 | 0.2410 | 0.7754 | 0.0799 | 0.4879 | 0.7043 | 0.9430 |
| ***DMetS2a*** | | | | | | | | | | |
| 4932414N04Rik | 68494543 | 68586520 | 0.7441 | 0.7082 | 0.3734 | 0.8268 | 0.6051 | 0.8022 | 0.1672 | 0.8710 |
| Lass6 | 68699498 | 68952339 | 0.3808 | 0.5896 | 0.2148 | 0.7525 | 0.9285 | 0.8520 | 0.0244 | 0.8252 |
| Nostrin | 68973857 | 69027387 | 0.4856 | 0.6349 | 0.8674 | 0.9086 | 0.5370 | 0.7932 | 0.2571 | 0.8710 |
| Spc25 | 69031952 | 69044251 | 0.0001 | **0.0018** | 0.6859 | 0.8899 | 0.0898 | 0.4980 | 0.3794 | 0.8986 |
| G6pc2 | 69049130 | 69065898 | 0.1714 | 0.4322 | 0.6246 | 0.8758 | 0.9936 | 0.8591 | 0.8287 | 0.9531 |
| Abcb11 | 69076339 | 69180671 | 0.0000 | **0.0000** | 0.8436 | 0.9070 | 0.0031 | 0.1582 | 0.5067 | 0.9152 |
| Lrp2 | 69262397 | 69424122 | 0.5275 | 0.6458 | 0.9057 | 0.9124 | 0.8536 | 0.8369 | 0.5333 | 0.9248 |
| Bbs5 | 69485228 | 69505628 | 0.3939 | 0.5914 | 0.4210 | 0.8391 | 0.3625 | 0.7093 | 0.9898 | 0.9621 |
| Kbtbd10 | 69508177 | 69522287 | 0.2991 | 0.5453 | 0.5438 | 0.8653 | 0.2350 | 0.6586 | 0.6625 | 0.9430 |
| Fastkd1 | 69524872 | 69551573 | 0.0054 | **0.0440** | 0.5631 | 0.8666 | 0.0036 | 0.1743 | 0.4463 | 0.9106 |
| Ppig | 69560602 | 69592069 | 0.5983 | 0.6668 | 0.1090 | 0.7019 | 0.8920 | 0.8443 | 0.0217 | 0.8252 |
| Phospho2 | 69627680 | 69638062 | 0.0698 | 0.2765 | 0.3140 | 0.7877 | 0.0798 | 0.4879 | 0.9245 | 0.9544 |
| Klhl23 | 69660001 | 69674708 | 0.2941 | 0.5423 | 0.5833 | 0.8716 | 0.6072 | 0.8022 | 0.7231 | 0.9430 |
| Ssb | 69699619 | 69709843 | 0.7099 | 0.7004 | 0.3405 | 0.8014 | 0.3012 | 0.6907 | 0.1319 | 0.8627 |
| Zfp650 | 69735303 | 69862070 | 0.5265 | 0.6452 | 0.2304 | 0.7650 | 0.6999 | 0.8105 | 0.5055 | 0.9144 |
| Sp5 | 70312980 | 70315786 | 0.9045 | 0.7311 | 0.0624 | 0.6276 | 0.4975 | 0.7719 | 0.5851 | 0.9366 |
| 4933404M02Rik | 70346876 | 70378941 | 0.1667 | 0.4269 | 0.0330 | 0.5912 | 0.3922 | 0.7263 | 0.3605 | 0.8971 |
| Gorasp2 | 70499633 | 70550693 | 0.5505 | 0.6476 | 0.0083 | 0.3723 | 0.3017 | 0.6907 | 0.1185 | 0.8627 |
| Tlk1 | 70550464 | 70663785 | 0.0864 | 0.3008 | 0.8819 | 0.9095 | 0.0734 | 0.4823 | 0.6629 | 0.9430 |
| Mettl8 | 70802618 | 70893640 | 0.5357 | 0.6476 | 0.2531 | 0.7770 | 0.4948 | 0.7719 | 0.2149 | 0.8710 |
| 4833418A01Rik | 70893385 | 70937199 | 0.6516 | 0.6838 | 0.8124 | 0.9032 | 0.2152 | 0.6508 | 0.3294 | 0.8930 |
| 4833418A01Rik | 70893385 | 70937199 | 0.5685 | 0.6560 | 0.1176 | 0.7079 | 0.6913 | 0.8083 | 0.2909 | 0.8778 |
| Dync1i2 | 71049763 | 71101360 | 0.6518 | 0.6838 | 0.9177 | 0.9124 | 0.6017 | 0.8022 | 0.5364 | 0.9250 |
| Slc25a12 | 71109120 | 71205806 | 0.0564 | 0.2359 | 0.9859 | 0.9207 | 0.6666 | 0.8083 | 0.4547 | 0.9106 |
| Hat1 | 71227015 | 71279679 | 0.0043 | **0.0369** | 0.5345 | 0.8653 | 0.0281 | 0.3594 | 0.1714 | 0.8710 |
| Metapl1 | 71291333 | 71363251 | 0.8044 | 0.7207 | 0.3985 | 0.8352 | 0.3927 | 0.7263 | 0.5366 | 0.9250 |
| Dlx1as | 71354511 | 71375962 | 0.2081 | 0.4646 | 0.4257 | 0.8410 | 0.5485 | 0.7990 | 0.4433 | 0.9106 |
| Dlx2 | 71381466 | 71384749 | 0.4483 | 0.6188 | 0.8982 | 0.9124 | 0.4798 | 0.7700 | 0.3045 | 0.8790 |
| Gm1631 | 71557446 | 71569023 | 0.4038 | 0.5968 | 0.2065 | 0.7519 | 0.5893 | 0.8022 | 0.7469 | 0.9430 |
| Itga6 | 71583673 | 71696473 | 0.0025 | **0.0231** | 0.1113 | 0.7061 | 0.1733 | 0.6191 | 0.0721 | 0.8387 |
| Pdk1 | 71711281 | 71741915 | 0.0000 | **0.0000** | 0.4075 | 0.8380 | 0.0468 | 0.4332 | 0.4565 | 0.9106 |
| Rapgef4 | 71819297 | 72095531 | 0.0081 | 0.0600 | 0.8821 | 0.9095 | 0.3646 | 0.7095 | 0.9281 | 0.9544 |
| ***DMetS2b*** | | | | | | | | | | |
| Zc3h15 | 83484592 | 83504779 | 0.3045 | 0.5497 | 0.8924 | 0.9103 | 0.4608 | 0.7606 | 0.2430 | 0.8710 |
| Itgav | 83564554 | 83647073 | 0.8484 | 0.7207 | 0.6915 | 0.8899 | 0.1608 | 0.6035 | 0.3730 | 0.8971 |
| Fam171b | 83652793 | 83723643 | 0.0000 | **0.0000** | 0.8738 | 0.9095 | 0.4073 | 0.7390 | 0.0416 | 0.8252 |
| Zswim2 | 83755236 | 83781385 | 0.8503 | 0.7207 | 0.9176 | 0.9124 | 0.5310 | 0.7919 | 0.6612 | 0.9430 |
| Tfpi | 84273012 | 84316932 | 0.0135 | 0.0889 | 0.6075 | 0.8716 | 0.6034 | 0.8022 | 0.7468 | 0.9430 |
| Catns | 84440228 | 84490922 | 0.5231 | 0.6445 | 0.8685 | 0.9086 | 0.5325 | 0.7919 | 0.8759 | 0.9544 |
| 2700094K13Rik | 84509375 | 84510927 | 0.0003 | **0.0041** | 0.8974 | 0.9124 | 0.0009 | 0.1077 | 0.5070 | 0.9152 |
| Med19 | 84518559 | 84528372 | 0.5966 | 0.6668 | 0.0759 | 0.6367 | 0.0711 | 0.4823 | 0.6057 | 0.9408 |
| Zdhhc5 | 84528127 | 84555337 | 0.8440 | 0.7207 | 0.1733 | 0.7387 | 0.8662 | 0.8369 | 0.1501 | 0.8710 |
| Clp1 | 84563279 | 84567507 | 0.8259 | 0.7207 | 0.1305 | 0.7239 | 0.3162 | 0.6935 | 0.9414 | 0.9544 |
| Ypel4 | 84574215 | 84578812 | 0.4838 | 0.6331 | 0.3363 | 0.7975 | 0.0927 | 0.4980 | 0.8389 | 0.9531 |
| Serping1 | 84605544 | 84615601 | 0.0000 | **0.0000** | 0.8129 | 0.9032 | 0.2347 | 0.6586 | 0.6983 | 0.9430 |
| Ube2l6 | 84638985 | 84650492 | 0.0113 | 0.0791 | 0.2585 | 0.7770 | 0.0215 | 0.3203 | 0.3402 | 0.8949 |
| Smtnl1 | 84651333 | 84662809 | 0.2692 | 0.5222 | 0.4448 | 0.8426 | 0.2470 | 0.6728 | 0.5162 | 0.9218 |
| Timm10 | 84667154 | 84670370 | 0.0000 | **0.0008** | 0.8684 | 0.9086 | 0.2744 | 0.6777 | 0.1648 | 0.8710 |
| Slc43a1 | 84679007 | 84703751 | 0.0029 | **0.0265** | 0.5775 | 0.8716 | 0.4067 | 0.7390 | 0.8395 | 0.9531 |
| Slc43a3 | 84776736 | 84798666 | 0.7316 | 0.7058 | 0.7405 | 0.8937 | 0.4936 | 0.7719 | 0.5494 | 0.9281 |
| Prg2 | 84820618 | 84823789 | 0.2391 | 0.5019 | 0.2533 | 0.7770 | 0.2913 | 0.6819 | 0.0742 | 0.8387 |
| Prg3 | 84828372 | 84834043 | 0.3368 | 0.5649 | 0.2696 | 0.7770 | 0.3378 | 0.6972 | 0.2037 | 0.8710 |
| Ssrp1 | 84877391 | 84887266 | 0.2425 | 0.5020 | 0.0057 | 0.3723 | 0.1126 | 0.5232 | 0.6724 | 0.9430 |
| Tnks1bp1 | 84888179 | 84913205 | 0.0785 | 0.2894 | 0.8211 | 0.9032 | 0.2821 | 0.6777 | 0.1238 | 0.8627 |
| Aplnr | 84976557 | 84980080 | 0.0220 | 0.1251 | 0.7464 | 0.8937 | 0.1194 | 0.5367 | 0.1144 | 0.8558 |
| Olfr988 | 85193152 | 85203215 | 0.1235 | 0.3686 | 0.9028 | 0.9124 | 0.5292 | 0.7909 | 0.6066 | 0.9408 |
| Olfr996 | 85419398 | 85420342 | 0.8481 | 0.7207 | 0.9449 | 0.9148 | 0.3329 | 0.6972 | 0.7057 | 0.9430 |
| Olfr1006 | 85514368 | 85515330 | 0.7342 | 0.7064 | 0.0375 | 0.5912 | 0.8153 | 0.8348 | 0.0574 | 0.8252 |
| Olfr1008 | 85529588 | 85530529 | 0.8238 | 0.7207 | 0.5249 | 0.8652 | 0.5111 | 0.7775 | 0.0332 | 0.8252 |
| Olfr1012 | 85599596 | 85600531 | 0.4935 | 0.6357 | 0.2098 | 0.7525 | 0.0399 | 0.3995 | 0.9064 | 0.9544 |
| Olfr1013 | 85609960 | 85610877 | 0.3745 | 0.5869 | 0.3639 | 0.8199 | 0.5393 | 0.7942 | 0.3287 | 0.8930 |
| Olfr1020 | 85689611 | 85690564 | 0.1538 | 0.4077 | 0.3736 | 0.8268 | 0.3900 | 0.7263 | 0.4642 | 0.9106 |
| Olfr1023 | 85726959 | 85727894 | 0.4896 | 0.6354 | 0.8193 | 0.9032 | 0.5321 | 0.7919 | 0.9483 | 0.9544 |
| Olfr1030 | 85823999 | 85824955 | 0.7817 | 0.7183 | 0.4351 | 0.8426 | 0.7973 | 0.8348 | 0.3169 | 0.8883 |
| Olfr1031 | 85831976 | 85832986 | 0.6298 | 0.6770 | 0.6820 | 0.8890 | 0.8228 | 0.8348 | 0.8920 | 0.9544 |
| Olfr1032 | 85847935 | 85848867 | 0.7113 | 0.7007 | 0.9967 | 0.9212 | 0.7694 | 0.8321 | 0.6106 | 0.9408 |
| Olfr1043 | 86002160 | 86003104 | 0.1133 | 0.3530 | 0.9065 | 0.9124 | 0.8181 | 0.8348 | 0.2863 | 0.8778 |
| Olfr1049 | 86094922 | 86095848 | 0.8661 | 0.7240 | 0.6735 | 0.8868 | 0.4210 | 0.7459 | 0.4815 | 0.9106 |
| Olfr1052 | 86137975 | 86138913 | 0.1517 | 0.4062 | 0.0840 | 0.6584 | 0.8632 | 0.8369 | 0.7715 | 0.9476 |
| Olfr1065 | 86285196 | 86286137 | 0.1975 | 0.4567 | 0.0679 | 0.6276 | 0.0162 | 0.3177 | 0.7482 | 0.9430 |
| Olfr1085 | 86497672 | 86498613 | 0.1816 | 0.4414 | 0.4195 | 0.8391 | 0.9955 | 0.8591 | 0.2865 | 0.8778 |
| Olfr1094 | 86668911 | 86669903 | 0.4269 | 0.6071 | 0.5142 | 0.8636 | 0.7631 | 0.8321 | 0.4225 | 0.9106 |
| Olfr1099 | 86798675 | 86799613 | 0.2034 | 0.4612 | 0.6002 | 0.8716 | 0.3110 | 0.6922 | 0.8898 | 0.9544 |
| Olfr1105 | 86873438 | 86874376 | 0.3703 | 0.5869 | 0.2222 | 0.7561 | 0.2696 | 0.6777 | 0.4115 | 0.9106 |
| Olfr259 | 86947604 | 86948542 | 0.4938 | 0.6357 | 0.2964 | 0.7822 | 0.1823 | 0.6323 | 0.7688 | 0.9476 |
| Olfr1110 | 86975538 | 86976476 | 0.9674 | 0.7437 | 0.4160 | 0.8380 | 0.4690 | 0.7606 | 0.6139 | 0.9408 |
| Olfr1113 | 87053051 | 87054031 | 0.5760 | 0.6563 | 0.1101 | 0.7061 | 0.9083 | 0.8470 | 0.6386 | 0.9430 |
| Olfr1130 | 87447547 | 87448491 | 0.0828 | 0.2948 | 0.8135 | 0.9032 | 0.0885 | 0.4959 | 0.9917 | 0.9623 |
| Olfr1132 | 87474976 | 87475902 | 0.9464 | 0.7410 | 0.0674 | 0.6276 | 0.8733 | 0.8385 | 0.2979 | 0.8790 |
| Olfr1161 | 87864881 | 87865846 | 0.4654 | 0.6236 | 0.6664 | 0.8868 | 0.4320 | 0.7531 | 0.2780 | 0.8733 |
| Olfr1166 | 87964190 | 87965140 | 0.3152 | 0.5560 | 0.2670 | 0.7770 | 0.1950 | 0.6404 | 0.3420 | 0.8949 |
| Olfr1167 | 87989224 | 87990174 | 0.3847 | 0.5896 | 0.8393 | 0.9062 | 0.2498 | 0.6728 | 0.2266 | 0.8710 |
| ***DMetS2c*** | | | | | | | | | | |
| Cd44 | 102651298 | 102741767 | 0.1646 | 0.4246 | 0.1117 | 0.7061 | 0.0535 | 0.4581 | 0.9417 | 0.9544 |
| Apip | 102913832 | 102932801 | 0.2529 | 0.5115 | 0.0372 | 0.5912 | 0.1880 | 0.6355 | 0.4638 | 0.9106 |
| Elf5 | 103251870 | 103291146 | 0.3301 | 0.5630 | 0.4191 | 0.8391 | 0.1170 | 0.5321 | 0.3064 | 0.8790 |
| Cat | 103294006 | 103325317 | 0.0000 | **0.0000** | 0.5029 | 0.8538 | 0.5133 | 0.7793 | 0.1277 | 0.8627 |
| Nat10 | 103561413 | 103601427 | 0.1985 | 0.4567 | 0.5284 | 0.8652 | 0.0265 | 0.3499 | 0.8958 | 0.9544 |
| Caprin1 | 103603098 | 103637806 | 0.0752 | 0.2873 | 0.9288 | 0.9124 | 0.3307 | 0.6972 | 0.1479 | 0.8710 |
| 4930547E08Rik | 103644609 | 103650779 | 0.4667 | 0.6238 | 0.3574 | 0.8148 | 0.4205 | 0.7459 | 0.9490 | 0.9544 |
| Lmo2 | 103798143 | 103822031 | 0.0000 | **0.0000** | 0.4062 | 0.8365 | 0.0010 | 0.1077 | 0.2588 | 0.8710 |
| 4931422A03Rik | 103807100 | 103868518 | 0.5912 | 0.6668 | 0.3029 | 0.7852 | 0.5786 | 0.8019 | 0.5694 | 0.9301 |
| Fbxo3 | 103867878 | 103903397 | 0.0051 | **0.0423** | 0.9170 | 0.9124 | 0.1802 | 0.6293 | 0.5931 | 0.9399 |
| Cd59b | 103910006 | 103931344 | 0.0000 | **0.0000** | 0.7362 | 0.8937 | 0.0937 | 0.4980 | 0.4781 | 0.9106 |
| Cd59a | 103935958 | 103955511 | 0.0000 | **0.0003** | 0.4616 | 0.8426 | 0.3221 | 0.6957 | 0.1704 | 0.8710 |
| A930018P22Rik | 103962926 | 103964906 | 0.0021 | **0.0199** | 0.2731 | 0.7770 | 0.3073 | 0.6922 | 0.1729 | 0.8710 |
| D430041D05Rik | 103983230 | 104250150 | 0.2620 | 0.5180 | 0.7852 | 0.9000 | 0.6831 | 0.8083 | 0.0627 | 0.8356 |
| Hipk3 | 104266638 | 104334603 | 0.0080 | 0.0600 | 0.1345 | 0.7239 | 0.1780 | 0.6260 | 0.1362 | 0.8627 |
| Cstf3 | 104430680 | 104505586 | 0.0000 | **0.0000** | 0.0630 | 0.6276 | 0.1991 | 0.6407 | 0.1616 | 0.8710 |
| EG622282 | 104551832 | 104552931 | 0.6053 | 0.6703 | 0.7522 | 0.8937 | 0.9251 | 0.8520 | 0.9030 | 0.9544 |
| 4732486I23Rik | 104594952 | 104656853 | 0.0006 | **0.0069** | 0.4802 | 0.8434 | 0.8317 | 0.8348 | 0.6517 | 0.9430 |
| 2310047K21Rik | 104656991 | 104659148 | 0.0001 | **0.0019** | 0.0486 | 0.6276 | 0.5091 | 0.7775 | 0.9580 | 0.9587 |
| Ga17 | 104839813 | 104857237 | 0.4069 | 0.5970 | 0.6203 | 0.8758 | 0.6283 | 0.8083 | 0.9343 | 0.9544 |
| 0610012H03Rik | 105064477 | 105219953 | 0.0109 | 0.0768 | 0.1946 | 0.7387 | 0.2288 | 0.6584 | 0.5672 | 0.9298 |
| Dph4 | 105806866 | 105843706 | 0.0097 | 0.0697 | 0.1876 | 0.7387 | 0.0472 | 0.4332 | 0.4698 | 0.9106 |
| ***DMetS4a*** | | | | | | | | | | |
| OTTMUSG00000010671 | 146866526 | 146887595 | 0.1386 | 0.3921 | 0.1347 | 0.7239 | 0.6698 | 0.8083 | 0.5448 | 0.9281 |
| Gm13154 | 146927386 | 146959307 | 0.3757 | 0.5869 | 0.1352 | 0.7239 | 0.2799 | 0.6777 | 0.1832 | 0.8710 |
| 2610305D13Rik | 146986046 | 147016622 | 0.0001 | **0.0013** | 0.4378 | 0.8426 | 0.0609 | 0.4581 | 0.1506 | 0.8710 |
| Gm13143 | 147197095 | 147222475 | 0.4362 | 0.6131 | 0.5397 | 0.8653 | 0.0342 | 0.3797 | 0.8842 | 0.9544 |
| D4Wsu114e | 147234887 | 147242775 | 0.0001 | **0.0013** | 0.8888 | 0.9095 | 0.9087 | 0.8470 | 0.0775 | 0.8387 |
| Fv1 | 147243088 | 147244467 | 0.0000 | **0.0006** | 0.0067 | 0.3723 | 0.7511 | 0.8288 | 0.0808 | 0.8387 |
| Mfn2 | 147247708 | 147278813 | 0.0077 | 0.0586 | 0.1517 | 0.7256 | 0.1923 | 0.6355 | 0.2684 | 0.8733 |
| Plod1 | 147283862 | 147310876 | 0.0282 | 0.1502 | 0.3766 | 0.8268 | 0.8843 | 0.8435 | 0.0335 | 0.8252 |
| 2510039O18Rik | 147315004 | 147321423 | 0.1391 | 0.3926 | 0.5410 | 0.8653 | 0.9264 | 0.8520 | 0.1203 | 0.8627 |
| Clcn6 | 147378368 | 147412930 | 0.7372 | 0.7064 | 0.1538 | 0.7258 | 0.7733 | 0.8321 | 0.9462 | 0.9544 |
| Agtrap | 147451170 | 147462140 | 0.0003 | **0.0034** | 0.4487 | 0.8426 | 0.3590 | 0.7085 | 0.5813 | 0.9349 |
| 2610109H07Rik | 147472546 | 147504807 | 0.3978 | 0.5946 | 0.9684 | 0.9153 | 0.8336 | 0.8348 | 0.8116 | 0.9531 |
| Mad2l2 | 147504493 | 147519808 | 0.0203 | 0.1181 | 0.7401 | 0.8937 | 0.8120 | 0.8348 | 0.9750 | 0.9614 |
| Fbxo6 | 147519825 | 147526249 | 0.0000 | **0.0000** | 0.0386 | 0.5912 | 0.5873 | 0.8022 | 0.1337 | 0.8627 |
| Fbxo44 | 147526912 | 147534203 | 0.9714 | 0.7446 | 0.5223 | 0.8652 | 0.2244 | 0.6567 | 0.3430 | 0.8951 |
| Fbs1 | 147534730 | 147540533 | 0.8462 | 0.7207 | 0.9407 | 0.9139 | 0.0421 | 0.4060 | 0.8993 | 0.9544 |
| Ptchd2 | 147614373 | 147662074 | 0.6217 | 0.6760 | 0.9579 | 0.9151 | 0.8862 | 0.8435 | 0.9897 | 0.9621 |
| Ubiad1 | 147808604 | 147818880 | 0.0000 | **0.0000** | 0.5128 | 0.8636 | 0.8134 | 0.8348 | 0.3722 | 0.8971 |
| Frap1 | 147822720 | 147931792 | 0.3066 | 0.5511 | 0.9996 | 0.9232 | 0.6694 | 0.8083 | 0.3760 | 0.8975 |
| Angptl7 | 147869292 | 147874569 | 0.6208 | 0.6760 | 0.0039 | 0.3723 | 0.7881 | 0.8321 | 0.0398 | 0.8252 |
| Exosc10 | 147932538 | 147956510 | 0.7138 | 0.7009 | 0.0208 | 0.5888 | 0.5275 | 0.7909 | 0.7895 | 0.9517 |
| Srm | 147965612 | 147969102 | 0.0002 | **0.0025** | 0.0061 | 0.3723 | 0.0050 | 0.2097 | 0.0249 | 0.8252 |
| Masp2 | 147976663 | 147989608 | 0.0000 | **0.0000** | 0.8641 | 0.9084 | 0.1900 | 0.6355 | 0.7267 | 0.9430 |
| Casz1 | 148178538 | 148328998 | 0.5642 | 0.6555 | 0.5131 | 0.8636 | 0.8462 | 0.8349 | 0.5284 | 0.9248 |
| Pex14 | 148334644 | 148473985 | 0.6640 | 0.6881 | 0.0553 | 0.6276 | 0.7414 | 0.8255 | 0.7523 | 0.9430 |
| Dffa | 148478255 | 148494756 | 0.0359 | 0.1745 | 0.1357 | 0.7239 | 0.0089 | 0.2620 | 0.0405 | 0.8252 |
| Cort | 148499143 | 148500872 | 0.0238 | 0.1333 | 0.3926 | 0.8352 | 0.5900 | 0.8022 | 0.7280 | 0.9430 |
| Apitd1 | 148501230 | 148511738 | 0.0140 | 0.0904 | 0.9222 | 0.9124 | 0.0083 | 0.2509 | 0.2173 | 0.8710 |
| Pgd | 148524100 | 148540880 | 0.0159 | 0.0998 | 0.0353 | 0.5912 | 0.0239 | 0.3298 | 0.1657 | 0.8710 |
| Kif1b | 148550428 | 148681802 | 0.1909 | 0.4526 | 0.1470 | 0.7239 | 0.3073 | 0.6922 | 0.6688 | 0.9430 |
| Ube4b | 148702525 | 148800858 | 0.6979 | 0.6962 | 0.6701 | 0.8868 | 0.9657 | 0.8567 | 0.7920 | 0.9518 |
| Rbp7 | 148823796 | 148829087 | 0.2399 | 0.5019 | 0.4977 | 0.8524 | 0.1477 | 0.5761 | 0.6910 | 0.9430 |
| Nmnat1 | 148841681 | 148859311 | 0.2402 | 0.5019 | 0.6272 | 0.8758 | 0.2380 | 0.6623 | 0.2058 | 0.8710 |
| Lzic | 148859338 | 148870777 | 0.1226 | 0.3676 | 0.5173 | 0.8648 | 0.1437 | 0.5694 | 0.8283 | 0.9531 |
| Ctnnbip1 | 148892345 | 148940546 | 0.0000 | **0.0007** | 0.8701 | 0.9093 | 0.6811 | 0.8083 | 0.5855 | 0.9366 |
| Pik3cd | 149023277 | 149076680 | 0.6573 | 0.6873 | 0.1799 | 0.7387 | 0.0921 | 0.4980 | 0.8175 | 0.9531 |
| Tmem201 | 149089484 | 149112153 | 0.0016 | **0.0157** | 0.9227 | 0.9124 | 0.7683 | 0.8321 | 0.6820 | 0.9430 |
| Slc25a33 | 149118145 | 149148386 | 0.0316 | 0.1604 | 0.5032 | 0.8538 | 0.2399 | 0.6651 | 0.0135 | 0.8252 |
| Spsb1 | 149270392 | 149329152 | 0.0000 | **0.0008** | 0.0951 | 0.6961 | 0.8678 | 0.8369 | 0.5875 | 0.9366 |
| H6pd | 149353584 | 149383132 | 0.0064 | 0.0507 | 0.7701 | 0.8977 | 0.0416 | 0.4037 | 0.7517 | 0.9430 |
| Eno1 | 149610830 | 149622988 | 0.0065 | 0.0507 | 0.0562 | 0.6276 | 0.7529 | 0.8288 | 0.1783 | 0.8710 |
| Errfi1 | 150228028 | 150243001 | 0.0061 | **0.0484** | 0.5002 | 0.8536 | 0.4755 | 0.7650 | 0.2766 | 0.8733 |
| Park7 | 150271242 | 150288546 | 0.0638 | 0.2595 | 0.1940 | 0.7387 | 0.0414 | 0.4037 | 0.0734 | 0.8387 |
| Tnfrsf9 | 150288671 | 150320211 | 0.6220 | 0.6760 | 0.9291 | 0.9124 | 0.2509 | 0.6728 | 0.7119 | 0.9430 |
| Per3 | 150377761 | 150418774 | 0.4610 | 0.6196 | 0.7505 | 0.8937 | 0.7008 | 0.8110 | 0.9386 | 0.9544 |
| Vamp3 | 150421409 | 150432072 | 0.0171 | 0.1046 | 0.6411 | 0.8795 | 0.8247 | 0.8348 | 0.4768 | 0.9106 |
| Camta1 | 150433634 | 151235985 | 0.0250 | 0.1377 | 0.6917 | 0.8899 | 0.0639 | 0.4664 | 0.5820 | 0.9349 |
| Dnajc11 | 151307800 | 151356246 | 0.0022 | **0.0208** | 0.2272 | 0.7597 | 0.4751 | 0.7650 | 0.0790 | 0.8387 |
| Thap3 | 151356748 | 151363106 | 0.5319 | 0.6465 | 0.7484 | 0.8937 | 0.1945 | 0.6400 | 0.7062 | 0.9430 |
| Phf13 | 151363742 | 151370367 | 0.0230 | 0.1299 | 0.0944 | 0.6947 | 0.5830 | 0.8022 | 0.7254 | 0.9430 |
| Klhl21 | 151382912 | 151391789 | 0.8248 | 0.7207 | 0.1602 | 0.7314 | 0.7793 | 0.8321 | 0.4840 | 0.9106 |
| Nol9 | 151413430 | 151435603 | 0.2560 | 0.5138 | 0.7098 | 0.8905 | 0.7365 | 0.8226 | 0.3864 | 0.9007 |
| Plekhg5 | 151446607 | 151489509 | 0.1987 | 0.4567 | 0.5309 | 0.8652 | 0.2296 | 0.6584 | 0.0854 | 0.8474 |
| Tnfrsf25 | 151490043 | 151494228 | 0.0560 | 0.2347 | 0.1498 | 0.7256 | 0.7660 | 0.8321 | 0.4671 | 0.9106 |
| Espn | 151494440 | 151526480 | 0.0018 | **0.0176** | 0.4369 | 0.8426 | 0.8704 | 0.8369 | 0.1357 | 0.8627 |
| Acot7 | 151552243 | 151645961 | 0.3896 | 0.5896 | 0.4228 | 0.8392 | 0.2717 | 0.6777 | 0.9560 | 0.9580 |
| Gpr153 | 151648341 | 151659446 | 0.0652 | 0.2628 | 0.5443 | 0.8653 | 0.6501 | 0.8083 | 0.4237 | 0.9106 |
| Icmt | 151671336 | 151681230 | 0.5764 | 0.6563 | 0.8590 | 0.9082 | 0.1557 | 0.5899 | 0.0480 | 0.8252 |
| Rnf207 | 151681128 | 151693083 | 0.0254 | 0.1378 | 0.7097 | 0.8905 | 0.0211 | 0.3177 | 0.2095 | 0.8710 |
| Rpl22 | 151699851 | 151708180 | 0.0000 | **0.0000** | 0.8055 | 0.9032 | 0.0253 | 0.3411 | 0.6445 | 0.9430 |
| Chd5 | 151712996 | 151764290 | 0.2813 | 0.5327 | 0.9144 | 0.9124 | 0.5649 | 0.7990 | 0.2228 | 0.8710 |
| Kcnab2 | 151764851 | 151851980 | 0.4470 | 0.6188 | 0.5757 | 0.8716 | 0.0911 | 0.4980 | 0.6908 | 0.9430 |
| Nphp4 | 151850815 | 151937158 | 0.0523 | 0.2264 | 0.1750 | 0.7387 | 0.4433 | 0.7538 | 0.9294 | 0.9544 |
| Ajap1 | 152747330 | 152856939 | 0.3508 | 0.5697 | 0.7386 | 0.8937 | 0.9417 | 0.8520 | 0.5705 | 0.9306 |
| ***DMetS6a*** | | | | | | | | | | |
| Cntnap2 | 45010060 | 47251368 | 0.8968 | 0.7311 | 0.3527 | 0.8128 | 0.9425 | 0.8520 | 0.4283 | 0.9106 |
| Cul1 | 47403397 | 47476138 | 0.0119 | 0.0816 | 0.7811 | 0.9000 | 0.7643 | 0.8321 | 0.6648 | 0.9430 |
| Ezh2 | 47480273 | 47545340 | 0.3107 | 0.5528 | 0.0505 | 0.6276 | 0.1025 | 0.5038 | 0.6093 | 0.9408 |
| Pdia4 | 47746141 | 47763355 | 0.8034 | 0.7207 | 0.8776 | 0.9095 | 0.0179 | 0.3177 | 0.0366 | 0.8252 |
| Zfp786 | 47769265 | 47780866 | 0.5711 | 0.6563 | 0.8884 | 0.9095 | 0.7157 | 0.8146 | 0.5003 | 0.9106 |
| Zfp398 | 47785660 | 47818256 | 0.0858 | 0.3008 | 0.2982 | 0.7849 | 0.8800 | 0.8407 | 0.4300 | 0.9106 |
| Zfp282 | 47827203 | 47858484 | 0.6277 | 0.6770 | 0.5288 | 0.8652 | 0.3820 | 0.7240 | 0.1376 | 0.8627 |
| Zfp212 | 47870475 | 47882638 | 0.0187 | 0.1118 | 0.4922 | 0.8483 | 0.0661 | 0.4748 | 0.4043 | 0.9106 |
| AI894139 | 47903389 | 47915299 | 0.7355 | 0.7064 | 0.5185 | 0.8648 | 0.4602 | 0.7603 | 0.6091 | 0.9408 |
| 2500002G23Rik | 47974204 | 47998910 | 0.1666 | 0.4269 | 0.0035 | 0.3723 | 0.8332 | 0.8348 | 0.7136 | 0.9430 |
| Zfp746 | 48012396 | 48036592 | 0.2702 | 0.5228 | 0.7668 | 0.8977 | 0.3433 | 0.6990 | 0.8045 | 0.9531 |
| A930040G15Rik | 48345585 | 48369544 | 0.1522 | 0.4066 | 0.9782 | 0.9194 | 0.6451 | 0.8083 | 0.2726 | 0.8733 |
| Zfp467 | 48377696 | 48395824 | 0.1116 | 0.3509 | 0.0011 | 0.3442 | 0.4523 | 0.7552 | 0.2097 | 0.8710 |
| Atp6v0e2 | 48487700 | 48491793 | 0.1813 | 0.4414 | 0.1155 | 0.7079 | 0.0237 | 0.3298 | 0.6236 | 0.9430 |
| Lrrc61 | 48504795 | 48520721 | 0.5067 | 0.6401 | 0.1955 | 0.7389 | 0.5854 | 0.8022 | 0.3220 | 0.8883 |
| Rarres2 | 48519697 | 48522669 | 0.0284 | 0.1502 | 0.9645 | 0.9153 | 0.1554 | 0.5899 | 0.4977 | 0.9106 |
| Repin1 | 48543882 | 48549081 | 0.2642 | 0.5203 | 0.1379 | 0.7239 | 0.0055 | 0.2097 | 0.0990 | 0.8511 |
| Zfp775 | 48563179 | 48573226 | 0.0000 | **0.0000** | 0.1046 | 0.7008 | 0.1039 | 0.5038 | 0.6664 | 0.9430 |
| Gimap8 | 48597233 | 48610874 | 0.0029 | **0.0265** | 0.3016 | 0.7852 | 0.2678 | 0.6777 | 0.2580 | 0.8710 |
| Gimap9 | 48626128 | 48629113 | 0.5179 | 0.6445 | 0.5931 | 0.8716 | 0.3097 | 0.6922 | 0.6815 | 0.9430 |
| Gimap4 | 48634548 | 48642059 | 0.2221 | 0.4801 | 0.3938 | 0.8352 | 0.4827 | 0.7717 | 0.8341 | 0.9531 |
| Gimap6 | 48651581 | 48658224 | 0.1008 | 0.3311 | 0.8630 | 0.9084 | 0.1839 | 0.6336 | 0.8543 | 0.9544 |
| Gimap5 | 48668606 | 48704209 | 0.0000 | **0.0000** | 0.0761 | 0.6367 | 0.1320 | 0.5552 | 0.6714 | 0.9430 |
| Gimap7 | 48668620 | 48674635 | 0.4833 | 0.6331 | 0.2169 | 0.7525 | 0.0525 | 0.4545 | 0.6167 | 0.9413 |
| Imap38 | 48689053 | 48693789 | 0.1237 | 0.3686 | 0.5355 | 0.8653 | 0.0879 | 0.4959 | 0.1548 | 0.8710 |
| Tmem176b | 48783828 | 48790977 | 0.6349 | 0.6775 | 0.1489 | 0.7239 | 0.0751 | 0.4839 | 0.5629 | 0.9281 |
| Tmem176a | 48791508 | 48795363 | 0.3021 | 0.5473 | 0.2782 | 0.7770 | 0.8375 | 0.8348 | 0.6614 | 0.9430 |
| Abp1 | 48845253 | 48859187 | 0.1835 | 0.4425 | 0.1429 | 0.7239 | 0.9687 | 0.8567 | 0.8618 | 0.9544 |
| Gpnmb | 48986612 | 49006778 | 0.1721 | 0.4333 | 0.1816 | 0.7387 | 0.1042 | 0.5038 | 0.8151 | 0.9531 |
| 2410003K15Rik | 49023794 | 49036750 | 0.9688 | 0.7437 | 0.2393 | 0.7717 | 0.2380 | 0.6623 | 0.7756 | 0.9476 |
| Igf2bp3 | 49035220 | 49164712 | 0.1203 | 0.3661 | 0.0007 | 0.3442 | 0.0250 | 0.3411 | 0.2128 | 0.8710 |
| Tra2a | 49193920 | 49214051 | 0.0005 | **0.0052** | 0.2936 | 0.7810 | 0.4663 | 0.7606 | 0.2499 | 0.8710 |
| Ccdc126 | 49269273 | 49291581 | 0.3712 | 0.5869 | 0.6323 | 0.8758 | 0.5952 | 0.8022 | 0.0280 | 0.8252 |
| D330028D13Rik | 49317738 | 49339904 | 0.1897 | 0.4506 | 0.0493 | 0.6276 | 0.0383 | 0.3937 | 0.2041 | 0.8710 |
| Npy | 49772709 | 49779506 | 0.2375 | 0.5019 | 0.7814 | 0.9000 | 0.0585 | 0.4581 | 0.4840 | 0.9106 |
| ***DMetS6b*** | | | | | | | | | | |
| Rpn1 | 88034514 | 88053950 | 0.8030 | 0.7207 | 0.7605 | 0.8964 | 0.0690 | 0.4808 | 0.0714 | 0.8387 |
| Gata2 | 88148328 | 88156483 | 0.3646 | 0.5837 | 0.5526 | 0.8666 | 0.5571 | 0.7990 | 0.8990 | 0.9544 |
| Dnajb8 | 88172262 | 88173251 | 0.6260 | 0.6766 | 0.7545 | 0.8943 | 0.2085 | 0.6474 | 0.2642 | 0.8730 |
| Eefsec | 88207865 | 88396507 | 0.6343 | 0.6775 | 0.9456 | 0.9148 | 0.2181 | 0.6517 | 0.8757 | 0.9544 |
| Ruvbl1 | 88415403 | 88447566 | 0.1611 | 0.4201 | 0.2114 | 0.7525 | 0.6904 | 0.8083 | 0.0503 | 0.8252 |
| Sec61a1 | 88453595 | 88468899 | 0.7814 | 0.7183 | 0.7275 | 0.8937 | 0.0925 | 0.4980 | 0.0799 | 0.8387 |
| Mgll | 88674406 | 88778354 | 0.0081 | 0.0600 | 0.1721 | 0.7387 | 0.4043 | 0.7371 | 0.3452 | 0.8951 |
| Abtb1 | 88785910 | 88791860 | 0.9019 | 0.7311 | 0.0257 | 0.5912 | 0.3305 | 0.6972 | 0.8924 | 0.9544 |
| Podxl2 | 88792552 | 88825038 | 0.3806 | 0.5896 | 0.9549 | 0.9148 | 0.7484 | 0.8288 | 0.4286 | 0.9106 |
| Mcm2 | 88833469 | 88848694 | 0.1185 | 0.3635 | 0.9335 | 0.9134 | 0.0016 | 0.1193 | 0.0570 | 0.8252 |
| Tpra40 | 88852245 | 88862232 | 0.0100 | 0.0713 | 0.7701 | 0.8977 | 0.4435 | 0.7538 | 0.7072 | 0.9430 |
| Plxna1 | 89268851 | 89312592 | 0.5379 | 0.6476 | 0.4719 | 0.8426 | 0.6677 | 0.8083 | 0.8082 | 0.9531 |
| Chchd6 | 89333154 | 89545609 | 0.7620 | 0.7119 | 0.0345 | 0.5912 | 0.0059 | 0.2126 | 0.0411 | 0.8252 |
| Txnrd3 | 89593982 | 89625523 | 0.0149 | 0.0956 | 0.6093 | 0.8716 | 0.1836 | 0.6336 | 0.1931 | 0.8710 |
| V1rb9 | 89696476 | 89697408 | 0.7928 | 0.7207 | 0.6104 | 0.8716 | 0.6994 | 0.8105 | 0.4506 | 0.9106 |
| V1ra2 | 89881644 | 89890501 | 0.9041 | 0.7311 | 0.1415 | 0.7239 | 0.2858 | 0.6777 | 0.6270 | 0.9430 |
| V1rb1 | 90057269 | 90058201 | 0.6088 | 0.6720 | 0.2507 | 0.7770 | 0.4801 | 0.7700 | 0.3808 | 0.8988 |
| V1ra8 | 90152811 | 90153650 | 0.5382 | 0.6476 | 0.2356 | 0.7717 | 0.5670 | 0.7990 | 0.3846 | 0.9004 |
| V1rb3 | 90173402 | 90174334 | 0.1354 | 0.3896 | 0.5428 | 0.8653 | 0.9728 | 0.8567 | 0.9619 | 0.9592 |
| Chst13 | 90259322 | 90275179 | 0.8220 | 0.7207 | 0.0644 | 0.6276 | 0.0869 | 0.4959 | 0.1772 | 0.8710 |
| Uroc1 | 90283300 | 90314545 | 0.8274 | 0.7207 | 0.2516 | 0.7770 | 0.7120 | 0.8139 | 0.3152 | 0.8883 |
| Zxdc | 90319486 | 90353484 | 0.0600 | 0.2470 | 0.8656 | 0.9084 | 0.6969 | 0.8089 | 0.7296 | 0.9430 |
| Klf15 | 90412570 | 90425232 | 0.3844 | 0.5896 | 0.2004 | 0.7469 | 0.8903 | 0.8443 | 0.9472 | 0.9544 |
| Aldh1l1 | 90436421 | 90550197 | 0.0007 | **0.0073** | 0.0874 | 0.6688 | 0.6604 | 0.8083 | 0.3720 | 0.8971 |
| Slc41a3 | 90554719 | 90596406 | 0.3310 | 0.5630 | 0.8580 | 0.9082 | 0.0698 | 0.4815 | 0.9032 | 0.9544 |
| D6Ertd349e | 90609592 | 90714135 | 0.9342 | 0.7392 | 0.0395 | 0.5912 | 0.5800 | 0.8019 | 0.9063 | 0.9544 |
| Nup210 | 90963062 | 91066823 | 0.0165 | 0.1016 | 0.5700 | 0.8705 | 0.8911 | 0.8443 | 0.9297 | 0.9544 |
| Hdac11 | 91106659 | 91124686 | 0.8857 | 0.7278 | 0.0660 | 0.6276 | 0.5109 | 0.7775 | 0.7448 | 0.9430 |
| Fbln2 | 91162449 | 91222534 | 0.4682 | 0.6238 | 0.6685 | 0.8868 | 0.6446 | 0.8083 | 0.1919 | 0.8710 |
| Chchd4 | 91414271 | 91423417 | 0.3221 | 0.5596 | 0.2199 | 0.7525 | 0.2075 | 0.6474 | 0.5168 | 0.9218 |
| Tmem43 | 91423697 | 91438453 | 0.0557 | 0.2347 | 0.4433 | 0.8426 | 0.0021 | 0.1391 | 0.2184 | 0.8710 |
| Xpc | 91439299 | 91465878 | 0.1320 | 0.3824 | 0.4125 | 0.8380 | 0.3091 | 0.6922 | 0.0637 | 0.8379 |
| Lsm3 | 91465922 | 91472619 | 0.1768 | 0.4366 | 0.6163 | 0.8752 | 0.0017 | 0.1193 | 0.7367 | 0.9430 |
| Slc6a6 | 91634089 | 91709055 | 0.3930 | 0.5907 | 0.8342 | 0.9051 | 0.0208 | 0.3177 | 0.3642 | 0.8971 |
| ***DMetS6c*** | | | | | | | | | | |
| Il5ra | 106660378 | 106699031 | 0.8172 | 0.7207 | 0.8243 | 0.9032 | 0.8448 | 0.8348 | 0.7736 | 0.9476 |
| Crbn | 106728243 | 106750068 | 0.0086 | 0.0635 | 0.8355 | 0.9051 | 0.1492 | 0.5805 | 0.9432 | 0.9544 |
| Lrrn1 | 107479777 | 107520204 | 0.3921 | 0.5899 | 0.2930 | 0.7810 | 0.1270 | 0.5518 | 0.2038 | 0.8710 |
| Setmar | 108015039 | 108027116 | 0.0198 | 0.1170 | 0.0416 | 0.6011 | 0.9571 | 0.8548 | 0.0763 | 0.8387 |
| Itpr1 | 108163112 | 108501103 | 0.3479 | 0.5686 | 0.3196 | 0.7877 | 0.1740 | 0.6191 | 0.0357 | 0.8252 |
| Bhlhb2 | 108610623 | 108616919 | 0.3566 | 0.5750 | 0.6943 | 0.8899 | 0.6294 | 0.8083 | 0.8891 | 0.9544 |
| Arl10c | 108733076 | 108773542 | 0.3231 | 0.5600 | 0.4483 | 0.8426 | 0.5956 | 0.8022 | 0.0479 | 0.8252 |
| Edem1 | 108778635 | 108809350 | 0.5310 | 0.6465 | 0.4505 | 0.8426 | 0.8081 | 0.8348 | 0.0737 | 0.8387 |
| ***DMetS7a*** | | | | | | | | | | |
| Atp10a | 65913702 | 66084161 | 0.4678 | 0.6238 | 0.0459 | 0.6276 | 0.8520 | 0.8368 | 0.1431 | 0.8648 |
| Ube3a | 66484122 | 66562097 | 0.0437 | 0.1993 | 0.8195 | 0.9032 | 0.9440 | 0.8520 | 0.4310 | 0.9106 |
| Snurf | 67133488 | 67144657 | 0.0001 | **0.0015** | 0.0084 | 0.3723 | 0.0051 | 0.2097 | 0.3728 | 0.8971 |
| EG664849 | 67300315 | 67301328 | 0.4830 | 0.6331 | 0.4608 | 0.8426 | 0.5363 | 0.7929 | 0.7424 | 0.9430 |
| Ndn | 69493163 | 69494814 | 0.0252 | 0.1377 | 0.9209 | 0.9124 | 0.9345 | 0.8520 | 0.0609 | 0.8252 |
| Magel2 | 69524573 | 69526522 | 0.9105 | 0.7335 | 0.9502 | 0.9148 | 0.5954 | 0.8022 | 0.8594 | 0.9544 |
| Mkrn3 | 69563293 | 69564927 | 0.0479 | 0.2122 | 0.8270 | 0.9034 | 0.5621 | 0.7990 | 0.6756 | 0.9430 |
| Chrna7 | 70243578 | 70357399 | 0.8316 | 0.7207 | 0.8439 | 0.9070 | 0.4949 | 0.7719 | 0.9185 | 0.9544 |
| Klf13 | 71036253 | 71083743 | 0.0078 | 0.0594 | 0.0667 | 0.6276 | 0.4506 | 0.7543 | 0.3409 | 0.8949 |
| E030018B13Rik | 71061743 | 71065425 | 0.1499 | 0.4035 | 0.3121 | 0.7877 | 0.3073 | 0.6922 | 0.2488 | 0.8710 |
| BB128963 | 71432556 | 71485692 | 0.0860 | 0.3008 | 0.1018 | 0.7008 | 0.7644 | 0.8321 | 0.1158 | 0.8580 |
| 6030441H18Rik | 71506378 | 71518981 | 0.0589 | 0.2449 | 0.8780 | 0.9095 | 0.2609 | 0.6757 | 0.7064 | 0.9430 |
| 2810453H10Rik | 71521462 | 71537154 | 0.4980 | 0.6369 | 0.5898 | 0.8716 | 0.3404 | 0.6987 | 0.1889 | 0.8710 |
| Mcee | 71537531 | 71557007 | 0.7818 | 0.7183 | 0.1449 | 0.7239 | 0.0005 | 0.0887 | 0.3166 | 0.8883 |
| Tjp1 | 72441051 | 72516125 | 0.6211 | 0.6760 | 0.6264 | 0.8758 | 0.8288 | 0.8348 | 0.1150 | 0.8558 |
| LOC381892 | 72657935 | 72672667 | 0.0764 | 0.2881 | 0.0128 | 0.4762 | 0.0434 | 0.4119 | 0.3220 | 0.8883 |
| Tarsl2 | 72789784 | 72836977 | 0.4871 | 0.6350 | 0.5355 | 0.8653 | 0.0181 | 0.3177 | 0.1842 | 0.8710 |
| ***DMetS7b*** | | | | | | | | | | |
| Rgma | 80520406 | 80564785 | 0.8513 | 0.7207 | 0.9056 | 0.9124 | 0.8937 | 0.8444 | 0.0610 | 0.8252 |
| Chd2 | 80574494 | 80686716 | 0.0892 | 0.3054 | 0.3645 | 0.8199 | 0.0567 | 0.4581 | 0.0857 | 0.8474 |
| 1810026B05Rik | 80700038 | 80703279 | 0.7604 | 0.7119 | 0.0079 | 0.3723 | 0.8217 | 0.8348 | 0.9055 | 0.9544 |
| ***DMetS7c*** | | | | | | | | | | |
| Prc1 | 87439403 | 87461145 | 0.4455 | 0.6188 | 0.1588 | 0.7314 | 0.0069 | 0.2355 | 0.8829 | 0.9544 |
| Rccd1 | 87461482 | 87469340 | 0.0000 | **0.0000** | 0.0050 | 0.3723 | 0.3919 | 0.7263 | 0.9671 | 0.9592 |
| Unc45a | 87470178 | 87485891 | 0.0145 | 0.0933 | 0.1993 | 0.7465 | 0.0079 | 0.2509 | 0.0482 | 0.8252 |
| Man2a2 | 87505336 | 87516012 | 0.0678 | 0.2725 | 0.4130 | 0.8380 | 0.0315 | 0.3757 | 0.0317 | 0.8252 |
| Fes | 87522641 | 87532781 | 0.0347 | 0.1707 | 0.1920 | 0.7387 | 0.0861 | 0.4959 | 0.0493 | 0.8252 |
| Furin | 87533471 | 87550322 | 0.0000 | **0.0000** | 0.7562 | 0.8943 | 0.8972 | 0.8448 | 0.8780 | 0.9544 |
| Blm | 87599977 | 87679996 | 0.0013 | **0.0130** | 0.4552 | 0.8426 | 0.0008 | 0.1077 | 0.1551 | 0.8710 |
| Crtc3 | 87731513 | 87833763 | 0.1578 | 0.4142 | 0.8518 | 0.9070 | 0.1764 | 0.6231 | 0.1083 | 0.8558 |
| Iqgap1 | 87857673 | 87948180 | 0.1953 | 0.4567 | 0.0597 | 0.6276 | 0.1148 | 0.5288 | 0.6875 | 0.9430 |
| Zscan2 | 88005806 | 88021402 | 0.5630 | 0.6547 | 0.1441 | 0.7239 | 0.0797 | 0.4879 | 0.1617 | 0.8710 |
| Wdr73 | 88035609 | 88046155 | 0.5398 | 0.6476 | 0.1064 | 0.7008 | 0.1909 | 0.6355 | 0.4395 | 0.9106 |
| Nmb | 88047114 | 88049962 | 0.7970 | 0.7207 | 0.2578 | 0.7770 | 0.5523 | 0.7990 | 0.8496 | 0.9544 |
| Sec11a | 88049775 | 88092666 | 0.0636 | 0.2595 | 0.7929 | 0.9014 | 0.9553 | 0.8548 | 0.1534 | 0.8710 |
| Zfp592 | 88138598 | 88190050 | 0.8454 | 0.7207 | 0.0238 | 0.5888 | 0.0582 | 0.4581 | 0.7509 | 0.9430 |
| Alpk3 | 88202486 | 88250498 | 0.2178 | 0.4754 | 0.7488 | 0.8937 | 0.2104 | 0.6474 | 0.6735 | 0.9430 |
| Cpeb1 | 88491912 | 88600351 | 0.0311 | 0.1598 | 0.6295 | 0.8758 | 0.0237 | 0.3298 | 0.5691 | 0.9301 |
| Ap3b2 | 88605285 | 88638811 | 0.7163 | 0.7009 | 0.4459 | 0.8426 | 0.8383 | 0.8348 | 0.4003 | 0.9106 |
| BC048679 | 88639161 | 88643167 | 0.8237 | 0.7207 | 0.0089 | 0.3843 | 0.4877 | 0.7719 | 0.1947 | 0.8710 |
| Fsd2 | 88679240 | 88711867 | 0.5250 | 0.6445 | 0.4407 | 0.8426 | 0.5467 | 0.7984 | 0.7467 | 0.9430 |
| Whdc1 | 88716164 | 88741374 | 0.0000 | **0.0000** | 0.0784 | 0.6449 | 0.9453 | 0.8520 | 0.1986 | 0.8710 |
| 9330120H11Rik | 88747139 | 88747525 | 0.8029 | 0.7207 | 0.2879 | 0.7801 | 0.4580 | 0.7596 | 0.9073 | 0.9544 |
| Homer2 | 88754601 | 88851695 | 0.0001 | **0.0018** | 0.2596 | 0.7770 | 0.1645 | 0.6107 | 0.8613 | 0.9544 |
| 2610204K14Rik | 88907811 | 88914377 | 0.0060 | **0.0484** | 0.2380 | 0.7717 | 0.2653 | 0.6777 | 0.3015 | 0.8790 |
| 3110040N11Rik | 88927068 | 88934364 | 0.3496 | 0.5697 | 0.4687 | 0.8426 | 0.0657 | 0.4748 | 0.3669 | 0.8971 |
| Btbd1 | 88938404 | 88974350 | 0.0396 | 0.1890 | 0.0965 | 0.7008 | 0.1082 | 0.5100 | 0.2266 | 0.8710 |
| Tm6sf1 | 89003887 | 89029320 | 0.2339 | 0.4978 | 0.7932 | 0.9014 | 0.0599 | 0.4581 | 0.0566 | 0.8252 |
| Bnc1 | 89111717 | 89137132 | 0.4216 | 0.6031 | 0.6055 | 0.8716 | 0.1262 | 0.5518 | 0.0822 | 0.8387 |
| Sh3gl3 | 89408482 | 89455929 | 0.2540 | 0.5119 | 0.3917 | 0.8352 | 0.8373 | 0.8348 | 0.3491 | 0.8971 |
| Adamtsl3 | 89722310 | 89762956 | 0.4235 | 0.6047 | 0.4472 | 0.8426 | 0.6152 | 0.8057 | 0.0910 | 0.8474 |
| Fam154b | 89781470 | 89797038 | 0.1872 | 0.4463 | 0.3085 | 0.7877 | 0.9806 | 0.8576 | 0.9856 | 0.9621 |
| Eftud1 | 89797124 | 89926362 | 0.0000 | **0.0007** | 0.2068 | 0.7519 | 0.0197 | 0.3177 | 0.0116 | 0.8252 |
| Tmc3 | 90733441 | 90772219 | 0.4872 | 0.6350 | 0.8807 | 0.9095 | 0.6678 | 0.8083 | 0.4438 | 0.9106 |
| Stard5 | 90780469 | 90801637 | 0.0183 | 0.1099 | 0.0746 | 0.6367 | 0.7966 | 0.8348 | 0.4258 | 0.9106 |
| Il16 | 90791358 | 90894236 | 0.1982 | 0.4567 | 0.2093 | 0.7525 | 0.2970 | 0.6858 | 0.4682 | 0.9106 |
| Mesdc1 | 91028383 | 91032815 | 0.0092 | 0.0668 | 0.7528 | 0.8937 | 0.3777 | 0.7215 | 0.8354 | 0.9531 |
| Mesdc2 | 91032976 | 91050042 | 0.7492 | 0.7101 | 0.8216 | 0.9032 | 0.3185 | 0.6954 | 0.4207 | 0.9106 |
| 9930013L23Rik | 91081367 | 91235012 | 0.3753 | 0.5869 | 0.5015 | 0.8536 | 0.7877 | 0.8321 | 0.1415 | 0.8648 |
| 2210412D01Rik | 91257866 | 91300403 | 0.8744 | 0.7260 | 0.4333 | 0.8426 | 0.3849 | 0.7250 | 0.0644 | 0.8379 |
| Fah | 91733669 | 91755232 | 0.0211 | 0.1207 | 0.4806 | 0.8434 | 0.7824 | 0.8321 | 0.9967 | 0.9631 |
| Zfand6 | 91763564 | 91827861 | 0.0013 | **0.0131** | 0.8451 | 0.9070 | 0.1333 | 0.5552 | 0.7498 | 0.9430 |
| ***DMetS8a*** | | | | | | | | | | |
| EG574083 | 22056169 | 22061750 | 0.6825 | 0.6933 | 0.8728 | 0.9095 | 0.3203 | 0.6954 | 0.1764 | 0.8710 |
| Defcr21 | 22165224 | 22166195 | 0.3024 | 0.5473 | 0.4882 | 0.8470 | 0.0788 | 0.4879 | 0.7559 | 0.9444 |
| Defcr-rs7 | 22331293 | 22332228 | 0.0092 | 0.0667 | 0.4016 | 0.8362 | 0.5690 | 0.7990 | 0.5926 | 0.9399 |
| Defcr-rs1 | 22465566 | 22466699 | 0.1324 | 0.3826 | 0.3150 | 0.7877 | 0.0189 | 0.3177 | 0.4611 | 0.9106 |
| Defcr20 | 22619730 | 22620709 | 0.1370 | 0.3906 | 0.1273 | 0.7239 | 0.1213 | 0.5427 | 0.0263 | 0.8252 |
| AY761184 | 22812991 | 22814117 | 0.5247 | 0.6445 | 0.4142 | 0.8380 | 0.8409 | 0.8348 | 0.1480 | 0.8710 |
| Defb1 | 22887071 | 22905657 | 0.3437 | 0.5686 | 0.3121 | 0.7877 | 0.3610 | 0.7093 | 0.7420 | 0.9430 |
| Defb15 | 23040271 | 23043182 | 0.7753 | 0.7175 | 0.8800 | 0.9095 | 0.3211 | 0.6954 | 0.8582 | 0.9544 |
| Ccdc70 | 23080247 | 23084513 | 0.4008 | 0.5956 | 0.5852 | 0.8716 | 0.3226 | 0.6957 | 0.0168 | 0.8252 |
| Atp7b | 23103257 | 23170777 | 0.0171 | 0.1046 | 0.1257 | 0.7239 | 0.9567 | 0.8548 | 0.5285 | 0.9248 |
| Alg11 | 23171193 | 23182099 | 0.3715 | 0.5869 | 0.9333 | 0.9134 | 0.7541 | 0.8295 | 0.6861 | 0.9430 |
| Nek3 | 23238755 | 23276907 | 0.1622 | 0.4203 | 0.2080 | 0.7519 | 0.0347 | 0.3816 | 0.1864 | 0.8710 |
| Ckap2 | 23278921 | 23296291 | 0.0052 | **0.0431** | 0.4323 | 0.8426 | 0.0014 | 0.1171 | 0.9042 | 0.9544 |
| Vps36 | 23303281 | 23331315 | 0.8843 | 0.7278 | 0.7733 | 0.8979 | 0.4674 | 0.7606 | 0.3845 | 0.9004 |
| Thsd1 | 23337785 | 23371806 | 0.6955 | 0.6962 | 0.3759 | 0.8268 | 0.1076 | 0.5100 | 0.0811 | 0.8387 |
| Slc25a15 | 23486479 | 23509025 | 0.0558 | 0.2347 | 0.2392 | 0.7717 | 0.0613 | 0.4581 | 0.1902 | 0.8710 |
| Mrps31 | 23521913 | 23539945 | 0.1152 | 0.3560 | 0.7832 | 0.9000 | 0.6880 | 0.8083 | 0.0736 | 0.8387 |
| AI316807 | 23573095 | 23587140 | 0.0050 | **0.0421** | 0.0294 | 0.5912 | 0.1388 | 0.5599 | 0.9930 | 0.9623 |
| Slc20a2 | 23587260 | 23680079 | 0.6755 | 0.6913 | 0.6934 | 0.8899 | 0.8913 | 0.8443 | 0.3800 | 0.8986 |
| Vdac3 | 23687573 | 23704230 | 0.4095 | 0.5970 | 0.8280 | 0.9035 | 0.3326 | 0.6972 | 0.2897 | 0.8778 |
| Dkk4 | 23734515 | 23738008 | 0.6491 | 0.6838 | 0.4889 | 0.8470 | 0.5542 | 0.7990 | 0.6429 | 0.9430 |
| Polb | 23738737 | 23763886 | 0.6684 | 0.6881 | 0.7097 | 0.8905 | 0.9134 | 0.8492 | 0.7514 | 0.9430 |
| Ikbkb | 23769684 | 23817061 | 0.9812 | 0.7474 | 0.0727 | 0.6367 | 0.2455 | 0.6728 | 0.0549 | 0.8252 |
| Plat | 23868240 | 23893316 | 0.3999 | 0.5956 | 0.2201 | 0.7525 | 0.8386 | 0.8348 | 0.2838 | 0.8778 |
| Ap3m2 | 23897827 | 23916099 | 0.3902 | 0.5896 | 0.4353 | 0.8426 | 0.0001 | 0.0849 | 0.5604 | 0.9281 |
| 1700041G16Rik | 23918747 | 23919204 | 0.1756 | 0.4354 | 0.3993 | 0.8352 | 0.4678 | 0.7606 | 0.7045 | 0.9430 |
| Myst3 | 23970007 | 24053731 | 0.8441 | 0.7207 | 0.8750 | 0.9095 | 0.8076 | 0.8348 | 0.8322 | 0.9531 |
| Ank1 | 24085294 | 24260969 | 0.4210 | 0.6031 | 0.2967 | 0.7822 | 0.4107 | 0.7390 | 0.5681 | 0.9298 |
| Agpat6 | 24284207 | 24318833 | 0.0435 | 0.1993 | 0.9007 | 0.9124 | 0.7292 | 0.8216 | 0.5887 | 0.9374 |
| Gins4 | 24337097 | 24348131 | 0.7179 | 0.7009 | 0.4142 | 0.8380 | 0.0095 | 0.2671 | 0.5566 | 0.9281 |
| Golga7 | 24351825 | 24367546 | 0.6656 | 0.6881 | 0.3150 | 0.7877 | 0.3345 | 0.6972 | 0.4931 | 0.9106 |
| Sfrp1 | 24521974 | 24560104 | 0.8232 | 0.7207 | 0.1927 | 0.7387 | 0.8146 | 0.8348 | 0.1564 | 0.8710 |
| 1810011O10Rik | 25549048 | 25549368 | 0.0128 | 0.0860 | 0.5307 | 0.8652 | 0.0380 | 0.3937 | 0.2493 | 0.8710 |
| Indol1 | 25642366 | 25686805 | 0.0061 | **0.0484** | 0.2237 | 0.7568 | 0.1309 | 0.5552 | 0.7921 | 0.9518 |
| Indo | 25694608 | 25707481 | 0.1370 | 0.3906 | 0.2721 | 0.7770 | 0.0568 | 0.4581 | 0.7715 | 0.9476 |
| Adam3 | 25787706 | 25836297 | 0.3882 | 0.5896 | 0.5668 | 0.8688 | 0.4899 | 0.7719 | 0.8210 | 0.9531 |
| Adam5 | 25837565 | 25934841 | 0.4554 | 0.6188 | 0.2275 | 0.7597 | 0.2560 | 0.6728 | 0.9135 | 0.9544 |
| Adam32 | 25946615 | 26059276 | 0.0780 | 0.2888 | 0.0328 | 0.5912 | 0.3812 | 0.7234 | 0.9666 | 0.9592 |
| Adam9 | 26060097 | 26127266 | 0.3182 | 0.5563 | 0.6705 | 0.8868 | 0.2784 | 0.6777 | 0.1793 | 0.8710 |
| Tm2d2 | 26127752 | 26133731 | 0.3459 | 0.5686 | 0.0597 | 0.6276 | 0.4958 | 0.7719 | 0.4673 | 0.9106 |
| Htra4 | 26135400 | 26149434 | 0.0048 | **0.0410** | 0.4270 | 0.8423 | 0.0435 | 0.4119 | 0.1466 | 0.8710 |
| Plekha2 | 26149616 | 26212666 | 0.2611 | 0.5173 | 0.1110 | 0.7061 | 0.0578 | 0.4581 | 0.6267 | 0.9430 |
| Tacc1 | 26265024 | 26311921 | 0.0252 | 0.1377 | 0.9868 | 0.9207 | 0.5711 | 0.7990 | 0.4648 | 0.9106 |
| Fgfr1 | 26624126 | 26685253 | 0.6509 | 0.6838 | 0.7189 | 0.8905 | 0.9557 | 0.8548 | 0.8247 | 0.9531 |
| D030041N04Rik | 26702132 | 26707968 | 0.6889 | 0.6944 | 0.1067 | 0.7008 | 0.3631 | 0.7093 | 0.9340 | 0.9544 |
| Whsc1l1 | 26712073 | 26830139 | 0.5206 | 0.6445 | 0.2480 | 0.7770 | 0.7618 | 0.8321 | 0.1572 | 0.8710 |
| Ppapdc1b | 26830533 | 26835359 | 0.3754 | 0.5869 | 0.4964 | 0.8524 | 0.6662 | 0.8083 | 0.2279 | 0.8710 |
| Ddhd2 | 26835821 | 26864672 | 0.0983 | 0.3253 | 0.7077 | 0.8905 | 0.9985 | 0.8591 | 0.9594 | 0.9590 |
| Bag4 | 26877669 | 26895674 | 0.2340 | 0.4978 | 0.6879 | 0.8899 | 0.2709 | 0.6777 | 0.2065 | 0.8710 |
| Lsm1 | 26895780 | 26913003 | 0.3083 | 0.5512 | 0.7888 | 0.9000 | 0.3200 | 0.6954 | 0.8955 | 0.9544 |
| Ash2l | 26926663 | 26958166 | 0.3538 | 0.5727 | 0.5793 | 0.8716 | 0.0237 | 0.3298 | 0.5267 | 0.9248 |
| Hgsnat | 27054931 | 27087216 | 0.0001 | **0.0009** | 0.8793 | 0.9095 | 0.6475 | 0.8083 | 0.0619 | 0.8310 |
| 4930444A02Rik | 27091076 | 27104593 | 0.4444 | 0.6188 | 0.4732 | 0.8426 | 0.5555 | 0.7990 | 0.1833 | 0.8710 |
| Fnta | 27109446 | 27126070 | 0.8803 | 0.7271 | 0.3137 | 0.7877 | 0.8313 | 0.8348 | 0.2000 | 0.8710 |
| Hook3 | 27131893 | 27229696 | 0.3767 | 0.5876 | 0.2808 | 0.7770 | 0.0011 | 0.1077 | 0.7023 | 0.9430 |
| 6720407G21Rik | 27229840 | 27254343 | 0.8236 | 0.7207 | 0.3577 | 0.8148 | 0.0705 | 0.4815 | 0.8340 | 0.9531 |
| Rnf170 | 27229840 | 27254343 | 0.1062 | 0.3406 | 0.2907 | 0.7810 | 0.8191 | 0.8348 | 0.6594 | 0.9430 |
| Thap1 | 27268684 | 27273412 | 0.3260 | 0.5630 | 0.4639 | 0.8426 | 0.3191 | 0.6954 | 0.8681 | 0.9544 |
| Zfp703 | 28087826 | 28091938 | 0.6052 | 0.6703 | 0.6946 | 0.8899 | 0.1991 | 0.6407 | 0.8773 | 0.9544 |
| Erlin2 | 28134331 | 28149896 | 0.0001 | **0.0008** | 0.0709 | 0.6319 | 0.2491 | 0.6728 | 0.4272 | 0.9106 |
| Prosc | 28153075 | 28165038 | 0.0000 | **0.0000** | 0.0206 | 0.5888 | 0.0478 | 0.4340 | 0.1104 | 0.8558 |
| Gpr124 | 28196313 | 28233890 | 0.2565 | 0.5139 | 0.0629 | 0.6276 | 0.2340 | 0.6585 | 0.0922 | 0.8474 |
| Brf2 | 28234304 | 28239104 | 0.0904 | 0.3066 | 0.0103 | 0.4120 | 0.8495 | 0.8355 | 0.5184 | 0.9223 |
| Adrb3 | 28336248 | 28340060 | 0.0004 | **0.0046** | 0.5931 | 0.8716 | 0.3671 | 0.7108 | 0.1338 | 0.8627 |
| Eif4ebp1 | 28370850 | 28385985 | 0.9351 | 0.7395 | 0.0043 | 0.3723 | 0.1748 | 0.6204 | 0.0889 | 0.8474 |
| Gm9731 | 28406517 | 28407672 | 0.0008 | **0.0089** | 0.5642 | 0.8666 | 0.6431 | 0.8083 | 0.4869 | 0.9106 |
| Chrnb3 | 28479183 | 28510201 | 0.2376 | 0.5019 | 0.2436 | 0.7770 | 0.4690 | 0.7606 | 0.0601 | 0.8252 |
| ***DMetS8b*** | | | | | | | | | | |
| Rnf150 | 85387255 | 85608713 | 0.0321 | 0.1617 | 0.1646 | 0.7345 | 0.6049 | 0.8022 | 0.4525 | 0.9106 |
| Tbc1d9 | 85689251 | 85796833 | 0.0106 | 0.0750 | 0.6727 | 0.8868 | 0.7605 | 0.8321 | 0.0019 | 0.4619 |
| Ucp1 | 85814261 | 85821951 | 0.8357 | 0.7207 | 0.3421 | 0.8017 | 0.3964 | 0.7305 | 0.2033 | 0.8710 |
| Elmod2 | 85836921 | 85856385 | 0.5223 | 0.6445 | 0.6926 | 0.8899 | 0.3323 | 0.6972 | 0.1872 | 0.8710 |
| Clgn | 85913766 | 85952451 | 0.0893 | 0.3054 | 0.5254 | 0.8652 | 0.5326 | 0.7919 | 0.0935 | 0.8474 |
| Scoc | 85958392 | 85982290 | 0.0012 | **0.0125** | 0.2132 | 0.7525 | 0.0200 | 0.3177 | 0.1647 | 0.8710 |
| Ndufb7 | 86090570 | 86095525 | 0.0940 | 0.3149 | 0.0029 | 0.3723 | 0.8433 | 0.8348 | 0.8910 | 0.9544 |
| Gpsn2 | 86095599 | 86118361 | 0.0000 | **0.0000** | 0.3164 | 0.7877 | 0.3368 | 0.6972 | 0.8736 | 0.9544 |
| Dnajb1 | 86132172 | 86135801 | 0.4027 | 0.5959 | 0.9524 | 0.9148 | 0.7752 | 0.8321 | 0.9998 | 0.9631 |
| Gipc1 | 86176597 | 86188580 | 0.0022 | **0.0210** | 0.0021 | 0.3576 | 0.1528 | 0.5887 | 0.7284 | 0.9430 |
| Pkn1 | 86193661 | 86223078 | 0.0000 | **0.0005** | 0.7573 | 0.8943 | 0.4503 | 0.7543 | 0.4905 | 0.9106 |
| Cd97 | 86247150 | 86265225 | 0.0429 | 0.1982 | 0.3152 | 0.7877 | 0.0049 | 0.2097 | 0.1837 | 0.8710 |
| Lphn1 | 86424004 | 86465853 | 0.2202 | 0.4775 | 0.1242 | 0.7237 | 0.4998 | 0.7733 | 0.4776 | 0.9106 |
| Asf1b | 86479406 | 86494096 | 0.3725 | 0.5869 | 0.6190 | 0.8758 | 0.0004 | 0.0849 | 0.4533 | 0.9106 |
| Prkaca | 86496887 | 86520334 | 0.1974 | 0.4567 | 0.1006 | 0.7008 | 0.1016 | 0.5035 | 0.0379 | 0.8252 |
| Samd1 | 86521571 | 86524285 | 0.3828 | 0.5896 | 0.8568 | 0.9082 | 0.0743 | 0.4823 | 0.6135 | 0.9408 |
| 1700067K01Rik | 86525422 | 86528669 | 0.0000 | **0.0001** | 0.4724 | 0.8426 | 0.6636 | 0.8083 | 0.2501 | 0.8710 |
| 4432412L15Rik | 86545370 | 86554194 | 0.9810 | 0.7474 | 0.3555 | 0.8131 | 0.5106 | 0.7775 | 0.6644 | 0.9430 |
| Il27ra | 86554217 | 86566474 | 0.5874 | 0.6641 | 0.3459 | 0.8033 | 0.3155 | 0.6932 | 0.4097 | 0.9106 |
| Rln3 | 86566966 | 86568878 | 0.5986 | 0.6668 | 0.3313 | 0.7948 | 0.3250 | 0.6972 | 0.2526 | 0.8710 |
| Rfx1 | 86590765 | 86620901 | 0.0000 | **0.0006** | 0.2013 | 0.7483 | 0.0287 | 0.3631 | 0.9126 | 0.9544 |
| BC057552 | 86620971 | 86628661 | 0.5679 | 0.6560 | 0.2706 | 0.7770 | 0.2307 | 0.6584 | 0.8994 | 0.9544 |
| Podnl1 | 86649888 | 86656426 | 0.1225 | 0.3676 | 0.5915 | 0.8716 | 0.9922 | 0.8591 | 0.6235 | 0.9430 |
| Cc2d1a | 86656727 | 86671796 | 0.4969 | 0.6366 | 0.3803 | 0.8291 | 0.3451 | 0.6990 | 0.5790 | 0.9349 |
| Zswim4 | 86735845 | 86760954 | 0.6670 | 0.6881 | 0.2825 | 0.7770 | 0.0514 | 0.4504 | 0.5213 | 0.9248 |
| D8Ertd738e | 86770143 | 86773609 | 0.2777 | 0.5297 | 0.5384 | 0.8653 | 0.4351 | 0.7531 | 0.8724 | 0.9544 |
| Ccdc130 | 86781694 | 86794259 | 0.0000 | **0.0000** | 0.5119 | 0.8636 | 0.1927 | 0.6355 | 0.5948 | 0.9401 |
| Cacna1a | 86912339 | 87164145 | 0.0004 | **0.0045** | 0.1880 | 0.7387 | 0.1084 | 0.5100 | 0.8990 | 0.9544 |
| ***DMetS10a*** | | | | | | | | | | |
| Btg1 | 96079661 | 96082261 | 0.0700 | 0.2765 | 0.3269 | 0.7917 | 0.5416 | 0.7947 | 0.4693 | 0.9106 |
| Dcn | 96945001 | 96980785 | 0.0000 | **0.0003** | 0.1306 | 0.7239 | 0.7337 | 0.8224 | 0.9775 | 0.9614 |
| Lum | 97028464 | 97035338 | 0.0000 | **0.0004** | 0.0811 | 0.6483 | 0.3879 | 0.7263 | 0.9049 | 0.9544 |
| Atp2b1 | 98377804 | 98486420 | 0.6929 | 0.6959 | 0.2282 | 0.7597 | 0.4928 | 0.7719 | 0.0965 | 0.8511 |
| Wdr51b | 98569670 | 98660708 | 0.1022 | 0.3332 | 0.6464 | 0.8808 | 0.8340 | 0.8348 | 0.4471 | 0.9106 |
| Galnt4 | 98570793 | 98575881 | 0.0199 | 0.1170 | 0.5580 | 0.8666 | 0.7279 | 0.8216 | 0.4982 | 0.9106 |
| Dusp6 | 98725865 | 98730118 | 0.1471 | 0.3994 | 0.5336 | 0.8653 | 0.7523 | 0.8288 | 0.5621 | 0.9281 |
| B530045E10Rik | 98883156 | 98885681 | 0.6592 | 0.6881 | 0.7261 | 0.8928 | 0.4322 | 0.7531 | 0.4090 | 0.9106 |
| Csl | 99220435 | 99221835 | 0.0527 | 0.2268 | 0.3749 | 0.8268 | 0.9637 | 0.8566 | 0.0235 | 0.8252 |
| ***DMetS10b*** | | | | | | | | | | |
| 4930430F08Rik | 100034969 | 100051876 | 0.0989 | 0.3264 | 0.7132 | 0.8905 | 0.3857 | 0.7252 | 0.0241 | 0.8252 |
| Trhde | 113835879 | 114239363 | 0.0594 | 0.2462 | 0.0381 | 0.5912 | 0.1546 | 0.5899 | 0.4405 | 0.9106 |
| Tbc1d15 | 114635514 | 114688521 | 0.0198 | 0.1170 | 0.2132 | 0.7525 | 0.2867 | 0.6777 | 0.4746 | 0.9106 |
| Rab21 | 114726919 | 114752647 | 0.0132 | 0.0875 | 0.1337 | 0.7239 | 0.6669 | 0.8083 | 0.0340 | 0.8252 |
| Tmem19 | 114777795 | 114799318 | 0.0000 | **0.0006** | 0.7456 | 0.8937 | 0.6160 | 0.8057 | 0.5400 | 0.9267 |
| Zfc3h1 | 114822015 | 114869828 | 0.8532 | 0.7207 | 0.0388 | 0.5912 | 0.0920 | 0.4980 | 0.6571 | 0.9430 |
| Lgr5 | 114887367 | 115024836 | 0.2422 | 0.5020 | 0.8467 | 0.9070 | 0.0923 | 0.4980 | 0.9763 | 0.9614 |
| Tspan8 | 115254340 | 115286672 | 0.0042 | **0.0359** | 0.4728 | 0.8426 | 0.4171 | 0.7446 | 0.7124 | 0.9430 |
| Ptprr | 115455269 | 115711988 | 0.4285 | 0.6074 | 0.6081 | 0.8716 | 0.3736 | 0.7187 | 0.7481 | 0.9430 |
| 4933416C03Rik | 115549539 | 115550675 | 0.5728 | 0.6563 | 0.8666 | 0.9084 | 0.9277 | 0.8520 | 0.8610 | 0.9544 |
| 3230402H02Rik | 115738562 | 115820989 | 0.0000 | **0.0000** | 0.8439 | 0.9070 | 0.7364 | 0.8226 | 0.3421 | 0.8949 |
| Ptprb | 115738562 | 115820989 | 0.0000 | **0.0000** | 0.9002 | 0.9124 | 0.2164 | 0.6517 | 0.1614 | 0.8710 |
| Kcnmb4 | 115854924 | 115910579 | 0.8915 | 0.7291 | 0.2555 | 0.7770 | 0.6163 | 0.8057 | 0.5813 | 0.9349 |
| Cnot2 | 115922222 | 116018557 | 0.0001 | **0.0013** | 0.5609 | 0.8666 | 0.0002 | 0.0849 | 0.3211 | 0.8883 |
| 5330438D12Rik | 116018540 | 116019211 | 0.0000 | **0.0000** | 0.0217 | 0.5888 | 0.0082 | 0.2509 | 0.7304 | 0.9430 |
| Gm239 | 116213601 | 116333935 | 0.7888 | 0.7198 | 0.7186 | 0.8905 | 0.2532 | 0.6728 | 0.9934 | 0.9623 |
| Rab3ip | 116343833 | 116387487 | 0.0000 | **0.0007** | 0.0014 | 0.3442 | 0.6897 | 0.8083 | 0.0446 | 0.8252 |
| Cct2 | 116488059 | 116500834 | 0.4565 | 0.6188 | 0.8298 | 0.9044 | 0.7307 | 0.8216 | 0.6148 | 0.9408 |
| Frs2 | 116507185 | 116585530 | 0.0004 | **0.0047** | 0.0697 | 0.6276 | 0.0322 | 0.3769 | 0.9085 | 0.9544 |
| Yeats4 | 116652277 | 116661546 | 0.0000 | **0.0000** | 0.7672 | 0.8977 | 0.1015 | 0.5035 | 0.4685 | 0.9106 |
| Lyzs | 116714389 | 116719320 | 0.0035 | **0.0305** | 0.4467 | 0.8426 | 0.3341 | 0.6972 | 0.2912 | 0.8778 |
| Lyz2 | 116714389 | 116719320 | 0.5773 | 0.6567 | 0.1083 | 0.7008 | 0.0011 | 0.1077 | 0.9479 | 0.9544 |
| Cpsf6 | 116792994 | 116814029 | 0.0000 | **0.0000** | 0.6343 | 0.8758 | 0.8667 | 0.8369 | 0.5531 | 0.9281 |
| ***DMetS14a*** | | | | | | | | | | |
| 1700112E06Rik | 22838934 | 23875307 | 0.8411 | 0.7207 | 0.8025 | 0.9032 | 0.7109 | 0.8139 | 0.6453 | 0.9430 |
| Gm7480 | 22851305 | 22855325 | 0.3511 | 0.5697 | 0.9500 | 0.9148 | 0.7928 | 0.8338 | 0.4904 | 0.9106 |
| Dlg5 | 24953177 | 25065142 | 0.1753 | 0.4354 | 0.7957 | 0.9032 | 0.5782 | 0.8019 | 0.7908 | 0.9517 |
| E330034G19Rik | 25112436 | 25129188 | 0.9796 | 0.7474 | 0.6967 | 0.8899 | 0.9802 | 0.8576 | 0.4994 | 0.9106 |
| Polr3a | 25267916 | 25306268 | 0.0268 | 0.1438 | 0.3633 | 0.8199 | 0.6431 | 0.8083 | 0.8098 | 0.9531 |
| Rps24 | 25309940 | 25315067 | 0.1799 | 0.4414 | 0.9706 | 0.9159 | 0.0722 | 0.4823 | 0.9471 | 0.9544 |
| D930049A15Rik | 26275223 | 26280614 | 0.6771 | 0.6918 | 0.7896 | 0.9000 | 0.2021 | 0.6407 | 0.0921 | 0.8474 |
| Zmiz1 | 26278671 | 26486229 | 0.5896 | 0.6661 | 0.1150 | 0.7079 | 0.2075 | 0.6474 | 0.6609 | 0.9430 |
| 4931406H21Rik | 26406290 | 26410147 | 0.0524 | 0.2264 | 0.7703 | 0.8977 | 0.1707 | 0.6190 | 0.4810 | 0.9106 |
| Ppif | 26513640 | 26519954 | 0.3283 | 0.5630 | 0.4295 | 0.8426 | 0.8626 | 0.8369 | 0.9239 | 0.9544 |
| Zcchc24 | 26531126 | 26588342 | 0.0041 | **0.0354** | 0.1872 | 0.7387 | 0.2738 | 0.6777 | 0.9883 | 0.9621 |
| Anxa11 | 26661669 | 26706290 | 0.1341 | 0.3866 | 0.3344 | 0.7975 | 0.9589 | 0.8551 | 0.6630 | 0.9430 |
| Gm9746 | 26743656 | 26746696 | 0.3722 | 0.5869 | 0.1656 | 0.7345 | 0.9310 | 0.8520 | 0.8734 | 0.9544 |
| Cphx | 26762030 | 26775851 | 0.1291 | 0.3764 | 0.9876 | 0.9207 | 0.4707 | 0.7606 | 0.5766 | 0.9346 |
| 4933413J09Rik | 27176060 | 27219942 | 0.6114 | 0.6721 | 0.0535 | 0.6276 | 0.9215 | 0.8520 | 0.9172 | 0.9544 |
| Slmap | 27232654 | 27354417 | 0.3002 | 0.5459 | 0.6232 | 0.8758 | 0.3261 | 0.6972 | 0.3698 | 0.8971 |
| A630054L15Rik | 27399047 | 27447895 | 0.7478 | 0.7096 | 0.7720 | 0.8977 | 0.2640 | 0.6777 | 0.4497 | 0.9106 |
| E430028B21Rik | 27484507 | 27489144 | 0.1005 | 0.3308 | 0.9596 | 0.9151 | 0.6039 | 0.8022 | 0.5345 | 0.9248 |
| Asb14 | 27707798 | 27728441 | 0.2259 | 0.4845 | 0.6644 | 0.8868 | 0.1009 | 0.5035 | 0.3842 | 0.9004 |
| 2900057D21Rik | 27732174 | 27784418 | 0.0003 | **0.0038** | 0.0787 | 0.6449 | 0.0371 | 0.3901 | 0.4926 | 0.9106 |
| Il17rd | 27852187 | 27920472 | 0.1071 | 0.3419 | 0.6421 | 0.8795 | 0.2512 | 0.6728 | 0.2062 | 0.8710 |
| Arhgef3 | 28051225 | 28217090 | 0.0010 | **0.0101** | 0.0691 | 0.6276 | 0.5658 | 0.7990 | 0.0092 | 0.8252 |
| D14Abb1e | 28242033 | 28294574 | 0.3164 | 0.5560 | 0.8906 | 0.9098 | 0.5478 | 0.7990 | 0.4981 | 0.9106 |
| Ccdc66 | 28295598 | 28321646 | 0.6797 | 0.6920 | 0.2166 | 0.7525 | 0.2491 | 0.6728 | 0.9862 | 0.9621 |
| Erc2 | 28435628 | 29291723 | 0.0007 | **0.0073** | 0.1074 | 0.7008 | 0.7294 | 0.8216 | 0.5481 | 0.9281 |
| ***DMetS15a*** | | | | | | | | | | |
| Phf20l1 | 66409134 | 66476817 | 0.2525 | 0.5115 | 0.3382 | 0.7975 | 0.4891 | 0.7719 | 0.5867 | 0.9366 |
| Tg | 66502332 | 66682275 | 0.0781 | 0.2888 | 0.2557 | 0.7770 | 0.1365 | 0.5573 | 0.4454 | 0.9106 |
| Sla | 66612434 | 66644284 | 0.0310 | 0.1598 | 0.1752 | 0.7387 | 0.2576 | 0.6728 | 0.9235 | 0.9544 |
| Wisp1 | 66722882 | 66754763 | 0.2243 | 0.4834 | 0.5616 | 0.8666 | 0.2322 | 0.6585 | 0.1727 | 0.8710 |
| Ndrl | 66760880 | 66801203 | 0.3261 | 0.5630 | 0.4553 | 0.8426 | 0.8706 | 0.8369 | 0.1675 | 0.8710 |
| St3gal1 | 66934437 | 67008392 | 0.0000 | **0.0000** | 0.3062 | 0.7852 | 0.3429 | 0.6990 | 0.5934 | 0.9399 |
| Zfat | 67915328 | 68090418 | 0.9382 | 0.7406 | 0.1893 | 0.7387 | 0.2468 | 0.6728 | 0.3644 | 0.8971 |
| ***DMetS16a*** | | | | | | | | | | |
| Nfkbiz | 55811488 | 55839012 | 0.1428 | 0.3965 | 0.0236 | 0.5888 | 0.6072 | 0.8022 | 0.5010 | 0.9106 |
| Gm1752 | 55843318 | 55866498 | 0.1253 | 0.3707 | 0.3171 | 0.7877 | 0.1503 | 0.5834 | 0.6000 | 0.9408 |
| Lrriq2 | 55900001 | 55934968 | 0.0000 | **0.0000** | 0.0818 | 0.6487 | 0.1363 | 0.5573 | 0.1295 | 0.8627 |
| Rpl24 | 55966388 | 55980835 | 0.9663 | 0.7437 | 0.2223 | 0.7561 | 0.7743 | 0.8321 | 0.4110 | 0.9106 |
| Zbtb11 | 55989079 | 56006355 | 0.4462 | 0.6188 | 0.1279 | 0.7239 | 0.2111 | 0.6474 | 0.0683 | 0.8387 |
| Pcnp | 56007358 | 56029852 | 0.0000 | **0.0000** | 0.7176 | 0.8905 | 0.5901 | 0.8022 | 0.6888 | 0.9430 |
| Rg9mtd1 | 56032722 | 56037932 | 0.0000 | **0.0000** | 0.9392 | 0.9139 | 0.2314 | 0.6584 | 0.0065 | 0.8252 |
| Senp7 | 56075522 | 56190124 | 0.0001 | **0.0009** | 0.0587 | 0.6276 | 0.4354 | 0.7531 | 0.6469 | 0.9430 |
| Impg2 | 56204426 | 56273869 | 0.0070 | 0.0542 | 0.3711 | 0.8268 | 0.5292 | 0.7909 | 0.8001 | 0.9531 |
| Abi3bp | 56478013 | 56689430 | 0.0000 | **0.0000** | 0.1758 | 0.7387 | 0.2836 | 0.6777 | 0.6772 | 0.9430 |
| Tfg | 56690445 | 56717563 | 0.0000 | **0.0003** | 0.1460 | 0.7239 | 0.1030 | 0.5038 | 0.1503 | 0.8710 |
| Gpr128 | 56724722 | 56795968 | 0.2655 | 0.5204 | 0.8516 | 0.9070 | 0.2189 | 0.6517 | 0.4003 | 0.9106 |
| Tmem45a | 56805274 | 56886279 | 0.5455 | 0.6476 | 0.0517 | 0.6276 | 0.3092 | 0.6922 | 0.8334 | 0.9531 |
| 2310005G13Rik | 57038793 | 57049695 | 0.2673 | 0.5212 | 0.2473 | 0.7770 | 0.6074 | 0.8022 | 0.2351 | 0.8710 |
| Tomm70a | 57121827 | 57154643 | 0.1694 | 0.4311 | 0.2229 | 0.7561 | 0.7465 | 0.8282 | 0.9094 | 0.9544 |
| Nit2 | 57157007 | 57167428 | 0.0000 | **0.0000** | 0.4591 | 0.8426 | 0.3310 | 0.6972 | 0.7627 | 0.9476 |
| Tbc1d23 | 57168979 | 57231579 | 0.5280 | 0.6458 | 0.3000 | 0.7852 | 0.6921 | 0.8083 | 0.0531 | 0.8252 |
| 2610528E23Rik | 57302113 | 57606956 | 0.0000 | **0.0000** | 0.1059 | 0.7008 | 0.0316 | 0.3757 | 0.0910 | 0.8474 |
| 4631422O05Rik | 57353206 | 57573239 | 0.2084 | 0.4646 | 0.4234 | 0.8392 | 0.4002 | 0.7323 | 0.5112 | 0.9189 |
| Col8a1 | 57624371 | 57754850 | 0.0026 | **0.0245** | 0.4977 | 0.8524 | 0.0107 | 0.2830 | 0.0035 | 0.5944 |
| St3gal6 | 58468238 | 58524356 | 0.1449 | 0.3972 | 0.1262 | 0.7239 | 0.3435 | 0.6990 | 0.8528 | 0.9544 |
| E330017A01Rik | 58635375 | 58638516 | 0.1462 | 0.3987 | 0.6321 | 0.8758 | 0.4187 | 0.7457 | 0.7812 | 0.9476 |
| Cpox | 58670405 | 58680474 | 0.0003 | **0.0040** | 0.3047 | 0.7852 | 0.3250 | 0.6972 | 0.7365 | 0.9430 |
| Gpr15 | 58717755 | 58718837 | 0.9421 | 0.7409 | 0.6634 | 0.8868 | 0.1917 | 0.6355 | 0.2474 | 0.8710 |
| Cldnd1 | 58728023 | 58734364 | 0.7467 | 0.7092 | 0.4423 | 0.8426 | 0.5388 | 0.7942 | 0.2863 | 0.8778 |
| Olfr173 | 58796675 | 58797640 | 0.4116 | 0.5980 | 0.8954 | 0.9117 | 0.5593 | 0.7990 | 0.0279 | 0.8252 |
| Olfr187 | 59035635 | 59036561 | 0.9407 | 0.7408 | 0.8992 | 0.9124 | 0.7893 | 0.8321 | 0.7997 | 0.9531 |
| Olfr192 | 59097892 | 59098816 | 0.2770 | 0.5291 | 0.2201 | 0.7525 | 0.2335 | 0.6585 | 0.5055 | 0.9144 |
| Olfr194 | 59118974 | 59119894 | 0.9661 | 0.7437 | 0.4039 | 0.8362 | 0.2384 | 0.6623 | 0.2923 | 0.8778 |
| Olfr198 | 59201330 | 59202250 | 0.9448 | 0.7409 | 0.8604 | 0.9082 | 0.0864 | 0.4959 | 0.9395 | 0.9544 |
| ***DMetS17a*** | | | | | | | | | | |
| Zfp213 | 23693737 | 23701096 | 0.0000 | **0.0000** | 0.5273 | 0.8652 | 0.1538 | 0.5899 | 0.9444 | 0.9544 |
| Zfp13 | 23712819 | 23736454 | 0.4064 | 0.5970 | 0.6420 | 0.8795 | 0.3110 | 0.6922 | 0.3379 | 0.8930 |
| BC038613 | 23797487 | 23805577 | 0.2869 | 0.5350 | 0.7803 | 0.9000 | 0.4890 | 0.7719 | 0.0491 | 0.8252 |
| Thoc6 | 23805581 | 23810849 | 0.0172 | 0.1046 | 0.6992 | 0.8905 | 0.4893 | 0.7719 | 0.8557 | 0.9544 |
| Hcfc1r1 | 23810924 | 23812192 | 0.6232 | 0.6761 | 0.0564 | 0.6276 | 0.1610 | 0.6035 | 0.3060 | 0.8790 |
| Tnfrsf12a | 23812414 | 23814400 | 0.0135 | 0.0889 | 0.1475 | 0.7239 | 0.3512 | 0.7057 | 0.0121 | 0.8252 |
| Cldn6 | 23816332 | 23819413 | 0.1071 | 0.3419 | 0.1598 | 0.7314 | 0.4476 | 0.7538 | 0.3441 | 0.8951 |
| Cldn9 | 23819551 | 23820985 | 0.7897 | 0.7198 | 0.1999 | 0.7468 | 0.9408 | 0.8520 | 0.7099 | 0.9430 |
| 1520401A03Rik | 23841455 | 23859750 | 0.3710 | 0.5869 | 0.5292 | 0.8652 | 0.4587 | 0.7596 | 0.9264 | 0.9544 |
| Pkmyt1 | 23869441 | 23873694 | 0.7211 | 0.7009 | 0.6801 | 0.8890 | 0.5026 | 0.7745 | 0.9301 | 0.9544 |
| Kremen2 | 23878166 | 23882796 | 0.1805 | 0.4414 | 0.8034 | 0.9032 | 0.4557 | 0.7587 | 0.2721 | 0.8733 |
| E030034P13Rik | 23886187 | 23888634 | 0.4021 | 0.5956 | 0.4501 | 0.8426 | 0.4692 | 0.7606 | 0.2726 | 0.8733 |
| Flywch2 | 23913883 | 23923048 | 0.0000 | **0.0000** | 0.1576 | 0.7313 | 0.0940 | 0.4980 | 0.6871 | 0.9430 |
| Srrm2 | 23940154 | 23961706 | 0.6659 | 0.6881 | 0.0282 | 0.5912 | 0.0205 | 0.3177 | 0.4272 | 0.9106 |
| Srrm2 | 23940154 | 23961706 | 0.0787 | 0.2894 | 0.2634 | 0.7770 | 0.1011 | 0.5035 | 0.2070 | 0.8710 |
| Tceb2 | 23961707 | 23966065 | 0.0118 | 0.0816 | 0.6909 | 0.8899 | 0.2199 | 0.6517 | 0.5226 | 0.9248 |
| Dcpp2 | 24035689 | 24037754 | 0.5433 | 0.6476 | 0.1969 | 0.7409 | 0.1433 | 0.5692 | 0.1434 | 0.8648 |
| Tmprss8 | 24109093 | 24112197 | 0.6697 | 0.6882 | 0.9251 | 0.9124 | 0.9270 | 0.8520 | 0.9683 | 0.9592 |
| Prss27 | 24175111 | 24182921 | 0.2730 | 0.5259 | 0.8076 | 0.9032 | 0.8218 | 0.8348 | 0.6920 | 0.9430 |
| Kctd5 | 24184701 | 24210452 | 0.2041 | 0.4619 | 0.1482 | 0.7239 | 0.3147 | 0.6932 | 0.3903 | 0.9024 |
| Pdpk1 | 24210647 | 24287891 | 0.0001 | **0.0009** | 0.3724 | 0.8268 | 0.0388 | 0.3937 | 0.7455 | 0.9430 |
| Amdhd2 | 24292800 | 24300733 | 0.0573 | 0.2391 | 0.1410 | 0.7239 | 0.0122 | 0.2921 | 0.0652 | 0.8387 |
| Tbc1d24 | 24315731 | 24342460 | 0.1692 | 0.4311 | 0.1059 | 0.7008 | 0.0702 | 0.4815 | 0.7619 | 0.9476 |
| Ntn2l | 24340793 | 24346332 | 0.5765 | 0.6563 | 0.6486 | 0.8808 | 0.0076 | 0.2487 | 0.9052 | 0.9544 |
| Ccnf | 24360177 | 24388270 | 0.7639 | 0.7119 | 0.4650 | 0.8426 | 0.8028 | 0.8348 | 0.7046 | 0.9430 |
| Abca17 | 24401204 | 24487974 | 0.9149 | 0.7344 | 0.2185 | 0.7525 | 0.0819 | 0.4889 | 0.2199 | 0.8710 |
| Abca3 | 24488895 | 24547146 | 0.0708 | 0.2780 | 0.9403 | 0.9139 | 0.0621 | 0.4581 | 0.4751 | 0.9106 |
| Rnps1 | 24551620 | 24562840 | 0.0000 | **0.0000** | 0.9163 | 0.9124 | 0.5637 | 0.7990 | 0.2208 | 0.8710 |
| Dci | 24563639 | 24576261 | 0.0001 | **0.0008** | 0.0881 | 0.6688 | 0.7842 | 0.8321 | 0.3042 | 0.8790 |
| Dnase1l2 | 24577026 | 24580050 | 0.5229 | 0.6445 | 0.8793 | 0.9095 | 0.4852 | 0.7719 | 0.3066 | 0.8790 |
| E4f1 | 24580737 | 24592256 | 0.3156 | 0.5560 | 0.6491 | 0.8808 | 0.0211 | 0.3177 | 0.0741 | 0.8387 |
| Pgp | 24607418 | 24608536 | 0.0900 | 0.3061 | 0.9303 | 0.9124 | 0.4868 | 0.7719 | 0.2836 | 0.8778 |
| Gbl | 24610497 | 24615996 | 0.0773 | 0.2888 | 0.6857 | 0.8899 | 0.8450 | 0.8348 | 0.1099 | 0.8558 |
| 3300002N10Rik | 24625728 | 24645850 | 0.7233 | 0.7018 | 0.0991 | 0.7008 | 0.3567 | 0.7076 | 0.4804 | 0.9106 |
| Traf7 | 24645795 | 24664775 | 0.2476 | 0.5069 | 0.9691 | 0.9153 | 0.6929 | 0.8083 | 0.8285 | 0.9531 |
| Rab26 | 24665999 | 24670833 | 0.3352 | 0.5643 | 0.3697 | 0.8268 | 0.3466 | 0.7004 | 0.1800 | 0.8710 |
| Tsc2 | 24732882 | 24769574 | 0.0153 | 0.0973 | 0.0139 | 0.4767 | 0.0409 | 0.4037 | 0.9228 | 0.9544 |
| Nthl1 | 24769655 | 24775782 | 0.0243 | 0.1346 | 0.0060 | 0.3723 | 0.1740 | 0.6191 | 0.2901 | 0.8778 |
| Slc9a3r2 | 24776233 | 24787223 | 0.0823 | 0.2948 | 0.8200 | 0.9032 | 0.1228 | 0.5459 | 0.2593 | 0.8710 |
| Npw | 24794275 | 24795151 | 0.3740 | 0.5869 | 0.8354 | 0.9051 | 0.6234 | 0.8083 | 0.9108 | 0.9544 |
| Zfp598 | 24806697 | 24818960 | 0.7970 | 0.7207 | 0.4819 | 0.8446 | 0.0091 | 0.2620 | 0.8357 | 0.9531 |
| Gfer | 24830136 | 24833018 | 0.7185 | 0.7009 | 0.1233 | 0.7237 | 0.4092 | 0.7390 | 0.2583 | 0.8710 |
| Noxo1 | 24833179 | 24837474 | 0.0060 | **0.0484** | 0.1683 | 0.7377 | 0.5342 | 0.7926 | 0.4858 | 0.9106 |
| Tbl3 | 24834894 | 24844605 | 0.2145 | 0.4705 | 0.1442 | 0.7239 | 0.2590 | 0.6743 | 0.2155 | 0.8710 |
| Rps2 | 24855061 | 24858874 | 0.0000 | **0.0000** | 0.7038 | 0.8905 | 0.0384 | 0.3937 | 0.0870 | 0.8474 |
| Ndufb10 | 24859005 | 24861423 | 0.6562 | 0.6871 | 0.7146 | 0.8905 | 0.2986 | 0.6875 | 0.0553 | 0.8252 |
| Rpl3l | 24869630 | 24873090 | 0.3448 | 0.5686 | 0.5810 | 0.8716 | 0.3348 | 0.6972 | 0.6276 | 0.9430 |
| Sepx1 | 24873587 | 24879723 | 0.1476 | 0.3997 | 0.0390 | 0.5912 | 0.4102 | 0.7390 | 0.6975 | 0.9430 |
| Hs3st6 | 24889948 | 24895628 | 0.3140 | 0.5560 | 0.3039 | 0.7852 | 0.0875 | 0.4959 | 0.4741 | 0.9106 |
| 4930528F23Rik | 24966014 | 24976644 | 0.0000 | **0.0000** | 0.6356 | 0.8758 | 0.3484 | 0.7024 | 0.0973 | 0.8511 |
| Fahd1 | 24986363 | 24987046 | 0.1014 | 0.3324 | 0.2502 | 0.7770 | 0.2732 | 0.6777 | 0.0667 | 0.8387 |
| Hagh | 24987435 | 25001395 | 0.0002 | **0.0030** | 0.8509 | 0.9070 | 0.0176 | 0.3177 | 0.2803 | 0.8753 |
| Igfals | 25015715 | 25018953 | 0.0034 | **0.0305** | 0.3264 | 0.7917 | 0.3603 | 0.7093 | 0.6722 | 0.9430 |
| Nubp2 | 25019564 | 25023273 | 0.0000 | **0.0000** | 0.7649 | 0.8977 | 0.7248 | 0.8208 | 0.3540 | 0.8971 |
| Spsb3 | 25023588 | 25029097 | 0.0000 | **0.0003** | 0.3993 | 0.8352 | 0.6507 | 0.8083 | 0.3858 | 0.9007 |
| Eme2 | 25025431 | 25032032 | 0.0000 | **0.0000** | 0.8028 | 0.9032 | 0.4550 | 0.7583 | 0.7816 | 0.9476 |
| Mapk8ip3 | 25029098 | 25073913 | 0.0000 | **0.0006** | 0.9231 | 0.9124 | 0.6876 | 0.8083 | 0.7531 | 0.9430 |
| Mrps34 | 25032061 | 25034447 | 0.3030 | 0.5476 | 0.0494 | 0.6276 | 0.7069 | 0.8138 | 0.6280 | 0.9430 |
| Nme3 | 25033445 | 25034467 | 0.0030 | **0.0274** | 0.1327 | 0.7239 | 0.6431 | 0.8083 | 0.9074 | 0.9544 |
| Hn1l | 25079415 | 25097568 | 0.0000 | **0.0000** | 0.0799 | 0.6456 | 0.7388 | 0.8240 | 0.0218 | 0.8252 |
| Cramp1l | 25098248 | 25152171 | 0.1271 | 0.3721 | 0.7520 | 0.8937 | 0.3424 | 0.6990 | 0.6846 | 0.9430 |
| Ift140 | 25153036 | 25236440 | 0.0283 | 0.1502 | 0.5293 | 0.8652 | 0.4346 | 0.7531 | 0.7579 | 0.9444 |
| Tmem204 | 25194647 | 25218126 | 0.1412 | 0.3951 | 0.6374 | 0.8768 | 0.3511 | 0.7057 | 0.0864 | 0.8474 |
| Telo2 | 25236515 | 25252912 | 0.0485 | 0.2134 | 0.2776 | 0.7770 | 0.8754 | 0.8394 | 0.8721 | 0.9544 |
| Clcn7 | 25270336 | 25299049 | 0.7619 | 0.7119 | 0.8802 | 0.9095 | 0.4249 | 0.7480 | 0.6003 | 0.9408 |
| Gm317 | 25299787 | 25308796 | 0.8121 | 0.7207 | 0.3170 | 0.7877 | 0.6397 | 0.8083 | 0.3078 | 0.8790 |
| BC003965 | 25321520 | 25322450 | 0.9610 | 0.7434 | 0.1155 | 0.7079 | 0.6325 | 0.8083 | 0.9251 | 0.9544 |
| Unkl | 25325342 | 25371388 | 0.6430 | 0.6823 | 0.4455 | 0.8426 | 0.3148 | 0.6932 | 0.5867 | 0.9366 |
| Gnptg | 25371262 | 25377061 | 0.0001 | **0.0018** | 0.1123 | 0.7061 | 0.1852 | 0.6352 | 0.0032 | 0.5944 |
| 0610007P22Rik | 25377149 | 25379742 | 0.0057 | **0.0461** | 0.0542 | 0.6276 | 0.4939 | 0.7719 | 0.1918 | 0.8710 |
| Baiap3 | 25379605 | 25389080 | 0.8552 | 0.7207 | 0.3986 | 0.8352 | 0.2938 | 0.6846 | 0.4411 | 0.9106 |
| Ube2i | 25397456 | 25410336 | 0.0000 | **0.0008** | 0.0296 | 0.5912 | 0.4572 | 0.7595 | 0.7801 | 0.9476 |
| Prss34 | 25435339 | 25437106 | 0.8500 | 0.7207 | 0.2875 | 0.7801 | 0.2054 | 0.6474 | 0.3569 | 0.8971 |
| Prss29 | 25457228 | 25459625 | 0.0000 | **0.0000** | 0.0617 | 0.6276 | 0.0058 | 0.2126 | 0.0002 | 0.1568 |
| Tpsab1 | 25480190 | 25482507 | 0.0549 | 0.2338 | 0.3193 | 0.7877 | 0.7665 | 0.8321 | 0.0609 | 0.8252 |
| Mcpt6 | 25503278 | 25505037 | 0.8548 | 0.7207 | 0.4737 | 0.8426 | 0.5415 | 0.7947 | 0.3371 | 0.8930 |
| Cacna1h | 25511230 | 25570728 | 0.2749 | 0.5288 | 0.1893 | 0.7387 | 0.6521 | 0.8083 | 0.4188 | 0.9106 |
| Sox8 | 25702838 | 25707631 | 0.4018 | 0.5956 | 0.5986 | 0.8716 | 0.2143 | 0.6508 | 0.5587 | 0.9281 |
| Lmf1 | 25716122 | 25799771 | 0.3713 | 0.5869 | 0.3981 | 0.8352 | 0.8139 | 0.8348 | 0.8835 | 0.9544 |
| Gng13 | 25854473 | 25856029 | 0.5590 | 0.6515 | 0.2313 | 0.7651 | 0.7113 | 0.8139 | 0.2446 | 0.8710 |
| Chtf18 | 25855976 | 25864347 | 0.1578 | 0.4142 | 0.1267 | 0.7239 | 0.2654 | 0.6777 | 0.7193 | 0.9430 |
| Rpusd1 | 25864696 | 25868401 | 0.4650 | 0.6236 | 0.9523 | 0.9148 | 0.0802 | 0.4879 | 0.8056 | 0.9531 |
| Msln | 25885558 | 25891272 | 0.9784 | 0.7474 | 0.5922 | 0.8716 | 0.7564 | 0.8308 | 0.2354 | 0.8710 |
| ***DMetS17b*** | | | | | | | | | | |
| 1300018I05Rik | 29686744 | 29842924 | 0.0004 | **0.0050** | 0.4097 | 0.8380 | 0.7325 | 0.8216 | 0.0520 | 0.8252 |
| Rnf8 | 29751735 | 29840304 | 0.0000 | **0.0007** | 0.1660 | 0.7345 | 0.0168 | 0.3177 | 0.9823 | 0.9621 |
| 1110021J02Rik | 29826323 | 29853957 | 0.6280 | 0.6770 | 0.1987 | 0.7459 | 0.9866 | 0.8578 | 0.7819 | 0.9476 |
| Mdga1 | 29964903 | 30024827 | 0.8150 | 0.7207 | 0.8882 | 0.9095 | 0.2633 | 0.6777 | 0.0177 | 0.8252 |
| Zfand3 | 30142032 | 30346964 | 0.0009 | **0.0098** | 0.7878 | 0.9000 | 0.7884 | 0.8321 | 0.0995 | 0.8511 |
| Btbd9 | 30357046 | 30667310 | 0.0238 | 0.1333 | 0.9864 | 0.9207 | 0.6515 | 0.8083 | 0.7798 | 0.9476 |
| Glo1 | 30729806 | 30749539 | 0.0000 | **0.0000** | 0.0688 | 0.6276 | 0.0178 | 0.3177 | 0.6759 | 0.9430 |
| Dnahc8 | 30763936 | 31012209 | 0.1982 | 0.4567 | 0.6353 | 0.8758 | 0.7031 | 0.8128 | 0.2775 | 0.8733 |
| Glp1r | 31038812 | 31073455 | 0.8265 | 0.7207 | 0.9637 | 0.9153 | 0.1958 | 0.6407 | 0.3340 | 0.8930 |
| Abcg1 | 31194643 | 31252722 | 0.8138 | 0.7207 | 0.4892 | 0.8470 | 0.2187 | 0.6517 | 0.5462 | 0.9281 |
| Tff3 | 31262251 | 31266591 | 0.1712 | 0.4322 | 0.1507 | 0.7256 | 0.2703 | 0.6777 | 0.2381 | 0.8710 |
| Tff2 | 31277994 | 31281227 | 0.3703 | 0.5869 | 0.9454 | 0.9148 | 0.4090 | 0.7390 | 0.0818 | 0.8387 |
| Ubash3a | 31344818 | 31379147 | 0.4139 | 0.5984 | 0.0228 | 0.5888 | 0.4895 | 0.7719 | 0.0742 | 0.8387 |
| Rsph1 | 31391969 | 31414252 | 0.2602 | 0.5173 | 0.8652 | 0.9084 | 0.1317 | 0.5552 | 0.2626 | 0.8716 |
| Slc37a1 | 31433702 | 31487569 | 0.3335 | 0.5632 | 0.4159 | 0.8380 | 0.2568 | 0.6728 | 0.6048 | 0.9408 |
| Wdr4 | 31632569 | 31649202 | 0.0376 | 0.1813 | 0.1157 | 0.7079 | 0.1831 | 0.6336 | 0.1600 | 0.8710 |
| 1500032D16Rik | 31657120 | 31668268 | 0.3652 | 0.5837 | 0.2171 | 0.7525 | 0.5509 | 0.7990 | 0.6572 | 0.9430 |
| 4833413E03Rik | 31694071 | 31696058 | 0.3895 | 0.5896 | 0.5478 | 0.8653 | 0.7579 | 0.8319 | 0.7795 | 0.9476 |
| Pknox1 | 31720641 | 31744629 | 0.5119 | 0.6432 | 0.9133 | 0.9124 | 0.5919 | 0.8022 | 0.6961 | 0.9430 |
| Cbs | 31749568 | 31774144 | 0.0812 | 0.2915 | 0.2722 | 0.7770 | 0.0129 | 0.2983 | 0.6633 | 0.9430 |
| U2af1 | 31784028 | 31795660 | 0.0001 | **0.0016** | 0.2759 | 0.7770 | 0.5933 | 0.8022 | 0.1812 | 0.8710 |
| Snf1lk | 31981193 | 31992737 | 0.0000 | **0.0000** | 0.0765 | 0.6367 | 0.8878 | 0.8442 | 0.0251 | 0.8252 |
| Rrp1b | 32173045 | 32199810 | 0.0037 | **0.0325** | 0.1210 | 0.7221 | 0.2526 | 0.6728 | 0.8931 | 0.9544 |
| Notch3 | 32257765 | 32303825 | 0.8405 | 0.7207 | 0.5769 | 0.8716 | 0.8485 | 0.8355 | 0.0212 | 0.8252 |
| Abhd9 | 32320715 | 32326494 | 0.7295 | 0.7047 | 0.0922 | 0.6845 | 0.8599 | 0.8369 | 0.0994 | 0.8511 |
| Brd4 | 32333219 | 32421667 | 0.0002 | **0.0029** | 0.6392 | 0.8784 | 0.0578 | 0.4581 | 0.4594 | 0.9106 |
| Akap8 | 32440621 | 32458098 | 0.2260 | 0.4845 | 0.3504 | 0.8085 | 0.5465 | 0.7984 | 0.9680 | 0.9592 |
| Akap8l | 32458370 | 32483746 | 0.9882 | 0.7506 | 0.7443 | 0.8937 | 0.2947 | 0.6850 | 0.6204 | 0.9430 |
| Wiz | 32491011 | 32526361 | 0.6426 | 0.6823 | 0.7874 | 0.9000 | 0.0232 | 0.3298 | 0.7420 | 0.9430 |
| A430107D22Rik | 32527604 | 32540528 | 0.5067 | 0.6401 | 0.2250 | 0.7580 | 0.0946 | 0.4980 | 0.7426 | 0.9430 |
| Cyp4f39 | 32589668 | 32630265 | 0.5437 | 0.6476 | 0.1699 | 0.7387 | 0.5147 | 0.7798 | 0.3972 | 0.9104 |
| Cyp4f16 | 32673574 | 32688742 | 0.0741 | 0.2858 | 0.3466 | 0.8033 | 0.0009 | 0.1077 | 0.3619 | 0.8971 |
| EG631304 | 32796431 | 32813425 | 0.3827 | 0.5896 | 0.1289 | 0.7239 | 0.5064 | 0.7775 | 0.4088 | 0.9106 |
| Cyp4f15 | 32822624 | 32840296 | 0.0002 | **0.0031** | 0.5115 | 0.8636 | 0.8067 | 0.8348 | 0.2108 | 0.8710 |
| 9030612M13Rik | 32910210 | 32924492 | 0.0000 | **0.0000** | 0.2466 | 0.7770 | 0.0000 | **0.0399** | 0.0538 | 0.8252 |
| Zfp811 | 32933958 | 32937883 | 0.4545 | 0.6188 | 0.8658 | 0.9084 | 0.2868 | 0.6777 | 0.1083 | 0.8558 |
| BC066107 | 33016173 | 33023028 | 0.0000 | **0.0000** | 0.1432 | 0.7239 | 0.5091 | 0.7775 | 0.3964 | 0.9104 |
| Cyp4f14 | 33042016 | 33054023 | 0.2831 | 0.5332 | 0.9365 | 0.9139 | 0.0054 | 0.2097 | 0.7681 | 0.9476 |
| Cyp4f13 | 33061633 | 33084347 | 0.0026 | **0.0246** | 0.0053 | 0.3723 | 0.0413 | 0.4037 | 0.5960 | 0.9406 |
| Zfp472 | 33102759 | 33116178 | 0.2386 | 0.5019 | 0.5572 | 0.8666 | 0.4342 | 0.7531 | 0.6865 | 0.9430 |
| 1700065O13Rik | 33153808 | 33170347 | 0.6519 | 0.6838 | 0.8213 | 0.9032 | 0.9468 | 0.8520 | 0.7116 | 0.9430 |
| ***DMetS18a*** | | | | | | | | | | |
| Dcc | 71418392 | 72510723 | 0.2155 | 0.4720 | 0.3934 | 0.8352 | 0.8789 | 0.8407 | 0.4477 | 0.9106 |
| ***DMetS19a*** | | | | | | | | | | |
| Stx3 | 11849609 | 11893893 | 0.0794 | 0.2905 | 0.4399 | 0.8426 | 0.0151 | 0.3177 | 0.0089 | 0.8252 |
| Patl1 | 11986948 | 12019456 | 0.1118 | 0.3509 | 0.8597 | 0.9082 | 0.3791 | 0.7230 | 0.3664 | 0.8971 |
| Osbp | 12040431 | 12066533 | 0.8385 | 0.7207 | 0.1862 | 0.7387 | 0.1557 | 0.5899 | 0.5656 | 0.9298 |
| Olfr1424 | 12133299 | 12134240 | 0.0738 | 0.2856 | 0.8893 | 0.9095 | 0.2015 | 0.6407 | 0.5273 | 0.9248 |
| Olfr76 | 12194211 | 12195164 | 0.9692 | 0.7437 | 0.9920 | 0.9208 | 0.1246 | 0.5490 | 0.0706 | 0.8387 |
| Olfr262 | 12315211 | 12316149 | 0.5029 | 0.6396 | 0.3857 | 0.8321 | 0.8587 | 0.8369 | 0.3361 | 0.8930 |
| Olfr232 | 12342722 | 12343660 | 0.7177 | 0.7009 | 0.7050 | 0.8905 | 0.0745 | 0.4823 | 0.2455 | 0.8710 |
| Olfr1436 | 12372673 | 12373620 | 0.4926 | 0.6357 | 0.4751 | 0.8426 | 0.5820 | 0.8019 | 0.6900 | 0.9430 |
| Olfr1437 | 12396377 | 12397315 | 0.4976 | 0.6369 | 0.9221 | 0.9124 | 0.9862 | 0.8578 | 0.0780 | 0.8387 |
| Mpeg1 | 12535546 | 12538291 | 0.0543 | 0.2329 | 0.6098 | 0.8716 | 0.0122 | 0.2921 | 0.2320 | 0.8710 |
| Dtx4 | 12540831 | 12575944 | 0.0121 | 0.0820 | 0.6737 | 0.8868 | 0.1122 | 0.5231 | 0.9880 | 0.9621 |
| 4632417K18Rik | 12620230 | 12664258 | 0.4596 | 0.6196 | 0.3884 | 0.8345 | 0.1403 | 0.5630 | 0.9336 | 0.9544 |
| EG240549 | 12674578 | 12702103 | 0.1963 | 0.4567 | 0.5699 | 0.8705 | 0.1192 | 0.5367 | 0.3584 | 0.8971 |
| Glyat | 12707798 | 12728401 | 0.4785 | 0.6298 | 0.0880 | 0.6688 | 0.7844 | 0.8321 | 0.7152 | 0.9430 |
| Olfr1443 | 12752656 | 12758333 | 0.0242 | 0.1346 | 0.4606 | 0.8426 | 0.6070 | 0.8022 | 0.4669 | 0.9106 |
| Keg1 | 12770276 | 12794392 | 0.0052 | **0.0431** | 0.6958 | 0.8899 | 0.3342 | 0.6972 | 0.4123 | 0.9106 |
| Zfp91 | 12838150 | 12870616 | 0.9126 | 0.7340 | 0.1181 | 0.7079 | 0.0699 | 0.4815 | 0.6925 | 0.9430 |
| Lpxn | 12873133 | 12908301 | 0.5407 | 0.6476 | 0.2725 | 0.7770 | 0.0201 | 0.3177 | 0.9785 | 0.9614 |
| EG433224 | 12980338 | 12980970 | 0.5243 | 0.6445 | 0.4384 | 0.8426 | 0.0333 | 0.3783 | 0.5002 | 0.9106 |
| Olfr1449 | 13009230 | 13010174 | 0.8500 | 0.7207 | 0.4478 | 0.8426 | 0.2753 | 0.6777 | 0.2447 | 0.8710 |
| Olfr1459 | 13220224 | 13221147 | 0.3367 | 0.5649 | 0.6052 | 0.8716 | 0.6835 | 0.8083 | 0.8277 | 0.9531 |
| Olfr1469 | 13485061 | 13485990 | 0.7708 | 0.7144 | 0.7865 | 0.9000 | 0.2412 | 0.6664 | 0.0686 | 0.8387 |
| Olfr1471 | 13519504 | 13520448 | 0.5487 | 0.6476 | 0.6037 | 0.8716 | 0.2373 | 0.6623 | 0.7845 | 0.9482 |
| Olfr1490 | 13728936 | 13729886 | 0.3078 | 0.5511 | 0.8069 | 0.9032 | 0.6500 | 0.8083 | 0.2362 | 0.8710 |
| Olfr1496 | 13855104 | 13856057 | 0.0545 | 0.2331 | 0.9441 | 0.9148 | 0.9169 | 0.8510 | 0.1075 | 0.8558 |
| Olfr1500 | 13901949 | 13902884 | 0.9760 | 0.7472 | 0.2883 | 0.7801 | 0.0584 | 0.4581 | 0.9750 | 0.9614 |
| Olfr1502 | 13936285 | 13937235 | 0.4055 | 0.5970 | 0.0851 | 0.6584 | 0.0172 | 0.3177 | 0.9142 | 0.9544 |
| Tle4 | 14522562 | 14672473 | 0.0140 | 0.0904 | 0.1849 | 0.7387 | 0.7844 | 0.8321 | 0.1174 | 0.8627 |
| Psat1 | 15979168 | 16021827 | 0.8223 | 0.7207 | 0.4444 | 0.8426 | 0.7306 | 0.8216 | 0.3079 | 0.8790 |
| Cep78 | 16030264 | 16059479 | 0.2976 | 0.5437 | 0.9423 | 0.9145 | 0.2275 | 0.6584 | 0.9941 | 0.9625 |
