# Supplementary material for: Genetic Effects at Pleiotropic Loci Are Context-Dependent with Consequences for the Maintenance of Genetic Variation in Populations
Source: PLoS Genet. 2011 Sep 8;7(9):e1002256. doi: 10.1371/journal.pgen.1002256 (PMC3169520; doi:10.1371/journal.pgen.1002256)
Supplement: Table S4 — Number of SNPs between LG/J and SM/J in both coding and non-coding sequence in MetS QTL. (DOC) [file pgen.1002256.s006.doc]

| **QTL** | **Start** | **Stop** | **Synonymous** | **nonSynonymous** | **Intronic** | **10kb-upstream** | **10kb-downstream** | **5'_UTR** | **3'_UTR** | **Intergenic** |
| --- | --- | --- | --- | --- | --- | --- | --- | --- | --- | --- |
| *DMetS1a* | 76371591 | 78164165 | 5 | 0 | 632 | 20 | 53 | 0 | 12 | 4239 |
| *DMetS1b* | 171317995 | 173523075 | 74 | 24 | 2974 | 1724 | 1346 | 22 | 80 | 854 |
| *DMetS2a* | 68441214 | 71852107 | 51 | 26 | 3023 | 761 | 545 | 17 | 89 | 940 |
| *DMetS2b* | 82390259 | 88186456 | 144 | 96 | 2160 | 3387 | 3555 | 21 | 60 | 3759 |
| *DMetS2c* | 102736588 | 106028947 | 48 | 20 | 3791 | 983 | 1242 | 13 | 96 | 1954 |
| *DMetS4a* | 146617280 | 152935160 | 115 | 26 | 6548 | 2214 | 2322 | 60 | 278 | 2757 |
| *DMetS6a* | 47108519 | 49866636 | 65 | 30 | 812 | 807 | 1068 | 32 | 85 | 446 |
| *DMetS6b* | 88011079 | 91810044 | 26 | 6 | 1203 | 451 | 415 | 4 | 22 | 1415 |
| *DMetS6c* | 105083653 | 111374046 | 13 | 9 | 11209 | 285 | 260 | 8 | 23 | 11041 |
| *DMetS7a* | 65961266 | 72799172 | 20 | 13 | 5716 | 736 | 497 | 12 | 38 | 7531 |
| *DMetS7b* | 78237095 | 80754000 | 2 | 0 | 403 | 50 | 99 | 0 | 27 | 4594 |
| *DMetS7c* | 87443176 | 93275341 | 113 | 69 | 6932 | 1566 | 1755 | 23 | 174 | 7075 |
| *DMetS8a* | 21545093 | 31866304 | 29 | 28 | 993 | 756 | 684 | 6 | 106 | 3556 |
| *DMetS8b* | 85363247 | 87124162 | 145 | 52 | 8422 | 3046 | 2923 | 17 | 227 | 3014 |
| *DMetS10a* | 95364897 | 100409761 | 47 | 20 | 2478 | 759 | 551 | 17 | 50 | 9954 |
| *DMetS10b* | 114179164 | 116830926 | 135 | 31 | 11312 | 2319 | 2584 | 56 | 220 | 5160 |
| *DMetS14a* | 23375824 | 28976151 | 3 | 1 | 296 | 40 | 15 | 3 | 1 | 66 |
| *DMetS15a* | 66420641 | 68421408 | 34 | 23 | 1662 | 365 | 261 | 3 | 40 | 4683 |
| *DMetS16a* | 53030825 | 59276040 | 246 | 141 | 23307 | 4146 | 4296 | 59 | 292 | 16537 |
| *DMetS17a* | 23495906 | 25901580 | 239 | 106 | 6118 | 4441 | 4300 | 90 | 202 | 1095 |
| *DMetS17b* | 29824483 | 33155342 | 95 | 42 | 4821 | 1107 | 1748 | 6 | 175 | 1881 |
| *DMetS18a* | 71804268 | 73508523 | 0 | 0 | 833 | 5 | 0 | 0 | 0 | 3667 |
| *DMetS19a* | 11893625 | 16606210 | 10 | 1 | 653 | 148 | 189 | 1 | 5 | 2420 |
