# Supplementary material for: Genetic Effects at Pleiotropic Loci Are Context-Dependent with Consequences for the Maintenance of Genetic Variation in Populations
Source: PLoS Genet. 2011 Sep 8;7(9):e1002256. doi: 10.1371/journal.pgen.1002256 (PMC3169520; doi:10.1371/journal.pgen.1002256)
Supplement: Table S5 — Markers used for QTL mapping in the F16 LG/J x SM/J Advanced Intercross. (DOC) [file pgen.1002256.s007.doc]

| **Chromosome** | **SNP ID** | **Map Position (cM)** | **Genome Coordinate (mm9; build 37)** |
| --- | --- | --- | --- |
| 1 | rs3683945 | 0.00 | 3187481 |
| 1 | rs3658242 | 0.00 | 4133259 |
| 1 | rs3677817 | 1.94 | 5176059 |
| 1 | rs13475709 | 1.94 | 6646044 |
| 1 | rs6321307 | 4.31 | 7664402 |
| 1 | rs13475717 | 5.22 | 8812597 |
| 1 | rs3698285 | 5.97 | 10019112 |
| 1 | rs3697012 | 13.10 | 10891409 |
| 1 | rs3681537 | 13.49 | 11924238 |
| 1 | rs13475748 | 43.96 | 18911762 |
| 1 | rs6334092 | 45.43 | 20153983 |
| 1 | rs13475757 | 54.14 | 21277037 |
| 1 | rs6361963 | 67.32 | 22671898 |
| 1 | rs13475765 | 74.30 | 23822074 |
| 1 | rs13475769 | 74.55 | 24958695 |
| 1 | rs13475774 | 82.91 | 26101762 |
| 1 | mCV25266528 | 82.91 | 27850773 |
| 1 | rs3713281 | 82.91 | 29009416 |
| 1 | rs13475788 | 82.91 | 29807982 |
| 1 | rs4137502 | 82.91 | 30887618 |
| 1 | rs3707642 | 83.66 | 32568345 |
| 1 | rs13475816 | 132.94 | 37614783 |
| 1 | rs3685569 | 138.64 | 38607247 |
| 1 | rs3680400 | 153.40 | 39913715 |
| 1 | gnf01.037.906 | 164.76 | 41151472 |
| 1 | rs3663706 | 170.98 | 42070333 |
| 1 | rs13475837 | 187.50 | 43168109 |
| 1 | rs13475863 | 192.92 | 50773412 |
| 1 | rs6217547 | 197.42 | 51990511 |
| 1 | rs13475874 | 204.48 | 54502962 |
| 1 | rs3653534 | 216.67 | 56866296 |
| 1 | rs8238898 | 221.62 | 58111643 |
| 1 | petAF067836-350A-1 | 231.46 | 58883769 |
| 1 | rs6353774 | 240.52 | 59780022 |
| 1 | rs13475894 | 243.10 | 63625005 |
| 1 | rs3163007 | 248.81 | 64087683 |
| 1 | rs6293581 | 251.60 | 64789338 |
| 1 | rs13475896 | 251.60 | 65111596 |
| 1 | rs13475902 | 259.26 | 66461486 |
| 1 | rs13475903 | 259.31 | 66782743 |
| 1 | rs13475906 | 259.31 | 67618356 |
| 1 | rs6288543 | 268.71 | 68556956 |
| 1 | rs6323094 | 272.21 | 70774788 |
| 1 | rs6191076 | 280.53 | 72000786 |
| 1 | rs3716722 | 285.68 | 72361385 |
| 1 | rs4222426 | 285.68 | 72440964 |
| 1 | rs13475919 | 288.86 | 73020555 |
| 1 | mCV23057534 | 291.12 | 73645269 |
| 1 | rs13475922 | 291.17 | 73785952 |
| 1 | rs13475927 | 297.40 | 74948766 |
| 1 | rs6405821 | 297.40 | 75283446 |
| 1 | rs13475931 | 305.11 | 76371591 |
| 1 | rs13475932 | 305.26 | 76557546 |
| 1 | rs3715800 | 305.26 | 76887644 |
| 1 | rs3703285 | 306.52 | 77565875 |
| 1 | gnf01.075.385 | 309.86 | 77901204 |
| 1 | rs3667200 | 309.96 | 78164165 |
| 1 | rs6182343 | 312.92 | 78312525 |
| 1 | rs13475939 | 313.22 | 78684289 |
| 1 | rs3692514 | 313.22 | 78729204 |
| 1 | rs8253473 | 313.22 | 79431922 |
| 1 | rs3692549 | 313.60 | 79761258 |
| 1 | mCV23431007 | 316.92 | 80322428 |
| 1 | rs13475945 | 316.92 | 80586665 |
| 1 | mCV23433457 | 328.60 | 80976565 |
| 1 | rs6290463 | 328.65 | 81372526 |
| 1 | rs13475950 | 328.75 | 81806553 |
| 1 | rs13475951 | 328.92 | 82023244 |
| 1 | rs6288514 | 337.13 | 83525051 |
| 1 | rs4222486 | 338.50 | 84300368 |
| 1 | rs3694406 | 340.60 | 88316693 |
| 1 | rs13475970 | 342.48 | 90246345 |
| 1 | rs13475973 | 347.80 | 91391585 |
| 1 | UT-1-92.862916 | 350.76 | 92847128 |
| 1 | rs3664800 | 352.70 | 93724097 |
| 1 | rs6268443 | 358.07 | 95279164 |
| 1 | rs3675505 | 360.54 | 97035544 |
| 1 | rs13475997 | 360.54 | 97723776 |
| 1 | rs13475999 | 360.54 | 98052037 |
| 1 | rs6342650 | 360.59 | 98418580 |
| 1 | rs3695980 | 360.59 | 101469745 |
| 1 | rs3692731 | 368.64 | 107878372 |
| 1 | rs13476036 | 370.16 | 108755358 |
| 1 | rs3664301 | 370.16 | 108980402 |
| 1 | rs13476044 | 370.16 | 110578148 |
| 1 | rs13476045 | 370.21 | 110960852 |
| 1 | rs3685919 | 370.56 | 111528321 |
| 1 | rs6382744 | 370.56 | 111758070 |
| 1 | rs6319405 | 370.86 | 112363108 |
| 1 | CEL-1-111503693 | 371.01 | 113377972 |
| 1 | rs6168275 | 371.01 | 113643051 |
| 1 | mCV22824651 | 371.06 | 114217862 |
| 1 | rs13476060 | 371.11 | 115486194 |
| 1 | rs3725808 | 373.11 | 116045359 |
| 1 | rs13476064 | 373.11 | 116488503 |
| 1 | rs13476067 | 373.21 | 117157941 |
| 1 | mCV24201027 | 373.21 | 117626780 |
| 1 | rs13476069 | 373.31 | 117932805 |
| 1 | rs3694226 | 379.09 | 119019101 |
| 1 | CEL-1-117526378 | 379.09 | 119395321 |
| 1 | gnf01.117.970 | 379.09 | 120206187 |
| 1 | rs3695581 | 379.09 | 120637062 |
| 1 | rs3719973 | 379.69 | 120970185 |
| 1 | rs13476079 | 380.40 | 121277333 |
| 1 | rs13476081 | 380.40 | 121756703 |
| 1 | rs3686671 | 387.45 | 123825217 |
| 1 | rs13476086 | 387.45 | 124120690 |
| 1 | rs13476089 | 388.87 | 124563739 |
| 1 | rs3717360 | 388.87 | 124897243 |
| 1 | rs13476090 | 388.87 | 125220908 |
| 1 | rs3694822 | 388.87 | 125741682 |
| 1 | rs6189020 | 390.54 | 126516498 |
| 1 | rs3687720 | 391.75 | 126908318 |
| 1 | rs13476094 | 391.75 | 127192398 |
| 1 | rs13476095 | 391.75 | 127481769 |
| 1 | rs3716165 | 391.75 | 127835242 |
| 1 | rs6228473 | 391.94 | 128523838 |
| 1 | rs3691374 | 406.33 | 128830105 |
| 1 | rs13476100 | 406.33 | 128842817 |
| 1 | rs6308228 | 414.55 | 133189179 |
| 1 | rs3678662 | 420.08 | 134378492 |
| 1 | gnf01.132.831 | 426.47 | 135115364 |
| 1 | rs13476122 | 429.11 | 136766730 |
| 1 | rs13476125 | 435.68 | 138014196 |
| 1 | rs6250257 | 437.83 | 139536983 |
| 1 | rs13476136 | 439.72 | 140723754 |
| 1 | rs6382880 | 443.12 | 144418077 |
| 1 | rs6186115 | 448.67 | 146560237 |
| 1 | rs13476158 | 451.68 | 147636179 |
| 1 | rs3711440 | 451.78 | 149096504 |
| 1 | CEL-1-148141836 | 452.23 | 150055073 |
| 1 | rs3023658 | 456.51 | 151663605 |
| 1 | rs6393307 | 456.76 | 152872094 |
| 1 | rs3688042 | 456.76 | 153102943 |
| 1 | rs6267646 | 464.10 | 154395967 |
| 1 | rs3674280 | 464.12 | 155080475 |
| 1 | gnf01.157.188 | 479.97 | 159709681 |
| 1 | rs3709285 | 490.15 | 160965088 |
| 1 | rs13476207 | 499.43 | 163036933 |
| 1 | rs3685643 | 502.08 | 164180191 |
| 1 | rs13476211 | 507.01 | 164990866 |
| 1 | rs8256589 | 515.21 | 165996952 |
| 1 | rs8242852 | 577.01 | 172980821 |
| 1 | rs13476237 | 582.26 | 173631526 |
| 1 | rs13476241 | 588.40 | 174792335 |
| 1 | rs13476248 | 592.85 | 176081804 |
| 1 | rs6213386 | 593.05 | 177262372 |
| 1 | rs3723788 | 608.72 | 178114837 |
| 1 | rs13476258 | 609.73 | 178912738 |
| 1 | mCV23509126 | 626.85 | 182669137 |
| 1 | CEL-1-182091301 | 628.47 | 183936096 |
| 1 | rs6157620 | 633.83 | 185385732 |
| 1 | mCV24555989 | 643.44 | 186627144 |
| 1 | rs13476286 | 650.11 | 187711002 |
| 1 | rs3694793 | 652.56 | 188745178 |
| 1 | rs13476293 | 672.58 | 190087098 |
| 1 | rs3654705 | 680.55 | 191159238 |
| 1 | mCV23990401 | 693.31 | 191982750 |
| 1 | rs13476308 | 719.48 | 193610592 |
| 1 | rs13493060 | 722.37 | 194439430 |
| 1 | rs13476312 | 725.68 | 195793450 |
| 2 | rs13476319 | 0.00 | 3379628 |
| 2 | rs6308296 | 0.70 | 4675003 |
| 2 | rs3699393 | 1.55 | 5890785 |
| 2 | rs6359983 | 3.23 | 6668095 |
| 2 | rs13476339 | 10.90 | 8241374 |
| 2 | rs13476343 | 13.56 | 9067020 |
| 2 | gnf02.007.482 | 25.52 | 10478454 |
| 2 | rs3688854 | 71.18 | 20203333 |
| 2 | mCV23209429 | 150.26 | 31135513 |
| 2 | rs13459063 | 154.24 | 32089220 |
| 2 | rs13476425 | 158.22 | 34478980 |
| 2 | rs6313371 | 176.55 | 39451529 |
| 2 | rs13476457 | 176.75 | 41980190 |
| 2 | rs13476462 | 176.80 | 43123156 |
| 2 | rs3680197 | 180.63 | 44064411 |
| 2 | rs13476472 | 186.30 | 45548746 |
| 2 | rs13476476 | 187.77 | 46848832 |
| 2 | rs3718711 | 233.18 | 56388226 |
| 2 | rs6247544 | 233.23 | 57167606 |
| 2 | rs13476523 | 241.62 | 58348075 |
| 2 | rs3708975 | 252.52 | 59438505 |
| 2 | rs3022886 | 268.71 | 62483390 |
| 2 | rs8263587 | 308.55 | 69085304 |
| 2 | rs13476563 | 315.40 | 69970052 |
| 2 | rs6295014 | 323.12 | 71612396 |
| 2 | rs6345126 | 329.02 | 72451387 |
| 2 | rs3710094 | 337.69 | 74197328 |
| 2 | rs3713848 | 347.53 | 75856730 |
| 2 | rs6360325 | 356.53 | 77317323 |
| 2 | CEL-2-79237503 | 357.77 | 79183600 |
| 2 | rs13476598 | 363.59 | 80863557 |
| 2 | mCV23002990 | 366.71 | 82390259 |
| 2 | gnf02.082.243 | 366.76 | 83443426 |
| 2 | rs8273639 | 366.76 | 84612491 |
| 2 | mCV24333820 | 370.09 | 87450633 |
| 2 | rs6314788 | 370.22 | 88186456 |
| 2 | rs3715323 | 371.97 | 89420674 |
| 2 | rs13476631 | 371.97 | 90319297 |
| 2 | rs13476637 | 374.34 | 91785647 |
| 2 | rs13476640 | 375.60 | 92814298 |
| 2 | rs6252400 | 382.21 | 94090103 |
| 2 | rs13476647 | 382.36 | 95438729 |
| 2 | rs6220079 | 382.51 | 97325901 |
| 2 | rs13476660 | 386.67 | 99057158 |
| 2 | rs6406705 | 388.88 | 100200136 |
| 2 | rs13476667 | 391.72 | 101424104 |
| 2 | rs6275228 | 392.32 | 102182519 |
| 2 | rs13469470 | 401.23 | 103290657 |
| 2 | rs3674721 | 403.43 | 104541486 |
| 2 | rs3715478 | 405.37 | 106028947 |
| 2 | rs6208879 | 410.20 | 109622852 |
| 2 | rs3658023 | 410.25 | 110654338 |
| 2 | rs3681694 | 410.35 | 112125008 |
| 2 | CEL-2-113459249 | 421.59 | 113408431 |
| 2 | rs3701250 | 430.34 | 114706732 |
| 2 | rs3708146 | 432.86 | 115832114 |
| 2 | rs13476723 | 443.13 | 117118532 |
| 2 | rs3725853 | 443.22 | 118084713 |
| 2 | rs3661254 | 443.37 | 119334908 |
| 2 | rs3718386 | 443.52 | 120587779 |
| 2 | rs6317255 | 443.72 | 121731797 |
| 2 | rs13476746 | 443.82 | 122736802 |
| 2 | rs13476750 | 446.29 | 123481211 |
| 2 | rs6401493 | 450.57 | 125123509 |
| 2 | rs13476761 | 453.68 | 127292727 |
| 2 | rs6411422 | 455.47 | 128199226 |
| 2 | rs6318992 | 461.81 | 129089789 |
| 2 | rs3661596 | 462.08 | 129970551 |
| 2 | rs13476774 | 463.91 | 131240778 |
| 2 | rs13476787 | 484.20 | 134965497 |
| 2 | rs13476790 | 486.73 | 135469718 |
| 2 | rs13476794 | 487.22 | 136567700 |
| 2 | rs6249968 | 505.18 | 137618158 |
| 2 | rs6200333 | 506.13 | 138895272 |
| 2 | rs6303304 | 520.23 | 141269051 |
| 2 | rs13476811 | 521.34 | 142540462 |
| 2 | rs6195594 | 521.34 | 143420471 |
| 2 | mCV23169908 | 533.93 | 145712335 |
| 2 | rs3701696 | 536.44 | 147254465 |
| 2 | rs3676033 | 547.92 | 149271015 |
| 2 | rs13476846 | 576.64 | 152857172 |
| 2 | rs6209403 | 600.91 | 153865947 |
| 2 | rs13476860 | 640.02 | 156093212 |
| 2 | UT-2-158.095429 | 641.29 | 157295559 |
| 2 | gnf02.161.674 | 642.06 | 158566515 |
| 2 | rs13476876 | 653.12 | 160184840 |
| 2 | rs6204920 | 660.23 | 161566106 |
| 2 | rs3673613 | 664.00 | 162677402 |
| 2 | rs3697980 | 668.17 | 163838228 |
| 2 | rs6185704 | 675.13 | 164779025 |
| 2 | rs13476894 | 684.58 | 165876926 |
| 2 | rs6160839 | 707.76 | 168792186 |
| 2 | rs3689258 | 713.06 | 169313400 |
| 2 | rs13476925 | 799.06 | 173100192 |
| 2 | rs8238755 | 802.94 | 174244010 |
| 2 | rs13476936 | 813.46 | 178942787 |
| 2 | rs13476944 | 828.51 | 180662143 |
| 2 | rs6402916 | 828.60 | 181542229 |
| 3 | rs13476950 | 0.00 | 3785418 |
| 3 | rs13476954 | 0.00 | 4786147 |
| 3 | rs13476958 | 0.05 | 5904283 |
| 3 | rs6274760 | 0.65 | 7013014 |
| 3 | rs13476964 | 1.50 | 7922127 |
| 3 | rs3668064 | 3.01 | 9269819 |
| 3 | rs13476970 | 3.31 | 10018673 |
| 3 | rs13476974 | 4.06 | 11119313 |
| 3 | rs3694133 | 4.06 | 12501777 |
| 3 | rs6172421 | 4.06 | 13643232 |
| 3 | rs13476985 | 5.12 | 14887895 |
| 3 | rs13476992 | 10.85 | 16819051 |
| 3 | rs13477007 | 17.96 | 20357186 |
| 3 | rs6235984 | 26.40 | 21430378 |
| 3 | rs13477017 | 32.34 | 22507002 |
| 3 | rs3677132 | 52.67 | 27819125 |
| 3 | CEL-3-31146697 | 67.75 | 31267065 |
| 3 | rs6351323 | 69.31 | 32363154 |
| 3 | rs4223864 | 70.47 | 32842750 |
| 3 | rs3720738 | 84.56 | 35886710 |
| 3 | rs13477066 | 111.37 | 38297775 |
| 3 | gnf03.036.164 | 112.62 | 39215759 |
| 3 | rs13477072 | 122.68 | 40358478 |
| 3 | CEL-3-46558020 | 125.09 | 46357974 |
| 3 | rs3671459 | 141.78 | 49927981 |
| 3 | rs3719360 | 147.47 | 51067242 |
| 3 | rs6335414 | 160.88 | 52821781 |
| 3 | gnf03.051.000 | 161.03 | 54081737 |
| 3 | rs13477127 | 178.65 | 56759332 |
| 3 | rs6363066 | 182.59 | 57627215 |
| 3 | rs13477144 | 200.45 | 61072170 |
| 3 | rs6176848 | 202.13 | 61878614 |
| 3 | rs13477154 | 202.82 | 63443860 |
| 3 | rs3674810 | 213.89 | 67946724 |
| 3 | rs13477178 | 218.69 | 69548776 |
| 3 | rs3659688 | 220.26 | 70659239 |
| 3 | rs3715136 | 221.51 | 72194392 |
| 3 | rs4137345 | 221.51 | 73027720 |
| 3 | gnf03.073.308 | 233.81 | 76037791 |
| 3 | rs3726567 | 238.25 | 77261245 |
| 3 | rs13477217 | 238.95 | 78503778 |
| 3 | rs3659866 | 243.98 | 80696460 |
| 3 | rs3685286 | 247.16 | 81299107 |
| 3 | rs13477230 | 250.12 | 82605961 |
| 3 | rs3708227 | 252.37 | 83829773 |
| 3 | rs6243021 | 255.71 | 84948288 |
| 3 | rs13477242 | 255.76 | 85887218 |
| 3 | rs13475064 | 257.54 | 88713874 |
| 3 | rs13477254 | 271.54 | 89894693 |
| 3 | UT-2-93.461287 | 273.52 | 90564824 |
| 3 | rs13477261 | 275.72 | 91922865 |
| 3 | rs13459185 | 290.34 | 95750438 |
| 3 | rs13477276 | 294.17 | 96735929 |
| 3 | rs13477279 | 297.07 | 97788738 |
| 3 | UT-3-102.173433 | 298.28 | 99156566 |
| 3 | rs3162061 | 299.43 | 100523616 |
| 3 | rs3671622 | 303.22 | 102185758 |
| 3 | rs3701653 | 304.49 | 103438050 |
| 3 | rs13477307 | 311.67 | 104372664 |
| 3 | rs13477309 | 313.35 | 105273322 |
| 3 | rs13477313 | 314.46 | 106287660 |
| 3 | rs3684333 | 314.81 | 107273619 |
| 3 | rs8256683 | 324.87 | 109782469 |
| 3 | rs3676545 | 325.47 | 110059229 |
| 3 | rs3711702 | 325.52 | 111115643 |
| 3 | gnf03.117.090 | 331.83 | 113399273 |
| 3 | rs3657795 | 331.83 | 113715227 |
| 3 | rs13477355 | 334.19 | 115813938 |
| 3 | rs13477364 | 364.02 | 118290074 |
| 3 | rs13477379 | 377.00 | 122540625 |
| 3 | rs3707706 | 385.24 | 123718240 |
| 3 | rs13477384 | 386.65 | 124511585 |
| 3 | rs8255228 | 387.11 | 125617732 |
| 3 | rs13477391 | 395.06 | 126861952 |
| 3 | rs3670168 | 395.40 | 127995452 |
| 3 | rs13477400 | 399.95 | 129089661 |
| 3 | rs3658914 | 428.72 | 130530956 |
| 3 | rs13477410 | 432.64 | 131729877 |
| 3 | rs13477421 | 448.05 | 133905167 |
| 3 | gnf03.138.849 | 450.09 | 135097436 |
| 3 | CEL-3-137067761 | 466.08 | 136180489 |
| 3 | rs13477438 | 466.18 | 137470980 |
| 3 | rs13477448 | 473.47 | 139617213 |
| 3 | rs6189225 | 474.68 | 141059766 |
| 3 | rs4224277 | 475.03 | 142235699 |
| 3 | CEL-3-145329096 | 491.32 | 144425238 |
| 3 | rs13477529 | 498.22 | 145744719 |
| 3 | rs13477477 | 509.46 | 146988827 |
| 3 | rs3722447 | 523.35 | 148608637 |
| 3 | rs13477488 | 531.83 | 150059131 |
| 3 | rs13477494 | 538.60 | 151227284 |
| 3 | rs3695139 | 540.72 | 152025332 |
| 3 | rs3695386 | 553.60 | 155445672 |
| 4 | rs13477541 | 0.00 | 6320882 |
| 4 | rs13477546 | 8.28 | 7682681 |
| 4 | rs13477550 | 11.06 | 8920439 |
| 4 | rs6324271 | 16.83 | 10242307 |
| 4 | rs13477558 | 16.86 | 10825523 |
| 4 | rs13477569 | 33.29 | 13886134 |
| 4 | rs13477576 | 35.33 | 15649658 |
| 4 | rs13477595 | 58.39 | 20446245 |
| 4 | gnf04.018.158 | 59.18 | 21343761 |
| 4 | rs13477608 | 61.28 | 23912431 |
| 4 | rs4224426 | 62.18 | 25177474 |
| 4 | rs3681849 | 62.73 | 26390959 |
| 4 | rs13477621 | 69.18 | 27943571 |
| 4 | rs13477623 | 80.08 | 29505438 |
| 4 | rs13477625 | 80.18 | 29960152 |
| 4 | CEL-4-30832818 | 80.68 | 30528618 |
| 4 | gnf04.029.494 | 83.90 | 32833342 |
| 4 | CEL-4-34055416 | 86.42 | 33911031 |
| 4 | rs3719299 | 99.34 | 36076761 |
| 4 | rs13477649 | 103.56 | 36837767 |
| 4 | rs3684104 | 114.04 | 38269952 |
| 4 | rs13477659 | 115.10 | 39159190 |
| 4 | CEL-4-40541402 | 116.67 | 40397017 |
| 4 | rs4138630 | 122.14 | 41604055 |
| 4 | rs6321462 | 122.89 | 43663720 |
| 4 | rs13477678 | 141.62 | 45502303 |
| 4 | rs3707178 | 147.72 | 47049015 |
| 4 | rs3663355 | 150.84 | 48126206 |
| 4 | rs13477694 | 158.47 | 49601465 |
| 4 | rs6269326 | 163.21 | 50499252 |
| 4 | rs13477711 | 168.63 | 53915357 |
| 4 | rs3677770 | 174.04 | 54732355 |
| 4 | rs13477725 | 197.79 | 57626582 |
| 4 | UT-4-58.126445 | 200.17 | 58386897 |
| 4 | rs3671277 | 202.76 | 59457409 |
| 4 | rs13477738 | 208.54 | 62690602 |
| 4 | rs13477741 | 208.71 | 63889889 |
| 4 | gnf04.062.327 | 214.02 | 66377278 |
| 4 | rs6370644 | 232.18 | 70203958 |
| 4 | rs13477769 | 233.09 | 72196782 |
| 4 | rs13477773 | 233.09 | 73016584 |
| 4 | rs6292114 | 235.61 | 74628116 |
| 4 | rs3708471 | 237.98 | 76516631 |
| 4 | rs3669136 | 240.09 | 78284862 |
| 4 | CEL-4-78089985 | 243.48 | 79461643 |
| 4 | rs2020477 | 244.78 | 80742555 |
| 4 | rs6323325 | 249.76 | 82052838 |
| 4 | rs3700579 | 250.16 | 83585215 |
| 4 | rs13477816 | 262.24 | 84970200 |
| 4 | rs3707373 | 270.52 | 86579087 |
| 4 | CEL-4-86185890 | 277.38 | 87557343 |
| 4 | rs13477831 | 281.10 | 88597633 |
| 4 | rs13477854 | 303.38 | 95486141 |
| 4 | CEL-4-95976899 | 311.11 | 97313314 |
| 4 | rs6255772 | 318.04 | 98540189 |
| 4 | rs13477868 | 327.02 | 99636848 |
| 4 | rs13477873 | 329.01 | 101102849 |
| 4 | rs13477876 | 329.23 | 102065882 |
| 4 | rs13477882 | 332.15 | 103321664 |
| 4 | rs13477886 | 334.31 | 104379930 |
| 4 | rs13477893 | 344.58 | 105972118 |
| 4 | rs3709496 | 351.29 | 107503962 |
| 4 | rs3670382 | 364.50 | 110513734 |
| 4 | mCV22668736 | 366.28 | 112333149 |
| 4 | rs3687391 | 373.72 | 114037342 |
| 4 | rs3022993 | 377.51 | 115159572 |
| 4 | mCV24303778 | 378.87 | 115925083 |
| 4 | rs6173859 | 390.29 | 117245337 |
| 4 | rs3678308 | 400.07 | 120416899 |
| 4 | CEL-4-120039566 | 402.55 | 121366805 |
| 4 | rs13477952 | 402.84 | 122629128 |
| 4 | rs3677161 | 413.30 | 124517596 |
| 4 | rs13474356 | 413.30 | 124745401 |
| 4 | rs13477968 | 423.52 | 127074544 |
| 4 | rs4138996 | 425.02 | 128554817 |
| 4 | rs3698956 | 429.43 | 129775759 |
| 4 | rs3673061 | 432.49 | 130913680 |
| 4 | rs4224808 | 438.39 | 132556150 |
| 4 | rs13477991 | 444.24 | 133435414 |
| 4 | rs3663950 | 462.24 | 135285446 |
| 4 | rs3023025 | 568.28 | 142772319 |
| 4 | rs4136314 | 580.08 | 144635389 |
| 4 | UT-4-146.099338 | 590.12 | 147360490 |
| 4 | UT-4-147.232882 | 591.95 | 148494034 |
| 4 | rs3720325 | 592.50 | 149136035 |
| 4 | rs13478051 | 594.60 | 150322202 |
| 4 | rs6378384 | 609.67 | 152935160 |
| 4 | rs3696703 | 627.78 | 154599781 |
| 4 | rs3720634 | 641.92 | 155281703 |
| 5 | rs13478093 | 0.00 | 3968775 |
| 5 | rs6190354 | 1.00 | 4858913 |
| 5 | rs3709946 | 1.84 | 6789766 |
| 5 | rs13478104 | 2.82 | 7975723 |
| 5 | rs6410022 | 2.87 | 8916539 |
| 5 | rs3676096 | 4.28 | 10671077 |
| 5 | rs3714258 | 11.57 | 12371156 |
| 5 | mCV25284008 | 12.78 | 13091979 |
| 5 | rs13478133 | 34.13 | 21120063 |
| 5 | rs4225096 | 46.48 | 23915346 |
| 5 | rs13478148 | 51.30 | 24860333 |
| 5 | rs3718492 | 72.84 | 32359429 |
| 5 | rs13478175 | 76.73 | 33930827 |
| 5 | rs13459085 | 76.78 | 35251425 |
| 5 | rs13478184 | 80.81 | 36393187 |
| 5 | rs13478204 | 92.13 | 41067292 |
| 5 | rs13478217 | 106.69 | 44555834 |
| 5 | rs13478223 | 115.34 | 45747535 |
| 5 | CEL-5-45872918 | 116.71 | 47638996 |
| 5 | rs6248036 | 134.59 | 53200019 |
| 5 | rs3711950 | 170.92 | 64403638 |
| 5 | rs6267669 | 188.25 | 66893863 |
| 5 | rs3684754 | 195.70 | 67751433 |
| 5 | rs3691938 | 199.53 | 68657751 |
| 5 | rs6409508 | 215.44 | 73519551 |
| 5 | gnf05.069.163 | 221.12 | 75274692 |
| 5 | rs6221589 | 223.03 | 76099099 |
| 5 | rs6257272 | 234.36 | 77316225 |
| 5 | rs3658150 | 236.56 | 80220903 |
| 5 | rs13478352 | 238.45 | 81559534 |
| 5 | rs13478355 | 241.47 | 82588916 |
| 5 | rs13478361 | 241.47 | 83810838 |
| 5 | rs3678577 | 241.63 | 85065018 |
| 5 | rs13459087 | 246.39 | 87521105 |
| 5 | rs13478383 | 250.89 | 89809980 |
| 5 | rs3716445 | 256.85 | 91079616 |
| 5 | rs13478392 | 259.65 | 92179790 |
| 5 | UT-5-94.545323 | 264.79 | 93604751 |
| 5 | rs13478400 | 266.51 | 96265315 |
| 5 | rs3661241 | 272.61 | 98266375 |
| 5 | rs6350578 | 290.45 | 106723995 |
| 5 | rs13478447 | 292.76 | 107833361 |
| 5 | rs13478451 | 296.14 | 109083780 |
| 5 | rs3658755 | 307.16 | 113197360 |
| 5 | rs13478473 | 310.11 | 114660997 |
| 5 | UT-5-114.525467 | 321.93 | 115904080 |
| 5 | gnf05.110.207 | 323.39 | 116556465 |
| 5 | mCV22554962 | 328.51 | 118017688 |
| 5 | rs3662655 | 338.05 | 118710087 |
| 5 | rs13478487 | 341.71 | 119758227 |
| 5 | rs8239888 | 364.41 | 122017884 |
| 5 | CEL-5-120064766 | 366.44 | 123000843 |
| 5 | rs13478501 | 371.39 | 124241062 |
| 5 | rs13478509 | 375.89 | 125995647 |
| 5 | rs3701266 | 386.20 | 127163995 |
| 5 | rs13478522 | 398.97 | 128978755 |
| 5 | rs6377710 | 432.68 | 132852074 |
| 5 | rs13478539 | 435.36 | 133538654 |
| 5 | rs13478540 | 449.83 | 134520936 |
| 5 | rs3023058 | 468.33 | 136305325 |
| 5 | rs3711751 | 474.92 | 137393985 |
| 5 | rs3023061 | 518.78 | 144692152 |
| 5 | mCV25009162 | 521.78 | 145297027 |
| 5 | rs4225575 | 532.56 | 145942771 |
| 5 | mCV22895274 | 534.23 | 147394031 |
| 5 | rs13478589 | 562.10 | 149012990 |
| 5 | rs3718776 | 565.43 | 150393227 |
| 5 | rs13478595 | 565.43 | 151148536 |
| 6 | rs13478602 | 0.00 | 3797192 |
| 6 | rs13478608 | 2.09 | 5219015 |
| 6 | rs13478612 | 8.02 | 6508472 |
| 6 | rs13478615 | 8.10 | 7222703 |
| 6 | CEL-6-9421791 | 19.31 | 9639647 |
| 6 | CEL-6-10519419 | 20.22 | 10733840 |
| 6 | rs13478631 | 20.52 | 11226305 |
| 6 | rs3655979 | 25.48 | 13135397 |
| 6 | rs3678711 | 29.03 | 14239843 |
| 6 | rs13478641 | 35.15 | 16011004 |
| 6 | UT-6-18.199327 | 39.92 | 18272206 |
| 6 | gnf06.016.989 | 41.01 | 19886052 |
| 6 | rs3710004 | 58.89 | 23153567 |
| 6 | rs3684494 | 63.92 | 24365693 |
| 6 | rs13478667 | 68.88 | 25547621 |
| 6 | rs13478671 | 70.40 | 26419434 |
| 6 | rs13478676 | 70.57 | 27605708 |
| 6 | rs13478681 | 71.80 | 28754995 |
| 6 | rs13478693 | 73.63 | 32013004 |
| 6 | rs6303641 | 75.16 | 33463435 |
| 6 | rs6297560 | 76.84 | 34603323 |
| 6 | gnf06.032.524 | 76.94 | 35481775 |
| 6 | gnf06.033.454 | 79.25 | 36435340 |
| 6 | rs13478717 | 85.43 | 37639577 |
| 6 | rs13478719 | 88.27 | 38495339 |
| 6 | gnf06.037.785 | 103.45 | 40749295 |
| 6 | rs13478726 | 111.37 | 42352269 |
| 6 | rs13478727 | 112.27 | 43776811 |
| 6 | rs13478730 | 117.00 | 44976417 |
| 6 | rs3684860 | 142.89 | 48936035 |
| 6 | rs3023069 | 162.89 | 52193140 |
| 6 | rs13478759 | 163.23 | 53330356 |
| 6 | rs13478762 | 192.08 | 54175670 |
| 6 | gnf06.058.959 | 201.65 | 59756735 |
| 6 | rs6378343 | 214.02 | 63773077 |
| 6 | rs8270116 | 215.90 | 65067887 |
| 6 | rs13478818 | 233.11 | 73053161 |
| 6 | rs6411497 | 234.22 | 75464921 |
| 6 | rs13478833 | 236.37 | 76580662 |
| 6 | rs13478839 | 250.07 | 77761517 |
| 6 | rs13478845 | 254.33 | 78947220 |
| 6 | rs13478853 | 257.81 | 80525800 |
| 6 | rs4226024 | 262.70 | 81908219 |
| 6 | CEL-6-83434907 | 269.00 | 83147368 |
| 6 | rs13459097 | 274.47 | 84840296 |
| 6 | CEL-6-86437630 | 274.61 | 86058922 |
| 6 | rs3677567 | 274.91 | 87222078 |
| 6 | rs13478880 | 278.13 | 88537081 |
| 6 | rs13478882 | 278.83 | 89615026 |
| 6 | rs6156752 | 286.00 | 90723697 |
| 6 | rs6223362 | 302.18 | 92570487 |
| 6 | gnf06.092.758 | 302.93 | 93813955 |
| 6 | gnf06.093.201 | 305.02 | 94255481 |
| 6 | rs13478949 | 354.89 | 105083653 |
| 6 | rs13478952 | 356.15 | 106255497 |
| 6 | gnf06.105.887 | 356.90 | 107015261 |
| 6 | gnf06.107.451 | 363.14 | 108599680 |
| 6 | rs13478965 | 363.19 | 109407240 |
| 6 | rs13478969 | 372.51 | 110706035 |
| 6 | rs13478974 | 382.67 | 111930722 |
| 6 | rs6393943 | 385.52 | 114357238 |
| 6 | rs6204829 | 391.31 | 115865552 |
| 6 | mCV23042866 | 391.74 | 116706746 |
| 6 | rs13478999 | 402.16 | 118752027 |
| 6 | rs3722157 | 414.81 | 122368125 |
| 6 | gnf06.122.747 | 419.95 | 124006512 |
| 6 | UT-6-127.071239 | 427.42 | 125094389 |
| 6 | CEL-6-127435117 | 439.90 | 126754347 |
| 6 | rs13479024 | 442.80 | 127541604 |
| 6 | rs3662241 | 446.03 | 129560177 |
| 6 | rs3654483 | 446.03 | 130435892 |
| 6 | rs3721822 | 446.03 | 131258177 |
| 6 | rs3704289 | 449.26 | 131940471 |
| 6 | CEL-6-132620146 | 450.12 | 132498747 |
| 6 | mCV22521691 | 450.12 | 132813398 |
| 6 | rs3655878 | 451.05 | 133582267 |
| 6 | rs13479051 | 461.29 | 133804968 |
| 6 | rs6339546 | 463.34 | 133917751 |
| 6 | rs13479053 | 465.49 | 134201251 |
| 6 | rs13479055 | 465.54 | 134571804 |
| 6 | rs3671932 | 467.37 | 134808128 |
| 6 | rs13479058 | 471.15 | 135272117 |
| 6 | rs13479059 | 471.15 | 135610188 |
| 6 | rs3711652 | 472.11 | 135928283 |
| 6 | rs3704502 | 472.26 | 136207977 |
| 6 | rs13479064 | 472.81 | 136560768 |
| 6 | rs13479066 | 473.16 | 136889372 |
| 6 | rs13479070 | 475.37 | 137928427 |
| 6 | rs13479071 | 475.37 | 138203532 |
| 6 | rs6261913 | 475.41 | 138598188 |
| 6 | rs6255954 | 481.33 | 139010268 |
| 6 | gnf06.139.257 | 481.63 | 139126179 |
| 6 | rs6288584 | 482.54 | 139468253 |
| 6 | rs3672808 | 482.54 | 139805730 |
| 6 | rs3664540 | 482.74 | 140066610 |
| 6 | rs6199136 | 483.54 | 140387757 |
| 6 | rs8268650 | 484.70 | 141480729 |
| 6 | rs6329892 | 484.75 | 142368838 |
| 6 | rs6235887 | 484.95 | 142672872 |
| 6 | rs6391555 | 485.55 | 142951483 |
| 6 | rs4226359 | 485.80 | 143246238 |
| 6 | rs6152631 | 489.37 | 144002632 |
| 6 | rs6386657 | 489.72 | 144262633 |
| 6 | rs13479084 | 492.39 | 144453463 |
| 6 | rs4140069 | 499.94 | 144814489 |
| 6 | rs13459098 | 500.64 | 145123190 |
| 6 | rs13479086 | 500.69 | 145244335 |
| 6 | rs6387265 | 504.34 | 145693849 |
| 6 | rs3023105 | 510.18 | 146425319 |
| 6 | rs3712253 | 510.18 | 146691561 |
| 6 | rs13479099 | 510.18 | 147147748 |
| 6 | rs6265387 | 510.18 | 147203378 |
| 7 | gnf07.010.101 | 0.00 | 16488106 |
| 7 | rs6295100 | 5.83 | 17046685 |
| 7 | UT-7-14.584506 | 10.10 | 18266812 |
| 7 | UT-7-16.347574 | 10.19 | 19942044 |
| 7 | CEL-7-12787527 | 16.95 | 24837175 |
| 7 | rs13479154 | 27.90 | 26076273 |
| 7 | rs13479163 | 43.29 | 27852866 |
| 7 | rs13479171 | 59.97 | 29935213 |
| 7 | rs13479174 | 75.55 | 30807922 |
| 7 | CEL-7-20534424 | 92.95 | 32294980 |
| 7 | CEL-7-21610843 | 93.18 | 34313345 |
| 7 | rs3694031 | 101.84 | 35699898 |
| 7 | rs6217275 | 122.61 | 37211722 |
| 7 | gnf07.032.360 | 136.24 | 47652851 |
| 7 | rs4232449 | 145.17 | 48581740 |
| 7 | rs6316536 | 147.53 | 50759749 |
| 7 | rs3719256 | 152.54 | 51794017 |
| 7 | rs3689409 | 161.41 | 52261292 |
| 7 | rs3717293 | 198.92 | 63232349 |
| 7 | rs3696018 | 201.76 | 64362355 |
| 7 | rs13479276 | 201.79 | 65717523 |
| 7 | rs3693478 | 212.68 | 67179979 |
| 7 | gnf07.056.997 | 212.82 | 69695364 |
| 7 | rs3679779 | 214.71 | 71545170 |
| 7 | rs6160140 | 222.09 | 73426174 |
| 7 | rs3667441 | 225.24 | 74778390 |
| 7 | rs6296859 | 228.96 | 76126377 |
| 7 | rs3723790 | 238.60 | 77309844 |
| 7 | rs13479325 | 248.05 | 78443151 |
| 7 | rs3676254 | 252.49 | 79831151 |
| 7 | mCV25303361 | 260.28 | 80754000 |
| 7 | rs13479342 | 271.45 | 82114281 |
| 7 | rs13479347 | 278.73 | 83432559 |
| 7 | rs13479351 | 279.99 | 84643102 |
| 7 | rs3686423 | 287.84 | 86728144 |
| 7 | rs8248433 | 295.46 | 87647472 |
| 7 | rs3663343 | 299.22 | 88684139 |
| 7 | rs6213614 | 302.65 | 89430611 |
| 7 | rs13479376 | 307.33 | 91596873 |
| 7 | CEL-7-73288107 | 307.38 | 93275341 |
| 7 | rs13479385 | 310.34 | 94282690 |
| 7 | rs3663323 | 311.55 | 95460791 |
| 7 | rs13479393 | 316.12 | 96556405 |
| 7 | CEL-7-77948585 | 316.56 | 97864533 |
| 7 | rs3663374 | 323.36 | 98602160 |
| 7 | rs3683030 | 333.90 | 100241831 |
| 7 | rs6224196 | 338.82 | 102315605 |
| 7 | UT-7-90.803899 | 344.37 | 106485053 |
| 7 | rs13479427 | 345.07 | 107177259 |
| 7 | rs13479429 | 346.03 | 108319539 |
| 7 | rs3713052 | 346.98 | 108918190 |
| 7 | rs13479437 | 348.66 | 110335247 |
| 7 | rs3662937 | 348.66 | 111381490 |
| 7 | rs13459173 | 352.10 | 112784815 |
| 7 | rs3711721 | 354.04 | 114369537 |
| 7 | rs6315049 | 355.35 | 116196648 |
| 7 | rs13479457 | 359.46 | 117574875 |
| 7 | rs13479461 | 366.66 | 119092066 |
| 7 | rs3726290 | 368.03 | 120495850 |
| 7 | rs13479470 | 370.29 | 121842519 |
| 7 | rs3656074 | 378.06 | 123277780 |
| 7 | gnf07.120.460 | 439.62 | 130717933 |
| 7 | rs13479507 | 453.32 | 132034868 |
| 7 | rs6403908 | 454.25 | 133241749 |
| 7 | rs6177803 | 460.53 | 134468592 |
| 7 | CEL-7-116160192 | 468.96 | 135825551 |
| 7 | rs8236684 | 482.94 | 138174654 |
| 7 | CEL-7-126301023 | 561.87 | 145854915 |
| 7 | rs6299045 | 566.93 | 147339788 |
| 7 | rs3702894 | 576.24 | 149537767 |
| 7 | rs6176332 | 580.22 | 150663801 |
| 7 | rs6216320 | 585.41 | 152367605 |
| 8 | rs6273176 | 0.00 | 7850107 |
| 8 | rs13479601 | 1.59 | 9055823 |
| 8 | rs6288205 | 27.23 | 12246849 |
| 8 | rs6287472 | 27.24 | 13314068 |
| 8 | rs6410533 | 30.63 | 14301463 |
| 8 | rs13479624 | 41.09 | 16859147 |
| 8 | rs13479628 | 49.83 | 18316446 |
| 8 | CEL-8-25677705 | 87.78 | 28002388 |
| 8 | rs13479769 | 331.66 | 55686243 |
| 8 | rs3659789 | 331.75 | 58485096 |
| 8 | UT-8-57.168981 | 332.22 | 59934830 |
| 8 | rs13479784 | 336.58 | 61240020 |
| 8 | rs6398181 | 338.57 | 62694477 |
| 8 | rs3672639 | 338.72 | 63700583 |
| 8 | rs6394046 | 348.58 | 64982540 |
| 8 | rs13479799 | 349.42 | 66039644 |
| 8 | gnf08.064.413 | 349.42 | 67369114 |
| 8 | rs3694940 | 349.72 | 68395872 |
| 8 | rs13479811 | 353.55 | 69577204 |
| 8 | rs13479814 | 355.02 | 70845435 |
| 8 | rs3698093 | 379.91 | 80549233 |
| 8 | rs6296891 | 393.92 | 82507845 |
| 8 | rs3696786 | 397.42 | 83525688 |
| 8 | rs13479860 | 400.97 | 84785535 |
| 8 | rs4227283 | 406.72 | 85796532 |
| 8 | UT-8-84.8318 | 409.39 | 86493714 |
| 8 | rs13479873 | 416.65 | 87754611 |
| 8 | rs13479879 | 421.15 | 89032866 |
| 8 | rs3682243 | 423.13 | 89857454 |
| 8 | rs3705695 | 458.69 | 97880021 |
| 8 | rs6374927 | 564.34 | 114155493 |
| 8 | rs3675125 | 722.30 | 124773828 |
| 8 | rs13480022 | 726.41 | 124978927 |
| 8 | rs13480023 | 728.03 | 125286790 |
| 8 | rs13480024 | 728.08 | 125418137 |
| 8 | rs3693295 | 728.08 | 125471593 |
| 8 | rs4227429 | 728.08 | 125792617 |
| 8 | rs8249856 | 731.84 | 126493954 |
| 8 | rs8238636 | 731.92 | 127080944 |
| 8 | rs6300613 | 733.39 | 127971008 |
| 8 | UT-8-130.396331 | 740.58 | 131027084 |
| 8 | rs3695597 | 740.62 | 131457798 |
| 9 | rs13480065 | 0.00 | 9290507 |
| 9 | mCV25073238 | 9.57 | 10573030 |
| 9 | rs13480073 | 17.97 | 13233763 |
| 9 | gnf09.009.970 | 22.85 | 15496546 |
| 9 | rs13480112 | 99.52 | 26413932 |
| 9 | rs6182405 | 101.72 | 27652042 |
| 9 | petM-05537-1 | 124.80 | 28930590 |
| 9 | CEL-9-29909656 | 129.24 | 29748545 |
| 9 | rs13480130 | 153.57 | 33099089 |
| 9 | rs3711756 | 156.96 | 34266429 |
| 9 | rs13480138 | 164.31 | 35323696 |
| 9 | mCV25302097 | 164.33 | 36242205 |
| 9 | rs3719607 | 164.42 | 37007430 |
| 9 | rs3694949 | 164.57 | 38263146 |
| 9 | rs3675289 | 165.17 | 39517700 |
| 9 | rs13480153 | 169.12 | 40483617 |
| 9 | rs3676086 | 187.77 | 42649933 |
| 9 | rs13480166 | 197.43 | 43879831 |
| 9 | rs13480172 | 199.51 | 46045437 |
| 9 | rs3673816 | 201.03 | 47039895 |
| 9 | rs13480180 | 210.98 | 48228876 |
| 9 | gnf09.044.276 | 213.29 | 49784973 |
| 9 | rs13480191 | 228.04 | 50964758 |
| 9 | rs13480194 | 239.26 | 52120901 |
| 9 | rs13480198 | 244.74 | 53090229 |
| 9 | rs8259443 | 252.02 | 54013403 |
| 9 | rs3660104 | 253.26 | 54832009 |
| 9 | rs6334600 | 256.12 | 56387055 |
| 9 | rs13480218 | 268.05 | 57836654 |
| 9 | rs6154433 | 269.45 | 59038347 |
| 9 | rs13480227 | 288.45 | 60898609 |
| 9 | rs13480232 | 298.04 | 62013984 |
| 9 | rs13480236 | 300.99 | 63055796 |
| 9 | rs3664300 | 301.59 | 64300970 |
| 9 | rs13480247 | 312.28 | 65394048 |
| 9 | rs3670579 | 314.64 | 66754573 |
| 9 | rs3716689 | 317.00 | 67961641 |
| 9 | rs13480258 | 330.65 | 68739815 |
| 9 | rs3703045 | 332.58 | 70160776 |
| 9 | rs13459110 | 340.29 | 71331038 |
| 9 | rs3711813 | 340.29 | 72210393 |
| 9 | rs13480273 | 348.73 | 73120560 |
| 9 | rs3724833 | 365.30 | 74735877 |
| 9 | mCV25328470 | 365.48 | 75892111 |
| 9 | rs13480288 | 366.39 | 77247379 |
| 9 | rs3658458 | 370.38 | 80151280 |
| 9 | rs3670195 | 371.49 | 81159485 |
| 9 | rs13480312 | 385.83 | 83687617 |
| 9 | rs13480315 | 387.58 | 84867140 |
| 9 | rs13480318 | 389.39 | 86201201 |
| 9 | rs6182207 | 391.86 | 87332104 |
| 9 | gnf09.087.298 | 396.75 | 90578037 |
| 9 | rs6202847 | 399.49 | 91592508 |
| 9 | CEL-9-92503233 | 401.17 | 92554642 |
| 9 | rs13480351 | 403.91 | 94179227 |
| 9 | rs3690580 | 411.22 | 95251199 |
| 9 | rs13480367 | 414.16 | 98823436 |
| 9 | gnf09.096.289 | 415.42 | 99654175 |
| 9 | rs6190068 | 426.29 | 101521003 |
| 9 | rs3657346 | 428.96 | 103073500 |
| 9 | rs13480387 | 429.11 | 103827403 |
| 9 | rs3711089 | 439.12 | 105393992 |
| 9 | rs6320810 | 530.99 | 115065091 |
| 9 | rs3669563 | 535.95 | 117827881 |
| 9 | rs6316481 | 548.42 | 118883562 |
| 9 | rs13480454 | 559.21 | 120606956 |
| 9 | rs6299531 | 565.59 | 122834340 |
| 9 | rs8241505 | 568.86 | 123699420 |
| 9 | rs8254378 | 575.31 | 124039192 |
| 10 | rs13480510 | 0.00 | 15801686 |
| 10 | rs13480516 | 2.04 | 17233244 |
| 10 | rs3703211 | 3.92 | 18256741 |
| 10 | rs13480527 | 9.67 | 19336109 |
| 10 | rs13480531 | 18.04 | 20493290 |
| 10 | rs13480536 | 20.13 | 21653710 |
| 10 | rs3679120 | 29.86 | 22641084 |
| 10 | rs13480547 | 36.80 | 24207368 |
| 10 | rs3672342 | 37.10 | 25413902 |
| 10 | rs13459121 | 44.86 | 26517513 |
| 10 | rs3023233 | 65.73 | 28315852 |
| 10 | rs13459120 | 91.41 | 35758852 |
| 10 | rs13480601 | 150.04 | 44026225 |
| 10 | rs6212358 | 153.55 | 45838855 |
| 10 | rs3676667 | 154.00 | 46672387 |
| 10 | rs3715820 | 154.30 | 48283697 |
| 10 | rs6164020 | 154.40 | 49468021 |
| 10 | rs6186864 | 158.34 | 52447434 |
| 10 | rs6243167 | 161.46 | 54504265 |
| 10 | rs6374078 | 184.90 | 60568067 |
| 10 | rs6312070 | 188.22 | 62537450 |
| 10 | rs6335027 | 197.27 | 64823642 |
| 10 | rs13480630 | 205.25 | 67283840 |
| 10 | rs13480638 | 216.25 | 68907415 |
| 10 | rs6295890 | 223.26 | 72110546 |
| 10 | rs13480652 | 236.04 | 74075171 |
| 10 | rs13480678 | 255.89 | 84148125 |
| 10 | rs13480684 | 261.83 | 85335328 |
| 10 | rs13480687 | 271.82 | 86559223 |
| 10 | gnf10.087.008 | 272.78 | 87488435 |
| 10 | rs3701829 | 290.55 | 88796791 |
| 10 | mCV25373751 | 291.05 | 90171935 |
| 10 | rs13480703 | 295.03 | 90880555 |
| 10 | rs3661495 | 302.81 | 92120896 |
| 10 | rs13480712 | 307.52 | 92978979 |
| 10 | rs3704401 | 330.44 | 98705184 |
| 10 | rs6390581 | 330.69 | 99291225 |
| 10 | rs3710293 | 331.50 | 99577432 |
| 10 | rs6185093 | 332.00 | 100205724 |
| 10 | rs13480734 | 338.29 | 101838025 |
| 10 | rs13480739 | 342.50 | 103185335 |
| 10 | rs13480740 | 342.50 | 103515831 |
| 10 | rs3688351 | 343.11 | 103953112 |
| 10 | rs3716899 | 343.11 | 104406107 |
| 10 | rs13480745 | 343.11 | 104621894 |
| 10 | rs3716443 | 343.11 | 104949412 |
| 10 | rs13480749 | 344.31 | 105437650 |
| 10 | rs13480752 | 345.62 | 106267142 |
| 10 | rs3654717 | 349.20 | 106641086 |
| 10 | rs13480754 | 349.43 | 106921183 |
| 10 | rs6174062 | 353.82 | 108964735 |
| 10 | rs6293699 | 354.93 | 109577883 |
| 10 | rs4228452 | 355.78 | 110731307 |
| 10 | rs6353445 | 357.20 | 111131157 |
| 10 | rs13480766 | 364.52 | 111320457 |
| 10 | CEL-10-113177617 | 370.04 | 112836909 |
| 10 | rs13480773 | 378.71 | 114179164 |
| 10 | rs6201222 | 378.71 | 114310930 |
| 10 | rs4228477 | 378.71 | 114515890 |
| 10 | rs6363315 | 393.75 | 116291531 |
| 10 | CZECH-10-116791624 | 393.75 | 116433049 |
| 10 | rs6335076 | 393.75 | 116582756 |
| 10 | rs3670118 | 394.40 | 116830926 |
| 10 | rs6256918 | 394.65 | 116988751 |
| 10 | rs6284081 | 396.01 | 117560628 |
| 10 | rs6317716 | 402.18 | 118914359 |
| 10 | rs13480792 | 409.63 | 119844877 |
| 10 | rs6197175 | 415.58 | 120131018 |
| 10 | rs13480793 | 416.73 | 120465110 |
| 10 | rs6237927 | 430.43 | 120809086 |
| 10 | rs6197961 | 437.08 | 121298514 |
| 10 | rs13480797 | 437.08 | 121364788 |
| 10 | rs13480808 | 459.95 | 123833688 |
| 10 | rs4140243 | 461.78 | 124274419 |
| 10 | rs13480811 | 461.78 | 124544360 |
| 10 | gnf10.124.898 | 464.31 | 124928136 |
| 10 | rs6281696 | 465.06 | 125024086 |
| 10 | rs3697243 | 468.89 | 126250066 |
| 10 | rs13480821 | 473.35 | 126934967 |
| 10 | rs6290842 | 480.01 | 128553075 |
| 11 | rs13480835 | 0.00 | 3234216 |
| 11 | rs3682937 | 0.34 | 4580335 |
| 11 | rs6393401 | 3.24 | 5771105 |
| 11 | rs13480853 | 10.30 | 7315081 |
| 11 | rs13480859 | 11.60 | 8378425 |
| 11 | rs3661631 | 13.36 | 9234548 |
| 11 | rs13480869 | 15.65 | 10903789 |
| 11 | rs13480880 | 26.72 | 13332444 |
| 11 | rs3689494 | 38.87 | 16181190 |
| 11 | rs3658216 | 68.30 | 19463109 |
| 11 | rs6393028 | 77.81 | 20753809 |
| 11 | rs3154937 | 79.00 | 21470891 |
| 11 | rs3661074 | 82.40 | 22652479 |
| 11 | rs13480913 | 82.40 | 23633252 |
| 11 | rs13480918 | 96.43 | 25018970 |
| 11 | rs3719024 | 103.05 | 26370845 |
| 11 | rs6276300 | 107.85 | 27148056 |
| 11 | UT-11-29.443258 | 117.51 | 29445002 |
| 11 | rs3707274 | 128.37 | 31821623 |
| 11 | rs3710499 | 132.48 | 33611127 |
| 11 | rs3690160 | 163.56 | 36828565 |
| 11 | rs13480993 | 177.74 | 42730880 |
| 11 | gnf11.045.072 | 178.34 | 43256667 |
| 11 | rs3660692 | 195.35 | 46029522 |
| 11 | rs6250130 | 206.18 | 47289620 |
| 11 | rs6326787 | 213.26 | 48524313 |
| 11 | rs6199956 | 222.16 | 50558769 |
| 11 | rs13481023 | 223.99 | 51824799 |
| 11 | rs6398148 | 228.65 | 52902235 |
| 11 | rs13481031 | 229.96 | 54232093 |
| 11 | rs3684076 | 240.58 | 56224409 |
| 11 | rs3023260 | 252.46 | 57088038 |
| 11 | rs3697686 | 255.46 | 58381052 |
| 11 | rs13481050 | 265.58 | 60567565 |
| 11 | rs3675335 | 270.30 | 62292218 |
| 11 | rs3714311 | 282.96 | 64084880 |
| 11 | rs13481068 | 289.20 | 64596125 |
| 11 | rs13481075 | 292.80 | 66316215 |
| 11 | rs3658906 | 296.63 | 68922568 |
| 11 | UT-11-70.128831 | 303.20 | 70371167 |
| 11 | rs13481090 | 307.40 | 71222163 |
| 11 | rs13481093 | 312.65 | 72364012 |
| 11 | CEL-11-73459270 | 316.36 | 73660216 |
| 11 | rs13481102 | 316.71 | 74873177 |
| 11 | UT-11-75.985289 | 316.81 | 76227625 |
| 11 | rs6384104 | 317.46 | 77606242 |
| 11 | rs6381209 | 320.09 | 78670070 |
| 11 | rs6379880 | 320.69 | 79608084 |
| 11 | rs6178421 | 328.32 | 80819720 |
| 11 | rs13481123 | 335.65 | 82070986 |
| 11 | rs13481127 | 343.47 | 83226031 |
| 11 | rs3661657 | 346.09 | 84545869 |
| 11 | rs13481135 | 346.89 | 85660691 |
| 11 | rs3719581 | 347.67 | 86772383 |
| 11 | gnf11.093.966 | 347.88 | 87177234 |
| 11 | rs3688955 | 363.10 | 90397848 |
| 11 | rs13481161 | 378.06 | 92322572 |
| 11 | rs3656982 | 381.07 | 93517064 |
| 11 | rs3710148 | 382.38 | 96386745 |
| 11 | rs3686162 | 386.71 | 98005075 |
| 11 | rs13474433 | 387.24 | 98913418 |
| 11 | rs3695865 | 409.14 | 101540306 |
| 11 | rs6180460 | 412.63 | 102815398 |
| 11 | rs6407687 | 451.16 | 105127376 |
| 11 | rs13481210 | 452.88 | 106174196 |
| 11 | rs6376890 | 457.40 | 107458251 |
| 11 | rs13481220 | 462.09 | 108381228 |
| 11 | rs3683086 | 462.14 | 109194240 |
| 11 | rs13481228 | 481.48 | 110456691 |
| 11 | rs13481233 | 485.67 | 111663109 |
| 11 | gnf11.121.400 | 506.80 | 112504632 |
| 11 | rs3662930 | 523.41 | 115383357 |
| 11 | gnf11.125.992 | 547.52 | 117108177 |
| 11 | CEL-11-118234030 | 556.69 | 118424214 |
| 12 | rs3699421 | 0.00 | 4057347 |
| 12 | rs6278204 | 1.47 | 6055338 |
| 12 | rs13481288 | 1.97 | 7793944 |
| 12 | rs3089046 | 2.66 | 8976679 |
| 12 | rs6209157 | 7.72 | 10580141 |
| 12 | rs3718992 | 8.27 | 12000845 |
| 12 | rs13481308 | 16.11 | 13333753 |
| 12 | rs6403731 | 16.41 | 14935382 |
| 12 | rs3717860 | 29.26 | 25413845 |
| 12 | rs6328018 | 34.88 | 26818221 |
| 12 | rs13481361 | 35.63 | 28250147 |
| 12 | UT-12-24.561109 | 72.41 | 30605487 |
| 12 | rs13481375 | 76.51 | 31803096 |
| 12 | rs6390855 | 78.92 | 32159391 |
| 12 | rs6223000 | 83.03 | 34867609 |
| 12 | gnf12.033.545 | 89.14 | 35806047 |
| 12 | rs3695382 | 103.82 | 37051243 |
| 12 | rs3088766 | 108.95 | 38515357 |
| 12 | rs13481408 | 115.85 | 41183035 |
| 12 | rs13481412 | 117.89 | 42722659 |
| 12 | CEL-12-38970880 | 119.46 | 44997964 |
| 12 | rs6317361 | 136.45 | 48451441 |
| 12 | rs6170134 | 140.44 | 49631172 |
| 12 | rs13481450 | 144.95 | 52491342 |
| 12 | rs3670749 | 145.00 | 54060261 |
| 12 | rs3700857 | 146.27 | 55020886 |
| 12 | gnf12.053.286 | 156.14 | 56092725 |
| 12 | rs3688680 | 160.86 | 57737098 |
| 12 | rs3719306 | 165.08 | 58773265 |
| 12 | rs3662939 | 178.39 | 59980759 |
| 12 | rs6287262 | 181.78 | 61268162 |
| 12 | rs3660822 | 187.15 | 62707394 |
| 12 | mCV24690992 | 187.15 | 64056443 |
| 12 | rs13481499 | 189.25 | 65488803 |
| 12 | rs3686891 | 192.10 | 66581575 |
| 12 | rs13481511 | 206.54 | 69125068 |
| 12 | rs13481514 | 209.11 | 70704785 |
| 12 | rs6335879 | 223.30 | 73020609 |
| 12 | rs3687032 | 228.14 | 74599410 |
| 12 | rs3709008 | 234.91 | 75782538 |
| 12 | rs13481538 | 241.14 | 76993654 |
| 12 | rs3682382 | 246.32 | 77400088 |
| 12 | rs3696769 | 254.35 | 80840850 |
| 12 | rs3654718 | 258.07 | 82075701 |
| 12 | rs13481561 | 268.55 | 83267516 |
| 12 | rs6263380 | 269.54 | 83911344 |
| 12 | rs13481571 | 289.34 | 87284801 |
| 12 | rs13481575 | 294.77 | 88620659 |
| 12 | rs3711162 | 309.76 | 90384550 |
| 12 | rs6288403 | 314.89 | 92209033 |
| 12 | rs13481592 | 323.10 | 94194942 |
| 12 | rs6207869 | 323.15 | 95601010 |
| 12 | rs3679514 | 336.55 | 99200775 |
| 12 | rs13481609 | 343.02 | 100501462 |
| 12 | rs6184745 | 346.38 | 102084079 |
| 12 | rs3700012 | 347.17 | 102997877 |
| 12 | rs13481618 | 351.05 | 103766711 |
| 12 | rs3724260 | 374.25 | 105957285 |
| 12 | rs13481632 | 381.98 | 107634513 |
| 12 | rs13481636 | 384.66 | 108786162 |
| 12 | rs13481641 | 393.56 | 109609891 |
| 12 | CEL-12-104545022 | 401.01 | 111035058 |
| 12 | rs13481651 | 403.75 | 112559305 |
| 12 | rs4229612 | 411.20 | 114496841 |
| 12 | rs3709829 | 411.74 | 115578792 |
| 12 | rs3654706 | 416.18 | 118319943 |
| 12 | rs3711281 | 418.92 | 119427047 |
| 12 | rs3679276 | 420.08 | 121123404 |
| 13 | rs6215262 | 0.00 | 3783664 |
| 13 | rs3695486 | 0.00 | 4160600 |
| 13 | rs13481669 | 0.00 | 4598037 |
| 13 | rs3686663 | 0.00 | 4915954 |
| 13 | rs6298246 | 0.00 | 5337650 |
| 13 | rs6318987 | 0.00 | 5977104 |
| 13 | rs6243819 | 0.10 | 7110520 |
| 13 | rs6343634 | 0.10 | 7287713 |
| 13 | rs6301008 | 0.10 | 7702924 |
| 13 | rs6239339 | 0.90 | 7998546 |
| 13 | rs6250327 | 0.90 | 8305113 |
| 13 | rs6396465 | 0.90 | 8777091 |
| 13 | rs6178370 | 0.90 | 9016559 |
| 13 | rs6329684 | 0.90 | 10065064 |
| 13 | rs13481689 | 8.63 | 10826261 |
| 13 | rs13481702 | 18.56 | 14848365 |
| 13 | rs13481706 | 35.34 | 16432650 |
| 13 | rs3678616 | 35.39 | 17380921 |
| 13 | gnf13.016.175 | 36.40 | 18955999 |
| 13 | rs6314295 | 38.39 | 20213058 |
| 13 | gnf13.018.407 | 39.91 | 21233634 |
| 13 | rs13481727 | 44.08 | 22255951 |
| 13 | gnf13.020.621 | 49.50 | 23480120 |
| 13 | rs6297873 | 53.33 | 25757099 |
| 13 | rs4229685 | 99.77 | 35965561 |
| 13 | rs6200652 | 106.58 | 37063969 |
| 13 | rs3724709 | 116.04 | 38329668 |
| 13 | gnf13.038.133 | 140.43 | 40134142 |
| 13 | rs13481777 | 147.66 | 40737156 |
| 13 | rs6271232 | 156.69 | 42551106 |
| 13 | rs13481789 | 161.06 | 43920412 |
| 13 | gnf13.042.658 | 161.06 | 44677950 |
| 13 | rs3688207 | 171.60 | 45454657 |
| 13 | rs6244558 | 174.21 | 47911359 |
| 13 | rs3699522 | 186.31 | 53085960 |
| 13 | rs8273881 | 201.27 | 55500311 |
| 13 | rs13481823 | 206.68 | 57139115 |
| 13 | gnf13.057.762 | 219.94 | 59805606 |
| 13 | rs6410679 | 223.60 | 61338453 |
| 13 | rs13481849 | 225.54 | 63809792 |
| 13 | rs3720797 | 227.11 | 64854223 |
| 13 | rs4229817 | 230.77 | 66981471 |
| 13 | rs3718727 | 238.16 | 69468028 |
| 13 | rs6381045 | 245.55 | 70429182 |
| 13 | rs3658685 | 249.64 | 71837347 |
| 13 | rs3688781 | 252.11 | 72990750 |
| 13 | rs3721965 | 261.09 | 74125404 |
| 13 | rs8274592 | 261.24 | 75230587 |
| 13 | rs13481886 | 261.94 | 76267668 |
| 13 | rs13481892 | 264.73 | 77537621 |
| 13 | rs13481897 | 267.04 | 78635221 |
| 13 | rs3705043 | 267.04 | 79536468 |
| 13 | rs3693887 | 267.69 | 80749451 |
| 13 | rs13481905 | 269.31 | 81246254 |
| 13 | mCV24625340 | 284.80 | 84841126 |
| 13 | rs13481919 | 288.69 | 85822888 |
| 13 | rs6296621 | 291.71 | 87102770 |
| 13 | gnf13.092.499 | 328.64 | 92011615 |
| 13 | rs13481947 | 348.27 | 94443434 |
| 13 | rs3697202 | 348.27 | 94569175 |
| 13 | rs13481958 | 359.17 | 97286533 |
| 13 | rs13481964 | 361.05 | 98551175 |
| 13 | gnf13.099.677 | 363.58 | 99793980 |
| 13 | rs3088752 | 368.26 | 100732161 |
| 13 | rs3688959 | 393.87 | 103899453 |
| 13 | rs6389588 | 393.92 | 104624703 |
| 13 | rs13481990 | 401.32 | 105824824 |
| 13 | rs13482000 | 410.31 | 108361817 |
| 13 | mCV24886326 | 434.34 | 111386024 |
| 13 | rs6247696 | 473.28 | 115957250 |
| 13 | rs13482028 | 477.17 | 117040319 |
| 13 | rs13482032 | 479.41 | 118129309 |
| 13 | rs3708633 | 495.30 | 119532056 |
| 13 | rs6397687 | 495.59 | 120179541 |
| 13 | rs3675054 | 496.73 | 120203851 |
| 14 | rs3687889 | 0.00 | 20508439 |
| 14 | rs6397486 | 4.97 | 21756074 |
| 14 | rs3690631 | 9.71 | 22571473 |
| 14 | rs3658866 | 35.04 | 23778000 |
| 14 | rs13482143 | 133.03 | 40378547 |
| 14 | rs6314716 | 134.08 | 41430491 |
| 14 | gnf14.040.022 | 147.90 | 47467188 |
| 14 | rs4230315 | 153.60 | 48876118 |
| 14 | rs13482174 | 156.60 | 50203389 |
| 14 | rs4197422 | 157.40 | 51202617 |
| 14 | rs3680568 | 158.42 | 52756514 |
| 14 | rs3140262 | 159.37 | 54416362 |
| 14 | rs13482191 | 159.95 | 55265183 |
| 14 | rs8244195 | 170.13 | 56589968 |
| 14 | mCV23128760 | 185.45 | 59729143 |
| 14 | rs13482206 | 186.09 | 59956945 |
| 14 | rs3701623 | 258.73 | 73597329 |
| 14 | rs13482254 | 259.37 | 74915869 |
| 14 | rs13482256 | 259.42 | 75621182 |
| 14 | rs13482262 | 271.56 | 78358585 |
| 14 | rs3706792 | 279.48 | 79390535 |
| 14 | rs6352085 | 280.08 | 80081344 |
| 14 | rs13482269 | 280.13 | 81304852 |
| 14 | rs13482272 | 282.23 | 82709164 |
| 14 | rs13482276 | 285.39 | 84040443 |
| 14 | rs13482281 | 285.98 | 85687812 |
| 14 | rs3663148 | 294.34 | 87234347 |
| 14 | rs13482290 | 294.48 | 88110766 |
| 14 | rs13482296 | 294.48 | 89316099 |
| 14 | rs3667933 | 298.82 | 90486236 |
| 14 | rs6407863 | 298.82 | 91733975 |
| 14 | rs4139735 | 302.53 | 93007407 |
| 14 | rs3718262 | 302.68 | 93929573 |
| 14 | rs6211694 | 419.25 | 109093071 |
| 14 | rs13482366 | 419.25 | 110287350 |
| 14 | rs13482372 | 424.44 | 111514481 |
| 14 | rs3683221 | 427.71 | 112960486 |
| 14 | rs13482386 | 438.25 | 116410091 |
| 14 | CEL-14-110783830 | 452.75 | 118861654 |
| 14 | rs3707842 | 472.14 | 120038197 |
| 14 | rs3682013 | 477.21 | 121076354 |
| 14 | gnf14.117.278 | 477.39 | 122117119 |
| 14 | CEL-14-115300803 | 484.27 | 123368683 |
| 14 | rs13482416 | 484.31 | 125113758 |
| 15 | rs13459176 | 0.00 | 3229129 |
| 15 | rs13482431 | 22.13 | 11241218 |
| 15 | rs13482434 | 23.54 | 12093989 |
| 15 | rs13482446 | 34.65 | 14475237 |
| 15 | CEL-15-15919629 | 40.95 | 15940188 |
| 15 | CEL-15-17259939 | 41.00 | 17183572 |
| 15 | rs13482461 | 47.85 | 18859365 |
| 15 | rs13482464 | 50.75 | 19568361 |
| 15 | rs13482467 | 50.75 | 20394321 |
| 15 | rs13482473 | 55.72 | 21941791 |
| 15 | rs6367927 | 59.44 | 23607474 |
| 15 | rs13482486 | 67.28 | 25661842 |
| 15 | rs3670581 | 78.96 | 27000415 |
| 15 | rs13482498 | 84.35 | 28428133 |
| 15 | rs3088525 | 89.84 | 29562128 |
| 15 | rs4230683 | 107.54 | 30896769 |
| 15 | rs6333696 | 108.85 | 32047788 |
| 15 | rs3091174 | 111.70 | 32625941 |
| 15 | rs6188239 | 112.55 | 34260987 |
| 15 | rs3089995 | 116.21 | 35545928 |
| 15 | rs3695416 | 130.09 | 38414152 |
| 15 | rs3677296 | 131.55 | 39941392 |
| 15 | rs13482536 | 136.27 | 41700827 |
| 15 | rs13482541 | 137.90 | 43373243 |
| 15 | CEL-15-44698021 | 138.90 | 44507024 |
| 15 | rs4138760 | 148.54 | 46186595 |
| 15 | rs13482558 | 148.79 | 46998205 |
| 15 | CEL-15-48446430 | 149.54 | 48256300 |
| 15 | rs3660290 | 159.51 | 50688749 |
| 15 | rs3692040 | 159.51 | 50907093 |
| 15 | rs6165881 | 160.67 | 51649192 |
| 15 | rs13482580 | 161.27 | 52854120 |
| 15 | rs13482589 | 169.35 | 54732921 |
| 15 | rs6400804 | 181.40 | 56637986 |
| 15 | rs13482595 | 189.07 | 58448078 |
| 15 | rs3654559 | 194.74 | 59794025 |
| 15 | rs3683495 | 195.60 | 61034057 |
| 15 | rs13482612 | 213.47 | 63933997 |
| 15 | rs13482618 | 215.46 | 65690150 |
| 15 | rs6197332 | 225.53 | 66838064 |
| 15 | rs13482628 | 226.74 | 67708701 |
| 15 | rs13482635 | 236.98 | 69276656 |
| 15 | rs13482637 | 241.88 | 70331556 |
| 15 | rs6204870 | 241.88 | 71347761 |
| 15 | rs13482642 | 243.92 | 72042386 |
| 15 | rs13482654 | 262.65 | 76524736 |
| 15 | rs3660192 | 266.81 | 77655020 |
| 15 | rs3721372 | 271.18 | 80236058 |
| 15 | rs6276391 | 275.15 | 81657104 |
| 15 | rs4230908 | 280.63 | 82956832 |
| 15 | rs3660367 | 284.79 | 84532537 |
| 15 | rs4230930 | 289.79 | 85688903 |
| 15 | rs13482691 | 299.02 | 86803133 |
| 15 | rs13482693 | 313.76 | 87908143 |
| 15 | rs3720931 | 328.08 | 89328896 |
| 15 | rs6287697 | 335.06 | 90596958 |
| 15 | rs13482711 | 348.09 | 91889319 |
| 15 | CEL-15-93579770 | 352.48 | 92992122 |
| 15 | rs13482719 | 357.90 | 93937416 |
| 15 | rs6285067 | 364.98 | 95144976 |
| 15 | rs13482726 | 371.10 | 95780106 |
| 15 | rs13482729 | 376.52 | 96941901 |
| 15 | rs13482732 | 382.11 | 97961248 |
| 15 | rs3708604 | 387.12 | 98955890 |
| 15 | CEL-15-100463048 | 387.31 | 99832039 |
| 15 | rs13482741 | 391.03 | 100949743 |
| 16 | rs4152386 | 0.00 | 3987017 |
| 16 | rs4152838 | 1.26 | 5029543 |
| 16 | rs4165119 | 61.42 | 20498908 |
| 16 | rs4165287 | 62.83 | 22028919 |
| 16 | rs4165425 | 71.80 | 23797302 |
| 16 | rs6294027 | 75.19 | 25709024 |
| 16 | rs4165602 | 85.85 | 27436402 |
| 16 | rs4166607 | 90.30 | 28564407 |
| 16 | rs4167574 | 97.10 | 29670125 |
| 16 | rs4167955 | 106.55 | 30294144 |
| 16 | rs4168890 | 110.55 | 31242827 |
| 16 | rs4170074 | 111.80 | 32194865 |
| 16 | rs4171440 | 116.52 | 33481261 |
| 16 | rs4172338 | 121.14 | 34723517 |
| 16 | rs4173709 | 131.64 | 36515545 |
| 16 | rs4174469 | 137.23 | 38170827 |
| 16 | rs4175608 | 145.49 | 39182601 |
| 16 | rs4177203 | 149.87 | 40235897 |
| 16 | rs4178513 | 154.54 | 41700977 |
| 16 | rs4179117 | 159.84 | 42732985 |
| 16 | rs4179233 | 159.84 | 43610878 |
| 16 | rs4180126 | 159.84 | 44662109 |
| 16 | rs4182243 | 172.32 | 46052769 |
| 16 | rs4183448 | 172.76 | 47197561 |
| 16 | rs4184315 | 172.86 | 48666415 |
| 16 | rs4185176 | 182.46 | 49891965 |
| 16 | rs4185639 | 183.16 | 50298727 |
| 16 | rs3696661 | 202.17 | 51676815 |
| 16 | rs6164817 | 202.17 | 53030825 |
| 16 | rs3687272 | 205.67 | 54656364 |
| 16 | rs6271301 | 208.41 | 56024196 |
| 16 | rs4189277 | 208.51 | 57292596 |
| 16 | rs4190414 | 208.51 | 58539260 |
| 16 | rs4192132 | 216.30 | 59644430 |
| 16 | rs4192837 | 221.60 | 60328964 |
| 16 | rs4197416 | 234.64 | 66406352 |
| 16 | rs4197725 | 238.63 | 67382247 |
| 16 | rs4199410 | 253.28 | 70195004 |
| 16 | rs4202372 | 267.39 | 72909885 |
| 16 | rs4203891 | 269.90 | 74084951 |
| 16 | rs4205524 | 275.08 | 75296782 |
| 16 | rs4206648 | 287.79 | 76657843 |
| 16 | rs4207452 | 288.11 | 77429428 |
| 16 | rs4211515 | 301.91 | 82393734 |
| 16 | rs4211731 | 305.23 | 83406629 |
| 16 | rs4212102 | 312.24 | 85097433 |
| 16 | rs4219239 | 353.85 | 91670409 |
| 16 | rs4219897 | 363.16 | 92790232 |
| 16 | rs4220529 | 374.42 | 94664455 |
| 16 | rs4220927 | 376.46 | 95226900 |
| 16 | rs3164088 | 376.56 | 96537690 |
| 17 | rs3724616 | 0.00 | 3000665 |
| 17 | rs6310831 | 0.00 | 3227819 |
| 17 | rs3686875 | 6.92 | 4366843 |
| 17 | rs13482854 | 20.48 | 7341324 |
| 17 | rs3090641 | 23.98 | 9250458 |
| 17 | rs3702484 | 30.29 | 9881216 |
| 17 | rs13482870 | 33.68 | 10917415 |
| 17 | rs13482875 | 38.64 | 11996762 |
| 17 | rs3723317 | 43.49 | 13855019 |
| 17 | rs13482887 | 43.49 | 14731878 |
| 17 | rs3675740 | 51.51 | 16396667 |
| 17 | rs3721884 | 55.22 | 17710047 |
| 17 | mCV25045820 | 58.83 | 19078136 |
| 17 | rs6341891 | 58.83 | 20363470 |
| 17 | CEL-17-21229557 | 58.83 | 22653181 |
| 17 | rs4231344 | 68.16 | 23811928 |
| 17 | rs3090500 | 68.76 | 25480235 |
| 17 | rs4136360 | 75.97 | 26322926 |
| 17 | rs6358703 | 85.99 | 28916155 |
| 17 | rs6308773 | 86.04 | 29318855 |
| 17 | gnf17.027.979 | 90.03 | 30582925 |
| 17 | rs13482947 | 106.16 | 31769796 |
| 17 | rs3705058 | 113.43 | 33155342 |
| 17 | gnf17.035.152 | 115.41 | 35192178 |
| 17 | rs3682923 | 115.85 | 35971077 |
| 17 | rs13482968 | 117.42 | 37268628 |
| 17 | rs13482973 | 117.42 | 38319110 |
| 17 | rs6395893 | 117.42 | 39314866 |
| 17 | rs3690398 | 118.63 | 40980351 |
| 17 | CEL-17-40073719 | 120.77 | 42064538 |
| 17 | rs4231494 | 122.71 | 43226181 |
| 17 | rs13482997 | 126.54 | 43961585 |
| 17 | rs3090988 | 129.22 | 46892981 |
| 17 | rs13483011 | 140.65 | 48549496 |
| 17 | mCV22888090 | 261.41 | 63948053 |
| 17 | rs13483075 | 272.13 | 66071696 |
| 17 | rs13483078 | 274.54 | 67111530 |
| 17 | gnf17.069.331 | 280.97 | 68378508 |
| 17 | rs3657117 | 283.60 | 69398842 |
| 17 | rs6210797 | 298.69 | 70050732 |
| 17 | rs6176494 | 300.93 | 71101565 |
| 17 | rs6322076 | 306.94 | 72598864 |
| 17 | rs13483101 | 309.09 | 73689870 |
| 17 | rs13465627 | 313.89 | 74787631 |
| 17 | rs3710559 | 315.04 | 75674110 |
| 17 | rs13483110 | 327.26 | 76561295 |
| 17 | rs6386440 | 336.89 | 78754732 |
| 17 | rs3684732 | 395.58 | 83407153 |
| 17 | rs3701338 | 405.32 | 84406258 |
| 17 | rs13483144 | 423.31 | 86285312 |
| 17 | rs3723150 | 438.00 | 88951898 |
| 17 | rs13483159 | 449.90 | 90120158 |
| 18 | rs13483183 | 0.00 | 3516539 |
| 18 | CEL-18-4270744 | 0.00 | 4100246 |
| 18 | gnf18.002.818 | 3.70 | 5772955 |
| 18 | rs3680906 | 3.71 | 6681132 |
| 18 | rs13483200 | 24.79 | 9902165 |
| 18 | rs13483210 | 27.42 | 11941011 |
| 18 | rs13483215 | 27.77 | 13229156 |
| 18 | mCV23617245 | 35.68 | 14689061 |
| 18 | rs13483223 | 40.18 | 15600329 |
| 18 | rs13483226 | 46.62 | 16226038 |
| 18 | rs13483228 | 47.73 | 16944846 |
| 18 | rs13483235 | 53.51 | 18851806 |
| 18 | rs6303064 | 53.51 | 19758792 |
| 18 | rs6194744 | 61.37 | 22017938 |
| 18 | rs6368237 | 62.68 | 23599008 |
| 18 | rs13483256 | 64.14 | 24789900 |
| 18 | rs13483262 | 70.23 | 26291550 |
| 18 | rs3722205 | 71.91 | 27839338 |
| 18 | rs13483277 | 77.87 | 30522237 |
| 18 | CEL-18-32158369 | 81.87 | 31860936 |
| 18 | rs6385006 | 82.31 | 32689438 |
| 18 | UT-18-33.980716 | 85.37 | 33955610 |
| 18 | rs3675531 | 94.14 | 35204414 |
| 18 | gnf18.033.953 | 99.08 | 36663058 |
| 18 | rs6230993 | 103.52 | 39622092 |
| 18 | rs13483319 | 110.06 | 41067707 |
| 18 | rs6313313 | 111.11 | 41821108 |
| 18 | rs3676196 | 126.01 | 43083464 |
| 18 | rs13483331 | 134.14 | 44011475 |
| 18 | rs4138936 | 136.35 | 45088844 |
| 18 | rs13483340 | 139.57 | 46215512 |
| 18 | rs6184541 | 142.37 | 47598812 |
| 18 | rs3684561 | 142.40 | 48822666 |
| 18 | rs13483356 | 157.76 | 50467095 |
| 18 | CEL-18-52103795 | 158.87 | 51783321 |
| 18 | gnf18.051.412 | 159.27 | 53262641 |
| 18 | CEL-18-55824525 | 174.82 | 55486266 |
| 18 | rs3654438 | 180.41 | 56457195 |
| 18 | rs13483378 | 188.63 | 57453544 |
| 18 | rs13483382 | 197.62 | 58554775 |
| 18 | CEL-18-60214752 | 203.50 | 59858816 |
| 18 | rs13483390 | 204.55 | 61033381 |
| 18 | rs13483394 | 214.33 | 62112817 |
| 18 | rs13483398 | 218.21 | 63246689 |
| 18 | rs3688789 | 231.16 | 64674000 |
| 18 | rs13483409 | 252.25 | 66039597 |
| 18 | rs3716803 | 258.86 | 67157702 |
| 18 | rs13483417 | 264.16 | 68040979 |
| 18 | rs13483423 | 282.72 | 69689996 |
| 18 | rs3675825 | 285.56 | 70854303 |
| 18 | gnf18.069.928 | 289.73 | 71804268 |
| 18 | CEL-18-73439948 | 294.12 | 73070072 |
| 18 | rs6302629 | 307.69 | 75116177 |
| 18 | rs13483444 | 307.88 | 76297243 |
| 18 | rs3705890 | 316.09 | 77108822 |
| 18 | rs3671707 | 341.70 | 81003049 |
| 18 | rs13483466 | 369.55 | 82409997 |
| 19 | rs3713033 | 0.00 | 5029790 |
| 19 | CEL-19-8529644 | 6.83 | 9505052 |
| 19 | rs13483526 | 17.01 | 10670707 |
| 19 | rs6316813 | 30.17 | 11396714 |
| 19 | rs6307076 | 43.71 | 14626637 |
| 19 | rs13483541 | 49.54 | 15843736 |
| 19 | rs8267392 | 68.63 | 17749922 |
| 19 | UT-19-18.800709 | 74.10 | 18713052 |
| 19 | rs6342493 | 107.78 | 22707529 |
| 19 | rs13483569 | 107.86 | 23846117 |
| 19 | rs3720897 | 121.49 | 25196437 |
| 19 | rs13483577 | 126.05 | 26429767 |
| 19 | rs6291559 | 137.06 | 27647714 |
| 19 | rs3714482 | 155.10 | 29011879 |
| 19 | UT-19-29.979736 | 159.05 | 30313714 |
| 19 | mCV24505422 | 159.05 | 30739933 |
| 19 | CEL-19-32349880 | 194.01 | 33111772 |
| 19 | rs13483599 | 194.21 | 34469905 |
| 19 | rs13483601 | 196.41 | 35024278 |
| 19 | rs13483606 | 207.83 | 36131092 |
| 19 | mCV23121032 | 240.76 | 41054337 |
| 19 | rs4232174 | 242.22 | 41841036 |
| 19 | rs13483650 | 306.42 | 47447898 |
| 19 | CEL-19-48242857 | 319.85 | 48972882 |
| 19 | rs6194426 | 321.27 | 50203520 |
| 19 | rs3718102 | 330.20 | 52338597 |
| 19 | rs13483677 | 338.11 | 53933993 |
| 19 | rs3654713 | 346.57 | 55093406 |
| 19 | rs3686750 | 370.66 | 56422639 |
| 19 | rs6211533 | 373.28 | 57066899 |
| 19 | rs3711945 | 383.02 | 58785854 |
