# Supplementary material for: Genetic Effects at Pleiotropic Loci Are Context-Dependent with Consequences for the Maintenance of Genetic Variation in Populations
Source: PLoS Genet. 2011 Sep 8;7(9):e1002256. doi: 10.1371/journal.pgen.1002256 (PMC3169520; doi:10.1371/journal.pgen.1002256)
Supplement: Table S6 — Chromosome-wise and genome-wide significance thresholds. (DOC) [file pgen.1002256.s008.doc]

| **Chromosome** | **Threshold** |
| --- | --- |
| 1 | 2.9243 |
| 2 | 2.8062 |
| 3 | 2.8062 |
| 4 | 2.7924 |
| 5 | 2.7482 |
| 6 | 2.8062 |
| 7 | 2.7160 |
| 8 | 2.5051 |
| 9 | 2.6990 |
| 10 | 2.6812 |
| 11 | 2.7482 |
| 12 | 2.6435 |
| 13 | 2.6990 |
| 14 | 2.4472 |
| 15 | 2.6812 |
| 16 | 2.5798 |
| 17 | 2.5315 |
| 18 | 2.6435 |
| 19 | 2.4472 |
| **Genome-wide** | **3.9759** |
