# Supplementary material for: Genetic Effects at Pleiotropic Loci Are Context-Dependent with Consequences for the Maintenance of Genetic Variation in Populations
Source: PLoS Genet. 2011 Sep 8;7(9):e1002256. doi: 10.1371/journal.pgen.1002256 (PMC3169520; doi:10.1371/journal.pgen.1002256)
Supplement: Table S7 — Significant crossing interactions at MetS QTL. (DOC) [file pgen.1002256.s009.doc]

| **QTL** | **Trait** | **Level of Interaction** | **p-value** | **Cohort (se)** | **LL genotypic value** | **SS genotypic value** |
| --- | --- | --- | --- | --- | --- | --- |
| *DMetS6c* | Glucose (mg/dL) | additive*diet | 0.0029 | Low-fat fed females (5.6) | 209.74 | 170.00 |
|  |  |  |  | High-fat fed females (6.4) | 182.53 | 201.77 |
| *DMetS8b* | Cholesterol (mg/dL) | additive*sex | 0.0048 | High-fat fed females (3.7) | 187.98 | 163.59 |
|  |  |  |  | High-fat fed males (4.1) | 179.11 | 192.28 |
| *DMetS15a* | Area Under Curve (10wks) | additive*diet | 0.0001 | Low-fat diet (264.0) | 6280.79 | 7302.06 |
|  |  |  |  | High-fat diet (448.8) | 14313.83 | 11854.17 |
| *DMetS16a* | Insulin (mg/dL) | additive*sex | 0.0041 | Females (.14) | 3.00 | 2.37 |
|  |  |  |  | Males (.19) | 4.55 | 5.06 |
